# Characterization of the glutathione S‐transferase genes in the sand flies *Phlebotomus papatasi* and *Lutzomyia longipalpis* shows expansion of the novel glutathione S‐transferase *xi* (X) class

**DOI:** 10.1111/imb.12769

**Published:** 2022-03-08

**Authors:** Faisal Ashraf, Gareth D. Weedall

**Affiliations:** ^1^ School of Biological and Environmental Sciences Liverpool John Moores University Liverpool UK

**Keywords:** detoxification, insecticide resistance, Leishmaniasis, *Lutzomyia longipalpis*, metabolic resistance, *Phlebotomus papatasi*, sand flies

## Abstract

Leishmaniasis control often relies upon insecticidal control of phlebotomine sandfly vector populations. Such methods are vulnerable to the evolution of insecticide resistance via a range of molecular mechanisms. There is evidence that two major resistance mechanisms, target site insensitivity and metabolic resistance, have evolved in some sandfly populations and further genetic characterization of resistance would be useful to understand and combat it. To facilitate the study of the mechanisms of metabolic resistance, here we improved the annotation and characterized a major detoxification gene family, the glutathione‐s‐transferases (GST), in the genomes of two sand fly species: *Phlebotomus papatasi* and *Lutzomyia longipalpis*. The compositions of the GST gene family differ markedly from those of *Aedes* and *Anopheles* mosquitoes. Most strikingly, the *xi* (X) class of GSTs appears to have expanded in both sand fly genomes. Our results provide a basis for further studies of metabolic resistance mechanisms in these important disease vector species.

## Graphical abstract

- By extensive manual editing of genome annotations, we determined the complement of glutathione S‐transferase genes in two phlebotomine sand fly species: *Phlebotomus papatasi* and *Lutzomyia longipalpis*.
- Major differences in GST composition were seen compared with mosquito and fruit fly genomes, especially in classes associated with insecticide resistance.
- A novel GST class (*xi*) is expanded in both sand fly species, while *delta* and *epsilon* GST classes were reduced or absent.

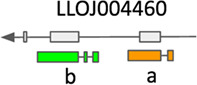

Schematic diagram showing results of manual editing of the gene model of a putative GST gene (LLOJ004460) in the genome of *Lutzomyia longipalpis*. The existing, incorrect, annotation is shown in grey and the modified annotations (splitting the gene into two genes: 'a' and 'b') in green and orange.

## INTRODUCTION

The Leishmaniases, a group of neglected parasitic diseases caused by *Leishmania* parasites, are transmitted by a number of species of phlebotomine sand fly (Burza et al., 2018). Leishmaniases are neglected diseases, often associated with poverty, that collectively exert a large disease burden in over 100 countries. Disease control methods differ in different contexts, though a major pillar of Leishmaniasis control relies upon control of its sand fly vectors (Wilson et al., 2020). However, insecticidal control methods are vulnerable to the evolution of insecticide resistance, so understanding the mechanisms of resistance is important for identifying, tracking and tackling it.

Insecticide resistance can evolve in a number of ways. One is via mutations in the genes encoding the insecticides' target proteins that render the insecticides ineffective (so‐called ‘target site resistance’). Another is via increased metabolism and removal of the insecticide (‘metabolic resistance’). Insecticides of the pyrethroid class (used in impregnated bednets and house spraying) and the organochlorine class (DDT, used in house spraying) both target the voltage‐gated sodium channel (VGSC) on the surface of neurons, while carbamate and organophosphate class insecticides target the acetylcholinesterase enzyme. Metabolic resistance is commonly due to overexpression of members of large multi‐gene families encoding detoxification enzymes such as cytochrome P450 monooxygenases (CYP), glutathione S‐transferases (GST) and carboxylesterases. Both target site and metabolic resistance are widespread in insects (Ffrench‐Constant, 2013) and threaten disease control programmes (Hemingway, 2018).

GSTs play roles in a number of endogenous processes such as hormone biosynthesis and intracellular transport, and they are critical in the detoxification of xenobiotic compounds (Ketterman et al., 2011). GSTs can confer resistance by direct mechanisms – metabolizing and sequestering toxic compounds – and indirect mechanisms – protecting against oxidative stress caused by exposure to the insecticide (Ketterman et al., 2011; Pavlidi et al., 2018). The GSTs form a superfamily, with different GST classes categorized based on sequence similarity. Two broad groups of GSTs – microsomal and cytosolic – are found in insects. Among the cytosolic GSTs, classes *omega* (GSTO), *sigma* (GSTS), *theta* (GSTT), *zeta* (GSTZ) are found ubiquitously in animals. In addition, in insects and some other arthropods, the GST classes *delta* (GSTD) and *epsilon* (GSTE) are found (Enayati et al., 2005; Ketterman et al., 2011) and are often expanded to outnumber the other GST classes (Ranson et al., 2002). Two additional classes, *xi* (GSTX) and *iota* (GSTI), were identified (with 2 and 1 members, respectively) and suggested to be specific to *Aedes* and *Anopheles* mosquitoes (Lumjuan et al., 2007). Insecticide resistance has most commonly been associated with the insect‐specific, cytosolic GSTD and GSTE classes (Enayati et al., 2005). Multiple lines of evidence have confirmed certain GSTs, such as the *epsilon* class GSTE2 in *Anopheles* and *Aedes* mosquitoes, as major drivers of insecticide resistance. Elevated expression of GSTE2 is seen in DDT‐resistant populations of *Anopheles funestus* (Riveron et al., 2014; Weedall et al., 2019; Kouamo et al., 2021), *Anopheles gambiae* (Ranson et al., 2001) and *Aedes aegypti* (Lumjuan et al., 2005). Population genetic analyses show GSTE loci evolving under strong directional selection in *An. funestus* (Weedall et al., 2020) and *An. gambiae* (Anopheles gambiae 1000 Genomes Consortium, 2017). Recombinant protein expression and *in vitro* biochemical characterization of GSTE2 allelic variants show different abilities to metabolize DDT (Lumjuan et al., 2005; Riveron et al., 2014; Mitchell et al., 2014), explained by protein structure analyses (Wang et al., 2008; Riveron et al., 2014; Mitchell et al., 2014). Heterologous *in vivo* expression of GSTE2 in *Drosophila melanogaster* (Riveron et al., 2014; Mitchell et al., 2014) has validated its role in conferring resistance. *In vivo* RNAi gene silencing of a number of GSTE genes in addition to GSTE2 in *An. funestus* (Kouamo et al., 2021) and *Ae. aegypti* (Lumjuan et al., 2011) has also implicated them in resistance to the pyrethroid deltamethrin. In addition to *delta* and *epsilon* classes, GSTS (Yamamoto et al., 2007; Gawande et al., 2014; Hassan et al., 2019) and possibly GSTX (Grant & Hammock, 1992; Lumjuan et al., 2007) have also been associated with insecticide resistance by similar experimental approaches.

Though little‐studied compared with aedine and anopheline mosquitoes, insecticide resistance has been reported in sand fly populations subjected to long‐term insecticide exposure. In India, Bangladesh and Nepal, where *P. papatasi* and *P. argentipes* transmit cutaneous and visceral Leishmaniasis, respectively, and DDT has been used since the 1950s in disease control programmes, DDT resistance has been reported in *P. papatasi* populations, reviewed by Dhiman & Yadav (2016). Target site mutations in the VGSC have been detected in *P. argentipes* in India (Dhiman & Yadav, 2016; Gomes et al., 2017; Sardar et al., 2018) and Sri Lanka (Pathirage et al., 2020), where elevated GST and esterase activity was also reported. Target site mutations in the VGSC have also been reported in a *Phlebotomus papatasi* population in Turkey (Fotakis et al., 2018). Reduced mortality to deltamethrin and permethrin was seen in sand flies in a region of Turkey with long‐term exposure but full susceptibility in another region without exposure (Karakus et al., 2017). Similarly in Sudan, *P. papatasi* in regions of historical insecticide exposure showed resistance to malathion and propoxur (Hassan et al., 2012). In Brazil, *Lutzomyia longipalpis* populations exposed to insecticides showed reduced mortality in bioassays (Alexander et al., 2009; Pathirage et al., 2020).

To understand the evolution and molecular mechanisms of metabolic resistance in sand flies, it is important to first characterize the major detoxification enzyme families. An important first step in this effort is manual editing and curation of the annotation of these gene families in genome assemblies (Weedall et al., 2015). Here, we defined one of these gene families, the glutathione s‐transferases (GST), in the *P. papatasi* and *L. longipalpis* genomes and compared its composition with that seen in other disease vector species. The composition of the GST gene family is very different in the sand flies, with a major expansion of the insect‐specific GSTX subfamily, which may influence resistance evolution in these species.

## RESULTS

**FIGURE 1:**
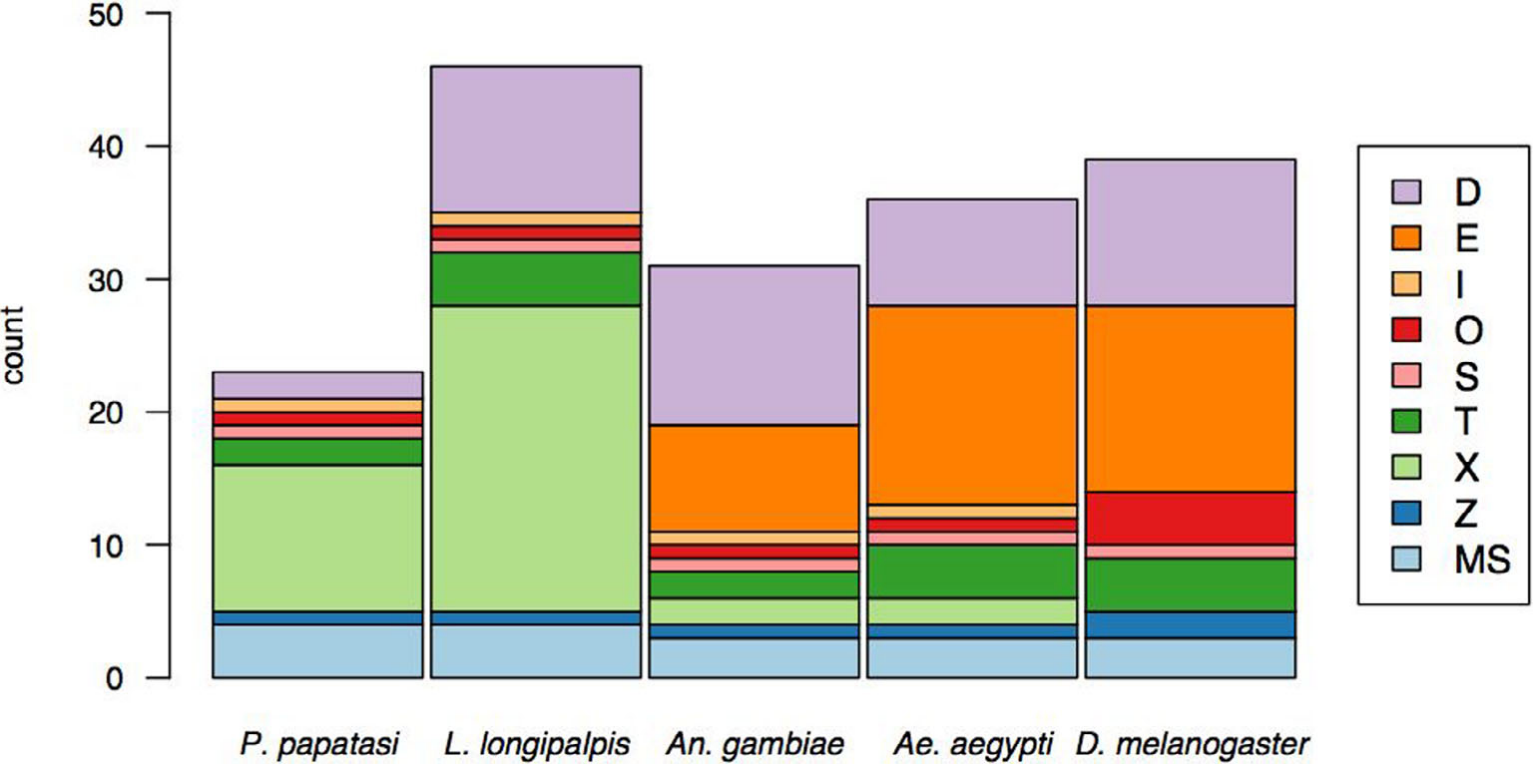
GST complements of the sand flies, compared *Anopheles gambiae*, *Aedes aegypti* and *Drosophila melanogaster*. Stacked bar charts of counts of genes belonging to each of the GST classes. The most striking contrast between the sand flies and the other species is the absence of the GSTE class (orange) and the expansion of the GSTX class (pale green)

Glutathione S‐Transferase (GST) proteins from the well‐annotated mosquitoes *Aedes aegypti* and *Anopheles gambiae* were used to search the predicted proteomes of *P. papatasi* and *L. longipalpis* using BLASTp. Putative GST proteins were carefully inspected and compared with annotated GSTs to assess and manually edit their gene models and produce an improved annotation and an inventory of GST genes in each of the sand fly genomes. This resulted in 23–25 GST genes annotated in the *P. papatasi* genome and 44–47 in the *L. longipalpis* genome. Table 1 and Figure 1 summarize these results. Full details of the gene edits and gene‐wise information are shown in Table S1 (for *P. papatasi*), Table S2 (for *L. longipalpis*) and Appendix S1 (gene edits in both genomes).

**TABLE 1:** Numbers of GST gene families identified in the two sand fly genomes compared with two mosquito species and the fruit fly *D. melanogaster*

GST family	*P. papatasi*	*L. longipalpis*	*An. gambiae*	*Ae. aegypti*	*D. melanogaster*
*delta* (D)	2	11	12–15^a^	8	11
*epsilon* (E)	0	0	8	15^b^	14
*iota* (I)	1	1–3^c^	1	1	0–1^d^
*omega* (O)	1	1	1	1	4
*sigma* (S)	1	1–2	1	1	1
*theta* (T)	2–3	4	2	4	4
*xi* (X)	11–12^e^	23	2	2	0
*zeta* (Z)	1^f^	1	1	1	2
microsomal (MS)	4	4	3	3–4^g^	3
Total	23–25	44–47	31–34	33–34	39–40

^a^ Three genes on unlocalized scaffolds show >98% amino acid identity with genes on chromosomes and are potentially allelic rather than paralogous.

^b^ In the *Ae. aegypti* reference genome, tandem duplications within the *epsilon* cluster have produced two extra copies each of GSTE2 and GSTE5 and three extra copies of GSTE7 – without these there are eight paralogous GSTE genes.

^c^ Three adjacent, partial genes, possibly all belong to a single gene.

^d^ High level of similarity to part of the GST‐containing FLYWCH zinc‐finger protein, *gfzf*.

^e^ Eleven complete coding sequences could be reconstructed, and additional gene could not be resolved into a complete coding sequence.

^f^ One putative gene made by joining two annotated partial genes on different scaffolds.

^g^ Gene AAEL023181, on an unlocalized scaffold, is identical to AAEL010157 and may be allelic rather than paralogous.

The most striking differences between the sand fly genomes and those of fellow nematocera *Aedes aegypti* and *Anopheles gambiae* mosquitoes and the more distantly‐related brachyceran *Drosophila melanogaster* fruit flies are the apparent complete absence of the *epsilon* class of GSTs from both sand fly genomes, the small number of *delta* GSTs in *P. papatasi* (though not *L. longipalpis*) and an apparent expansion of the GSTX class (absent from *Drosophila melanogaster* and present in only two copies in each mosquito species genome). The large difference between the two sandfly species in the overall number of GST genes, almost twice as many in *L. longipalpis* than in *P. papatasi*, is mostly due to the greater number of *delta‐* and *xi‐*class genes in *L. longipalpis* (Table 1, Figure 1). Each GST class is described in more detail below.

### *GST* delta*,* epsilon *and* xi

The GST complements of *D. melanogaster*, *Aedes aegypti* and *Anopheles gambiae* are dominated by expanded GSTD and GSTE classes. These insect‐specific classes tend to occur in gene clusters (Ranson et al., 2002) and members of these classes are commonly associated with insecticide resistance (Han et al., 2016; Hu et al., 2014; Lu et al., 2016; Kouamo et al., 2021; Lumjuan et al., 2005, 2011; Mitchell et al., 2014; Ranson et al., 2001; Riveron et al., 2014; Zhang et al., 2016).

No putative GSTE genes were identified in either the *P. papatasi* or the *L. longipalpis* genome. The best matches based on reciprocal BLASTp searches using GSTE proteins as queries were to proteins defined as GSTD and GSTX.

Six genes (PPAI001211, PPAI006595, LLOJ007285, LLOJ004462, LLOJ004461, LLOJ004460) were identified as putative GSTD. Exon numbers and predicted protein sizes varied greatly among these genes and the gene models were carefully inspected. For *P. papatasi*, the PPAI001211 gene model was left unchanged and PPAI006595 edited to add two exons (new gene model denoted PPAI006595_a). For *L. longipalpis*, LLOJ007285 and LLOJ004462 were left unchanged. One gene (LLOJ004461) was very long and clearly consisted of multiple individual genes misanotated as a single gene. This was edited to split the gene and add and extend exons into seven complete individual genes (LLOJ004461_a–LLOJ004461_g). LLOJ004460 was edited and split into two incomplete genes (LLOJ004460_a, LLOJ004460_b) spanning sequencing gaps. LLOJ004460_a had an internal gap (spanning part of the second and third of its three exons). LLOJ004460_b had a gap at its 3′/C‐terminal end that covered part of the third of the three exons. After this extensive manual editing (Appendix S1), two GSTD genes were annotated in the *P. papatasi* genome and 11 were annotated in the *L. longipalpis* genome (Table 1, Tables S1 and S2, Figure S1).

**FIGURE 2:**
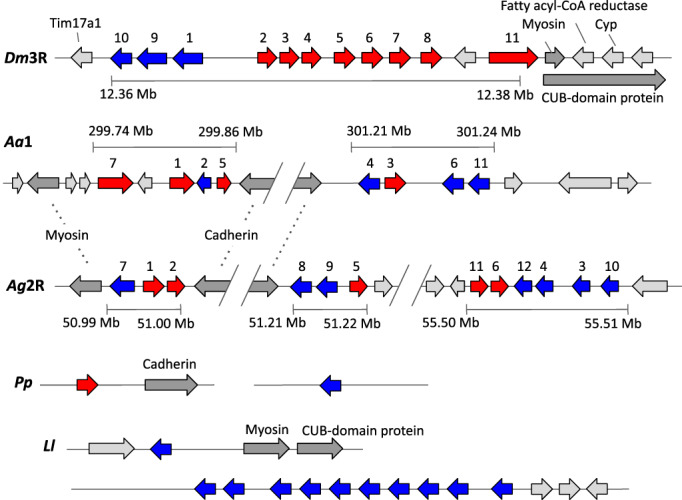
GSTD gene clusters on *D. melanogaster* 3R (*Dm*3R), *Ae. aegypti* chromosome 1 (*Aa*1) and *An. gambiae* 2R (*Ag*2R), compared with *P. papatasi* (*Pp*) and *L. longipalpis* (*Ll*) GSTD genes. GSTD genes are shown in blue (negative strand) and red (positive strand), with their numbers shown above the genes. Approximate positions of the clusters on each chromosome arm are shown. Light grey arrows show non‐GST flanking genes with no clear orthology to other flanking genes. Dark grey arrows indicate flanking genes with orthology to those in other species. Cyp = cytochrome P450 monooxygenase

GSTD genes occur in clusters in insect genomes. In *D. melanogaster*, one large cluster on chromosome 3R consists of GSTD10, D9, D1, D2, D3, D4, D5, D6, D7, D8, D11. The mosquito *Ae. aegypti* has two clusters on chromosome 1: GSTD7, D1, D2, D5 in one cluster and GSTD4, D3, D6, D11 in the other. *An. gambiae* has three clusters on chromosome 2R: GSTD7, D1, D2 in one, GSTD8, D9, D5 in another and GSTD11, D6, D12, D4, D3, D10 in another. The patterns seen in these species suggest a high rate of gene shuffling, with some conservation still detectable between more closely related mosquito species (e.g., GSTD7, D1, D2 in the two mosquitoes). The *D. melanogaster* GSTD cluster is flanked on one side by a gene encoding myosin light chain 2 V and a CUB‐domain containing gene (adjacent to GSTD11). Similarly, the *Ae. aegypti* cluster (GSTD7,1,2,5) and *An. gambiae* cluster (GSTD7,1,2) are both flanked by the myosin light chain 2 V gene on one side and a cadherin gene on the other (Figure 2). The sand fly GSTD genes were compared with these to try to elucidate their genomic distribution. In *P. papatasi*, PPAI001211 occurred on its own on scaffold JH662257.1; BLASTx identified no other genes on the scaffold. PPAI006595_a occurred on a scaffold with one other gene (BLASTx identified no other genes on the scaffold), a putative cadherin orthologous to cadherin genes that sit adjacent to GSTD5 (in *Ae. aegypti*) and GSTD2 (in *An. gambiae*), suggesting orthology with these two mosquito GSTD clusters (Figure 2). In *L. longipalpis*, LLOJ007285 occurred adjacent to a myosin light chain 2 V gene and a CUB‐domain containing gene, suggesting orthology with *Dm*GSTD11, or with *Aa*GSTD7 and *Ag*GSTD7. The large cluster of 10 *Ll*GSTD genes was flanked on one side by genes showing no orthology to flanking genes seen in the other species, so no clear inference can be drawn (Figure 2).

Two GST genes originally annotated as ‘unclassified’ in *Anopheles gambiae* (Ding et al., 2003) and labelled ‘GSTu2’ and ‘GSTu3’ (the ‘u’ here represents ‘unclassified’ and is not to be confused with the plant‐specific *tau* class GSTU proteins) were later designated as members of a novel, putatively mosquito‐specific class, *xi* (X), in *An. gambiae* and *Aedes aegypti* (Ding et al., 2003; Lumjuan et al., 2007). Phylogenetically, the GSTX class falls between GSTD and GSTE classes (Ding et al., 2003; Lumjuan et al., 2007).

Twenty‐one genes (PPAI008305, PPAI010868, PPAI010869, PPAI010870, PPAI010871, PPAI010872, LLOJ001842, LLOJ009529, LLOJ009528, LLOJ010488, LLOJ010539, LLOJ010538, LLOJ010537, LLOJ010536, LLOJ010535, LLOJ010534, LLOJ010533, LLOJ010532, LLOJ010530, LLOJ010531, LLOJ010963) were identified as putative GSTX. The genes varied greatly in size and were extensively edited (Appendix S1). In *P. papatasi* the six genes were edited to produce 11 genes (a possible twelfth, PPAI010871_e, could be identified but not reconstructed into a full‐length gene). Ten of these genes (PPAI010868, PPAI010869, PPAI010870_a‐c, PPAI010871_a‐d, PPAI010872; 11 including PPAI010871_e) formed a gene cluster on scaffold JH660956.1 while a single, partial gene (PPAI008305_a) was found on scaffold JH665757.1, a very small scaffold with no other annotated genes on it. Therefore, the evidence suggested a single gene cluster of GSTX in *P. papatasi*. In *L. longipalpis* the 15 genes were edited to produce 23 genes. Two large gene clusters were reconstructed: seven genes (LLOJ001842_a‐g) on scaffold JH690728 and 15 genes (LLOJ009529, LLOJ009528_a‐b, LLOJ010488, LLOJ010539, LLOJ010538, LLOJ010537, LLOJ010536, LLOJ010535_a, LLOJ010534, LLOJ010533_a, LLOJ010532, LLOJ010530, LLOJ010531_a‐b) on scaffold JH690204, with a single gene (LLOJ010963) on scaffold JH689654 (Table 1, Tables S1 and S2, Figure S1).

**FIGURE 3:**
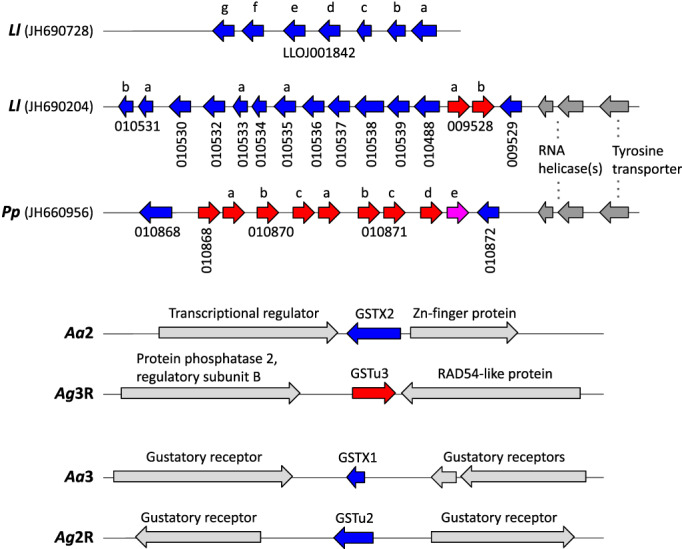
GSTX gene clusters in the *P. papatasi* (*Pp*) and *L. longipalpis* (*Ll*) genomes. Species and scaffold ID or chromosome are shown on the left (*L. longipalpis* = *Ll*; *P. papatasi* = *Pp*; *Ae. aegypti* = *Aa*; *An. gambiae* = *Ag*). GSTX genes are shown in blue (negative strand) and red (positive strand). For the sand flies, the gene code (prior to editing) is shown below each gene (for presentation purposes, the beginning of each is not shown: PPAI for *P. papatasi* and LLOJ for *L. longipalpis*) and a single letter above to distinguish genes (e.g., 010531 refers to gene LLOJ010531, which was edited to form two genes: LLOJ010531_a and LLOJ010531_b). PPAI010871_e is shown in pink as a complete in‐frame coding sequence could not be reconstructed for this gene. Light grey arrows show non‐GST flanking genes with no clear orthology to other flanking genes. Dark grey arrows indicate flanking genes with orthology to those in other species. For example, orthologous RNA helicase and tyrosine transporter genes downstream of major GSTX clusters in the sand fly genomes indicate they occur in orthologous genomic regions (though the orientation and order of GSTX genes indicates gain and loss of genes in the cluster)

The GSTX gene cluster in *P. papatasi* (PPAI010868‐PPAI010872) and the larger of the two gene clusters in *L. longipalpis* (LLOJ009529‐LLOJ010531_b) are orthologous, occurring adjacent to two annotated RNA helicase genes (PPAI010873/PPAI010874 and LLOJ009530/LLOJ009531) and a tyrosine transporter gene (PPAI010875 and LLOJ009532) at one end (Figure 3). At the other end of the *P. papatasi* cluster is a large region with no annotated genes followed by two genes (PPAI010866, PPAI010867) that may both be fragments of a single gene encoding a transmembrane protein; its apparent orthologue in *L. longipalpis* (LLOJ002194) occurs alone on a scaffold (JH690844). The *L. longipalpis* gene cluster (LLOJ009529‐LLOJ010531_b) runs to the end of the scaffold, with no non‐GST gene flanking it. The other *L. longipalpis* gene cluster (LLOJ001842_a‐g) occurs alone on a scaffold (JH690728), so its position relative to the other gene cluster (whether it is distinct or forms part of the same gene cluster) cannot be determined. Overall, the data suggest an expansion of the GSTX class in the lineage leading to the sand flies, concentrated in one large gene cluster in both *Phlebotomus* and *Lutzomyia* genera, with a possible second cluster in *Lutzomyia*. The GSTX cluster in the sand flies is not clearly orthologous to the location of either of the single GSTX genes in the mosquitoes, possibly due to gene loss or genomic rearrangement in one or other lineage (Figure 3).

**FIGURE 4:**
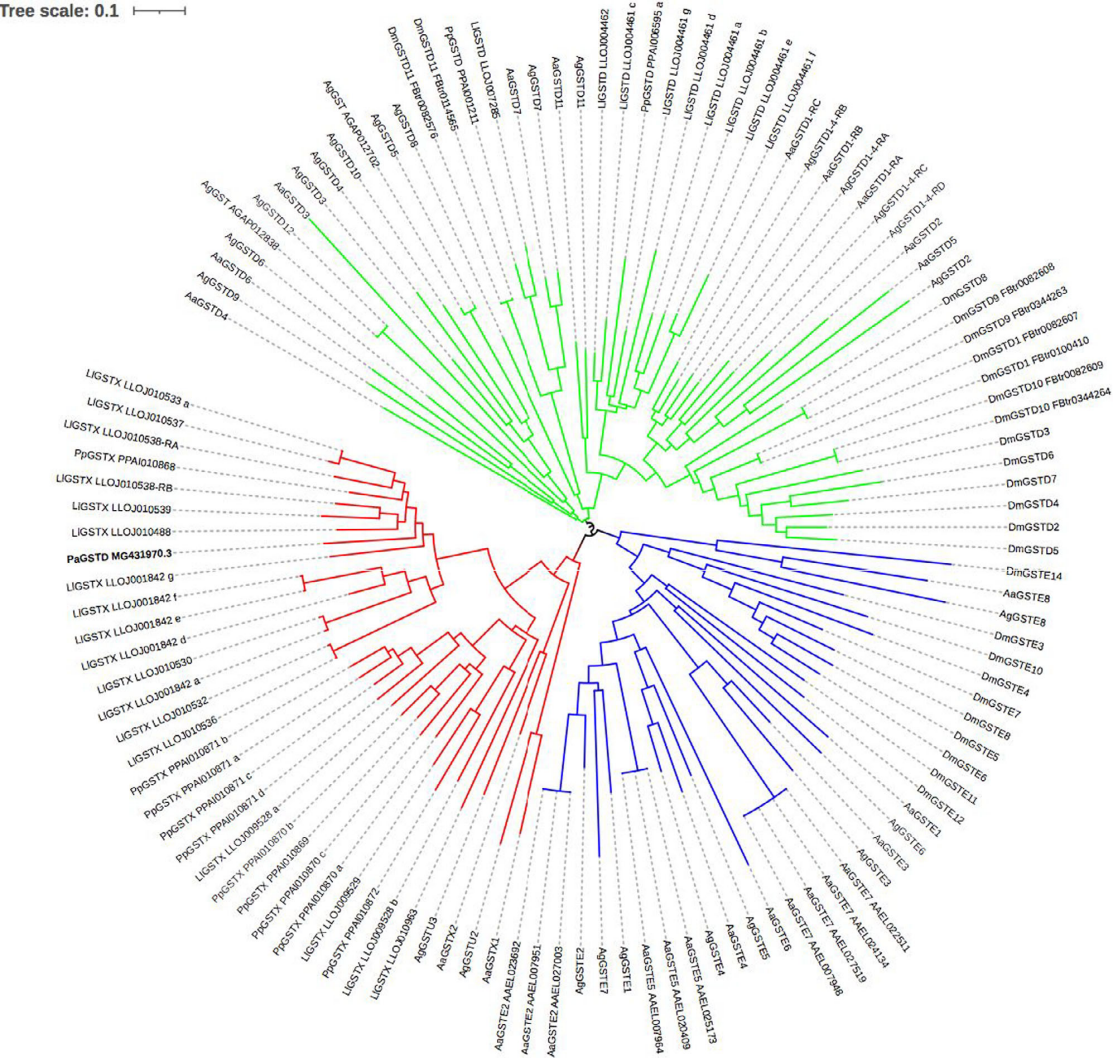
Phylogeny of GST classes *delta*, *epsilon* and *xi*. Neighbour‐joining phylogeny of GSTD, E and X proteins from *P. papatasi* (*Pp*), *L. longipalpis* (*Ll*), *An. gambiae* (*Ag*), *Ae. aegypti* (*Aa*) and *D. melanogaster* (*Dm*). Branches leading to the GSTDs are shown in green, to GSTEs in blue and to GSTXs in red. Protein isoforms are indicated by ‐RA, ‐RB and so forth (or a transcript ID for *Drosophila*). Gene IDs are included to distinguish proteins with the same names. Edited gene models in the sand flies are indicated by a, b, c and so forth. The tree shown is an NJ tree where gap‐containing sites were removed. Bootstrap support values for the branches basal to the GSTX, E and D classes were 75%, 60% and 46%, respectively (84%, 91% and 84% when gap‐containing sites were retained). A putative GSTD from the sand fly *Phlebotomus argentipes* is shown in bold. Its position in the tree indicates it is a GSTX

To further study the evolutionary relationship among the GSTE, D and X classes, a phylogeny of all of the proteins from these three classes from the two sand fly species, the mosquitoes *Ae. aegypti* and *An. gambiae* and the fruit fly *D. melanogaster* was constructed (Figure 4). Each GST class forms a separate clade in the tree and within the GSTX clade the sand fly GSTX proteins tend to cluster by species, with some exceptions. This pattern suggests recent independent GSTX gene expansions (or gene conversion among paralogous GSTX genes) in each sand fly lineage. For example, PPAI010870_a, _b, _c and _d all form part of a chromosomal gene cluster and all cluster together in the tree, suggesting they may have arisen by successive tandem duplication of genes (or gene conversion among adjacent genes of the cluster). Reflecting this pattern, the sand fly GSTX proteins showed greater similarity (in BLAST searches) to paralogous GSTXs than to any *An. gambiae* or *Ae. aegypti* GST genes: the closest matching *An. gambiae* or *Ae. aegypti* proteins were GSTX and GSTD in *D. melanogaster* (Tables S1 and S2). This pattern indicates that *D. melanogaster* lacks GSTX entirely and that GSTD is the next‐best match, as the percentage identity between sand fly GSTX and *Dm*GSTD was only around 40% compared with around 60% for sand fly GSTD versus *Dm*GSTD (Tables S1 and S2).

**FIGURE 5:**
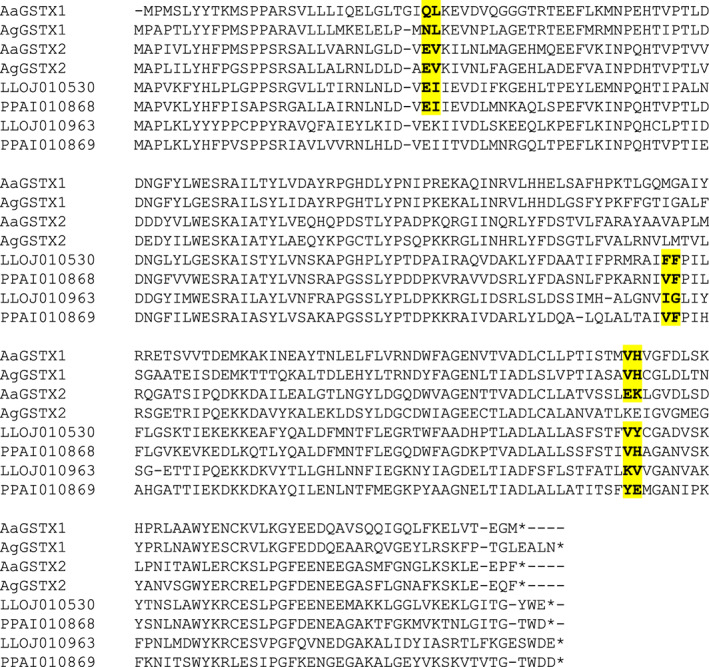
Intron–exon structure of GSTX proteins. An alignment of GSTX proteins is shown, with exon–exon junctions shown in bold and highlighted in yellow (including the amino acid immediately upstream and downstream of the junction). Of the sand fly GSTX genes, one representative protein of the 4‐exon gene structure and one of the 3‐exon structures is shown for each species. AgGSTX1 = AGAP003257 (originally annotated as GST unclassified 2, GSTU2); AgGSTX2 = AGAP009342 (originally annotated as GST unclassified 3, GSTU3); AaGSTX1 = AAEL000092; AaGSTX2 = AAEL010500. In the protein identifiers: AGAP = *Anopheles gambiae* PEST strain; AAEL = *Aedes aegypti* Liverpool strain; PPAI = *Phlebotomus papatasi* Israel strain; LLOJ = *Lutzomyia longipalpis* Jacobina strain; ‘‐PA’, ‘‐PB’ and so forth indicate protein isoforms of the same gene; ‘_a’, ‘_b’ and so forth (for the sand fly proteins only) indicate separate genes created by manual editing of a gene model

The *GstX* gene models differed between sand flies and mosquitoes (Figure 5). Mosquito GSTX genes typically had three exons and two introns (though *Ag*GSTU3 lacked the second intron). Sand fly GSTX genes typically had 3 or 4 exons (with more of the 3‐exon form in *P. papatasi* and more of the 4‐exon form in *L. longipalpis*). The 4‐exon form shared intron 1 and 2 positions with the mosquito GSTX genes but had an additional novel intron in what is exon 2 (of the mosquito GSTXs). The 3‐exon form lacked what is intron 1 (of the mosquito GSTXs) but had the novel intron and shared (mosquito) intron 2.

### *GST* iota*,* omega*,* sigma*,* theta*,* zeta *and microsomal GSTs*

A GST originally annotated as ‘unclassified’ in *Anopheles gambiae* (Ding et al., 2003) and labelled ‘GSTu1’ (the ‘u’ here represents ‘unclassified’ and is not to be confused with the plant‐specific *tau* class GSTU proteins) was later designated as a member of a novel class, *iota*, in *An. gambiae* and *Aedes aegypti* (Ding et al., 2003; Lumjuan et al., 2007). In the sand fly genomes, two genes (PPAI009870, LLOJ002711) were identified as putative GSTI. For *P. papatasi*, the PPAI009870 gene model was left unchanged. For *L. longipalpis*, LLOJ002711 was edited to form three incomplete genes (LLOJ002711_a ‐ LLOJ002711_c). The poor quality of the genome assembly at this region (the gene or genes crosses two sequencing gaps) mean that it is possible that these are not three distinct genes, but may be fragments of a single gene that is not fully collapsed in the assembly. This is supported by the observation that the overlapping regions are identical at the amino acid level (Appendix S1).

The best match in the *D. melanogaster* genome was to a ‘GST‐containing FLYWCH zinc‐finger protein’, *gfzf* (FBgn0250732) (Dai et al., 2004). The percentage identity was considerably higher to this gene (around 76%) than to the next‐best matches (around 44%). Two isoforms of the *gfzf* gene contain large exons upstream of the GST region that contain repeated FLYWCH zinc‐finger domains. One of these isoforms (from transcript FBtr0091512) was used to search the proteomes and genomes of the two sand flies in order to try to extend the gene models in those species. However, neither BLASTp nor tBLASTn searches could identify the non‐GST region of the gene. We therefore annotated the genes as GSTI, as defined by Lumjuan et al. (Lumjuan et al., 2007).

**FIGURE 6:**
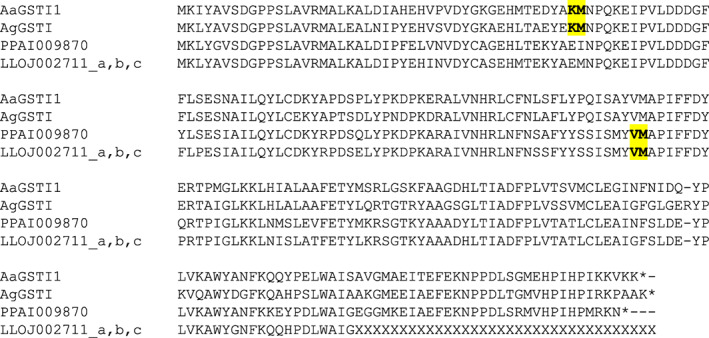
Intron–exon structure of GSTI proteins. An alignment of GSTI proteins is shown, with exon–exon junctions shown in bold and highlighted in yellow (including the amino acid immediately upstream and downstream of the junction). Three partial genes (LLOJ002711_a, _b and _c) from a poorly‐resolved region of the *L. longipalpis* genome, that were identical at the amino acid level in their overlapping regions were merged to form a single gene for the alignment (LLOJ002711_a,b,c). Unknown amino acids (due to genome assembly gaps) are represented by ‘X’. AgGSTI = AGAP000947 (originally annotated as GST unclassified 1, GSTU1); AaGSTI1 = AAEL011752. In the protein identifiers: AGAP = *Anopheles gambiae* PEST strain; AAEL = *Aedes aegypti* Liverpool strain; PPAI = *Phlebotomus papatasi* Israel strain; LLOJ = *Lutzomyia longipalpis* Jacobina strain; ‘‐PA’, ‘‐PB’ and so forth indicate protein isoforms of the same gene; ‘_a’, ‘_b’ and so forth (for the sand fly proteins only) indicate separate genes created by manual editing of a gene model

The *GstI* gene models differed between sand flies and mosquitoes (Figure 6). Both mosquito and sand fly GSTI genes had two exons and one intron, but the intron occurred in a different place in the mosquito and sand fly genes.

**FIGURE 7:**
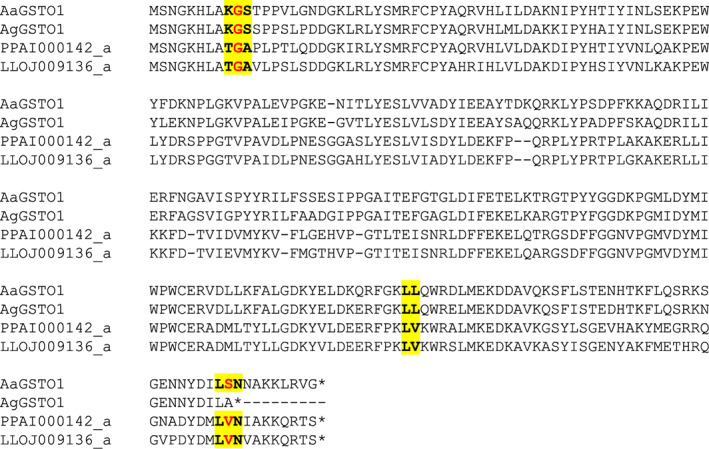
Intron–exon structure of GSTO proteins. An alignment of GSTO proteins is shown, with exon–exon junctions shown in bold and highlighted in yellow (including the amino acid immediately upstream and downstream of the junction and those spanning the intron shown in red). AgGSTO1 = AGAP005749; AaGSTO1 = AAEL017085. In the protein identifiers: AGAP = *Anopheles gambiae* PEST strain; AAEL = *Aedes aegypti* Liverpool strain; PPAI = *Phlebotomus papatasi* Israel strain; LLOJ = *Lutzomyia longipalpis* Jacobina strain; ‘‐PA’, ‘‐PB’ and so forth indicate protein isoforms of the same gene; ‘_a’, ‘_b’ and so forth (for the sand fly proteins only) indicate separate genes created by manual editing of a gene model

Two genes (PPAI000142, LLOJ009136) were identified as putative GSTO. Both gene models were edited (Appendix S1), to alter the boundaries of exons (LLOJ009136_a) and, for PPAI000142_a, the first half of the gene was found, unannotated, on a different scaffold (AJVK01076482.1) by a tBLASTn search. After editing, each sand fly genome contained 1 GSTO gene like *An. gambiae* and *Aedes aegypti* but unlike *D. melanogaster*, which has 4 (Table 1, Tables S1 and S2). The *GstO* gene models were largely the same between sand flies and mosquitoes with four exons and three introns (*An. gambiae* lacked a fourth exon) occurring at the same locations in the genes (Figure 7).

Three genes (PPAI002540, LLOJ009036, LLOJ009037) were identified as putative GSTS. PPAI002540 appeared to be a partial gene, lacking the first exon. The full‐length scaffold JH662805.1 up to the gene PPAI002540 was searched for evidence of the first exon of the gene but a BLASTx search of the six‐frame translation of the scaffold against *Aedes aegypti* and *Anopheles gambiae* predicted proteomes identified nothing. The scaffold was highly fragmented, with a lot of sequence gaps, which may account for this. A tBLASTn search of all *P. papatasi* contigs, using the N‐terminal region of the *Anopheles gambiae* gene identified exon 1, unannotated on another scaffold (AJVK01074729.1), allowing a putative full‐length protein (PPAI002540_a) to be reconstructed. LLOJ009036 and LLOJ009037 were adjacent to one another on the same scaffold, in a region containing sequencing gaps. LLOJ009036 was edited and appeared to consist only of the first exon of the gene (the partial gene denoted LLOJ009036_a). LLOJ009037 was left unchanged, and contained three exons, producing a full‐length protein. It is possible that both are fragments of a single gene that is not fully collapsed in the assembly (the amino acid sequence of LLOJ009036_a was identical to exon 1 of LLOJ009037). After editing (Appendix S1), it could be determined that each genome contained at least one GSTS gene.

**FIGURE 8:**
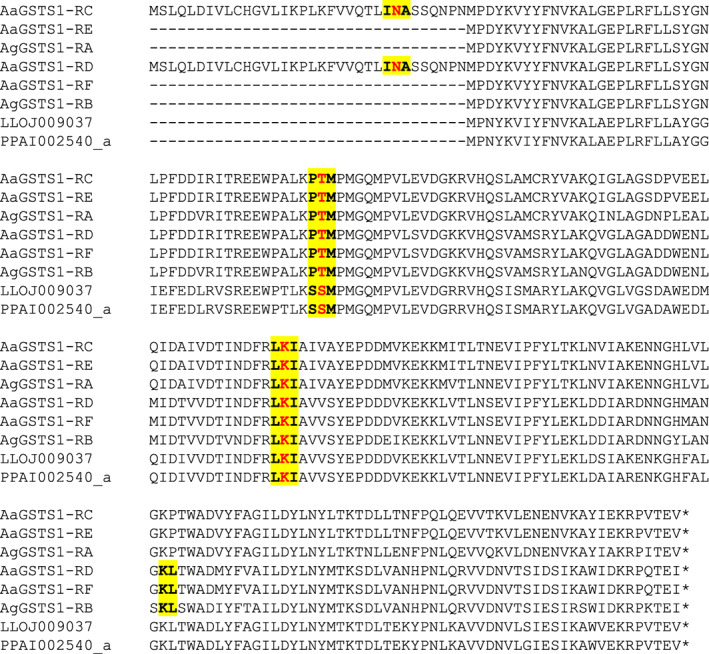
Intron–exon structure of GSTS proteins. An alignment of GSTS proteins (including isoforms) is shown, with exon–exon junctions shown in bold and highlighted in yellow (including the amino acid immediately upstream and downstream of the junction and those spanning the intron shown in red). A partial gene (LLOJ009036_a) identical to exon 1 of LLOJ009037 is not shown. AgGSTS1 = AGAP010404; AaGSTS1 = AAEL011741. In the protein identifiers: AGAP = *Anopheles gambiae* PEST strain; AAEL = *Aedes aegypti* Liverpool strain; PPAI = *Phlebotomus papatasi* Israel strain; LLOJ = *Lutzomyia longipalpis* Jacobina strain; ‘‐PA’, ‘‐PB’ and so forth indicate protein isoforms of the same gene; ‘_a’, ‘_b’ and so forth (for the sand fly proteins only) indicate separate genes created by manual editing of a gene model

Sand fly *GstS* genes shared most structural similarity with *Ae. aegypti GstS* isoform E and *An. gambiae* isoform A, lacking the additional upstream exon of *Ae. aegypti GstS* isoforms C and D and the final intron of *Ae. aegypti GstS* isoforms D and F and *An. gambiae* isoform B. However, they shared sequence similarity with both *Aa*CE‐*Ag*A‐type and *Aa*DF‐*Ag*B‐type isoforms in their C‐terminal regions, which in the mosquitoes are encoded by homologous but non‐overlapping exons. This pattern suggests that the mosquito isoforms may have arisen by duplication of these exons and that they remain un‐duplicated in the sand flies (Figure 8, Figure S2).

**FIGURE 9:**
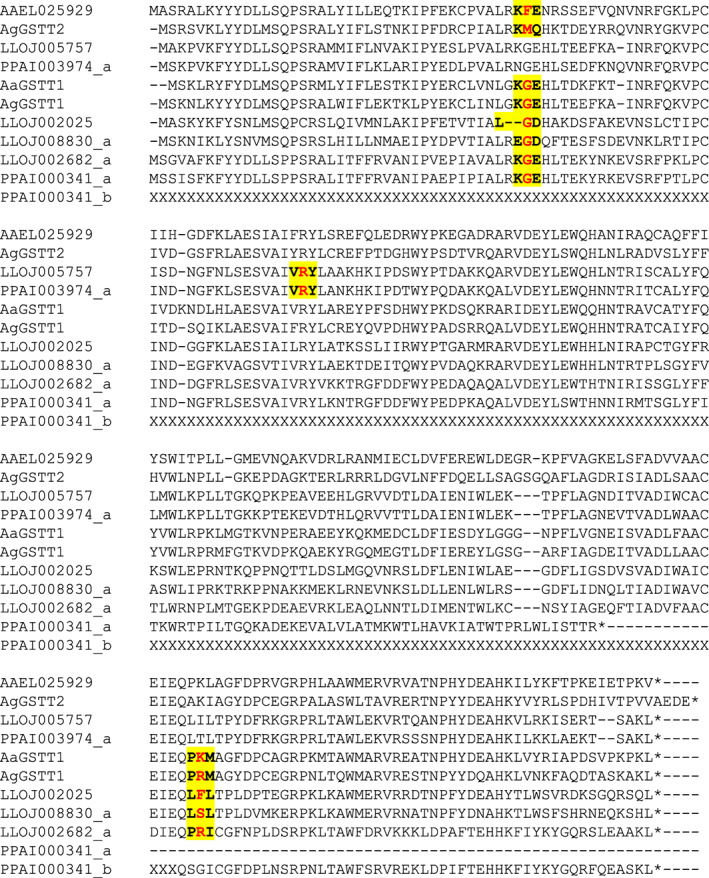
Intron–exon structure of GSTT proteins. An alignment of GSTT proteins is shown, with exon–exon junctions shown in bold and highlighted in yellow (including the amino acid immediately upstream and downstream of the junction and those spanning the intron shown in red). Unknown amino acids are represented by ‘X’. AgGSTT1 = AGAP000761; AgGSTT2 = AGAP000888; AaGSTT1 = AAEL009017. In the protein identifiers: AGAP = *Anopheles gambiae* PEST strain; AAEL = *Aedes aegypti* Liverpool strain; PPAI = *Phlebotomus papatasi* Israel strain; LLOJ = *Lutzomyia longipalpis* Jacobina strain; ‘‐PA’, ‘‐PB’ and so forth indicate protein isoforms of the same gene; ‘_a’, ‘_b’ and so forth (for the sand fly proteins only) indicate separate genes created by manual editing of a gene model

Six genes (PPAI003974, PPAI000341, LLOJ005757, LLOJ002025, LLOJ008830, LLOJ002682) were identified as putative GSTT. In *P. papatasi*, PPAI003974 was edited to reconstruct a full‐length, two‐exon gene (PPAI003974_a). The intron occurred downstream in the gene compared with other GSTT genes (Figure 9), but this gene model was also seen in the two‐exon *L. longipalpis* gene LLOJ005757 (which was left unchanged) and these genes were reciprocal best BLASTp matches. The three‐exon gene LLOJ002025 was left unchanged and two unusually long genes, LLOJ008830 (786 aa, nine exons) and LLOJ002682 (446 aa; six exons), were edited to reconstruct 3‐exon GSTT genes LLOJ008830_a (229 aa; three exons) and LLOJ002682_a (230 aa; three exons). These three genes shared their intron locations with a gene in *Ae. aegypti* (AAEL009017) and *An. gambiae* (AGAP000761). The *P. papatasi* gene PPAI000341 was edited (to PPAI000341_a) to alter the length of its first exon, producing a two‐exon gene. However, PPAI000341_a was shorter than other GSTT genes and its 3′ end did not align well, suggesting misannotation and a possible missing third exon (a nearby downstream gap may have prevented this being found). Downstream of this gap, a partial sequence matching the end of a GSTT was found (PPAI000341_b) and PPAI000341_a and PPAI000341_b may be a single gene, but they could not be reconstructed into a good single gene model. Overall, four GSTT genes were identified in the *L. longipalpis* genome and 2–3 in the *P. papatsi* genome (Table 1, Tables S1 and S2).

**FIGURE 10:**
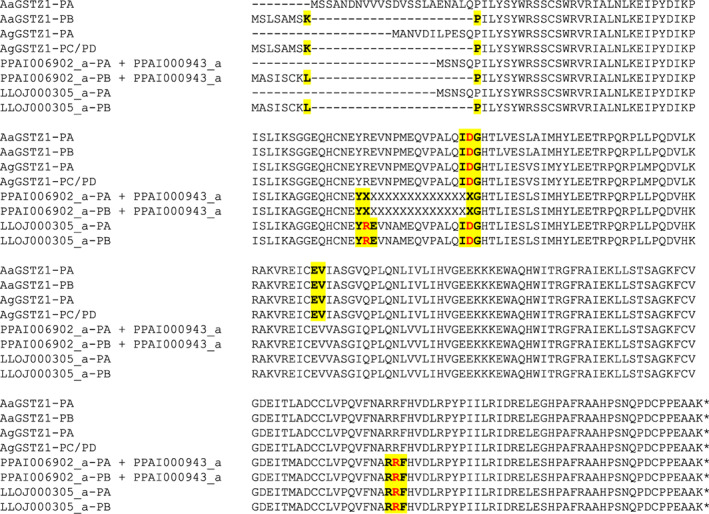
Intron–exon structure of GSTZ proteins. An alignment of GSTZ proteins is shown, with exon–exon junctions shown in bold and highlighted in yellow (including the amino acid immediately upstream and downstream of the junction and those spanning the intron shown in red). Two *P. papatasi* genes (PPAI006902_a and PPAI000943_a) were merged to form one putative gene (missing a middle exon). In each case, both protein isoforms (differing at the first exon/intron) are shown. Unknown amino acids (due to genome assembly gaps) are represented by ‘X’. AgGSTZ1 = AGAP002898; AaGSTZ1 = AAEL011934. In the protein identifiers: AGAP = *Anopheles gambiae* PEST strain; AAEL = *Aedes aegypti* Liverpool strain; PPAI = *Phlebotomus papatasi* Israel strain; LLOJ = *Lutzomyia longipalpis* Jacobina strain; ‘‐PA’, ‘‐PB’ and so forth indicate protein isoforms of the same gene; ‘_a’, ‘_b’ and so forth (for the sand fly proteins only) indicate separate genes created by manual editing of a gene model

Three genes (PPAI006902, PPAI000943, LLOJ000305) were identified as putative GSTZ. Closer inspection (Appendix S1) suggested that PPAI006902 and PPAI000943 might be the start and end of the same gene (though missing an internal exon) on different scaffolds. LLOJ000305 was a complete gene. At the beginning of each gene, evidence of alternative splicing patterns could be seen allowing two protein isoforms to be reconstructed (as seen in *Aedes* and *Anopheles* orthologues). This suggested that a single GSTZ gene, encoding at least two protein isoforms, was present in each genome. In both sand fly genomes, the number and position of the introns differed from those seen in both mosquitoes (Figure 10).

**FIGURE 11:**
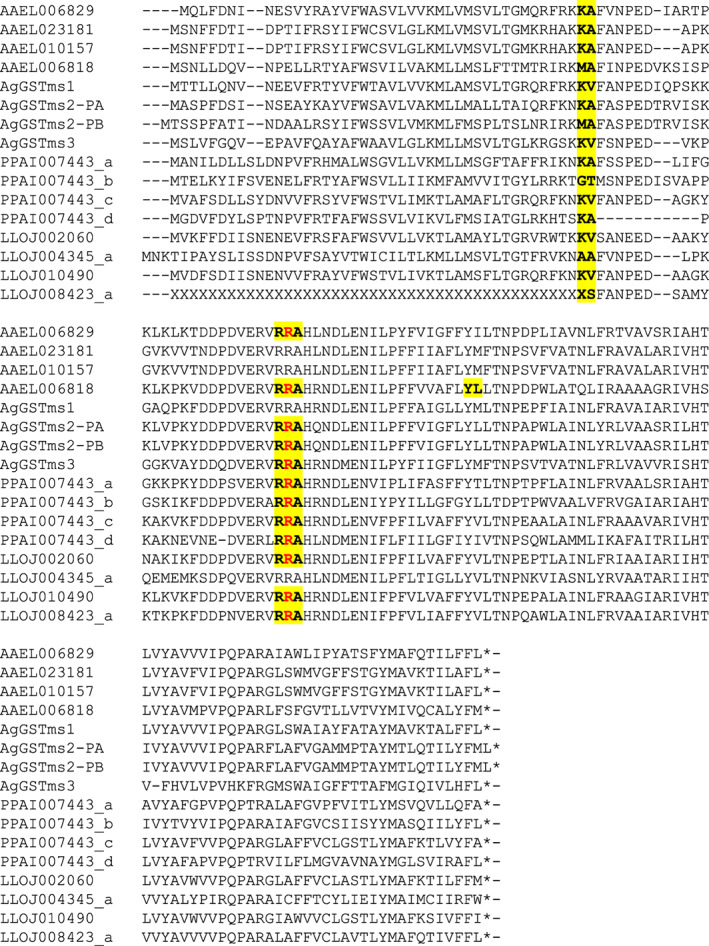
Intron–exon structure of microsomal GST proteins. An alignment of microsomal GST proteins is shown, with exon–exon junctions shown in bold and highlighted in yellow (including the amino acid immediately upstream and downstream of the junction and those spanning the intron shown in red). Unknown amino acids (due to genome assembly gaps) are represented by ‘X’. AgGSTms1 = AGAP000165; AgGSTms2 = AGAP000163; AgGSTms3 = AGAP009946. In the protein identifiers: AGAP = *Anopheles gambiae* PEST strain; AAEL = *Aedes aegypti* Liverpool strain; PPAI = *Phlebotomus papatasi* Israel strain; LLOJ = *Lutzomyia longipalpis* Jacobina strain; ‘‐PA’, ‘‐PB’ and so forth indicate protein isoforms of the same gene; ‘_a’, ‘_b’ and so forth (for the sand fly proteins only) indicate separate genes created by manual editing of a gene model

Five genes (PPAI007443, LLOJ002060, LLOJ010490, LLOJ008423, LLOJ004345) were identified as putative microsomal GSTs. PPAI007443 was split into four separate genes (PPAI007443_a–PPAI007443_d). LLOJ002060 and LLOJ010490 were not changed. LLOJ004345 and LLOJ008423 were both edited but LLOJ008423_a remained incomplete, lacking a first exon possibly due to a sequencing gap upstream of the annotated exons. The four *P. papatsi* genes were all adjacent to one another on scaffold JH665380.1, as were LLOJ004345_a and LLOJ010490 (on scaffold JH689577). The other two *L. longipalpis* genes were on different scaffolds (LLOJ002060 on JH689469; LLOJ008423_a on JH690025). Overall, four microsomal GST genes were identified in the *L. longipalpis* genome and four in the *P. papatsi* genome (Table 1, Tables S1 and S2, Appendix S1). The gene models were largely the same between sand flies and mosquitoes (Figure 11).

## DISCUSSION

We characterized the glutathione S‐transferase complement in the genomes of *Phlebotomus papatasi* and *Lutzomyia longipalpis*. From the study we drew two main conclusions: (i) accurate gene annotation in these species is difficult due to the fragmentary nature of the genome assemblies; (ii) the insect‐specific GST complement of these sand flies differs markedly from those of mosquitoes and of *Drosophila melanogaster*, with no GSTE class and an expansion of the GSTX class.

Overall, 23–25 GST genes were identified in *P. papatasi* and 44–47 in *L. longipalpis*, both within the typical range for insects but quite different from one another. It is difficult to conclude definitively if this difference in numbers is due to a real biological difference, or is an artefact of the differing quality of the two genome assemblies. The *L. longipalpis* assembly (11,532 scaffolds; N50 = 85,093) is much more contiguous than that of *P. papatasi* (106,826 scaffolds; N50 = 27,956), which may affect the number of GST genes identified in each genome. Genes are more likely to go unannotated in, or to be missing from more fragmented assemblies, and during manual editing of the GST gene annotations, we saw a number of cases of genes running into unsequenced assembly gaps in scaffolds and some cases of genes split across two scaffolds. These factors all affect the quality of genome annotation, so our estimates of gene numbers are necessarily cautious ones. With this in mind, we did try to find additional unannotated GST genes (using tBLASTn searching of the genome assemblies), which did identify some partial genes, though not large numbers. We did not, for example, find any unannotated GSTD genes in *P. papatasi* in addition to the two we annotated, though it remains a possibility that they exist on parts of the genome not represented in the assembly. Nor did we find any evidence for GSTE genes in either genome, lending weight to the conclusion that this class has been lost from the sand fly lineage.

Extensive manual editing and curation of the annotation of key gene families is often required in draft genome assemblies and is an important prerequisite for further analysis such RNAseq‐based gene expression profiling (Weedall et al., 2015). Here, the value of manual curation of the gene models and the draft status of the existing annotation are illustrated by the amount of editing that was required. In *P. papatasi*, only five gene models were left unedited while 20 resulted from manual editing. In *L. longipalpis*, 16 gene models were left unedited and 31 resulted from manual editing. This editing ranged from minor alteration of intron–exon boundaries to splitting of large ‘genes’ into multiple members of gene clusters. Additional genome sequencing to improve the genome assemblies, alongside RNAseq (Petrella et al., 2015) and sequencing of more sand fly genomes would markedly improve the genome annotation of these species.

Compared with *Anopheles gambiae*, *Aedes aegypti* and *Drosophila melanogaster*, the microsomal GSTs and the cytosolic GSTs found across the animal kingdom (GSTO, S, T, Z) showed similar numbers in the sand flies. The most striking contrasts were seen in the ‘insect‐specific’ expanded classes: the absence of the GSTE class from both sand fly genomes; the apparent reduction in the size of the GSTD class in *P. papatasi* (to two genes) but not *L. longipalpis* (11 genes); and the expansion of the GSTX class seen in both sand fly genomes (from two copies seen in the mosquitoes and none in *D. melanogaster* to 11–12 in *P. papatasi* and 23 in *L. longipalpis*). Given the importance of these insect‐specific GST classes in insecticide resistance, these differences may be important for understanding resistance in sand flies.

GSTEs play key roles in resistance to organochlorine, pyrethroid and organophosphate class insecticides in disease vector species including mosquitoes of the genera *Anopheles* and *Aedes* (Ranson et al., 2001; Lumjuan et al., 2005, 2011; Mitchell et al., 2014; Wilding et al., 2015; Kouamo et al., 2021; Riveron et al., 2014) and in agricultural pest species including the oriental fruit fly *Bactrocera dorsalis* (Hu et al., 2014; Lu et al., 2016), the Colorado potato beetle *Leptinotarsa decemlineata* (Han et al., 2016) and the tobacco cutworm moth *Spodoptera litura* (Zhang et al., 2016). GSTDs (two genes in *P. papatsi* and 11 in *L. longipalpis*) are also associated with resistance in many species, including *B. dorsalis* (Hu et al., 2014; Lu et al., 2016), *L. decemlineata* (Han et al., 2016), the diamondback moth *Plutella xylostella* (You et al., 2015), the red spider mite *Tetranychus urticae* (Pavlidi et al., 2015), the fungus gnat *Bradysia odoriphaga* (Tang et al., 2019; Tchouakui et al., 2019) and the mosquitoes *Culex pipiens* (Xu et al., 2017) and *An. gambiae* (Pavlidi et al., 2015; Isaacs et al., 2018). Given the importance of these GST classes, the apparent absence of GSTE from the genomes of the two sand fly species may affect their ability to evolve resistance to these insecticide classes, as might the apparent reduction in the size of the GSTD class in *P. papatasi*.

The fact that the GSTX class is expanded in both sand fly species suggests an adaptive role in sand fly evolution, yet their precise roles are not known, nor whether they may be involved in the detoxification of insecticides. It is interesting to speculate on whether GSTX may perform in the sand flies the roles performed by the related GSTE and GSTD classes in mosquitoes and other insects. The roles of the two GSTX genes (GSTX1 and GSTX2) of *Aedes aegypti* and *Anopheles gambiae* in metabolic insecticide resistance are unclear (Grant and Hammock, 1992; Lumjuan et al., 2007). While overexpression of GSTX2 (called GST‐2 in that study) in *Ae. aegypti* from South America was associated with DDT resistance (Grant and Hammock, 1992), a subsequent study found no catalytic activity of GSTX2 on DDT (Lumjuan et al., 2007). That study did, however, identify hematin binding activity in GSTX2, not previously seen in other GSTs, and the authors suggested a possible protective role of GSTX in blood‐feeding insects, such as the reduction of heme toxicity after a blood meal. A recent study on the sand fly vector of visceral Leishmaniasis, *Phlebotomus argentipes*, from Bihar, India reported the amplification, cloning and enzymatic characterization of a GST reported to be a GSTD (Hassan et al., 2021). *P. argentipes* populations have evolved DDT resistance in this region (Dinesh et al., 2021) and target site resistance mutations have been reported (Gomes et al., 2017) but no metabolic resistance mechanisms. Importantly in this regard, Hassan et al. (Hassan et al., 2021) showed that this protein could metabolize DDT *in vitro*, making it a key metabolic resistance candidate. Based on the GST family characterization work we report here, we found the best matches to this protein in *P. papatasi* and *L. longipalpis* are the class we have defined here as GSTX. Therefore, this would indicate that a *P. argentipes* GSTX can metabolize an insecticide, making proteins of this novel GST class key candidates for further study of metabolic resistance in sand flies. Further work is needed to functionally characterize the sand fly GSTX genes. This would include the full range of complementary approaches applied to the GST genes of other species, including *in vitro* characterization of enzyme activity (Lumjuan et al., 2005; Riveron et al., 2014; Mitchell et al., 2014), *in vivo* heterologous protein expression in a model organism (Riveron et al., 2014; Mitchell et al., 2014), gene silencing *in vivo* (Kouamo et al., 2021; Lumjuan et al., 2011), protein structure analysis and 3D modelling (Wang et al., 2008; Riveron et al., 2014; Mitchell et al., 2014) and gene expression and population genetic analyses (Riveron et al., 2014; Weedall et al., 2019; Kouamo et al., 2021; Ranson et al., 2001; Lumjuan et al., 2005; Weedall et al., 2020; Anopheles gambiae 1000 Genomes Consortium, 2017). Together, these would allow us to further elucidate the biological roles of the novel GSTX class and its potential to confer insecticide resistance in sand flies.

The work presented here, alongside ongoing work to define the cytochrome P450 monooxygenase and carboxylesterase families in these genomes (in preparation), will provide a basis for further study of metabolic resistance mechanisms in these important vectors of Leishmaniasis.

### Experimental procedures

Two well‐annotated mosquito species, *Aedes aegypti* (Liverpool strain) (Matthews et al., 2018) and *Anopheles gambiae* (PEST strain) (Holt et al., 2002), were used to generate initial GST protein lists to query the predicted proteomes of two sand fly species, *Phlebotomus papatasi* (Israel strain) and *Lutzomyia longipalpis* (Jacobina strain), in VectorBase (Megy et al., 2012) (https://www.vectorbase.org/). This was done using BLASTp with default parameters and search results defined initial gene family candidate lists for each sandfly species. These lists were filtered by reciprocal BLASTp searching against *Ae. aegypti*, *An. gambiae* and *D. melanogaster* predicted proteomes, retaining genes with best matches to GSTs and removing genes with best matches to other gene families.

Protein lengths and exons numbers were recorded for candidate GST genes in sand flies and for their best matches in the mosquito species and *D. melanogaster*. Where sand fly and mosquito homologues showed strikingly different gene length or exon number, misannotation of the sandfly gene was suspected and gene models were manually edited. This process was guided by sequence alignments of the sand fly, mosquito and fruit fly genes and proteins made using the MUSCLE algorithm (Edgar, 2004) implemented in Seaview version 4.5.4 (Gouy, Guindon and Gascuel, 2010; Petrella et al., 2015). These alignments helped identify missing or truncated exons in the sand fly genomes, which were edited and new gene models checked using the ExPASy translate tool (Artimo et al., 2012) (https://web.expasy.org/translate/) to ensure they encoded full‐length open‐reading frames. BLASTp was used to determine the best matches of these edited sandfly genes in other insect species and this information was used to assign each GST to a class. In addition to protein versus protein BLASTp searches, sand fly DNA versus non‐sand fly protein BLASTx searching was used to identify unannotated sand fly exons, and non‐sand fly GST protein versus sand fly genomic DNA tBLASTn searches to locate unannotated genes. All BLAST searches were carried out in vEuPathDB/Vectorbase (Megy et al., 2012) (https://www.vectorbase.org/).

Phylogenetic analysis of sequence alignments was implemented in Seaview (Gouy, Guindon and Gascuel, 2010; Petrella et al., 2015), using the Neighbour Joining method with Poisson distances and 100 bootstrap replicates. Trees were visualized using iTol (Letunic and Bork, 2021).

## CONFLICT OF INTEREST

There are no conflicts of interest to declare among the authors of the manuscript.

## Supporting information


**Figure S1.** Editing of mis‐annotated genes in the genomes of *P. papatasi* and *L. longipalpis* to form GST gene clusters. (A) A cluster of GSTD genes in *L. longipalpis*. (B and C) Two clusters of GSTX genes in *L. longipalpis*. (D) A cluster of GSTX genes in *P. papatasi*. In each case, the position of the gene(s) in an assembled scaffold (line) is indicated by a pale grey box; dark grey boxes indicate non‐GST flanking genes. Below this, a zoomed‐in view of the gene(s) is shown. Here, the lines connect the exons (pale grey boxes) of each gene and the arrow head indicates the DNA strand the gene(s) is on. Below this, the edited gene models are shown. Lines link exons (boxes) on the same gene. The colours (green or orange) distinguish adjacent genes. Lower‐case letters (a, b, c and so forth) are used to distinguish different genes formed by editing a single larger gene model. The pale blue box in panel D indicates a gene, PPAI010871_e, for which the gene model could not be fully reconstructed though the start and end of the gene were present (hence the intron–exon structure is not shown).

**Figure S2.** Editing and reconstruction of *GstS* genes in the genomes of *P. papatasi* and *L. longipalpis*. (A) Reconstruction of a *GstS* gene split across two scaffolds in *P. papatasi*. (B) Reconstruction of 1–2 *GstS* genes in *L. longipalpis*. (C) Homology between *GstS* exons in *Ae. aegypti* (*Aa*), *An. gambiae* (*Ag*) and the two sand fly species. Exons (green/orange boxes) are linked by introns (lines). The isoforms annotated in the two mosquito species are shown, with grey boxes indicating shared (within‐species) and orthologous (between‐species) exons. The dotted lines indicate shared intron locations. The red arrow shows a model of how the isoforms may have arisen in the mosquitoes, with duplication of the two final exons followed by the generation of a novel intron (red dotted lines). If this process occurred only in the mosquito lineage and not in the sand fly lineage it may explain the pattern of sequence homology seen between the final exons.

**Table S1.** Glutathione S‐Transferase genes in *Phlebotomus papatasi*. The gene ID, protein length (amino acids; aa) and number of exons (protein coding exons only; exons in UTR excluded) are shown for *P. papatasi* genes and their best matching genes in *Aedes aegypti*, *Anopheles gambiae* and *Drosophila melanogaster*. Gene IDs followed by a letter (e.g., ‘_a’) were manually edited from the original gene model.

**Table S2.** Glutathione S‐Transferase genes in *Lutzomyia longipalpis*. The gene ID, protein length (amino acids) and number of exons are shown for *L. longipalpis* genes and their best matching genes in *Aedes aegypti*, *Anopheles gambiae* and *Drosophila melanogaster*. Gene IDs followed by a letter (e.g., ‘_a’) were manually edited from the original gene model.

**Appendix S1.** Supporting information.

## Data Availability

The data that support the findings of this study are available in VEuPathDB at https://veupathdb.org/veupathdb/app.
