# Supplementary material for: Characterization of the glutathione S‐transferase genes in the sand flies Phlebotomus papatasi and Lutzomyia longipalpis shows expansion of the novel glutathione S‐transferase xi (X) class
Source: Insect Mol Biol. 2022 Mar 8;31(4):417–33. doi: 10.1111/imb.12769 (PMC9540044; doi:10.1111/imb.12769)
Supplement: Supplementary file 1 — Figure S1. Editing of mis‐annotated genes in the genomes of P. papatasi and L. longipalpis to form GST gene clusters. (A) A cluster of GSTD genes in L. longipalpis. (B and C) Two clusters of GSTX genes in L. longipalpis. (D) A cluster of GSTX genes in P. papatasi. In each case, the position of the gene(s) in an assembled scaffold (line) is indicated by a pale grey box; dark grey boxes indicate non‐GST flanking genes. Below this, a zoomed‐in view of the gene(s) is shown. Here, the lines connect the exons (pale grey boxes) of each gene and the arrow head indicates the DNA strand the gene(s) is on. Below this, the edited gene models are shown. Lines link exons (boxes) on the same gene. The colours (green or orange) distinguish adjacent genes. Lower‐case letters (a, b, c and so forth) are used to distinguish different genes formed by editing a single larger gene model. The pale blue box in panel D indicates a gene, PPAI010871_e, for which the gene model could not be fully reconstructed though the start and end of the gene were present (hence the intron–exon structure is not shown). [file IMB-31-417-s001.pdf]

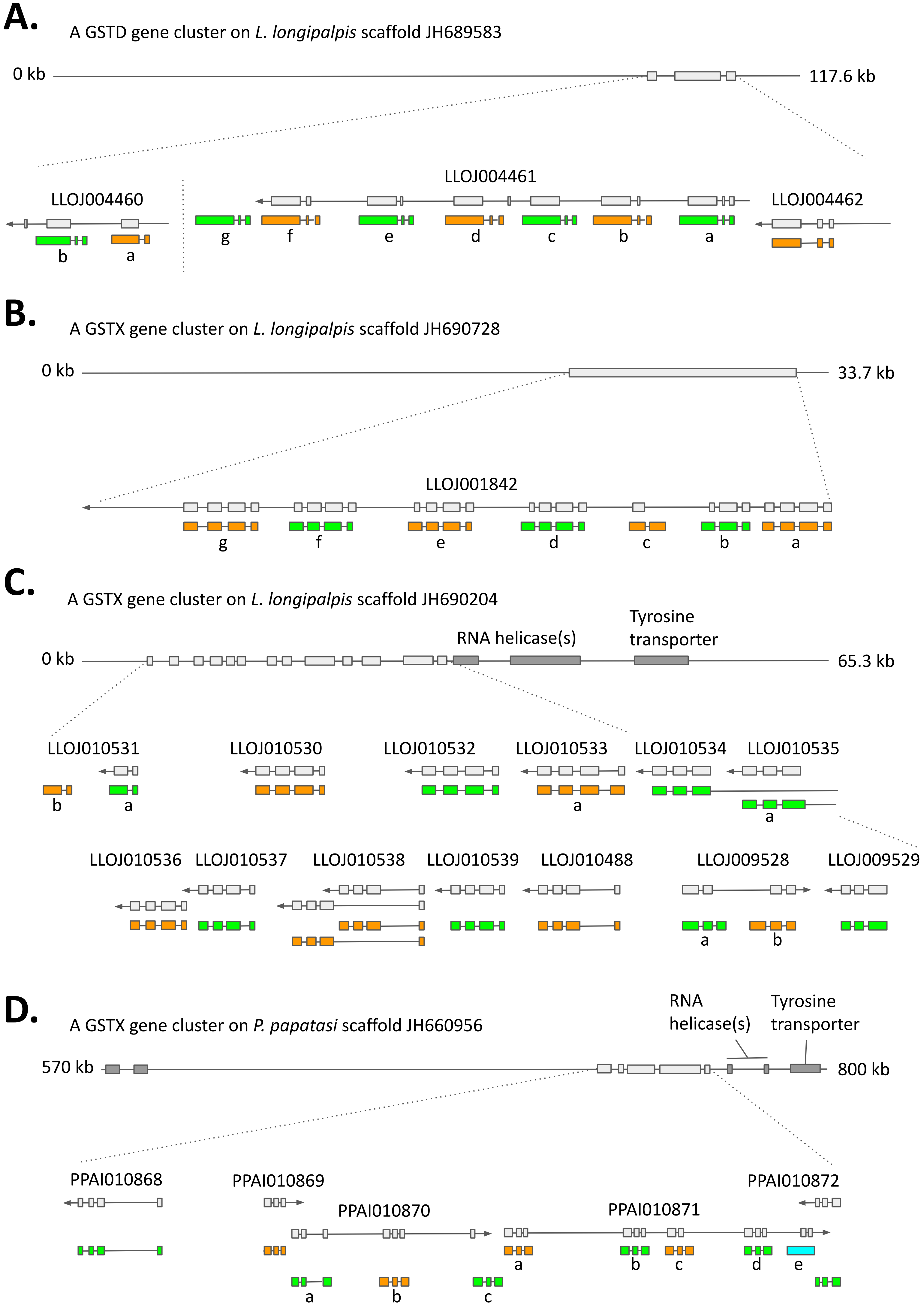
A.
A GSTD gene cluster on L. longipalpis scaffold JH689583
0 kb
117.6 kb
LLOJ004460
b
a
LLOJ004461
g
f
e
d
c
b
a
LLOJ004462
B.
A GSTX gene cluster on L. longipalpis scaffold JH690728
0 kb
33.7 kb
LLOJ001842
g
f
e
d
c
b
a
C.
A GSTX gene cluster on L. longipalpis scaffold JH690204
RNA helicase(s)
Tyrosine transporter
0 kb
65.3 kb
LLOJ010531
b
a
LLOJ010530
LLOJ010532
LLOJ010533
a
LLOJ010534
LLOJ010535
a
LLOJ010536
LLOJ010537
LLOJ010538
LLOJ010539
LLOJ010488
LLOJ009528
a
b
LLOJ009529
D.
A GSTX gene cluster on P. papatasi scaffold JH660956
RNA helicase(s)
Tyrosine transporter
570 kb
800 kb
PPAI010868
PPAI010869
PPAI010870
a
b
c
PPAI010871
a
b
c
d
e
PPAI010872
