# Supplementary figures and images for: Characterization of the glutathione S‐transferase genes in the sand flies Phlebotomus papatasi and Lutzomyia longipalpis shows expansion of the novel glutathione S‐transferase xi (X) class
Source: Insect Mol Biol. 2022 Mar 8;31(4):417–33. doi: 10.1111/imb.12769 (PMC9540044; doi:10.1111/imb.12769)

**A.***P. papatasi* GSTS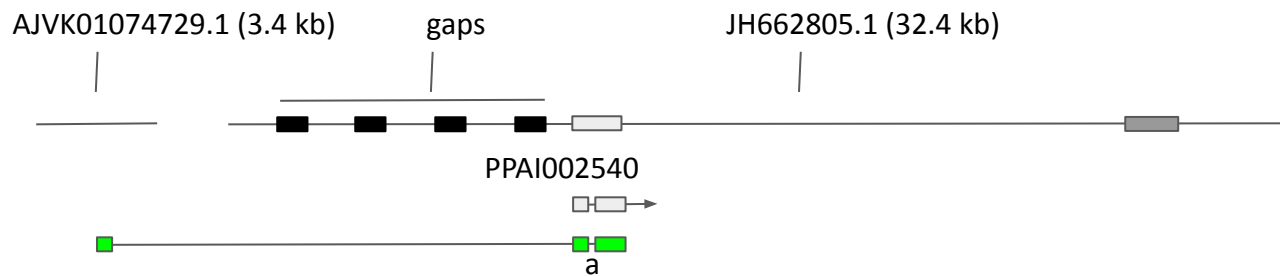**B.***L. longipalpis* GSTS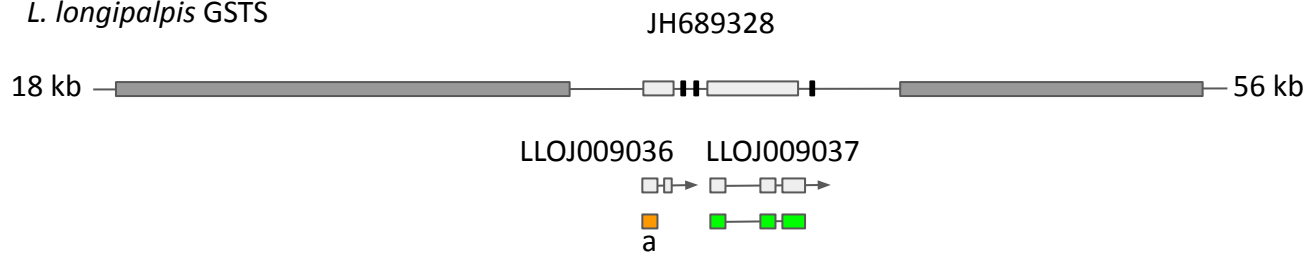**C.**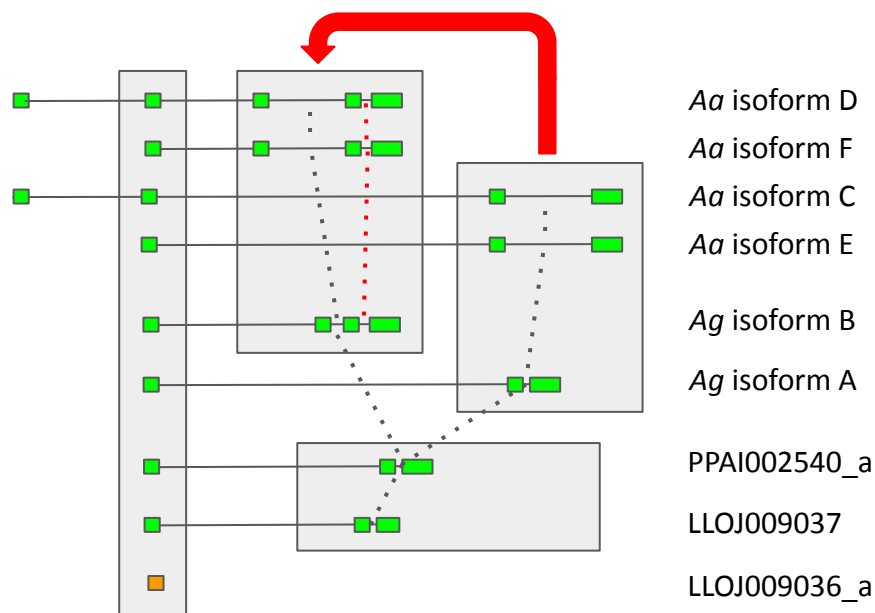

Supplement: Supplementary file 2 — Figure S2. Editing and reconstruction of GstS genes in the genomes of P. papatasi and L. longipalpis. (A) Reconstruction of a GstS gene split across two scaffolds in P. papatasi. (B) Reconstruction of 1–2 GstS genes in L. longipalpis. (C) Homology between GstS exons in Ae. aegypti (Aa), An. gambiae (Ag) and the two sand fly species. Exons (green/orange boxes) are linked by introns (lines). The isoforms annotated in the two mosquito species are shown, with grey boxes indicating shared (within‐species) and orthologous (between‐species) exons. The dotted lines indicate shared intron locations. The red arrow shows a model of how the isoforms may have arisen in the mosquitoes, with duplication of the two final exons followed by the generation of a novel intron (red dotted lines). If this process occurred only in the mosquito lineage and not in the sand fly lineage it may explain the pattern of sequence homology seen between the final exons. [file IMB-31-417-s004.pdf]
