# Supplementary material for: Characterization of the glutathione S‐transferase genes in the sand flies Phlebotomus papatasi and Lutzomyia longipalpis shows expansion of the novel glutathione S‐transferase xi (X) class
Source: Insect Mol Biol. 2022 Mar 8;31(4):417–33. doi: 10.1111/imb.12769 (PMC9540044; doi:10.1111/imb.12769)
Supplement: Supplementary file 3 — Table S1. Glutathione S‐Transferase genes in Phlebotomus papatasi. The gene ID, protein length (amino acids; aa) and number of exons (protein coding exons only; exons in UTR excluded) are shown for P. papatasi genes and their best matching genes in Aedes aegypti, Anopheles gambiae and Drosophila melanogaster. Gene IDs followed by a letter (e.g., ‘_a’) were manually edited from the original gene model. [file IMB-31-417-s003.pdf]

Supplementary Table 1. Glutathione S-Transferase genes in *Phlebotomus papatasi*. The gene ID, protein length (amino acids; aa) and number of exons (protein coding exons only; exons in UTR excluded) are shown for *P. papatasi* genes and their best matching genes in *Aedes aegypti*, *Anopheles gambiae* and *Drosophila melanogaster*. Gene IDs followed by a letter (e.g. “_a”) were manually edited from the original gene model.

Phlebotomus papatasi	Subfamily	*Aedes aegypti*	*Anopheles gambiae*	*Lutzomyia longipalpis*	*Drosophila melanogaster*
PPAI001211 (217 aa; 3 exons)	GSTD	AAEL001090, GSTD7 (218 aa; 4 exons)	AGAP004163, GSTD7 (218 aa; 3 exons)	LLOJ007285 (220 aa; 3 exons)	FBgn0038029, GSTD11 (222/243 aa; 3/3 exons)
PPAI006595_a [1] (224 aa; 3 exons)	GSTD	AAEL001061, GSTD1 (216/209/211 aa; 2/2/2 exons)	AGAP004164, GSTD1-4 (209/209/216/186 aa; 2/2/2/2 exons)	LLOJ004462, GSTD (212 aa; 3 exons)	FBgn0001149, GSTD1 (209/209 aa; 1/1 exon)
PPAI009870 (229 aa; 2 exons)	GSTI	AAEL011752, GSTI1 (231 aa; 2 exons)	AGAP000947, GSTU1 (233 aa; 2 exons)	LLOJ002711 (339 aa; 4 exons)	FBgn0250732, GST-containing FLYWCH zinc-finger protein, gfzf (234/1045/1045 aa; 2/4/4 exons)
PPAI000142_a [2] (253 aa; 4 exons; gene split across 2 scaffolds)	GSTO	AAEL017085, GSTO1 (257 aa; 4 exons)	AGAP005749, GSTO1 (248 aa; 3 exons)	LLOJ009136_a, GSTO2 (253 aa; 4 exons)	FBgn0035907, GstO1 (254 aa; 4 exons)
PPAI002540_a [3], GSTS1 (203 aa; 3 exons)	GSTS	AAEL011741, GSTS1 (237/237/203/203 aa; 4/5/3/4 exons)	AGAP010404, GSTS1 (203/203 aa; 3/4 exons)	LLOJ009037, GSTS1 (203 aa; 3 exons)	FBgn0010226, GstS1 (249/249/249/250 aa; 4/4/4/4 exons)
PPAI003974_a [4] (227 aa; 2 exons)	GSTT	AAEL009017, GSTT1 (229 aa; 3 exons)	AGAP000761, GSTT1 (229 aa; 3 exons)	LLOJ005757 (226 aa; 2 exons)	FBgn0031117, GstT3 (228/228/268 aa; 3/3/4 exons)
PPAI000341_a [5] (167 aa; 2 exons)	GSTT	AAEL025929 (231 aa; 2 exons)	AGAP000761, GSTT1 (229 aa; 3 exons)	LLOJ002682 (446 aa; 5 exons)	FBgn0031117, GstT3 (228/228/268 aa; 3/3/4 exons)
PPAI000341_b [5] (>51 aa; >1 exon)	GSTT	AAEL009017, GSTT1 (229 aa; 3 exons)	AGAP000761, GSTT1 (229 aa; 3 exons)	LLOJ002025 (227 aa; 3 exons)	FBgn0050000, GstT1 (228 aa; 4 exons)
PPAI010868 (221 aa; 4 exons)	GSTX	AAEL010500, GSTX2 (218/192 aa; 3/3 exons)	AGAP009342, GSTU3 (218 aa; 2 exons)	LLOJ010538 (222/221 aa; 4/4 exons)	FBgn0063497, GstE3 (220 aa; 1 exon)
PPAI010869 (221 aa; 3 exons)	GSTX	AAEL010500, GSTX2 (218/192 aa; 3/3 exons)	AGAP009342, GSTU3 (218 aa; 2 exons)	LLOJ009528 (277 aa; 4 exons)	FBgn0063494, GstE6 (222 aa; 1 exon)
PPAI010870_a [6] (219 aa; 3 exons)	GSTX	AAEL010500, GSTX2 (218/192 aa; 3/3 exons)	AGAP009342, GSTU3 (218 aa; 2 exons)	LLOJ010488 (221 aa; 4 exons)	FBgn0010044, GstD8 (212 aa; 1 exon)
PPAI010870_b [6] (221 aa; 3 exons)	GSTX	AAEL010500, GSTX2 (218/192 aa; 3/3 exons)	AGAP009342, GSTU3 (218 aa; 2 exons)	LLOJ009529 (225 aa; 3 exons)	FBgn0063494, GstE6 (222 aa; 1 exon)
PPAI010870_c [6]	GSTX	AAEL010500,	AGAP009342,	LLOJ009528	FBgn0010044, GstD8

(221 aa; 3 exons)		GSTX2<br>(218/192 aa; 3/3 exons)	GSTU3<br>(218 aa; 2 exons)	(277 aa; 4 exons)	(212 aa; 1 exon)
PPAI010871_a [7]<br>(221 aa; 3 exons)	GSTX	AAEL010500, GSTX2<br>(218/192 aa; 3/3 exons)	AGAP009342, GSTU3<br>(218 aa; 2 exons)	LLOJ009529<br>(225 aa; 3 exons)	FBgn0010044, GstD8<br>(212 aa; 1 exon)
PPAI010871_b [7]<br>(221 aa; 3 exons)	GSTX	AAEL010500, GSTX2<br>(218/192 aa; 3/3 exons)	AGAP009342, GSTU3<br>(218 aa; 2 exons)	LLOJ009529<br>(225 aa; 3 exons)	FBgn0010044, GstD8<br>(212 aa; 1 exon)
PPAI010871_c [7]<br>(221 aa; 3 exons)	GSTX	AAEL010500, GSTX2<br>(218/192 aa; 3/3 exons)	AGAP009342, GSTU3<br>(218 aa; 2 exons)	LLOJ010539<br>(221 aa; 4 exons)	FBgn0010044, GstD8<br>(212 aa; 1 exon)
PPAI010871_d [7]<br>(221 aa; 3 exons)	GSTX	AAEL010500, GSTX2<br>(218/192 aa; 3/3 exons)	AGAP009342, GSTU3<br>(218 aa; 2 exons)	LLOJ009528<br>(277 aa; 4 exons)	FBgn0010043, GstD7<br>(224 aa; 1 exon)
PPAI010871_e [7]<br>(cannot reconstruct an in-frame gene model)	GSTX	-	-	-	-
PPAI010872<br>(220 aa; 3 exons)	GSTX	AAEL010500, GSTX2<br>(218/192 aa; 3/3 exons)	AGAP009342, GSTU3<br>(218 aa; 2 exons)	LLOJ009529<br>(225 aa; 3 exons)	FBgn0063497, GstE3<br>(220 aa; 1 exon)
PPAI008305_a [8]<br>(>114 aa; >1 exon; partial - C-terminal end missing)	GSTX	AAEL010500, GSTX2<br>(218/192 aa; 3/3 exons)	AGAP004163, GSTD7<br>(218 aa; 3 exons)	LLOJ010963<br>(221 aa; 3 exons)	FBgn0038020 GstD9<br>(218/218 aa; 1/1 exon)
PPAI006902_a<br>+<br>PPAI000943_a [9]<br>(>201/204 aa; >3/4 exons)	GSTZ	AAEL011934, GSTZ1<br>(233/219 aa; 3/4 exons)	AGAP002898, GSTZ1<br>(222/263/219/219 aa; 3/5/4/4 exons)	LLOJ000305<br>(219 aa; 5 exons)	FBgn0037697, GstZ2<br>(220/215/227 aa; 4/4/3 exons)
PPAI007443_a [10]<br>(151 aa; 3 exons)	Microsomal	AAEL006829<br>(149 aa; 3 exons)	AGAP000165, GSTMS1<br>(151 aa; 2 exons)	LLOJ002060<br>(151 aa; 3 exons)	FBgn0025814, Mgstl<br>(151/151/152 aa; 2/2/2 exons)
PPAI007443_b [10]<br>(153 aa; 3 exons)	Microsomal	AAEL006829<br>(149 aa; 3 exons)	AGAP000165, GSTMS1<br>(151 aa; 2 exons)	LLOJ002060<br>(151 aa; 3 exons)	FBgn0025814, Mgstl<br>(151/151/152 aa; 2/2/2 exons)
PPAI007443_c [10]<br>(151 aa; 3 exons)	Microsomal	AAEL006829<br>(149 aa; 3 exons)	AGAP000165, GSTMS1<br>(151 aa; 2 exons)	LLOJ010490<br>(151 aa; 3 exons)	FBgn0025814, Mgstl<br>(151/151/152 aa; 2/2/2 exons)
PPAI007443_d [10]<br>(141 aa; 3 exons)	Microsomal	AAEL023181, (148 aa; 2 exons) / AAEL010157 (148 aa; 2 exons)	AGAP000163, GSTMS2 (152/153 aa; 3/3 exons)	LLOJ002060<br>(151 aa; 3 exons)	FBgn0025814, Mgstl<br>(151/151/152 aa; 2/2/2 exons)

[1] Modified from original gene PPAI006595 (169 aa; 1 exon)
[2] Modified from original gene PPAI000142 (50 aa; 2 exons), with an additional unannotated exon on a different scaffold
[3] Modified from original gene PPAI002540 (158 aa; 2 exons)
[4] Modified from original gene PPAI003974 (258 aa; 2 exons)
[5] Modified from original gene PPAI000341 (175 aa; 2 exons)
[6] Modified from original gene PPAI010870 (399 aa; 7 exons)
[7] Modified from original gene PPAI010871 (840 aa; 13 exons)
[8] Modified from original gene PPAI008305 (117 aa; 1 exon)
[9] Modified and joined two partial GSTZ genes (PPAI006902, PPAI000943) into one (still incomplete - lacking a middle exon) gene model
[10] Modified from original gene PPAI007443 (504 aa; 10 exons)
