# Supplementary material for: Characterization of the glutathione S‐transferase genes in the sand flies Phlebotomus papatasi and Lutzomyia longipalpis shows expansion of the novel glutathione S‐transferase xi (X) class
Source: Insect Mol Biol. 2022 Mar 8;31(4):417–33. doi: 10.1111/imb.12769 (PMC9540044; doi:10.1111/imb.12769)
Supplement: Supplementary file 4 — Table S2. Glutathione S‐Transferase genes in Lutzomyia longipalpis. The gene ID, protein length (amino acids) and number of exons are shown for L. longipalpis genes and their best matching genes in Aedes aegypti, Anopheles gambiae and Drosophila melanogaster. Gene IDs followed by a letter (e.g., ‘_a’) were manually edited from the original gene model. [file IMB-31-417-s005.pdf]

Supplementary Table 2. Glutathione S-Transferase genes in *Lutzomyia longipalpis*. The gene ID, protein length (amino acids) and number of exons are shown for *L. longipalpis* genes and their best matching genes in *Aedes aegypti*, *Anopheles gambiae* and *Drosophila melanogaster*. Gene IDs followed by a letter (e.g. "_a") were manually edited from the original gene model.

Lutzomyia longipalpis	**Subfamily**	***Aedes aegypti***	***Anopheles gambiae***	***Phlebotomus papatasi***	***Drosophila melanogaster***
LLOJ007285<br>(220 aa; 3 exons)	GSTD	AAEL001090,<br>GSTD7<br>(218 aa; 4 exons)	AGAP004163,<br>GSTD7<br>(218 aa; 3 exons)	PPAI001211<br>(217 aa; 3 exons)	FBgn0038029, GSTD11<br>(222/243 aa; 3/3 exons)
LLOJ004462, GSTD<br>(212 aa; 3 exons)	GSTD	AAEL001061,<br>GSTD1<br>(216/209/211 aa;<br>2/2/2 exons)	AGAP004164,<br>GSTD1-4<br>(209/209/216/186<br>aa; 2/2/2/2 exons)	PPAI006595<br>(169 aa; 1 exon)	FBgn0001149, GSTD1<br>(209/209 aa; 1/1 exon)
LLOJ004461_a [1]<br>(215 aa; 3 exons)	GSTD	AAEL001061,<br>GSTD1<br>(216/209/211 aa;<br>2/2/2 exons)	AGAP004164,<br>GSTD1-4<br>(209/209/216/186<br>aa; 2/2/2/2 exons)	PPAI006595<br>(169 aa; 1 exon)	FBgn0001149, GSTD1<br>(209/209 aa; 1/1 exon)
LLOJ004461_b [1]<br>(217 aa; 3 exons)	GSTD	AAEL001061,<br>GSTD1<br>(216/209/211 aa;<br>2/2/2 exons)	AGAP004164,<br>GSTD1-4<br>(209/209/216/186<br>aa; 2/2/2/2 exons)	PPAI006595<br>(169 aa; 1 exon)	FBgn0001149, GSTD1<br>(209/209 aa; 1/1 exon)
LLOJ004461_c [1]<br>(213 aa; 3 exons)	GSTD	AAEL001061,<br>GSTD1<br>(216/209/211 aa;<br>2/2/2 exons)	AGAP004164,<br>GSTD1-4<br>(209/209/216/186<br>aa; 2/2/2/2 exons)	PPAI006595<br>(169 aa; 1 exon)	FBgn0001149, GSTD1<br>(209/209 aa; 1/1 exon)
LLOJ004461_d [1]<br>(217 aa; 3 exons)	GSTD	AAEL001061,<br>GSTD1<br>(216/209/211 aa;<br>2/2/2 exons)	AGAP004164,<br>GSTD1-4<br>(209/209/216/186<br>aa; 2/2/2/2 exons)	PPAI006595<br>(169 aa; 1 exon)	FBgn0001149, GSTD1<br>(209/209 aa; 1/1 exon)
LLOJ004461_e [1]<br>(218 aa; 3 exons)	GSTD	AAEL001061,<br>GSTD1<br>(216/209/211 aa;<br>2/2/2 exons)	AGAP004164,<br>GSTD1-4<br>(209/209/216/186<br>aa; 2/2/2/2 exons)	PPAI006595<br>(169 aa; 1 exon)	FBgn0001149, GSTD1<br>(209/209 aa; 1/1 exon)
LLOJ004461_f [1]<br>(222 aa; 3 exons)	GSTD	AAEL001061,<br>GSTD1<br>(216/209/211 aa;<br>2/2/2 exons)	AGAP004164,<br>GSTD1-4<br>(209/209/216/186<br>aa; 2/2/2/2 exons)	PPAI006595<br>(169 aa; 1 exon)	FBgn0001149, GSTD1<br>(209/209 aa; 1/1 exon)
LLOJ004461_g [1]<br>(218aa; 3 exons)	GSTD	AAEL001061,<br>GSTD1<br>(216/209/211 aa;<br>2/2/2 exons)	AGAP004164,<br>GSTD1-4<br>(209/209/216/186<br>aa; 2/2/2/2 exons)	PPAI006595<br>(169 aa; 1 exon)	FBgn0001149, GSTD1<br>(209/209 aa; 1/1 exon)
LLOJ004460_a [2]<br>(>128 aa; 3 exons; partial - internal gap)	GSTD	AAEL001061,<br>GSTD1<br>(216/209/211 aa;<br>2/2/2 exons)	AGAP004164,<br>GSTD1-4<br>(209/209/216/186<br>aa; 2/2/2/2 exons)	PPAI006595<br>(169 aa; 1 exon)	FBgn0001149, GSTD1<br>(209/209 aa; 1/1 exon)
LLOJ004460_b [2]<br>(>176 aa; 3 exons; partial - C-terminal gap)	GSTD	AAEL001061,<br>GSTD1<br>(216/209/211 aa;<br>2/2/2 exons)	AGAP004164,<br>GSTD1-4<br>(209/209/216/186<br>aa; 2/2/2/2 exons)	PPAI006595<br>(169 aa; 1 exon)	FBgn0001149, GSTD1<br>(209/209 aa; 1/1 exon)
LLOJ002711_a [3]<br>(>112 aa; ~2 exons)	GSTI	AAEL011752,<br>GSTI1<br>(231 aa; 2 exons)	AGAP000947,<br>GSTU1<br>(233 aa; 2 exons)	PPAI009870<br>(229 aa; 2 exons)	FBgn0250732,<br>GST-containing FLYWCH zinc-finger protein, gfzf<br>(234/1045/1045 aa; 2/4/4 exons)
LLOJ002711_b [3]<br>(>161 aa; ~2 exons)	GSTI	AAEL011752,<br>GSTI1<br>(231 aa; 2 exons)	AGAP000947,<br>GSTU1<br>(233 aa; 2 exons)	PPAI009870<br>(229 aa; 2 exons)	FBgn0250732,<br>GST-containing FLYWCH zinc-finger protein, gfzf<br>(234/1045/1045 aa; 2/4/4

					exons)
LLOJ002711_c [3] (>87 aa; ~2 exons)	GSTI	AAEL011752, GSTI1 (231 aa; 2 exons)	AGAP000947, GSTU1 (233 aa; 2 exons)	PPAI009870 (229 aa; 2 exons)	FBgn0250732, GST-containing FLYWCH zinc-finger protein, gfzf (234/1045/1045 aa; 2/4/4 exons)
LLOJ009136_a [4], GSTO2 (253 aa; 4 exons)	GSTO	AAEL017085, GSTO1 (257 aa; 4 exons)	AGAP005749, GSTO1 (248 aa; 3 exons)	PPAI000142 (50 aa; 2 exons)	FBgn0035907, GstO1 (254 aa; 4 exons)
LLOJ009036_a [5] (>44 aa; >1 exon)	GSTS	AAEL011741, GSTS1 (237/237/203/203 aa; 4/5/3/4 exons)	AGAP010404, GSTS1 (203/203 aa; 3/4 exons)	No matches	FBgn0010226, GstS1 (249/249/249/250 aa; 4/4/4/4 exons)
LLOJ009037, GSTS1 (203 aa; 3 exons)	GSTS	AAEL011741, GSTS1 (237/237/203/203 aa; 4/5/3/4 exons)	AGAP010404, GSTS1 (203/203 aa; 3/4 exons)	PPAI002540, GSTS1 (158 aa; 2 exons)	FBgn0010226, GstS1 (249/249/249/250 aa; 4/4/4/4 exons)
LLOJ005757 (226 aa; 2 exons)	GSTT	AAEL009017, GSTT1 (229 aa; 3 exons)	AGAP000761, GSTT1 (229 aa; 3 exons)	PPAI003974 (258 aa; 2 exons)	FBgn0031117, GstT3 (228/228/268 aa; 3/3/4 exons)
LLOJ002025 (227 aa; 3 exons)	GSTT	AAEL025929 (231 aa; 2 exons)	AGAP000761, GSTT1 (229 aa; 3 exons)	PPAI003974 (258 aa; 2 exons)	FBgn0031117, GstT3 (228/228/268 aa; 3/3/4 exons)
LLOJ008830_a [6] (229 aa; 3 exons)	GSTT	AAEL009017, GSTT1 (229 aa; 3 exons)	AGAP000761, GSTT1 (229 aa; 3 exons)	PPAI003974 (258 aa; 2 exons)	FBgn0031117, GstT3 (228/228/268 aa; 3/3/4 exons)
LLOJ002682_a [7] (230 aa; 3 exons)	GSTT	AAEL025929 (231 aa; 2 exons)	AGAP000761, GSTT1 (229 aa; 3 exons)	PPAI003974 (258 aa; 2 exons)	FBgn0031117, GstT3 (228/228/268 aa; 3/3/4 exons)
LLOJ001842_a [8] (221 aa; 4 exons)	GSTX	AAEL010500, GSTX2 (218/192 aa; 3/3 exons)	AGAP009342, GSTU3 (218 aa; 2 exons)	PPAI010868 (221 aa; 4 exons)	FBgn0038029, GstD11 (222/243 aa; 3/3 exons)
LLOJ001842_b [8] (>167 aa; >3 exons; partial - C-terminal end missing)	GSTX	AAEL010500, GSTX2 (218/192 aa; 3/3 exons)	AGAP009342, GSTU3 (218 aa; 2 exons)	PPAI010868 (221 aa; 4 exons)	FBgn0038029, GstD11 (222/243 aa; 3/3 exons)
LLOJ001842_c [8] (>109 aa; >2 exons; partial - N-terminal end missing)	GSTX	AAEL010500, GSTX2 (218/192 aa; 3/3 exons)	AGAP009342, GSTU3 (218 aa; 2 exons)	PPAI010868 (221 aa; 4 exons)	FBgn0010039, GstD3 (199 aa; 1 exon)
LLOJ001842_d [8] (221 aa; 4 exons)	GSTX	AAEL010500, GSTX2 (218/192 aa; 3/3 exons)	AGAP009342, GSTU3 (218 aa; 2 exons)	PPAI010868 (221 aa; 4 exons)	FBgn0038029, GstD11 (222/243 aa; 3/3 exons)
LLOJ001842_e [8] (222 aa; 4 exons)	GSTX	AAEL010500, GSTX2 (218/192 aa; 3/3 exons)	AGAP009342, GSTU3 (218 aa; 2 exons)	PPAI010868 (221 aa; 4 exons)	FBgn0063494, GstE6 (222 aa; 1 exon)
LLOJ001842_f [8] (221 aa; 4 exons)	GSTX	AAEL010500, GSTX2 (218/192 aa; 3/3 exons)	AGAP009342, GSTU3 (218 aa; 2 exons)	PPAI010868 (221 aa; 4 exons)	FBgn0063494, GstE6 (222 aa; 1 exon)

LLOJ001842_g [8]<br>(221 aa; 4 exons)	GSTX	AAEL010500, GSTX2<br>(218/192 aa; 3/3 exons)	AGAP009342, GSTU3<br>(218 aa; 2 exons)	PPAI010868<br>(221 aa; 4 exons)	FBgn0038029, GstD11<br>(222/243 aa; 3/3 exons)
LLOJ009529<br>(225 aa; 3 exons)	GSTX	AAEL010500, GSTX2<br>(218/192 aa; 3/3 exons)	AGAP009342, GSTU3<br>(218 aa; 2 exons)	PPAI010872<br>(220 aa; 3 exons)	FBgn0001149, GstD1<br>(209/209 aa; 1/1 exon)
LLOJ009528_a [9]<br>(221 aa; 3 exons)	GSTX	AAEL010500, GSTX2<br>(218/192 aa; 3/3 exons)	AGAP009342, GSTU3<br>(218 aa; 2 exons)	PPAI010869<br>(221 aa; 3 exons)	FBgn0010043, GstD7<br>(224 aa; 1 exon)
LLOJ009528_b [9]<br>(221 aa; 3 exons)	GSTX	AAEL010500, GSTX2<br>(218/192 aa; 3/3 exons)	AGAP009342, GSTU3<br>(218 aa; 2 exons)	PPAI010872<br>(220 aa; 3 exons)	FBgn0010044, GstD8<br>(212 aa; 1 exon)
LLOJ010488<br>(221 aa; 4 exons)	GSTX	AAEL010500, GSTX2<br>(218/192 aa; 3/3 exons)	AGAP009342, GSTU3<br>(218 aa; 2 exons)	PPAI010868<br>(221 aa; 4 exons)	FBgn0063497, GstE3<br>(220 aa; 1 exon)
LLOJ010539<br>(221 aa; 4 exons)	GSTX	AAEL010500, GSTX2<br>(218/192 aa; 3/3 exons)	AGAP009342, GSTU3<br>(218 aa; 2 exons)	PPAI010868<br>(221 aa; 4 exons)	FBgn0010044, GstD8<br>(212 aa; 1 exon)
LLOJ010538<br>(222/221 aa; 4/4 exons)	GSTX	AAEL010500, GSTX2<br>(218/192 aa; 3/3 exons)	AGAP009342, GSTU3<br>(218 aa; 2 exons)	PPAI010868<br>(221 aa; 4 exons)	FBgn0001149, GstD1<br>(209/209 aa; 1/1 exon)
LLOJ010537<br>(221 aa; 4 exons)	GSTX	AAEL010500, GSTX2<br>(218/192 aa; 3/3 exons)	AGAP009342, GSTU3<br>(218 aa; 2 exons)	PPAI010868<br>(221 aa; 4 exons)	FBgn0001149, GstD1<br>(209/209 aa; 1/1 exon)
LLOJ010536<br>(221 aa; 4 exons)	GSTX	AAEL010500, GSTX2<br>(218/192 aa; 3/3 exons)	AGAP009342, GSTU3<br>(218 aa; 2 exons)	PPAI010868<br>(221 aa; 4 exons)	FBgn0038029, GstD11<br>(222/243 aa; 3/3 exons)
LLOJ010535_a [10]<br>(>192 aa; >3 exons; partial - N-terminal end missing)<br>LLOJ010534 [10]<br>(>191 aa; >3 exons; partial - N-terminal end missing)	GSTX	AAEL010500, GSTX2<br>(218/192 aa; 3/3 exons)	AGAP009342, GSTU3<br>(218 aa; 2 exons)	PPAI010868<br>(221 aa; 4 exons)	FBgn0010041, GstD5<br>(216 aa; 1 exon)<br>FBgn0038029, GstD11<br>(222/243 aa; 3/3 exons)
LLOJ010533_a [11]<br>(221 aa; 4 exons)	GSTX	AAEL010500, GSTX2<br>(218/192 aa; 3/3 exons)	AGAP009342, GSTU3<br>(218 aa; 2 exons)	PPAI010868<br>(221 aa; 4 exons)	FBgn0001149, GstD1<br>(209/209 aa; 1/1 exon)
LLOJ010532<br>(221 aa; 4 exons)	GSTX	AAEL010500, GSTX2<br>(218/192 aa; 3/3 exons)	AGAP009342, GSTU3<br>(218 aa; 2 exons)	PPAI010868<br>(221 aa; 4 exons)	FBgn0038029, GstD11<br>(222/243 aa; 3/3 exons)
LLOJ010530<br>(221 aa; 4 exons)	GSTX	AAEL010500, GSTX2<br>(218/192 aa; 3/3 exons)	AGAP009342, GSTU3<br>(218 aa; 2 exons)	PPAI010868<br>(221 aa; 4 exons)	FBgn0038029, GstD11<br>(222/243 aa; 3/3 exons)
LLOJ010531_a [12]<br>(>115 aa; >2 exons; partial - C-terminal end missing)	GSTX	AAEL010500, GSTX2<br>(218/192 aa; 3/3 exons)	AGAP009342, GSTU3<br>(218 aa; 2 exons)	PPAI010868<br>(221 aa; 4 exons)	FBgn0038029, GstD11<br>(222/243 aa; 3/3 exons)
LLOJ010531_b [12]	GSTX	AAEL010500,	AGAP009342,	PPAI010868	FBgn0038029, GstD11

(>115 aa; >2 exons; partial - C-terminal end missing)		GSTX2 (218/192 aa; 3/3 exons)	GSTU3 (218 aa; 2 exons)	(221 aa; 4 exons)	(222/243 aa; 3/3 exons)
LLOJ010963 (221 aa; 3 exons)	GSTX	AAEL010500, GSTX2 (218/192 aa; 3/3 exons)	AGAP009342, GSTU3 (218 aa; 2 exons)	PPAI010869 (221 aa; 3 exons)	FBgn0010044, GstD8 (212 aa; 1 exon)
LLOJ000305_a [13] (216/219 aa; 4/5 exons)	GSTZ	AAEL011934, GSTZ1 (233/219 aa; 3/4 exons)	AGAP002898, GSTZ1 (222/263/219/219 aa; 3/5/4/4 exons)	PPAI000943 (141 aa; 2 exons)	FBgn0037697, GstZ2 (220/215/227 aa; 4/4/3 exons)
LLOJ002060 (151 aa; 3 exons)	Microsomal	AAEL006829 (149 aa; 3 exons)	AGAP000165, GSTMS1 (151 aa; 2 exons)	PPAI007443 (504 aa; 10 exons)	FBgn0025814, Mgstl (151/151/152 aa; 2/2/2 exons)
LLOJ010490 (151 aa; 3 exons)	Microsomal	AAEL006829 (149 aa; 3 exons)	AGAP000165, GSTMS1 (151 aa; 2 exons)	PPAI007443 (504 aa; 10 exons)	FBgn0025814, Mgstl (151/151/152 aa; 2/2/2 exons)
LLOJ008423_a [14] (>107 aa; >2 exons)	Microsomal	AAEL006829 (149 aa; 3 exons)	AGAP000163, GSTMS2 (152/153 aa; 3/3 exons)	PPAI007443 (504 aa; 10 exons)	FBgn0025814, Mgstl (151/151/152 aa; 2/2/2 exons)
LLOJ004345_a [15] (153 aa; 2 exons)	Microsomal	AAEL023181, (148 aa; 2 exons) / AAEL010157 (148 aa; 2 exons)	AGAP000165, GSTMS1 (151 aa; 2 exons)	PPAI007443 (504 aa; 10 exons)	FBgn0025814, Mgstl (151/151/152 aa; 2/2/2 exons)

[1] Modified from original gene LLOJ004461 (1034 aa; 12 exons)
[2] Modified from original gene LLOJ004460 (195 aa; 3 exons)
[3] Modified from original gene LLOJ002711 (339 aa; 4 exons)
[4] Modified from original gene LLOJ009136 (268 aa; 4 exons)
[5] Modified from original gene LLOJ009036 (64 aa; 2 exons)
[6] Modified from original gene LLOJ008830 (786 aa; 9 exons)
[7] Modified from original gene LLOJ002682 (446 aa; 6 exons)
[8] Modified from original gene LLOJ001842 (1202 aa; 24 exons)
[9] Modified from original gene LLOJ009528 (277 aa; 4 exons)
[10] LLOJ010535_a and LLOJ010534 both partial isoforms (lacking shared exon 1) of a single gene, paralogous to LLOJ010538
[11] Modified from original gene LLOJ010533 (207 aa; 4 exons)
[12] Modified from original gene LLOJ010531 (99 aa; 2 exons)
[13] Modified from original gene LLOJ000305 (219 aa; 5 exons)
[14] Modified from original gene LLOJ008423 (98 aa; 2 exons)
[15] Modified from original gene LLOJ004345 (102 aa; 2 exons)
