# Supplementary material for: Characterization of the glutathione S‐transferase genes in the sand flies Phlebotomus papatasi and Lutzomyia longipalpis shows expansion of the novel glutathione S‐transferase xi (X) class
Source: Insect Mol Biol. 2022 Mar 8;31(4):417–33. doi: 10.1111/imb.12769 (PMC9540044; doi:10.1111/imb.12769)
Supplement: Supplementary file 5 — Appendix S1. Supporting information. [file IMB-31-417-s002.pdf]

Appendix 1

## Manual editing of gene annotation
## Previous coding exon annotation indicated by bold and underlined text (some in italic)
## Manually edited exon annotation indicated by colour highlights
## (for multiple genes, different colours indicate different genes)
## Predicted protein(s) after editing are shown beneath each annotated genomic region

#########################
####### GST delta #######
#########################

####### PPAI001211 - GSTD #######
>JH662257.1 reverse | **PPAI001211** (pre-editing) | PPAI001211 (post-editing)
TTCATAACCTTTTATTGATTGGGAAATTCTTTTTTGCCATTTATTTCAAACACGTGATTTGTCGCAAATTAGAGATCCATAGGAAATTCCTATACATGCTTTGTCTTG
AGTTTTTCTATATATTATTTCGCTTTTTATACTATAATAAATTGTATAATAGAAGAATTATGTAAAACATATCCGATTCTAGGGGTGTAAAA**ATGCCTGTGAAACTTTT**
CTATTTGCCTGAGAGTCCACCCTGTCGCACGGTTTTGCTATGTGGGCGACTCTTGAATGTTAATTTTGACCTCAAAGTCGTAAATATTCTCAATGGAGAGCAGCTTAAACCT
GAATTCATACAAGTAAGTCCTTATATTTATAATAAATTCAAAGTACAGATAACTCAATTTTTCTTAACAG**TTAAATCCTCAGCACTGCATTCCAACCATGGAAGACAATG**
ATCTCGTGCTTTGGGAGAGCCGAGTAATTCTAATGTATTTAGTGTCAGCTTATGCAGAAGATGACACTTTGTACCCCAAAGATCTCCAAAAACGAGCGATGGTTGATCAACG
AATTCATTTTGACCTAGGGACTCTCTACCAAAGAGCAGGAGACTATTTTGTAAAATTTTAATTTGTTATACTCATAAAGAAACAAAATAATTTTATTTTATTTTTTTACA
G**TTTCCTACACTTTTCTTTGGGGCCCATTTGGATGAAACGAAAAAAGCTCGCCTGGCTGAGGCATTAGGATGGCTTGAAGATATTCTCAAAGGTCGTACTTGGGTAGCAACG**
GAAAACTTTACTATTGCGGATCTCATTCTTTGTGTAACTATATCGCAAATTGAGGCTTTTGGATTTGAATTAGGACCCTACCCAAAGATAAGAGCCTGGTTCCAACTGTGCA
AAGAAAAACTGGAGCCTTATGGATATGAAGAAATTAATGAAGCCGGAGCAAACGCTCTTGCAGACCTTTTTAGATCTAAATTAAATTGATTGTTGTTGTGAATATAATTTT
TGAGCAAAATACTTACTTTTCATCTACATACCTAATTCACTCTAAATCCTTTTGCATGCAAGTTGTTTATTTACGTCCTAGATAATGGTTTTAGAACACACTTTTTGT
TGCTCAAATATAGAATATCAATACTATTTAGATATGTGAAAAATTCGAATATATTAGGGTTTTTATTTGG

>PPAI001211 (217 aa; 3 exons) | UNCHANGED
MPVKLFYLPESPPCRTVLLCGRLLNVNFDLKVVNILNGEQLKPEFIQLNPQHCIPTMEDNDLVLWESRVILMYLVSAYAEDDTLYPKDLQKRAMVDQRIHFDLGTLYQ
RAGDYFFPTLFFGAHLDETKKARLAEALGWLEDILKGRTWVATENFTIADLILCVTISQIEAFGFELGPYPKIRAWFQLCKEKLEPYGYEEINEAGANALADLFRSKL
N-

####### PPAI006595 - GSTD #######
>AJVK01058452.1 forward | **PPAI006595** (pre-editing) | PPAI006595_a (post-editing)
ACACAATATATTAGCGGGATTTTCTATGGATGCCCAGGCGGAAAAATAACAGAAAAAATCCACGGATTTTTCTTTTAAAGCGAAACTTTGTGGCCGATCGGCTAAAGG
GACTGTAGATGGAAACGTCAAACTTTTGTCAAAAATGCAATTTTTGACGAAAATTGCTTATATTGTGTAGAGGCCTTAAAGCTGCTATTCAAATGAAAAATGAACATA
TTTTTTTTAGTATCTTATTATTCTTAAGTGTTTCTGCCTTATCGACTTTCTATGCATTGGTTCCTGTCTCGACTGTTTTTCTAAGTCTTCGCAGTGTTCTAAAACCAC
CGAACAGTCGTGACAGGAACCAACACAAAGAAAAGTCGATAATACAGAAGCACTCGAGGACAGTCGTGTGCAAAAAAATTAATCTCTTTTCGTTTTGTTTTAGGAAAT
AAAAAAACCGCTCTAAAAATCCTAATTTAACTCAAATATCTTAATTTTCTTTCTAATAG**GTTAATTATCAGCATTGTGTGCCAACTCTCGTGGACAATGGCTTTTCTCTT**
TGGGAATCCCGTGCCATTTTGGCGTATCTCGTGGAGAAGTACGGAAAGGATGATGCCCTGTACCCCAAGGATCCCGAGAACAGAGCTCGAGTGAACCAAATGCTCTACTTCG
ACATGGGAACTCTCTATCAGCGTTTTGCTGAGTACTATTACCCAGCAATCTTCGCCAAGGCTCCTTTCAGTCCTGATGGATTCAAGAAGATGGAAGATGCCGTTGGTTTCCT
CAATACCGGGCTCGAAGGACGAAAATTCGCTGCCGGAGATACTCTTACCATCGCCGATATGGCTCTTGTAGCCACAGTTGCCACCTACGATGTCTTTAAATTCGACTTCTCC
AAGTATCCCAACGTGACCAGATGGTACGAGAGCTGCAAAAAGACCATTTCTGGCTACGATGAGATCAACCAAAAGGGAGCTGATGAGTTCTTCGCGAAATTCGGTGCTGTTC
TCAACCAATAAATGCAGAGAGAACCTTCCGGGAGCCTTTTCTCCTCAACAACAATTCCCATTGCTAGCTAGTACTTAGGCAACTTTAGAGACGTGAGACCTCAGACAAT
CACCAGATTGTAAATTAAAATACCTAGAAAATACCTAAAACAATGTGTAAATACTGTATAAATGATCTGAAGAGTTTTATAAAAAAAGATTTTATGAATGAA

>PPAI006595_a (224 aa; 3 exons) | EDITED TO ADD 2 EXONS
MKNEHIFFSILLFLSVSALSTFYALVPVSTVFLSLRSVLKPPNKKSIIQKHSRTVVNYQHCVPTLVDNGFSLWESRAILAYLVEKYGKDDALYPKDPENRARVNQMLY
FDMGTLYQRFAEYYYPAIFAKAPFSPDGFKKMEDAVGFLNTGLEGRKFAAGDTLTIADMALVATVATYDVFKFDFSKYPNVTRWYESCKKTISGYDEINQKGADEFFA
KFGAVLNQ-

####### LLOJ007285 - GSTD #######
>JH689876 reverse | **LLOJ007285** (pre-editing) | LLOJ007285 (post-editing)
TTCTTTTGCCATTTTTTTCGCAGAGGCGTGGAGGTAACAACCACGTGCTCAACCCTTAGTGAAATAAATTAAAGGGTCGCATAGAATTCCCATGCGCTGAGTTATGTT
TTTTTTCTCCTTTATCGCTTGTTTACTTCTTTTTTGCCTTGTGTTATGAGAGGGGAAATACCTTTGCAAAAATATCTTTAAGGGGCATCAAA**ATGCCAGTAAAGCTGTA**
CTACTTGCCCGAAAGTCCACCCTGTAGGACAATTTTACTCCTTGGGAGACTCCTGAAAATTGATTTTGATCTCAAAATTGTCAATATACTCGATGGGGATCAACTTAAACCG
GATTTTGTGCAAGTAAAAGCTCAAAATGAATTATTTAAACCTTCAAAATTTAATTAATAAATTCCTGGCAG**TTGAATCCGCAACACTGCATCCCTACAATGGATGACAAT**
GATCTTGTTCTGTGGGAGAGCCGCGTAATTCTTATGTATTTGGTGTCAGCCTATGCTGAGGATGATATTCTGTACCCAAAGGACATCCTACAGAGAGCAATGGTGGATCAAC
GAATCCATTTTGATTTGGGTACTCTCTACCAAAGAGCTTCTGATTATTTCGTAAGGATTCTTTACTATAAAAACGAAATATTCCTTAAATAATTCCAAGGAATTGAAAGG
TAAATTTTTACCATTTGTAG**TTTCCCACACTATTTGTTGGAGCACATTTAGATGAAACTAAAAAAGCTCGCCTAGCTGAGGCCCTAGGATGGTTCGATGACATCCTTAAAG**
GACGCAAATGGGTGGCAACGGACAATTTTACAATCGCCGATCTCACTCTGTGCGTCACTGTGTCACAAATTGAAGCATTTGGATTTGAATTGGGTCCATATGGTCGTGTCAG
GGCATGGTTTCAGCACTGCAAAGATGAACTTGAGCCCCATGGATACGATGAGATCAATCAGGTGGGAGCAGATGCCCTCGGAGAAATGTTTCGGTCGAAATTGCAACAAGAG

AAATAATTTTTTTCTTTAAACTGTTGAACGATTAATAAAAGTTATAAAAATGCTGGTTAAATGAATTTTTGTTATGCCCTAGATGTCGCTACAACATTATTCGGCCCT
CTGGTGGAAAAAATCCCGTTTCGCGTTTAAATAGAATTTTATTACTTTCCTTCGACAATAAATCCACATTCAAAACGACAGAATGTAATATTAGTTAC

>LLOJ007285 (220 aa; 3 exons) | UNCHANGED
MPVKLYYLPESPPCRTILLLGRLLKIDFDLKIVNILDGDQLKPDFVQLNPQHCIPTMDDNDLVLWESRVILMYLVSAYAEDDILYPKDILQRAMVDQRIHFDLGTLYQ
RASDYFFPTLFVGAHLDETKKARLAEALGWFDDILKGRKWVATDNFTIADLTLCVTVSQIEAFGFELGPYGRVRAWFQHCKDELEPHGYDEINQVGADALGEMFRSKL
QQEK-

####### *LLOJ004462*, LLOJ004461, *LLOJ004460* (*LLOJ004462* and *LLOJ004460* in italics) - GSTD #######
>JH689583 reverse | ***LLOJ004462***, **LLOJ004461**, ***LLOJ004460*** (pre-editing) | LLOJ004462, LLOJ004461_a,
LLOJ004461_b, LLOJ004461_c, LLOJ004461_d, LLOJ004461_e, LLOJ004461_f, LLOJ004461_g, LLOJ004460_a,
LLOJ004460_b (post-editing)
CATTTCTAAAATCAATTAATTTTCCAATAATTCATGAATGTGATCTTAATTTTATTTCCTCAACTGTGTGAATTGCTTCATTAAATGTTTGAGCACCTAAAACAAGAA
ATCAAACACCTTAATGATTATTCTTCAATTTGTAGGGCACATTGAATCAACTTTGGAAGTCCTAAATATTTGATCAACTCATCAATCTAAAC***ATGGATTTGTACTACCT***
TCCGGGATCTGCCCCCTGCCGCAGCGTTCTCCTGTGTGGAAAAGCTCTTGGCCTTGATTTCAATCTCAAAACTCTCGGTAGGTTTTATCAGACTTATTAGCCCTTTAATTT
TTTTTGACCTTTACTGATTCTTTTTCATGGTTTACTCACTCGTCAAAAAATTAGACATTTTATCTAATAATAAATCGTTATTATATAATTATAG***ATTTAATGGCTGGAG***
AGCACTTGAAACCGGAATTCCTAAAGGTAAGTTGAATAGAGTACAATGACCTACATTTTCGTCATGCCACCAACGAAAAACGTGATGGGCTATCATTTTCTTAAAGGTT
TCAATCCGTAATTTTGAATCAACGCCCAATGATATGACTTGACAGAAATTTTGGTATAGATAAGATTAGAATTGAGTTTCATAATTAGACGGATTTGTCCGTTCTTAT
CTAGCAGCTTGAAATTTCTCTTTGAAAGAAATTGAAAGTAATTGCGTGATATCATTGAAGGTCACCGCATTCATTCATTTTATTACACTGGGTAACATTTTTATTTCT
TTTCTCTCCGATTAG***ATCAACTACCAACACTGCATACCTACTCTTGTGGATAATGGCTTTGCACTGTGGGAATCTCGTGCTATCCTCGCCTATTTGGTGGAGAAATACGGA***
AAGGATGATTCGCTGTACCCGAAGGATCCCCAGAAGAGATATTTTGTGAATCAGAAACTCTACTTCGACATGGGAACACTCTACCAGCGCTTTGCGGATTACTACTATCCTG
TGCTTTTTGCAAAGGCTCCATACAATCCAGAGGCATACAAAAAGATGGAAGAGGCTGTTGGTTTCCTCAATACGGCTCTCGAGGGAAAAACTTATGCTGCTGGAGATAATCT
TACTATTGCTGATCTTGCGCTGGTCGCTACCATTTCAACCTATGATGGCTTCAAATTTGATTTCTCGAAGTATCCCAATGTGGCTAAATGGTATGAGACGTGCAAGAAGATT
CCAGGCTATGAGGTGAATCAAAAGGGTGTTGATGAGTTTGTGGAGAAATTTGGACCACTGCTTCCCAAGTAAATGTGGTTCAAGGAAAATAATTTTTAAATTTTTCTTTTG
CATGCAATTTTCATAAAATTATATTTACTAAAAAAAATGTTCACTTCAGAGAAGAAACTATTAATTTGTTCAGTATAAACTGCATTTTCTAATACATATAAAATAAAT
ACATTTTTGGAAATTTAAATTGAGTTTTTTTTCTATTTTGATGATGCAAAGGATCGGGAATGAAAGAATTGATTGTAACGAAAATTCTTGTTAAGCTTCGCTCTTTTA
AAAGAAAATTGAGTTACAAATTTTATACGCCAACTGTAGAGATATCAGTATTACTCATAAAAATATCTTGCAAATCATCATCGTCGAACACAACATTTCAGACAAACC
GTATAAATAGAAGAAGAGCGACACAATATTTAGTCACATAATTTTGTGTGTTTATTCTGCTGGAACGACTGCAGACCTCTGAATATTTTGAGTTTTATCAGGTAAGTC
GAAATTCACTGCAGCAGCTCAAATTTGAACATTAAAAATAGTTTTCAAACTGGAAAAGCTTTAAAAATCATGTTCTTGTGAAAGAAAATGTGAAAATTATGTGATTAC
GAATTTTTCCTCAGTTGTGAGCAACCCCACCAAGTGATCAACTTCAATCTAATTTTTTCCACGAAGAGGCTTTTAACATTTAGTCAAG**ATGACTATTGATTTTTACTAT**
TCCGCGCTCTCTGCACCCTGTCGGAGCATCCTTCTTCTTGCCAAAACCCTTGGAATTGAATTAAATCTCAAGCCAATGGGTAAGATAAATTGGAAACTTTAGTTATTTTGC
AATTAATGCAGAGAGAAAACATCTTGCTTCTTTAATTTTCTTGTAG**ACTTGATGGCTGGTGATCAGTTGACACCAGAATTTATAAAG**GTATGGTAAAGATCTGCGGAACG
ATGACTTGTTTTTAATAGCAGTAGAAAAAATTAATTGAATGCTCTAATCTCATTGTCAG**CTAAACTATCAGCATACAATTCCAACTCTCGTGGACAATGGTTTTTCAATT**
TGGGAATCACGTGCCATCCTCGCTTATTTGGCGGAAAAGTACGGAAAGAATGACTCGCTCTATCCGAAGGATCCTGAGAAGAAAGCCCGCGTGAACCAAAAGCTCTACTTCG
ATATGGGGGTGCTCTACCAACGCTTTGGTGACTACTACTACAAACCAATCCAAACAAAGACTCCGTATGATCCTGCTGCTTACAAGAAAATGGAAGAGGCTGTCGGTTTCCT
CAATACAGGCCTCGAGGGCCAAAAGTATATTGTTGGTGACACACTGACCGTTGCAGATTTCGCAATCATTGCAACGATCTCAACTTACGATGTTCTCAAATTTGATTTACA
CCCTACCCCAATGTTGTTCAATGGTATGAGAGGTGCAAGAAGACCATGCCAGGATATGAGATTAATCAAAAAGGTGCAGATGAGTTTCTTGAGAAAATCGGTCCTATGCTA
CCAAAATAAGTTGAATTTTTAATTGTTTTGGTATTTCAATGAAAACATAAAAATTGTTCACTCAAAAAATATTCTTTTAATACATGCATTCGAAAACATTAAATTATG
GATTTTATTCTTTTCTGTGTGTGTAAGTTTAGTTTAACTATATAAATAAAGCTTTATTCAGAATATTGGAGTAAAAATCTCACCAACAGCAGCTCTTCTGCATGATTC
TTCTTACCTTGAGATTGGGTTAGCAATTTTAGGCAGCACCTGTTGAGATTGATATCACTGCTACTCATACAATTATCTAACTTTGTATCTACCAAATCATCATCGTCT
GACCTACGAAAAAGCGGACCATATAAGTAGAGGAGTGTCCATAAAATTGTCAGTCACAGATTTCTGTTTTATTGTGAGACATCTCATAGACTTTTGAAGTGTTTTTCG
GGTAAGTAAAGACGTGAGAGAATTGACAGATTGACCAAATAAGGTTTGAGAGCTTTTTCTTATCTAAAAAGAATCGAAGATTAAATGCAGTTTTAAACGAAATAAAAA
AAAACTTTTCTTCGTGTCGTGTGCATGATTTCAAATTAATGTTCAACCGTGATTATCATTACAGAAGTCGTCATAATTTTTTGTGGGTGATCAAATATCTAATCTCTT
CTTTTACAGAGCTTTGGTTGAAAGAGAGCATAAAGTGAACAATACACAATCAAAATGACTCTTGATTTTTACTATTCTCCACTTTCTGCGCCATGCCGGAGCATTCTT
CTTCTTTGCAAAACTCTTGGAATTGAATTAAACCTCAAGGAAATAGGTAAATGAAAATGCAACACTTATTTCTTCTTTTAAATAAAAAAAATGCTGAAATTTAATTTT
TTTACAG**ACATTTTGGGTGGTGAGCACCTGAAACCGGAATTCTTAAAG**GTAATAAAATTGCCAAATTCTCAAAAAAATCAATGTTACAAACTTTTTTTATGAATGAATTT
AAGTCTATACGTAAATAAGATTTTCCTAAATAATTATGTTCTTTTTTACTCCAG**ATCAACTACCAGCACACTATTCCTACGCTAGTGGACAATGGTTTTACAATGTGGGA**
ATCACGTTCTATTCTTGCCTATTTAGTGGAGAAATACGGAAAGGATGATTCGCTGTACCCAACTGATCCTCAGAAGAGAGCTCTTGTGAATCAGAAGCTCTACTTCGATATG
GGAGTACTCTATCAGCGCTTTGCTGACTACTACTACAAACCAATCCAAACGAAGACTCCCCTTGATCCTGCTGCTTACAAGAAGATAGAAGAGGGCCTTGGTTTCCTCAATA
CCGGCCTGGAGGGCCAAAAGTTTGCTGCTGAAGATTCCCTGACTATTGCAGACTTTGCTCTAATTACCACTATCTCAACTTACGATGGCCTTAAATTTAATTTAACGCCTTA
TCCGAATGTGGCTCAATGGTATGAGCGGTGCAAGAAGACCATGCCAGGATATGATCTTAATGTGAAAGGCGTAGAGCAGTTCCTTGAGAAAATTGGTCCTATGCTACCAA
AATTTCCATGAAAATATAAAAATATTTTCAATTAAATAAAATTCTTTTAATACATGCATTCGAAAACAATAAATTATGAATTTATTTTTTCTTTTCTGTGTGTGCAAG
ATTAAGAAAGAAAAATAAATAAATAAAGCTTTATTCAGAATATTGGATTAAAAATCATCACCAACAGCAGCTCTTCTGCATGATTCTTCTTACCTTGAGATTGGGTTA
GCAATTTCAGGCAGCACCTGTTGAGATTGATATCAGTGCTACTCATACAAATATCTACCAAATCATGATCGTCAAACACACGAAATTATGGCCCATATAAGTAGAGGA
GTGTCCATAAGATTGTCAGTCACAGATTTGTGTTTTATTGTGAGACATCTCATAGACTTTTGAAGTGATTTTCGGGTAAGTAAAGACGTGAGAGAATTGACAGATTGA
CCAAATAAGGTTTGAGAGCTTTTTCTTATTTGCAAAGAATCGAAGATTAGATACAGTTTTAAACAAAATAAAATGTATTGTGGGATTATCAATTACAGAAGTTCCTTT
TTTGTAATTGATCCACTCTTAATTTCTTGTTTTACAGAGCTTTAGTTGAAGGAAGCATAAAGTGAACCACTCAACATGACTCTTGATTTTTACTATTCTCCACTATCT
GCGCCATGTCGGAGCATTCTTCTTCTTTGCAAGAATCTTGGAATTGAATTAAACCTCAAGGAAATAGGTAAAAGAAAACACTTTCGTCTTTACAATTAATGAATGAAA
TAAATTCTGTTTCATTTTCTTCAAGACATTTTGGGTGGCGAGCACTTGAAACCGGAATTTTTAAAAGTAAGTAATAAAATTTTCATCAGGAATGACTTGCTCTTATTC
TCATGGACACGATAGCAAAAAAAATATCTATGTTAGTGTCTTTTAAATGTGAATGAATTTAAATTGTTTTTCTTTACTCCAG**ATCAACTACCAGCACACCATTCCTACG**
CTGGTGGACAATGGCTTTGCAATGTGGGAATCACGTGCTATCCTCGCCTATTTGGTGGAGAAATACGGAAAGGATGACTCGCTGTACCCGAAGGATCCCCAAAAGAGATATT
TTGTAAATCAAAAGCTGTACTTTGATATGGGAACACTCTATCAGCGCTTTGCGGATTACCACTATCCAGTGATTTTTAAGAAAACCTCATCATACGATCCGGAAGCATTTAA
GAGGATGGAACAGGGTGTTGAATTTCTCAATACAGCCCTCGAGGGGAAGACCTACGTTGTTGGAGATAATCTTACTATTGCTGATCTTGCGCTGGTTGCTACTATTTCAACA
TACGATGGCCTCAAATTTGACTTCTCGAAGTATCCCAATGTGGCTAAATGGTATGAGACGTGCAAGAAGATGCCAGGCTATGAAGTCAACCAGAAGGGTGTGGATAAATTC
GTTGAAATAATGGAATCGAAGAAATAATATTTTTCGTTGTGAATGATTTAAAATTTTACACATTCTTTTTTCAATTAATTCTTGCTCTTTAGAAGTAAATTCCTTGAT
CAATTCATAAGAGAAAATTATTGCGTATATGTTCAAAACATTTTTCATTCTGATTTCTTGAAAATCTTGATATAAAATCAATCTGTTTGACATGGATTTTAATTAAAA

TAAAATAGAAGACATTTTAAAGATCTTTTTAATTTGAAGAAAATCCGAAAATGTACTTTTTGGTCAATTCAAAAAGTACTTAGCTGGACATAAACTGGATTCATCGAC
GTTTACCAGAACCTGTAAATTTTGATTATAAAAAATATATAATTACTCACACAG**AGTGAGTATTCAGCGTACGAAGATCCGTCAAATTTATCA**GTTAGTTGAAAGTTGTA
TTGAGGAATCATCATCATCCCTGACTTCTCTCTTGATTGTACAGAAGCTGCAAAGCTTAGGTATGATTTTTTTTTAATAATTAAAATATTTTAAAAATATAAAACATT
TTATTCATTTCACAATGTTATTTTCCTTTTTTCTTTATAGAGGACCATTTTTCAAGAATTTTCATTCAGAAGAAAAACTAAAGAAGAGTTTCGAAGATGACGATTGAT
TTATACTACTCGCCAGCTTCCTCGCCCTGTCGTAGCATTCTTCTTCTGGGAAAAACTCTTGGAATTCATTTCAACCTCATTGAAACACGTAAGGAAATTATATTTCTT
TTTCTTTTCTAACAAATGTGTAAAATAATTTATTTAAAATTTCTTCTTATAGGGTTATGTAGAGGGGATAATTTCAGGCCGGAATTCTCAAGGGTAATATCAAAAATT
CTCTATTTTCATTGTAATTTTTAATTATTAAAATTTTAATTATTCATTTTTAG**ATTAATTACGAACACAGCATACCTACATTAGTGGACAATGGCTTTGCATTGTGGGAA**
TCCCGAGCAATCCTTATCTATTTGGCAGATAAATACGGAAAAGAAGACACGCTGTACCCGAAAAATGCACAGAGGAAGGCTATTGTGAATCAGAGACTCTACTTCGATATGG
GGACCCTCTTTCAACGTCTCGCTGATTGTTACCTCAAACCCGTCATAGAAAAGAAACCTGTCGATCCGCAAGATTCGTGGAAAATGGAGGAGGCTGTTGGTTTTCTTAATAT
CGCTCTTGCGGGCCATAAGTATGCTGCTGGAGACACCATGACCATTGCAGACTTTGCTCTTGTTGCAACCATCTCAACGTATGAAGGACTCAAGTTTGATTTGACCCCCTAC
GCCAATGTGACCAAATGGTTTAACCGGTGCAAAAAGCATATGCGTGGGTATGAAATTAATCAAAGGGGTGTGGATGATTTTGTGAGAACAGTTGCGCCATTGCTACCAAAA
ACTGAATGAACATTATTTTATGTTTTTTTATGAACATTTTAGTGTTTTTCCCAAATTAATAAGAAAAATAATTGTAGGATCCTTCACTCTATATTAAGGATAAAGCTC
TAAGAACCTTTTCCGATTATGCTTACACAACTGTATTCATATCATTAATTTTGACCACCATCTGTACTTATGATGATAATAAATTGATATGAATACTCATACAGGTGT
TTGATATATTGGTGTACTCAAAAACAAACCATACTTAAAGCCCAGAGAGGCGAAAATTACTGTCAGTAATACAAAATTTGTCTTTTAATAAACATTAAAGTCATTTAT
AAATCCAACTAAACTTTCTTTGCTTTCTTATCTTATCGTTTACGTAAATGTATGTGTTAGAACAATCGCGCACTTTATAAATTAAATTCTAGAGCAGCGCGACCTGCC
GCGTGCCCGTTCACTTTGGAATCTCTGACCGATCTAACAAGTTTTTATCATTGACACAAAGTCATGATTTCCAGTCTATTTTTCCTTTGACATTTTGGAACTTTCCAG
TGACCACCTCTGGAATTCTTCAAGCCTTCAAATGGCGCCAAATTTGAATTTATTAAGACAATTTGCAATTATTTCAACAGTATGTATTTTCTTCAGAGTGATAAAAGA
TAAAACCGTGATGTAATACGTTTGAAAAGCATTATTTAGCTTAATTGTATTAGTGTTCATAATATTGATGAAAATGTCTGTTTTCCTATTTTTCTTTGAATTATAATA
AATTTTCTCTTGTTTTCTAGATAACTTCTAATCAGGAAATTGAAAAAAAGCTCTCAATATGCCGATTGATTTGTACTACTCACCTGTTTCCGCCCACTGCCGTAGCGT
TCTCCTCTTAAACCAAATTCTTGGAATTGAATTTAATCTCAAGGAAATGCGTAAGTTTTCATTGCTGGAAAATTGAGTACATAATAACTTGAATTTAAAAAAAATAAG
AGGTAGTAAAGAAATTAACGTCTTAATTTGTACTAAAAAGAATTAATAAACTTTCTTTTTTGCAG**ATAAAAGTGCCG**GTGATTTCACGAAACCAGAATTTCTTAAGATA
AGAAATGTTTAATCCGTGAGAATTTCTTTATCTTTCTATTTATAATTTTTTAATTGTAAATCAATGAATTTTTTTCATATTTTGTTAACAG**ATAAATTACCAACATATT**
GTACCCACAATCGTCGATGATGGCTTTACATTATGGGAATCCCGAGCAATACTTCAGTATTTGGTGGAAAAATACGGAAAGGATGATTCGCTTTACCCAAAGGATCCCCAGA
AGAGAGCTCTCGTAAACCAGAAGCTCTACTTCGACATGGGAACGCTTCATCAACGTCTTGCTGATTACTACTACAAACCAATTTTTACAAAAACTTCTTTTGATCCGGCTGT
CTTCAAGAAAATAGAAGAAGCAATGGGCTTCTTTAATACAGGGCTCGAGGCGCAGCTTTTTGCAGCTGGAGATTCCCTAACAATTGCAGATTTTTCCCTGATGGCTACTGTA
TCGACTTACGATGCTCTAAAATTTGATTTAACTCCGTATCCTAATGTTGCTCAATGGTTTGAGCGATGCAAAAAGACTATGCCAGGATATGAAATTAATCAGAAAGGTGTTG
AGAAATTTGTTGAGAGAATTTTGCCAATGCTTCCGAAATAATTTTTTCTTAACTTGTATATTTTTTAAAATAAAATTATGGACTTAAAACTGAGTTTGATACTTAAAT
TGTTTAAAAAAAAAGCTCCAATATTTTTGAACAGTAACTGTAGATAATACTACTACACTGAAATATTCTAATATATCACCAACATTACACCTCCCAATATCTAAGAGC
ACGGAAACTTCTCTCAGTCGATCAAATAATTTTGTGAACTCACTTTGGCTCCAGCCGGCGTTTAGATAAGTTGAAAATAAGGAATGTAAGAACCTTTTCCGATTTATG
CTTACAAAACTGCCTATTCATATCATTAATTTTGACCACCATCTGTACTTCTGATGATAATAAAATGATATGAATACTCATACAGATGTCTGATATATTGGTGTACTC
AAAAACAAACCACACTTAAGGCTCAGTAAGACGAAAATACTGTCAGTAATATAAAATTTGTCTTTTAATAAACATTAAAGTCATTTAGAAATCCCACAAAACTTTCTT
TGCTTTCTTATATTATCGTTTACGTAAATGTATGTATTTTTTTTCTGAGTGATAAAAGATAAAACCGATAAAACCGTGATGTAATACGTTTGAAAAGCATTATTTAGCT
TAATTGTATTAGTGTTAATAATAGTGATGAAAATGTCTGTTTTCCTATTTTTCTTTGAATTATAATAAATTTTCTCTTGTTTTCTAGATAACTTCTAATCAGGAAATT
GAAAAAAAGCTCTCAATATGCCGATTGATTTGTACTACTCACCTATTTCCGCCCACTGCCGTAGCGTTCTCCTCTTAAACCAAATTCTTGGAATTGAATTTAATCTCA
AGGAAATATATTCAGGTATTGGTTAGCGTCACATGGGATATACTCTTTCCACTCATTATTATAAATGGATGGGAAAAGTACATCCTACGCTAACCAATACCTAACTGT
ACGTAAGTTTTCATTGCTGGAAAATTGGAGTACATAACATAACAATTTAAATTTTTTCTTTTACACAAAGAAAATGATTTTATAAGGAGTACCTATTGAAATAATTTA
AATTAAAAAAAATTAAGAGGTAGTTAAGAAATTAACAACGTCTTAATTTGTACTAAAAAGTTTTAATAAACATTTTTTTGCAG**ATAATAGGCGGAATCAAATGTTGCAG**
AAAAAATCAGAATTTCGTAAGGTAAAAAATGTTTAATCTGTGAGAAATTCTATATCTTACTTATTTATAAATTTTTGATTGTAAATCAATATTTTTTTTCTTATTTTAA
AACAG**ATAAATAACCAAGAAATCTTACCCACAATCGTCGATGATGGCTTTACTTTGTGGGGATGCCGAGAAATCCTTCAATATTTGGTGGAAAAATACGGAAAGGATGATTC**
ACTTTACCCAAAGGATCCTCAGAAGAGAGCTCTTGTGAATCAGAAGCTCTACTTCGATATGGGAACGCTCCATCAACCTTTTGCTGACTACTACTTCAAACGAATTTTTGCA
AAAACTTCTATCGATCCGGCTGCCTTCAAGAAAATAGAAGAAGCAATGGGCTTCTTTAATACAGGCCTCGAGGGGCAGCTTTTTGCAGCTGGAGATGCCCTGACTATTGCAG
ACTTTTCTCTGATGGCTACTGTTTCGACTTACGATGCTTTTAAATTTGATTTAACCCCGTATCCCAATGTTGCGCAATGGTTTGAGAGGTCCAAGAAGACTATGCCAGGATA
TGAGGAGATTAATCAGAAAAGTTCTGAGATGGAGGAATATCACGAGAAAATTTTGTCAAGGCTTCAAAAATAATTTTTTCATATCCTCTATATTTTTTTAAATAAATATTG
GACTTAAAATACCCTAATTTGATACTTAAATTGTTTCAAAAAAAAAGCGCCAATATTTTTGAGCAGTATCTGTAGATAGTATGTACTACTACACTGAAAAATTCTAAT
ATATCACCAACTTAAACCTCCCATATAAATATCAGAGAGCACGGAAACGTTTCTCAGTCGAACAAATCATTTTGTGAACACACTTTGGTTCCAGCCGGCGTTAAGGTA
AGTTGAAAATTCTCCGTATCTCTGTAGGTGCAATTAACGTGTTTCTCAGTGATAAAAAGTGCTAATTGAAGGTGGAAAAAATTGTGTAATAATGACTTGCTTCTTTGA
ATAATTTTTTTAATTAAAGTTTTTTACATTTAAACGGTGATTGGTTTCTAAACGTCTAACAAATACTTTCAGAATAAAATTCAACACAAATTTCCATAGAAGAGCGTT
AATACAACCATACAGCAGCAGAATGACGATTGATTTTTACTACACTGATATTTCTGCGCCTTGCCGCAGCATTCTTCTTTTGGGAAAAGCTCTTGGGATTGAATTCAA
TCTCAAAGAGATGGGTAAGCTGTTTTTGTTATTTTATTTTAGAGATTTTCCTTATTTAAACGTTTGAATATTTGAAAACAAATTATTAATTATTCAATATTGTACATT
TTTCACGCAGACTTAATGGCCGGGGATCATTTGAAACCAGAATTTGTGAAGGTAAGAAAAGCTCTCTATTTTATTTCTTATTAAAAATTTTACAATACTCGAAGCAAT
AACAAAAAAATTCTAAATTTAGATCAAATTAAGTAATTTTTATCAGTTACCAAATGTCGTGACTCAAGAGTTTGCTAAATGATAAGATTACAGAGTTTTCAATTGAAT
GCTTTTAAAAATATTCAAACAGAAAACTCTATATGAACTCTAATACAAAATAGTTAATTTTATAATAATTTCAAGAATTATTCTTAATGATTTTTAAATTAATTTAAT
TTATCAGATTAACTACCAACACTGTGTGCCCACCATTGTGGACAATGGCTTTGCATTGTGGGAATCACGAGCAATTCTCATCTACTTGGTTGACAAATACGCAAAGAA
CGATTCTCTGTACCCATCTGATCCTCAGAAGAGGGCCCGTGTGAATCAGAAGCTCTACTTCGACATGGGTACACTCTATCAGCGCTTTGCTGAATACTATTATCCACC
AATTTTTGCAAAAGCTCACTTTGATCCGGCCGCTTTCAAGAAAATGGAAGATGCTGTTGTATTCCTCAATACTGGTCTCGAGGGTCAAAAGTATGCTGCTGGTGATTC
CCTGACCATTGCTGATTTCGCTCTCGTAGCCACTATTGCTACATTTGATGCGTTTAAATTTGATTTGTCCAAGTACCCGAATGTTGTGCAATGGTATGAGACGTGCAA
GAAGACCATCACTGGCTATGAGGTCAATCAAAAGGGTGCTGATGGATTTATGGCAAAATACGGAGCTGCCTTGGATAAGCAGAAGGCCTAAAGCAGTCTAAATTTTTT
AAGGAATCAATGCAATTACTTTGTGAATTTCTTTAAAATCTTTGAAAATCTTTTGTAAAAATGTCTCGAAGTTATTCAAACGATTCAATGAGAATAAAAATTATTTTG
TAATGTAAAAAAGCTTTTTTTCTTATACCTCCTTCTCTTATTGATAAGATATTAAAACATTTGCTGGATCGCATATAGTTCCCTTAATAGCGTCAGATATCTTAACAC
GGAACGCTCTCGCATTAACATCAGTTAAATTGAGAGGTGTTTGGCTATGAAATATTATAGGGAGGTGATCAGAAAGGAACCAAAGGTAACCGAAGGGATCAATAAACA
TATCGGAAATGAATGGAATTGCATCTGATTCAAAAATCACCCCGATTGTATCAGGATTGAGAAGAAGTTCAGAGTTCCAACAGGAAATCCTTTTGGATTGGATACTTC
CGTAAAATACTACGCCTGTAGTATAATCAAATACACGACGTAGTGCTTGAGAATCACAACCTCTGTAGGAGAATATTGAGAAATCATCTGGTTGGTAGTTGGTAGGAG
CTTTCTTTGCATTTTTCAGAGTTTTCGTAGTAGTCACATATTCTGCCGTGCTAGAACCGGGAGCGTAGTAAACAATTCTATTGCCATATTTGTCTGGCCAGCCAAGGG
TCATGCTTGAAATTCCAATTTCCATAAACCACGGAATAGTAGAGTCAAAAATAAAGTTTGATTCTGAAATTATGGGGTTCATTNNNNNNNNNNNNNNNNNNNNNNNNN
NNNNNNNNNNNNNNNNNNNNNNNNNCAAGTGAGGAGTTCGGTTAGTAGCTACAATTAATCTCTCAGATGGAAAATCTTTGGAAATGTCGGAAAATTCCACCAACTTAA
TTACCCCCACAGGTGACAATATTAGTCGACGGATTCTTAAAAAAGTCAGAAGCGGAAGGGTTCAGGAAAAGCTCGAAAACTCATATTTCCCGTGGAGATAGAGAAAAG
CGGTCTGAGACAAAGTTGTAGGCAAGGAAATTTCCTATGAGAATGACCTATCGAGGAAAATTTCTTGCCCTGTAGGAGTCCTGTAGATAGGGATTTATCCAAATTGAC
ACAAATAGCCCCGCCCTCCCCTATTTTATTTATTTTGCTTTATTAAGAATGTTGGAGTAAAAATCATCACCAACAGCTCGTCTGCATGATTTTATTACCTTAAGATTG
GGTTAGCAATTTCAGGCAGCACCTGTTGAGATTGATATCTCTGCTACTCATACAAATATCAAACTTTGTATCTACCAAATCATTATCGTCTGACCTACGAAAAAGCAT

ACCATATAAGTAGATGAATGTCCAAAAAATTTTCAGTCACAGATTTGTGTTTTATTGTGAGACATCTCATAGACTTTTTGAGGTGTTTTTCGGATAAGTAAAGACGTG
AGAGAATTGACAGATTGACCAAAAAAGGTTTGGGAGCTTTTTCTTATCTGAAAAGAATCGAAGATTAAATGCAGTTTTGAACAAAATAAAAAAAAACTTCTCCTCGTG
TCGTGTGCATGATTTCAAATTAATGTTCAACAGTGATTATCATTACAGAAGTCGTCATAATTTTTGGTGGGTGATCAAATATCTAATCTCTTGTTTTACAGAGCTTTG
GTTGAAAGAGAGTATAAAGTGAACAATACACAATCAAAATGACTCTTGATTTTTACTATTCTCCACTTTCTGCGCCATGCCGAAGCATTCTTCTTCTTTGCAAAAGCC
TTGGAATTGAATTAAACCTCAAGGAAATAGGTAAATGAAAATGCAACACTTATTTCTTCTTTTAAATAAAAAATAGCTGAAATTTAATTTTTTTACAGACATTTTGGG
TGNNNNNNNNNNNNNNNNNNNNNNNNNNNNNNNNNNNNNNNNNNNNNNNNNNCTCATCATACGATCCGGAAGCATTTAAGAGG***ATGGAACAGGGTGTTGAATTTCTCAA***
TACAGCCCTCGAGGGGAAGACCTACGTTGCTGGAGATCATTTGACTATCGCTGATCTTGCATTAGTCGCTACCATTTCAACATACGATGGCCTCAAATTTGACTTCTCGAAG
TATCCCAATGTGACCAAATGGTATGAGACGTGCAAGAAGATGCCAGGCTATGAAGTCAACCAGAAGGGTGTGGATAAATTCGTTGAAATAATGGAATCGAAGAAATAATAT
TTTTTGTTGCGAAAGATTTAAAATTTTTAAACAGTCTTGCATTATATTCAAAACATTTTTCATTCTATGATTTCCTGAAAATCTTGATATAAAATCAATCTGTTTGAC
ATGGATTTTAATTAAAATAAAATAGAAGACATTTTAAAGATCTTTTGAATTTGAAGAAAATCTGAATATCTTTTTGGTCGATCTAATATAGCTGGACTGGACATAAAC
TGGATTCATCGACTTTTATCAGAACCTGTAAATTTAGATTATAAAAAATATATAATTACTCACACTATCAAATATATTAATCTGGATGTTTGAAATGCACAAAACAAT
CAATTCAGAATGCGAAGATCCGTCAAATTTGTCAGTTAGTTGAAAGTTGTATTGAGGAATCATCCCTGACTTCTCTCTTTTTGTGCAGAAGCTGCAGAGCTTAGGTAT
GTATTTTTTTTAATTTAAAATGTTCTAAAAATATTTTGTTCTTTTTACAATATTATTTTCCTTTTTCCTTTATAGTGAACCATTTTCCAAGAATTTCCAATCAGAAGA
AAAACTAAGAAGAGTTTCGAAGATGACGATTGATTTATACTACTCACCAGCTTCCTCGCCCTGTCGTAGCATTCTTCTTCTGGGAAAAACTCTTGGAATTCATTTTAA
CCTCATTGAAACGCGTAAGGAAATTACATTTCTTTTTCTATTATAACAATGTAAAAAAAAGTGTAAAATAATTTATTTGAAATTTCTTCTTAGGGTTATGTAGAGGGG
ATAATTTCAAGCCAGAATTCTCAAGGGTAAAATAATAAATTCTCTATTTTCATTATTATTTTTTGAATTAATTAAATATGTAATTTTTTGGCAG***ATTAATTACGAGCAT***
AGCATACCTACATTGGTGGACAATGGCTTTGCATTGTGGGAATCCCGAGCAATTCTCATCTATTTGGCAGATAAATACGGAAAAGAAGACACGCTGTACCCGAAAAATGCAC
AGAGGAAGGCTATTGTGAATCAGAGACTCTACTTCGATATGGGAACCCTCTTTCAACGTCTCGCTGATTGTTACCTCAAACCCGTGATTGAAAAGAAACCTGTCGATCCGCA
AGATTTGTGGAAAATGGAGGAGGCGGTTGGCTTCCTTAATATCGCTCTTGCGGGTCATAAGTATGCTGCTGGAGACACCATGACCATTGCAGACTTTGCTCTTGTTGCAACC
ATCTCAACGTATGAAGGACTCAAATTTGATTTGACCCNNNNNNNNNNNNNNNNNNNNNNNNNNNNNNNNNNNNNNNNNNNNNNNNNTTTGCCAATGCTTCCAAAATAA
TTTTTTTCTTAACTTGTATATTTTTAAAAATAAAATTATGGACTTAAAAACTGAGTTTGATACTTAATTTGTTAAAAAAAAGACTCCAATATTTTTGAGCAGTATCTG
TAGATAGTACTACTACACTGAAAAATTCTAATATATCACCAACTTAAACCTCCCATATAAATATCAGAGAGCACGGAAACAGTTCTCAGTCGAACAGATCATTTTGTG
AACTCACTTTGGCTCCAGCCGGCGTTTAGGTAAGTTGAAAATTCTCCGTAGCTCTGTAG***GTGCAATTAA***CGTGTTTCTCAGTGATAAAATGTGCTAATTGAAGGTGGAA
AATATTGTGTAATAATGACTTGCTTCTTTGAATATTTTTTTTAATTAGAGTTTTTTGCATTTAAGCAGTGATTGGTTTCTAAACATCCAACAAATACTTTCAGATTCA
AATTCAAG

>LLOJ004462 (212 aa; 3 exons) | UNCHANGED
MDLYYLPGSAPCRSVLLCGKALGLDFNLKTLDLMAGEHLKPEFLKINYQHCIPTLVDNGFALWESRAILAYLVEKYGKDDSLYPKDPQKRYFVNQKLYFDMGTLYQRF
ADYYYPVLFAKAPYNPEAYKKMEEAVGFLNTALEGKTYAAGDNLTIADLALVATISTYDGFKFDFSKYPNVAKWYETCKKIPGYEVNQKGVDEFVEKFGPLLPK-

>LLOJ004461_a (215 aa; 3 exons) | EDITED
MTIDFYYSALSAPCRSILLLAKTLGIELNLKPMDLMAGDQLTPEFIKLNYQHTIPTLVDNGFSIWESRAILAYLAEKYGKNDSLYPKDPEKKARVNQKLYFDMGVLYQ
RFGDYYYKPIQTKTPYDPAAYKKMEEAVGFLNTGLEGQKYIVGDTLTVADFAIIATISTYDVLKFDFTPYPNVVQWYERCKKTMPGYEINQKGADEFLEKIGPMLPK-

>LLOJ004461_b (217 aa; 3 exons) | EDITED
MTLDFYYSPLSAPCRSILLLCKTLGIELNLKEIDILGGEHLKPEFLKINYQHTIPTLVDNGFTMWESRSILAYLVEKYGKDDSLYPTDPQKRALVNQKLYFDMGVLYQ
RFADYYYKPIQTKTPLDPAAYKKIEEGLGFLNTGLEGQKFAAEDSLTIADFALITTISTYDGLKFNLTPYPNVAQWYERCKKTMPGYDLNVKGVEQFLEKIGPMLPKF
P-

>LLOJ004461_c (213 aa; 3 exons) | EDITED
MTLDFYYSPLSAPCRSILLLCKNLGIELNLKEIDILGGEHLKPEFLKINYQHTIPTLVDNGFAMWESRAILAYLVEKYGKDDSLYPKDPQKRYFVNQKLYFDMGTLYQ
RFADYHYPVIFKKTSSYDPEAFKRMEQGVEFLNTALEGKTYVVGDNLTIADLALVATISTYDGLKFDFSKYPNVAKWYETCKKMPGYEVNQKGVDKFVEIMESKK-

>LLOJ004461_d (217 aa; 3 exons) | EDITED
MTIDLYYSPASSPCRSILLLGKTLGIHFNLIETRLCRGDNFRPEFSRINYEHSIPTLVDNGFALWESRAILIYLADKYGKEDTLYPKNAQRKAIVNQRLYFDMGTLFQ
RLADCYLKPVIEKKPVDPQDSWKMEEAVGFLNIALAGHKYAAGDTMTIADFALVATISTYEGLKFDLTPYANVTKWFNRCKKHMRGYEINQRGVDDFVRTVAPLLPKT
E-

>LLOJ004461_e (218 aa; 3 exons) | EDITED
MPIDLYYSPVSAHCRSVLLLNQILGIEFNLKEMHKSAGDFTKPEFLKIRNINYQHIVPTIVDDGFTLWESRAILQYLVEKYGKDDSLYPKDPQKRALVNQKLYFDMGT
LHQRLADYYYKPIFTKTSFDPAVFKKIEEAMGFFNTGLEAQLFAAGDSLTIADFSLMATVSTYDALKFDLTPYPNVAQWFERCKKTMPGYEINQKGVEKFVERILPML
PK-

>LLOJ004461_f (222 aa; 3 exons) | EDITED
MPIDLYYSPISAHCRSVLLLNQILGIEFNLKEIYSDNRRNQMLQKKSEFRKINNQEILPTIVDDGFTLWGCREILQYLVEKYGKDDSLYPKDPQKRALVNQKLYFDMG
TLHQPFADYYFKRIFAKTSIDPAAFKKIEEAMGFFNTGLEGQLFAAGDALTIADFSLMATVSTYDAFKFDLTPYPNVAQWFERSKKTMPGYEEINQKSSEMEEYHEKI
LSRLQK-

>LLOJ004461_g (218 aa; 3 exons) | EDITED
MTIDFYYTDISAPCRSILLLGKALGIEFNLKEMDLMAGDHLKPEFVKINYQHCVPTIVDNGFALWESRAILIYLVDKYAKNDSLYPSDPQKRARVNQKLYFDMGTLYQ
RFAEYYYPPIFAKAHFDPAAFKKMEDAVVFLNTGLEGQKYAAGDSLTIADFALVATIATFDAFKFDLSKYPNVVQWYETCKKTITGYEVNQKGADGFMAKYGAALDKQ
KA-

>LLOJ004460_a (>128 aa; ~3 exons) | EDITED TO ADD 2 AND EXTEND 1 EXONS (PARTIAL GENE - INTERNAL GAP)
MTLDFYYSPLSAPCRSILLLCKSLGIELNLKEIAFWVXXXXXXXXXXXXXXXXXXXXXXXXXXXXXXXXXXXXXXXXXXXXXXXXXXXXXXXXXXXXXXXXXXXXXX
XXXXXXXXXXXXSSYDPEAFKRMEQGVEFLNTALEGKTYVAGDHLTIADLALVATISTYDGLKFDFSKYPNVTKWYETCKKMPGYEVNQKGVDKFVEIMESKK-

>LLOJ004460_b (>176 aa; ~3 exons) | EDITED TO ADD 2 AND EXTEND 1 EXONS (PARTIAL GENE - C-TERMINAL GAP)

MTIDLYYSPASSPCRSILLLGKTLGIHFNLIETRLCRGDNFKPEFSRINYEHSIPTLVDNGFALWESRAILIYLADKYGKEDTLYPKNAQRKAIVNQRLYFDMGTLFQ
RLADCYLKPVIEKKPVDPQDLWKMEEAVGFLNIALAGHKYAAGDTMTIADFALVATISTYEGLKFDLTXXXXXXXXXXXXXXXXXXXXXXXXXXXXXXXXXXXX

########################
####### GST iota #######
########################

####### PPAI009870 - GSTI #######

>JH661013.1 reverse | **PPAI009870** (pre-editing) | PPAI009870 (post-editing)

TGGATATTGTATGCTTAAGATATATAGTTAAATGTTGCATTCTTGCGATTGTGAAGGCAACATTCTAGGCGTTGTCGCTTTCCGTGACGAAAATTGCGCAACTTTTAG
CGGTAATTTTCCCATGGAAAAATCCCTGTTCTACTAAAGATCACTCGTTGCTGGGTCAAGTACTAACCTCAGGACGGTTTTTCGATCTAGAT**ATGAAGCTCTACGGTGT**
ATCAGATGGACCACCATCTTTGGCTGTCCGGATGGCTCTGAAGGCTCTGGACATTCCCTTTGAACTTGTGAATGTTGATTATTGTGCCGGAGAACATCTAACGGAGAAATAT
GCTGAAATTAATCCGCAAAAGGAGATTCCAGTTCTGGATGATGATGGGTTTTATCTGTCCGAAAGCATTGCTATTTTGCAGTATCTCTGCGATAAATATCGTCCGGATTCTC
AACTCTATCCCAAGGATCCTAAGGCAAGGGCCATCGTTAACCATCGACTCAATTTCAACTCGGCATTTTACTACAGCAGCATTTCCATGTATGTGGTAAGTATAAAAAGTT
TTGATAAATTGGAAGATAAATATGTTTTTTATTTGCAAGAATTAGCAAATATTCTCACATGGCTACAAGGCTTTCCATTTTTTTTTATAATTTGAATATTGTTGAAAA
ATCATTGAATCTTTGCGTACTCGTAGAACGAATATATATTCCGTTGATGCAAGAAGCGACGGGACGTTTGCATCACTGTTTGCACCAAATGCAGATACGACAACGGTC
GATGCACCCGACGATATCTTTGAAAATCAGGCTATTTTCTGTTAATTTAAGGCCTAGGCACACTTACGACTTAAGCCGAGGGACGACTTAGTGGAAAATTATAGAAAT
GTATTTTAATCATTATTTCAAATATAATTACACTGACTGAGAGAAATCCGAAAAAGTTAAAATAACATTCCGAAAATGTTAATTTTACCCTGCAGTATTGATCCGAAA
TCGGTGGAAATATTACCCTTTTTAGGTGTATTAGGGGTAAAATGTACCCTTTTCCATGTTGATTTGACCCTTAATAAGGTGTAAAATTAACATTAAAAAATGTTGATA
TATTTTTACACCTAGAAAGTGTTAAATTTATGAGGAAAAAATGTTAATCGCACCCTCTTTTTTCTCAGTGTACGCTAAGTCGTCTCTCGGCTAATCCTCAAGTCTGTC
GAGGCCCTTACCTCCTCATCGTCCTCAGACCTAGAAACCAAAAACCAAGCCGAAGATAGCTAGGTACTTTGCATGATTTCAGAATTATCTCGAAAACGTCGCACAATC
TTTTGTATAACTTTTAGAACTTGTCTAGGCGGAACCGTTTATTATACAAAAATATTGTTTTCAATCATTTCTTATAAGAGCTTTTTCAAGGTCAAAAGTTAAAGTGGC
TCTAGGGAGCATATTTTTCAACCGAATTAATAATGTTCTGATTCGTTGGAAAGGTTTTGGAATCCCCTTACAAATCTGAACTGGTTCCAATAGAATATTAATCGATAA
TAAACCTTTAATTTTTAGTCGAAAAAGTTGAAACGCTTTAAAATTTTATTATGCTATTTAAATATAAAACTGACAAAACTAGGAGCATAGTATTTAATGTTCGGATAA
AATTTGGAAAGGCAGACGCTTAACCCATTCCTTACCATGGTATTATACATCGTACGCAAATTGAGTTATTTTAGATGATTTATTTCTGGGCAATTGATATTCGAATTG
CTGTCGGGAATGGATTTTTAATTACCTTAGTTCTTCAATTACTTGGGAAAAAATAGTCCGACAGAAATGGGAATACATTTTGTTGTTCTTTTCTTGCTTCACGTGAAGT
CATGTAAAAATTTTGCTCAAAATGGCGGACGGAAAGTTCGACATCCCGTCGAATTTGTTAAAATTTGCCTTCATCTTCTCTGATTTGGTCATGAATTGGTCTTAAGTG
TAGCTAAAAGACCTTATTTCTGGCATAAAGAATAAGAGATCAAGTCCACAATATTAAAATAATTCTCATTGATCTGATTTGTTTCACTTTTATTTAAAAAATCGGGAT
CGTCCCTATTTGAATTTCGCTCAAAAATCACCTAAAAATTTGATATTTGGCTGGCGTTCACGCACGAACGCAATTGCGCGTGGGTCACTACCATAAAAAGTCTTGTTG
GATAGGCCTATATTATACAAAAAAATATTAGGTCGATCCGACTTACAGACCACCCACACCACACACTTATAAGTCCGTCCCTATTTGAATTTCGCTCACTGTATTTCC
AGAAGATGACCCTACTAAAATTCTTATATACGCGGACGATATCGTCATCTATAATTCCACTCCTGATAGTGTTGAACAATATATAGAACTTCAAAAGCGTCTAAACGA
AATGGGTAGATGGCCGTCTGACAACGGATTTAAGTTTTCACCTCGGTTTTCACCCTCCAAAACGATGGCAATGCACTTTTGTCGAAAACATAGGTGTCCACATCAAAC
ATTTACTCTCAACGGTTCCTGCCTCAAACACACAGATTCATACAAATTCTTAGGCATCTTTCTTGACCCTAAGCTATCCTTTAAAACACATATTCAGAACTTAAAAGT
CTCGTGTAATAAAAGACTAAATGTGTTAAAGTGTCTCTCAGGACTAACCTGGGGATCCGATAGAAGCTCACTTGTGAAAGTTCACGAAGCCACTGTTCTATCTAAGAT
GTTCTATGGGTGCGAAGTATATGCAACAGCTGGATCTCAAACTTATTTAAATACTCTCAACAGTATTTACAATACAGGATACAGGTTTAGTTTAGGCGCGTTTGTAAC
TTCCCCTACAAATAGTATTCTTTCAGAAGCAGGAGTTCCTCCACTCCGGCAGCGATTTGACTTCATATGTGCTAAGACTGCAATCCGGATGGCCTCATCCTACGGGAT
TACAGGGCATACCGATATAATTGCATATGAAGAAAGAAAAAGTCGCAAAAGCTTCTTCTCAAGTGGTGGCAGTTTGGCTTACTCAATTATGGATGGACTTCAATTTGA
GGAAAATAAAATACAAGGTCTCCCTCCATGGCTCTTTAAATAAAACTCCATTAATACTTCACTAACTACCACTTCCTACAAGGACATGTTGCCTGATGAAATTTCCGT
ATATTTTAATAGAATCTGCAATAATATATCACCTTCTTCTACAGTCCTTTACACTGATGGCTCCGCCAGTGGGGAAAGTAGAGGATTCGCAGTCGTCTTTGATCATGA
ACTTATATAGTGCCAAATGCCATAGTAGTTTATCAGTTTATGATTTGGAAGCCAGGGGTATTGAAAAGGCGCTAGAAGCAATCCAAAGTCCCGACGTTGCCACCCAAC
ACTATATTACTGCATCCGATTCCCTGAGTGTCATTACTGCTGTGGACAACTTAGCTATCAATTCCCCCATCATTCGAAGAATCAGAAACCTACTGATTGAATTAGGAG
ATCAGACCACACTACTTTGGATTCCGGGTCACAAGGGCATTCCAGGGAATGAGAGAGCAGACAGAGAATCCAAAGCAGCTTCCTTTTTACCTTCCATCACTGATACTT
GTGTCCCCGTCAAATCTGCAATTTCATTGTGCAAAAACTTTTACGACGACTTAAAACTAAAATGGTTCAATTATGAGGTAACTGGGTTCATAAAAAAAACAATTTACG
TACCAAAACGTCCTATTATCCCTAAATCACTTACCAGAAAAGAATGCACCATAATTTCAAGATTAAGAATTGGACACTGTCGATTAACCCAGTCCTTCAAATTAACTA
AATCTCCCATACCCCTCTGTCCATCCTGTAAAAGATATCTTACCATATAGAACACATCTTACTCGAATGTAAAAAACTAAGTAAAATAAGGAAACAAATTTTTGGAAA
TAAAAACATATTAAGCTTGCTGAGTTATTCAAAGTCAGATATAGATTTAATAATAAAATTCATTAAAGAATTAGAACTTTTGGAAGAAATTTAAACGTTTCTGTAATC
TATTTTTTCTTACTCTTTCTTTTCATTTTACAAGTATTTCTTTTTCTTCTTTTCATTGAACTTATCCTCCAATTGTAATATACATTAACCACTAATATAAAACTATGT
AAAATTATACGGTGCTATATGAAAAAATGCACCTAATGAAATTTGAATAAATAAATAATAATAATAATTTTACAACTTTCCCCTGGGAATTTTAAGTCGGAATGCAA
TTTTATTCACTAAATTTGTGTCCTATCACGGAAAACGGTCGTTGCATTAAACTTTCATACACGATAAAAGTGCACGAGGCTCTTTAAATCTGACTGAATTGGTGAAGA
TAAATAATAATTATTCATTCGAGAAAAATATTTGAGAAAGTCTTAAAGAAAAATGGAAAAGAAATGACAGTTCTTCATTCGATAGTTATAATATTGGTATTACAGAGT
GATTATATTTTATTCAGTCATAATCGATACATTTTACCGAACATAATGCCGTGATTATATCTTAAAATAAATATTTGTTATATTTCGGATGTAAAAATTCCCCATTCA
CTCAAATATGGACCTCTTTTATGCGGTTATATAGAAGAAATTTGTAAAGTTTTCGCCATCGTTTTCGTATAGATGTCAGACATCTCTACTCATTTATAGCTCTAAATG
CTCCTTGGGAGAGAAATCCGAAAAAGTTAAAATAATAGTTATAAAGGTGTAAAATTAACAGTAAAAAATGTTGATATATTTTTACACCTTAAAAGTGTTAAAGTTATG
AGGAAAAAAAGTTAATCGCACCCTCTTTTTTTCTCTATGTTCCCCTAATCTATTTTAACAAAATTTTGTTCTGAGCTAGAAAAAAATCTTCAGAATGGCTAGGCTGTT
TCAGGCAAGGAGAAATTTCAATATAAAATTTCAAATGCGACTTGAAAAAAAGTTTTAGCTGCCTGACACTGCCCTATCCTCCCCTACATTGTATTTTTTCCATCTTAT
TTAGGAAAATTATTTAATTTTTTTCTATATAGGGTAAGTGCGCCANNNNNNNNNNNNNNNNNNNNNNNNNNNNNNNNNNNNNNNNNNNNNNNNNNNNNNNNNNNNNNNNN
NNNNNNNNNNNNNNNNNNNNNNNNNNNNNNNNNNNNNNNNNNNNNNNNNNNNNNNNNNNNNNNNNNNNNNNNNNNNNNNNNNNNNNNNNNNNNNNNNNNNNNNNNNNNNN
NNNNNNNNNNNNNNNNNNNNNNNNNNNNNNNNNNNNNNNNNNNNNNNNNNNNNNNNNNNNNNNNNNNNNNNNNNNNNNNNNNNNNNNNNNNNNNNNNNNNNNNNNNNNNN
NNNNNNNNNNNNNNNNNNNNNNNNNNNNNNNNNNNNNNNNNNNNNNNNNNNNNNNNNNNNNNNNNNNNNNNNNNNNNNNNNNNNNNNNNNNNNNNNNNNNNNNNNNNNNN
NNNNNNNNNNNNNNNNNNNNNNNNNNNNNNNNNNNNNNNNNNNNNNNNNNNNNNNNNNNNNNNNNNNNNNNNNNNNNNNNNNNNNNNNNNNNNNNNNNNNNNNNNNNNNN
NNNNNNNNAGTCTACTGTAATTCAACCATAGGTACTGAGGACACCCAAATATTAACAAATTAAATAGAAAGAGCACAGCTCTTAAATTTAAGTGTGAAGTAAGGTCAC
AGCCAATAAATACATTTCTATGGGGAGTGATATGGTCCCGGCGATTTAAGCCACAGATATACCTGACACAATTATTGAAAGAAACTGTTAGACGACGGAAGGTGTCAC
TATCCGCCGAAAAGAAGAGTTCTGCTCCGTAGTTAAAGAAAGGAAGAAGAAGCGCTCTCGCGAGGAGCAAGCGAGTTATTCAGTCATAATCGATACATTTTACCGTGA
TATATAATAATGCGTCATAATGCCGTGATTATATCTTAAAATAAATATTTGTTATATTTCGGATGTAAAAATTCCCCATTCACTCAAATATGGACCTCTTTTATGCGG
TTATATAGAAGAAATTTGTAAATTTTTCGCCATCGTTTTCGTAAAGATGTCAGACATCTCTACTCATTTATGGCGATAAATGTTCCTTGGGTGAGAAGTGCAGGAAGT
GTTGGAAGTTTTGCAACATTTTCGACAGTGTTGCGTATTTTCGGAAGTGTGAAGGTATCTTCCATACAAATTGTGGAAGTGCCTGGAAGTGGTGCACACATTTTCACC
CAAATATCTCCCCCTTGATTCAAATTTATTTCATTTAAAATTATAAATTGTTTATGCTTATGATTTCAATGGCAATAGTCTTTATGCTTTGCATCGTATTACGTGATT
ATTCTTAATAAGAACGTGTGAAATGATTTCTGCAAATTTATGCAGCTACTGGAAAGGCACAAATAATTCATTAGACAGGTTTTCCTTAAGAACCACAATTATTCTGAT
GCAATAAGAAGATTGTCAATAAGTTCTTCAGAATTTTCAATCTCATGTGGGATTTTTTTTGGCAG**ATGGCTCCCATTTTCTTCGACTACCAACGCACTCCAATTGGACTG**
AAGAAGCTGAATATGTCCTTGGAGGTCTTTGAAACTTATATGAAACGCTCTGGAACAAAATATGCAGCTGCAGATTATCTGACGATTGCTGACTTCCCTCTCGTAACAGCCA
CTTTGTGCTTGGAAGCTATCAACTTCAGCCTGGATGAATATCCACTAGTGAAGGCTTGGTATGCTAATTTCAAGAAGGAATATCCCGATTTGTGGGCAATTGGAGAGGGTGG

AATGAAGGAAATTGCTGAGTTCGAGAAGAACCCACCAGATCTCAGCCGCATGGTCCATCCCATTCATCCAATGCGAAAGAATTGAGGATTCAAGGGGTTTTCAAAATAAAA
TACTCTGTGTCATTTTTGATGTATCTTTCTTTGCAAAGAAAACTGTTGCACAACATTTACTTCTTCTGTCTTTGCTCTTAATTTGTTTTAAGCACATTCAGCATCCAT
GAATTACACCCTAATGATTCCCATAGGTTTTTAGTCTCACATCTATCCCACAAACTTCGCGCCCAT

>PPAI009870 (229 aa; 2 exons) | UNCHANGED
MKLYGVSDGPPSLAVRMALKALDIPFELVNVDYCAGEHLTEKYAEINPQKEIPVLDDDGFYLSESIAILQYLCDKYRPDSQLYPKDPKARAIVNHRLNFNSAFYYSSI
SMYVMAPIFFDYQRTPIGLKKLNMSLEVFETYMKRSGTKYAAADYLTIADFPLVTATLCLEAINFSLDEYPLVKAWYANFKKEYPDLWAIGEGGMKEIAEFEKNPPDL
SRMVHPIHPMRKN-

####### LLOJ002711 - GSTI #######

>JH689493 reverse | **LLOJ002711** (pre-editing) | LLOJ002711_a, LLOJ002711_b, LLOJ002711_c (post-editing)
ACTAAAAAGCCCAATTAACCTTTAGAACAAAACAAAGAAAAATTCTTTACGACTTAATTTAATTTTTTTTTCAACTATTTCTCATGAAGAAGACCCATAAAACAACTT
TATTTTTAAAATAATTTTTAAACGAATTCATTTTTTTAAATACATTGTCTTGTTTTTTTTAAAGAAAATATATTTTATTTGAATGCAGAACA**ATGAAGCTGTACGCTGT**
GTCTGATGGACCCCCCTCATTGGCCGTCCGAATGGCCCTAAAAGCTTTGGACATTCCGTATGAGCACATCAACGTGGATTATTGTGCTTCAGAGCACATGACGGAGAAGTAC
GCCGAGATGAATCCGCAGAAGGAGATCCCCGTTCTCGATGACGACGGATTCTTCCTTCCCGAAAGCATTGCCATTCTGCAGTATCTTTGCGATAAATACCGCCCGGATTCTG
AGCTCTACCCAAAAGACCCAAAAGCTCGCGCCATTGTCAATCATCGTCTCAATTTTAATTCTTCCTTCTACTACAGCAGCATTTCTATGTATGTGGTGAGTATTTCATTTT
ATTTTTCTTTTGTTATTTAATTTTTTTAAGAAAGTTTTTAATTTTTTTGAAAATTAAAAATTGGAATTGAATTTACTAAAAGAAATTGTCTATGAAATATTTGTCAAA
GTTTTATGTGGAAATTCTTTAGATTTTCAATGGGATTTACGGTATTTGTGATATTTGCAGTATTTGTAAAAATAGGATTTGTCCTTAATAGCTTTCAAATAATTTTAA
AATAATATAAATGTTTCTAAATCCTTAAAATACTTGATGTTTGATACCAACAATGAACTAATAATATTATTCTTTTTACTCTTTCAATTACCGCAAATACCAGAAATA
CAATAAGTCCCATCAGTTTTTTTAATAG**AGCACAAATACAAGAACATTCTACAAGAGTTTTTA**NNNNNNNNNNNNNNNNNNNNNNNNNNNNNNNNNNNNNNNNNNNNNN
NNNN**CTCTGGACATTCCGTATGAGCACATCAACGTGGATTACTGTGCTTCAGAGCACATGACGGAGAAGTACGCCGAGATGAATCCGCAGAAGGAGATCCCCGTTCTCGATG**
ACGACGGATTCTTCCTTCCCGAAAGCATTGCCATTCTGCAGTATCTTTGCGATAAATATCGCCCGGATTCTGAGCTCTACCCAAAAGACCCAAAAGCTCGCGCTATTGTCAA
TCATCGTCTCAACTTTAATTCTTCCTTCTACTACAGCAGCATTTCTATGTATGTGGTGAGTATTTGTTTTATTTTTCTTTGTTCCTTAAATTTTTTTAAGAAAGTTTTTA
AAATTATAAATTTAGAATTAGAATTGAATTTATTAAGAGAAATTGACAAAGAAATATTTTTCGTATTTCTCAAAGTTTCATGTAGAAGTTCTTAAGATTTTTGGTGGA
ATTTACGGTATTTGTGGTATTTGCAGTATTTGTAAAAGTATGATTTGTCTTTAATACCTTCCAAATAATTTTAAAATGATAAAAATATTTTTAAATTCTTCAAATACT
TGGTATTTCATACCAACAATGCCAACAATGAACTAATAATTATTTCTTTCCCATTCAATTACCTCAAATACCAGAAATACCATAAGTCCTTTTAATAGTGCACAAATA
CAAGAACATTCTACAAGAGTTTTTAGACTTCTCAAATAATAAAGAAGGCAAAAAAAAATTCTAAAATCCTTCGAATACCTTTAAATAGTTTTTTTTGTTTAATCTTTC
TCTTCCTCTTCTTCAAATTAATACAAATCTCGTAAGCTTTTGATCACCGACTACTCGCACCTGTTGCATTCCAAACGCCTCCAATAAATTTTAAAATTTGCATTTCTC
ACAGCAGAAAGATTTATGAGAGGATGGCTAGAAAATTGAAAGTAGTTCTCCTTCCTTTGCGCTGATTAATCAGTGATTAACCCTTTCGCGTCTATATTTCTCAATCAG
AACTTCTTCTTTGGCATATTCTGCGTAAAAGTAACGTACCTATTTTCAATTTAAGAAAAAAATTAAGTCAAGTTTTTGGGAACCAAATTAAGTTAACCCTTTAAGGTT
TTTTGGGTCATACGCTGACCCAAACGTGAAACATTTTATTTTTTCAATTATTTATGAATGTAATTGTTTTTCCATGTTACAAATGCATCAAATTCACGCATAAGGGGT
CAAAGAAGACGTTCTGAGTGAGAAAAGTCCTCTAAAAAAATTCATAGAATAAAAATCGCGATAAAAGAGCGCATGTTTCAATGTTTTTATGTTTCACGTTGGACGTTA
AAGGGTTAATTTAAAATTAAGAAAATAATTAAGGGTGAAATTCACTAGAAAATCGAGGTTTTTTTAAATCGTTTTTTCTAGGTTGGTCTTAAAACTAAGTCAGTCTTA
AAATTTTAAGTTAGTTATTTAGGTCGGGCTTAAAATTTTCAGTCGATCCTAAAGTTTTAAGTCAGTTTTTCAGGTCGGGCTTAAAATTTTTGTTAGTTTTTTTAGGTC
GGGCTTTAAAATTGAGGTGTCTTAAAATCTTAAACCTTTTTTTAAGTCCGATTTAAAAAACTGACTCAAAGTTAAAGAACGGCATAAAACCGACCTAAAATTTTAAGC
TTGACTTAAAATTTTTGCCCCAAACATTTCATATTTAGGCCAGCATATGACCCAATGAACGCGAAGGGGTTAATTAAATCGAATAAATTCGACGTATTTTTTTTTTTAA
CAGATGGCTCCAATTTTCTTCGACTACCCGCGCACGCCAATTGGTTTGAAG**AAGCTCAACATCTCTCTGGCTACATTTGAGACTTACCTCAAGCGCTCTGGGACGAAGTA**
CGCTGCTGCTGATCATCTCACCATCGCTGACTTCCCTCTTGTAACGGCCACCCTCTGCCTCGAGGCGATTGGCTTTAGTCTCGATGAGTACCCCCTTGTTAAGGCNNNNNN
NNNNNNNNNNNNNNNNNNNNNNNNNNNNNNNNNNNNNNNNNNNNGATCATCTACAGACCGCACCTGTTGCATTCCTCTAATAAATTTTAAAATTTGCATTTCTCACAG
CAGAAAGATTTATGAGAGGATGGCTAGAAAATTGAAAGTAGTTCTCCCTCCTTTGTGCTGATTAATCAGATATTAACCCTTTCGCGTCTATATTTCTCAATCAGAACT
TCTTCTTTGACATATTCTGCGTAAAATCTACATATTTTCAGTTAAAAATATATATAATTAAGGTAATTTAAAATCAAGAAAATAAGTAAGGAAATTCACTAGAAAGTC
GAGGTTAAATTTTTTAGTCGGTTTTTCAAGTTTGGTCTTAAAACTTTAAGTCAGTCCTAAAATTTTAAGTCAGGTTTTTAGATCAGTATTAAAATTTTAAATCGGTCC
TAAAGTTTTAAGTCAGTTTTTTAGATCGGTATTAAAATTTAAGTCGGTCCTAAAGTTTTAAGTCAGTTTTTAAGTCAGCCCTAAAATTTTAAGTCAGTTTTTTAGATC
AGTATCAAAATTTTAAGTCGGTCCTAAAGTTTAAGTCAGTTTTTAAGTCAGTCTTAAAAAGAACGACTTAAAAACCGAATTAAAATTTAAATATAGACCTAAAAATCG
AATTAAAATTTTAAGCTCGACTTACTTGTGTGAATTTTGCCTGAAACGTGTCATATTTAGGTCAGCATATGACCCAATGAACGCGAAAGGGTTAATTAAATCGAATAA
ATTGGGCGTTAATTTTTTTTTAACAGATGGCTCCAATTTTCTTTGACTACCCGCGCACGCCAATTGGTTTGAAG**AAGCTCAACATCTCTCTGGCTACATTTGAGACTTA**
CCTCAAGCGCTCCGGGACGAAGTACGCTGCTGCTGATCATCTCACTATCGCTGACTTCCCTCTTGTAACGGCCACCCTCTGCCTCGAGGCGATTGGCTTTAGTCTCGATGAG
TACCCCCTCGTTAAGGCTTGGTACGGCAATTTCAAGCAACAACATCCAGATCTGTGGGCCATTGGGGNNNNNNNNNNNNNNNNNNNNNNNNNNNNNNNNNNNNNNNNNN
NNNNNNNNNNNNNNNNNNNNNNNNNNNNNNNNNNNNNNNNNNNNNNNNNNNNNNNNNNNNNNNNNNNNNNNNNNNNNNNNNNNNNNNNNNNNNNNNNNNNNNNNNNNNNNNN
N

>LLOJ002711_a (>112 aa; ~2 exons) | EDITED (PARTIAL GENE - EXON 2 MISSING)
MKLYAVSDGPPSLAVRMALKALDIPYEHINVDYCASEHMTEKYAEMNPQKEIPVLDDDGFFLPESIAILQYLCDKYRPDSELYPKDPKARAIVNHRLNFNSSFYYSSI
SMYV

>LLOJ002711_b (>161 aa; ~2 exons) | EDITED (PARTIAL GENE - N-TERMINAL GAP, C-TERMINAL GAP)
LDIPYEHINVDYCASEHMTEKYAEMNPQKEIPVLDDDGFFLPESIAILQYLCDKYRPDSELYPKDPKARAIVNHRLNFNSSFYYSSISMYVMAPIFFDYPRTPIGLKK
LNISLATFETYLKRSGTKYAAADHLTIADFPLVTATLCLEAIGFSLDEYPLVK

>LLOJ002711_c (>87 aa; ~2 exons) | EDITED (PARTIAL GENE - EXON 1 MISSING, C-TERMINAL GAP)
MAPIFFDYPRTPIGLKKLNISLATFETYLKRSGTKYAAADHLTIADFPLVTATLCLEAIGFSLDEYPLVKAWYGNFKQQHPDLWAIG

#########################
####### GST omega #######
#########################

####### PPAI000142 - GSTO #######
>AJVK01076482.1 forward | **PPAI000142** (pre-editing) | PPAI000142_a (post-editing)
TTATTATTATTTGAAACTAGAAACCACACTATGGTTCTTTCGAGTTCTACATAGCATCATGTACATAATACAATTGAATATTCTGAGCATGAGAATACAATTTCTAAT
TGATATGTTTTTCCTCACAAGGCAGGTGTGTATGAAACACAAATATGAGTATAATAGATTTTGAATGTCTCACTTTACAATTGTCGTAACTGTCTAAAATATAGCTAT
AGTATTCATCTCTTAAGACGATATTATATCATTTACAAATTATCTAACATATTGTGCGACCTGTACGACTGATGACGTGTTTTCAGAATTCTCTCGCTAATATCACAT
AATTCGTGTTTAATTCCCATCTTTAACACAATAAAACAATTGCGATGAAAATATCTAGACAAGTGGAAATATATTAATCTCTAATTAGTTAAAATTGAATTTACTACA
ACGATAAAAGCTTTTTTAATCGCACTTTAGATCAAATTGACAGTACAATTAAAAATAATGTTGAATCATTTAAAAGTAATTAAATCAAATGTCCATTATTTTTATGAT
CTAAAAATTGTCAAGTGACCACAAATTAGTTTTTATATTGTACAATTTAGGGTTATTGAATTTATCTAATCCATGCAATATCTGCAATAAGTGGAAGCACGATGCCTT
CAGACGGCTCAAGCTTCGAACAACTGATTTTTTTCAACACGTTTTTAATGAATACGATCCATTATAATATTATAATTTAATAGCCAGTTCAATGTCTCAAATTTTCTA
ATAAAGAGTCATAGGGTAAAGTGATATAATTTGGAATTAGTGTTACAAAGTTGGACAATTCGCCGGTACAAGTTGGACATGGCTTTTTTCGTGATAAATGCAGTACAA
GATTTGTTTTTAAGCACAAGGAACCATATTATAAAACTAAAGCAATAAAAATATACAAATAAAAATGAAGTACTAAATTTATCAAGACAAAAAGGGGAAGGAGGTACA
CACAATACGATGAATGGTTATTAAATAAAATAATTAAATTCAAATTTATAATTACGATAATTACAAAAAAAAAATTATCATTATAATAATAGGTCTATGTGGTCTATGT
GAATATTATAAATAAATAGGTGTGATTAGGAAGGAATCACACCTAACACAAGGGAAGGAATATTCGTGGAGTATTCTTAAACTCATTAGGTCCTATTTATAAGCTTTA
TCAGATATCCTTAAAACCTTTTACAAAATTCTTTTTATTCGTTTATTTATTTACATTGGACTATTAGAGTATTATTATCCGGAAAAAGAATTTTTTTCACACTAAAAT
AGGGTTAAAAACAGACAGATGGACAGATTGACAAAAAATAAGTATATGGACAAAATACATAAAAAAATGTCCTCTATAGGGCAAAGTGATAAAAAGTTGGTCAGTAGTG
GTACTAGTTGGACAATGGTACAATTTGGACAGGGCAGTACATATATATTTGAATCTGGTACCATCGCTTTTCTAAACAGAATAAAAATTCGGATCTTAAGCACAGTTT
AGACTCGCACCATGCACAAATGGCACATGACTTCATCGGCTGCCCCCCAACCCCTCCTCTTTAACTAACGCGATGTCAAAAGTAACTCGAAAGTTCAGTCACTTGCCG
GAATCTTTGGGTATTTGGCACACAGCAAAAACTACGGTTTTTAATAAAAGAAATAAGGTTTGTTCAAGAAGTTTAACCACCTGTGTAGACCTCAGAAAAAGTGAAAAC
ATGAGCAACGGAAAACACTTAGCTACAGGTGAGTTATTCATATATCTTTTGCGGGTTATTTTGGGATTAATTTATCTCTGATAATTCTATGTAGAGTCAAGATAACTT
TGAAAAATTTTATTAGGAATTTTCTAATAAATTATTTTAAAGATTTACTATATGCACAGAATTTTTTTTTCTTTTCTAAAAAGTGCTAATTTATTATAAAATAGTAAA
TATACTTGTAAGATATGTGGAGAAAGTGATTTTTTAAAAGAAAAAAGCGTCAGTGGCATATTTTTAGAAATGACGTAATAACATTGAAACTTCTCTTTTTCACTGAAA
TCTTCATGCCGTGAGAAACATCAATAAAATGTGTTGTATGGATGCAAGAGTTATTTCGTAAAATATTTTAGGTGCCCCCTTGCCCACCCTTCAGGATGATGGTAAGAT
CCGCCTCTACTCCATGCGCTTCTGCCCATATGCCCAACGTGTTCATCTTGTTCTGGATGCTAAGGATATTCCCTATCATACGATCTACGTAAATCTTCAGGCAAAGCC
AGAATGGCTCTATGACCGAAGCCCTCCAGGTACCGTTCCCGCTGTGGATCTACCCAACGAAAGTGGAGGTGCCAGTCTATATGAATCTTTGGTCATATCAGACTATCT
CGATGAGAAATTCCCACAGAGGCCCTTATATCCACGTACACCTTTGGCAAAAGCCAAGGAGCGTCTGCTGATTAAAAAGTTCGATACAGTGATTGACGTGATGTACAA
AGTGTTTTTGGGGGAACATGTCCCTGGAACTTTGACTGAGATCAGCAACAGACTTGACTTTTTTGAGAAAGAACTTCAAACACGTGGATCCGACTTTTTTGGCGGGAA
TGTACCCGGGATGGTGGATTATATGATCTGGCCATGGTGTGAAAGAGCTGATATGCTTACATACCTTCTGGGAGATAAGTACGTCCTGGATGAGGAGAGATTTCCCAA
ATTGGTTAGTCATCCATTTAAAAGTTTAATAAGTTTTAAATAGATTCACACACTGAGAAGGAGGGTG

>JH661954.1 forward | **PPAI000142** (pre-editing) | PPAI000142_a (post-editing)
AAATTTTATTTTTAAATAATATTAATAGAATTATTTATACCGATTTTGGATCAATACTGGAGGGTAAAATGAACATTTCCGGAATGTTATTTTAATTTTTTCGGATTT
CTCTCAGTGCAGTGTTCACAGTGGGCAAAAATTGCACATCTTGACTGTTTTAACAAAAGATCAAGATCTAATGATAAATAATTCTATTAATATTATTTAAAAATAAAA
TTTTATAGGTCAAGTGGAGAGCTCTG**ATGAAGGAGGACAAAGCCGTTAAGGGATCCTACCTGTCAGGTGAGGTGCATGCCAAGTACATGGAAGGACGTCGACAAGGAAATG**
CTGATTATGATATGCTTGTGTAAGCGGAAAATTGCCCCTACCCTACAACTAACAATATATATCAACTAATTAAGTGTGGCCTGCACTAATTTTCACAATCATTCATTCA
ATTTCTTTATCACTATTTTTTTCTGCGGCCATATCACAGAACTTTCGATTTTGTATACACTATGAAAAAAAATAGATTGCTATTTTCTTATATTTTGCTTTAAAAAAA
TCTAAGAGAATTGGATTTTTTATCTTTATTTCAGAAATATTGCTAAGAAACAGCGAACGTCCTAATTTTTGTATCATGATTTTATCATTTGATCACTCTGGCACTGTT
CTTACGATTTTTATTTTCACAACTTTGCATTTCAATGTAAATCAGGGCCTTTATTAATATTTTCTATATCACTGTTAATGAGAGAAAAAGATTTATTTTGTACTTTAA
AAAAAGCATGCATGATAAAGCGATTTAAAAACGAATCAATGTAAAAGGAATTAATAAAGATTTTTGTAAGCAAGTTTATTTCGTTAGCTGAAAGAAAAAATGTTTAAA
GTTGAAATTTTAAAAGTATTGCGTACAGTGTTTACACGACCTTCGTCTTGAATTGGTGCTCTAACATGATGGTTCAGCTTATGTAATTAATTAAGCAATCGTTCATAT
TCACGAAATTTTTAATGAAGGTTTCAAAACAATTTTCCAGAAAAAAAAATCAAATTAGACAAATTCACTATGCAGTACACAATCATTTGTACATTTTTCAATTTGCTAA
ACCGCCTGCAAATGTGTTCAAGCTTCTTACCTTTGACTTTGACTCAAGATCTCCCTTCTGAAATTGTGCCCTTCTCTCTTTTCCAG**ACGTCTCGGAAGGAAAAGAATCT**
TCCACCACACAATTGAGAGAATTTGAACAAAAAAGAAACCATTTCACATCTCCTTAAGAAAAAGCCTTTAAAATACCTCTTAAGAGAAAACAAAACTCTTAATTTTATT
CTCTTTTCTTTTTTTTTACTCTTTAAATTAAACTTTTGGGTTGGTTTCCAGTCATGTCTATTTCTTTTTTCAAATAAATTCATAATTTATTTTTATAATAAATGTATAT
ATCGTAAAAAATATAATCACTTGAATAAGTTTTTTTGTTCTTATATACAAATCTTCAAATTGCACGAAAGAACATCGACGAAATATTCTATATATATTGATTTTAAT
TAGGGAGGAAAACACGGGATGTGCGTTTACTGGGTCTATCTGTTAACTCTATCTCTGAAATATTTTCTTTTATTTATTTAGTTTTTTTTGTTTTTTACGAAAAAGATG
AAGATTTTAAAATAACAATTTCATAGTTTTATTAATTATCTGTTTGATAAAATACCTTCATAAGGAATTTTTCTCAAAAGCAGAAAAATTCTATTATCGGAAACCCAC
TAGAAGAGTTGCATGCAACTATTTCGAAACATTTTCTATTACTCGCTAAGATAGAGTTTTACTAGAGGGAAGATATTTCGCGTAAAACAACTTGAGCTAAAATTAGTA
TAACTTGGAAATTTCACGGTAAATGCCCTAAAAGTTTCATCTGAAAATTTTACAAATCAATTTCTATTGATCTTTAAAAATGATCTGGTTAAAAAATCGTTAGCTTTT
CTTGTTTTCGATCTCAATAAATTAAATATGTAAATAGTTTCAGTAAAGAATAAACTAATCAATAAATGCGAAAATATGATAAATTTAATTTTCAAATCATTATAAACG
AGGGTCCCGGTCAAAATCCCGAAAGCCAAAATCCCGAACGCCAAAATCCCGAATGCCAAAATCCCAAATTCTTAAAAGTGTCATAGCTACTCTTACGATTGTACCCGC
GCTTGCTGGGGGCAAAGGGAAATCTTCTGTGTCTTGGGAAATTATTGTAAACAT

>PPAI000142_a (253 aa; 4 exons) | EDITED (EXONS ALTERED, GENE SPLIT ACROSS 2 SCAFFOLDS)
MSNGKHLATGAPLPTLQDDGKIRLYSMRFCPYAQRVHLVLDAKDIPYHTIYVNLQAKPEWLYDRSPPGTVPAVDLPNESGGASLYESLVISDYLDEKFPQRPLYPRTP
LAKAKERLLIKKFDTVIDVMYKVFLGEHVPGTLTEISNRLDFFEKELQTRGSDFFGGNVPGMVDYMIWPWCERADMLTYLLGDKYVLDEERFPKLVKWRALMKEDKAV
KGSYLSGEVHAKYMEGRRQGNADYDMLVNIAKKQRTS-

####### LLOJ009136 - GSTO #######
>JH689401 forward | **LLOJ009136** (pre-editing) | LLOJ009136_a (post-editing)
AAAAGCTCCAAAGCTTAAAAATACTACATAATTTAAATACAGAATCAGAAACATTTTTTCTGAACCCCTCTGTAGGAGAATTCCGTTGACTTTTTGAAGCGTGGAACA
GTGAAGATGCAAACCAGCGTGGAATGGAAGCATCTGGCCTCCATCAGCTGCCCCCCATTCCACTTCTCCTGCCCACCCAGTCACAGTAGGTG**ATGTCAAAAGTTGTTCG**

TGAGTTCAGTCACTCGCCGGAGTTGAAGATTGTTGTTTGGCACACCGCTAAAGCCACCTCCGTTGGTTTCTTTGCACCTCTTCTGAGGCATCTTAAAACTTGGTCAGTCCAC
CACAAGTGCCGTGAAATGAGCAACGGAAAACACTTGGCTACAGGTGAGTTGATTCGCCGGCAATGAGCCCCCAGAAAATTCATGGCGAGGTTGTGCATAAATTAAAACCA
CGAAGGAGATTTTCTTGGGCGTGTTGCATGAAGAATTCTTCAAGAACAAAAATTTCTTAAAGGCTTTAGATGAGTTTTTGGGAAGGTTTTGGGTGTTCAATGAAATTT
TCTAAGGGGCTCAATGAATGGGTGAAAGTGATTTTAGAGAGAGAGCGTCAGTGACATATTTTTAGGCATGACGTAATAACATTGAACGACGACTCTTTCACTGAAACC
TCCTCCTGAGCAGCAGCAACAATACAGCAGTAAAGTGTGAGAATTTCAAGAATTTCTCCATTTTTCTTATAAGAAAAAATATTATTTAATATGCTGCTATAGAGATTA
GAAGGAGATTAGTTGAAGATTGAAAGAAAGAAAAAAGAGGCCTCAGAGTTCGAGTTTTTTTTATCTCCCTCAAGATAAGAAGACTCTTGAAAAAATCTCAAAGAAGAA
ATTGATGAAGATAAAAAATTTCACATTTCAGGTGCTGTGTTGCCGTCCCTGAGCGACGATGGGAAGCTCCGGCTGTATTCAATGCGCTTCTGCCCGTATGCCCATCGTATT
CACCTCGTCCTGGACGCCAAGGACATTCCCTACCACTCGATCTATGTGAATTTGAAGGCAAAACCCGAATGGCTGTACGACCGTAGTCCCGGTGGCACCGTGCCGGCTATTG
ATTTGCCCAACGAGAGCGGCGGCGCCCATTTGTACGAATCCCTCGTCATTGCTGACTATCTGGATGAGAAGTTCCCACAGCGCCCCCTCTACCCGCGTACCCCACTGGGGAA
GGCCAAGGAGCGGCTGCTCATCAAGAAATTCGACACGGTCATCGAGGTGATGTACAAGGTCTTCATGGGCACTCACGTACCAGGCACCATCACGGAGATCAGCAACAGGCTG
GACTTCTTCGAGAAGGAGCTCCAGGCACGCGGAAGTGACTTCTTCGGCGGCAACGTACCCGGTATGGTGGACTACATGATCTGGCCGTGGTGCGAAAGGGCTGATATGCT
CACCTACCTATTGGGCGACAAGTACGTCCTCGATGAGGAGAGATTCCCCAAATTGGTGAGATTTATTTAATTTTTTTAATGAGCTTTTAACCCTTTCTCGTTCTTTGG
ATCATACGAGGACTTTGAAGAATTTAATTCTTTTTCCAAGAGAGAAATTCGTTGAATTTACGTAAATTAGATCAAAGAATACGTTTTGACTTAAAAATATTCTCTGAG
AGTCTCATAAAATAAAGAAATTCGTGATGTAAAGATCGATTTTCTATGTTTCATGCGGACGCAAAAAGCCTAAATGGTTAAATTTAGTGTTCTTAATATTTTAGAACG
GAGCTAAATATTAAAGAGAAATCAAGTTTAGCTTTAGAAAGAAAACCAAGATTATTATTTGGGATTGATAATTAAAAAAAAGAAAAGAAAACATAGATTAGATAGATT
GATAAGAAATACAAACAAAAAATCAAGAATGATGAGAGTTTCAAATTGTTTGTATATTAAATTAAATACAAGAGCCACTCATATTGATTTCTGCAACTTTATACCTCG
ATATGATTAGAGGTTTAAGTCTATTTGTCGCTATCTGAATCAGCAAACATAGTCACAAGATATAGATTTATCAGCGGATGCGACATTTTGCTGATTAAACTAAAGCTT
TTGTTTTAGTTTATATTAGAGAATTAAATGAGTTTTTAATGGTCAGATATCAGTACATTTATTTTTAAAAAATCTATGTTTTAAGATTAAAACATAGAAAGTAAAAAA
TCGCGGTAATAATAAACATGTAAGAAATCATATCTTTTTGAAGCTCAAACAGTCAATTATTTGTTTTCTAATTATTCTTTTAAAATCTCAAAGTACGAAAACATCGAA
TTTTGAGAAAAATTTAAGTCATCGAATTTAGATTAGGACGTCGGAATTTATAATTTAATGTTTAAATTTTAAATCCATAAACTTAATTTAAAAGATTGAAATGGATGG
AAATGATAAAATTTTATAATTGACGTTAAAACATCGAATAAACTAATAAATTTTATTTCTTTTTTGCAAGTTAGTACCTAAGTTCTATTTATCAATTTCGATATTTTA
ATTGTTGGCAAAGAAATTAGAAAAGTTTTTCATTCATTCGATGTTTTAACTTAAATTGATAAAATTCTATGTTCTTCTATGAACATAGAAGGAATAGGATGAGATGAA
AATTCTCTGTTAAAATATCAGCAATAAAACATAGATTTCTTTTTATTTAAACAATATTTCATAATGATAAAAATTCAGAAATTAAAACGAATAAATTCGATATAACTT
ACCAATTTTCTTCTTCTTGTTTTAGGTCAAGTGGAGGAGCCTGATGAAGGAGGATAAAGCTGTGAAAGCTTCCTACATTTCCGGCGAGAACTACGCTAAATTCATGGAGAC
ACACCGCCAGGGAGTCCCTGACTACGATATGCTCGTGTAAGCGGACCTATCCACATCCTTTTAACAAAACCAATGTTCTCGACTAATTCCGTGTGGCCTGCACTAATTT
TCACACAGCAATTCATCACCTTTGACCTCCTTTTAATTGACTTTTTGTTTTTAATTCTCACTCAAACATTCAAAATTCTTTTTGGCCAAAATGCTAATTTATTGTTTT
TTTAACGATTTGTTTTGCGGGGAAAGTTTTCTAAAAACGAAATCTGGATTTTTTTCTCTACATTTCAGAAATGTTGCTAAAAAACAGCGAACATCCTAACAAATCTCGT
GCTTTTCAAGTGCCCACATCCTTTTAATTATTTTTTTTTATTTACAAACTTTTTGTCATTATAATTTGTCTCGCATAACGCTCTGATGGCCTTAAGGATCTTTTCCAA
GATTATGAAATTCTTTTTAATATTAGATGATGCATTTATGGATGTTTACACGATGTTGAAAAAAAATAATTATCAAAAATAA

>LLOJ009136_a (253 aa; 4 exons) | EDITED (EXONS ALTERED)
MSNGKHLATGAVLPSLSDDGKLRLYSMRFCPYAHRIHLVLDAKDIPYHSIYVNLKAKPEWLYDRSPGGTVPAIDLPNESGGAHLYESLVIADYLDEKFPQRPLYPRTP
LGKAKERLLIKKFDTVIEVMYKVFMGTHVPGTITEISNRLDFFEKELQARGSDFFGGNVPGMVDYMIWPWCERADMLTYLLGDKYVLDEERFPKLVKWRSLMKEDKAV
KASYISGENYAKFMETHRQGVPDYDMLVNVAKKQRTS-

#########################
####### GST sigma #######
#########################

####### PPAI002540 - GSTS #######

>AJVK01074729.1 forward | **PPAI002540** (pre-editing) | PPAI002540_a (post-editing)
CCACAGAAGCCATATAGAAAAATCGCGATCACTTTGCATTAAAAATTGCGCCAACACATACGATCGCGATCTTCAACCATATTTAGTCAACATTTGCTATTGTTATCC
ATTCATCCGGACTTATCCATTTTCTCATTCTCATATTCTCTTGTACATTGCAGATTCAGTGAACAGGATAGTGAGAGAGGAAGATCCCAAATATGCCCAACTACAAAG
TGATCTACTTCAATGTTAAGGCTCTGGCCGAGCCACTGCGTTTCCTCCTGGCCTATGGTGGCATTGAATTTGAGGATTTGCGTGTATCACGCGAAGAATGGCCCACCC
TCAAGTCCTGTAAGTATTATTGCTTCTCATCCAATGCATAGAGCATACAATAGATTTTTTTTGTCAATGATCGAGAGCCCACTTTTGGTGATTGAGCATCAATGACAC
AGTCACCCCAATCGAACACCCAGAAATAACCTGCGGTACTTTCGAGGGGGCCTATCAATTGATTGTCGCGATTTTCGAGTGATTTCTAATAATACTTCCAA

>JH662805.1 forward | **PPAI002540** (pre-editing) | PPAI002540_a (post-editing)
AAACTTCTTCGTGTGCTTGCATGAAACTGCCCCTCACGAAATTTCGGAACACAAATCAAAATTGTCAGAAACGTCTTAGATTTCATTCAATGCTAAATGCCTTATAAT
AAACATAAAACCACTAGTAATTTAATAAAATTATGTTACTTCGATAGATAAGTGCAATTAGTTCAGAAATTCTTGAAAATAAAATATTACAAAATGTTTCATTTTGAT
GCAGTTTTATAAAATCTATTACAGAATTCAAACGAGAAGCGACACGAAATTAATTATTTTTATGAACATGGATCTCAGTTGTCTCTTCAATAACATAAAGGTTTTATC
CCATTCCTTTCAAAGGGATAAGAAGAATATGCAAAAATTGAAACTGCCCCGTCGATAAACTGCCCCACCCTATTGAAGGTCAAAGGTCAACCACTTTATAGGGCCCAT
TTTTCAATCGATTTGGTTAAATTCAGATTTTTTGGAAAGGTATTGAAATTTCGAACCCGTCTGCATCGGTCTTCATCGCTAATGAATCGGTTATGTACCGATTTTTAT
GATTTTCACTTTTTGTGATATATGAATCAATACGATCTCTAGTAGACCAATAAGCACCAAAAGATGATACGAAAGACTAATTCACGGAGAGCTCTATCTCGTGAGTTT
GAGCATTCCGCAAAACTGGGACGCTTTGCTATCTCTTTACGAAACGAGTATAATCTGTGTTCAAACTAACCATATCCTTTTAGGTTCTCTTGAAACTACAAAGTGAAA
ATCCGTAATTTTGGATACCTGGAACAAGCCTGAAGATAAGTTTATCGAAAATTTAATCGATAGTATGCCTAATCTCATTCTTGAAATACTTCAGAAAAACTTTGGAAT
CACTAATTTTAAAGATTTGCATTAAAATCATTTCGAATAATAACATTACAAAAGAATTTATTGACATTAATGTAGTTTTACGATCAAAAACGTATAAAATTGATATTT
TCTCAATTCAAATTTAAAAAAGATGTTTCACTGTCAAAAAATTATCATAAATACGATCCAATCAAAGTATTTTGTGGAAAAAAAAATTAAATAAAAATATATATAGTAG
AAATAGAAAATGTGATTATTGACAAGTGACCTTATGGTTTTGATACACTGGAAAACCATAAAAATCAGCATATCTTCAATGGAAATATGGGAAAGCCGTTGCAGTGTC
AAAAAATTACAATAATCTATTGTCAATTAATTCAATTCAAAGTATTTTGCAGAAAAAAAAATTAAATAAAACGAATACAGAAAAAAAATTTGAGTTCTGTAAAGTGAC
CTTATGGTAATGGTACGCACTTTATATAATACAAGTTTTACATTGTTATATTCAAAACCAAATATGTAAAATATTTTTTACGGGATATATCATTCTGCATATATTACA
TTCTGTAAAATATTAATGAGAAAATTGCTAAATATCGCTATAATTAAAATCACATTGTTTTCTTTTAGTGGTTTTGGATTAATATTCCCTTTTTCTAATTGTATGCGA
AATTCACTATAAAATATTTGCGAAATGTTTTATTTTTTTAGTAATTCTCGTTCCTCAATTTTCGAACCAAGATCGAAATCTAAAAATTCAAATTTTTTTGTAATTATA
AACTATTAAGAAAATTTATAATATGCAGTTCATACTTTTTTAGTTCTTATAAAGTTTTCTTCCTAATACTCAAACGTTAAAACTATTGAAATTACTTAGTTTAATGGT
ATTTGTCATAAGAAAATCTTGAAATTTTATTTTTATCTTGGTATATCTTGGTTGTTTTAGATTCAATGAGCCCCTCAACTCGCTTTAACTAAACTAAACCATTAAATT
TAGTTGTTTTTGCACAGCACAGTACCTCGAATCACCAACTGATGGCTGTGTTGGTACGTTATCAGAGGATTGCTCAACACTCCATTCCTTATCACAATCAGCCTGTCC
CTCCTCTTCATGCGCTATTTCTTCATCTGGATGACTCATTTGTGGTTCAGGGACTTCTGCCTCTTCGACAGGCTCTTCATTTGTCTCCTCCTGAGGCTCAGGTGTTAT
CCTGTGCATTTCTTTGAACTGCCTTGGGCACCTTGGCTCCTCACAATTCTCCTGACACAGACGTTCAAACTGCAATCGACGCATGTAGACGATGGATTTGGTAGAGGA
ACCCCGGATTCCCCGTGGGGGCCTCTCAGGGGCTCTTTCTGTTTCACATTCAAAGGAACTTGTGCCACTGTCACTAGAAATTCGATTGAGGGCCTCATTCTCCATTCT
TTGGCGCCTCAATTTATAGAAAAAATCACAAAGCACAGAAGATCAAAAATTCTTAATGAATTATTATGAGCAAAATAGTCACACGTTCACTCAGTATAAATCTTCAAA
AAACACTGACACAGTTTTCACACTTGCTTCCAAATTGATGGGGTTTTTGCCTTATCATATGCTGATACAAATAAAGATACTTTAGTATAGGTGTGATAACATTTTCAG
GTGAGGTACAATTTCTTCAAGATTTTTTTCCAAATATGAAAATTTAATGAAAATATGAAAAATCTCGAAGATATTTCAGCACACAGCAATTAATTATAGCGTGTGAAT
TCCATTCAGTGCAAAATAGCCGCATATGATCTAAGAAAATCACTTTTTTCCACAATACATTCCTCACCATGAAAATCACAATACACTAGTAACACTTGTTGCTCTGAT
AAAAGAAAGCTTTTCAATTATTTTGTCTGAAAGGTTCAGTATCTAGCTAGAATATAGGAACATTTTGAAAAGCTTTAAGCTCACAAGTTTGAGAACACCGCATTGAAA
CAGTTTCAAAGCTCAAAGTCATCAACGTATCTTCTCTAATTTAAATTTAAATAATTTTTTGTATTATTTTTTTATTTCGAATATATTTGGAAAATATTGAAAGTATAAA
TAAATAGTCTGCATTAGTATCTTTATTGCACAGAAATATAACAAAAATTGTTGTTAATAGTTTTCAGTTCAGTTTATTTTTCCTTAAAGAAAGTAAAGTTATAACAAG
AGCCCTAAGTATCTCAACTGAATCAAAAAGATAGTGAAACCAAATAAAGCCCCGATTTTCAATTGAAATCAAATAAAGTGTAGCCCTTTAAAAAATTAAATAAATATT
TTGATACACTCAGAAAAATAATCGAGTATAAAACTTACTCGAATCGATGTTGAATTGACTCTTTTTCGTTGTGATTTTCGATTAACTATATTTTAGAGTTGATTTCAC
TTCTATTTAGGTGTAAATATTCGGAAGAGTTGTTTTAACTTTTTTTCCTAAAATTTATATGACAAAAGAGTTAAAATAATTCTTTTTTAGACTGAAAATAACTCGTTT
TAAGAGTTAAAATAACTCGTTCCAAGAGTTAAAAAAATNNNNNNNNNNNNNNNNNNNNNNNNNNNNNNNNNNNNNNNNNNNNNNNNNNNNNNNNNNNNNNNNNNNNNNN
NNNNNNNNNNNNNNNNNNNNNNNNNNNNNNNNNNNNNNNNNNNNNNNNNNNNNNNNNNNNNNNNNNNNNNNNNNNNNNNNNNNNNNNNNNNNNNNNNNNNNNNNNNNNNN
NNNNNNNNNNNNNNNNNNNNNNNNNNNNNNNNNNNNNNNNNNNNNNNNNNNNNNNNNNNNNNNNNNNNNNNNNNNNNNNNNNNNNNNNNNNNNNNNNNNNNNNNNNNNNN
NNNNNNNNNNNNNNNNNNNNNNNNNNNNNNNNNNNNNNNNNNNNNNNNNNNNNNNNNNNNNNNNNNNNNNNNNNNNNNNNNNNNNNNNNNNNNNNNNNNNNNNNNNNNNN
NNNNNNNNNNNNNNNNNNNNNNNNNNNNNNNNNNNNNNNNNNNNNNNNNNNNNNNNNNNNNNNNNNNNNNNNNNNNNNNNNNNNNNNNNNNNNNNNNNNNNNNNNNNNNN
NNNNNNNNNNNNNNNNNNNNNNNNNNNNNNNNNNNNNNNNNNNNNNNNNNNNNNNNNNNNNNNNNNNNNNNNNNNNNNNNNNNNNNNNNNNNNNNNNNNNNNNNNNNNNN
NNNNNNNNNNNNNNNNNNNNNNNNNNNNNNNNNNNNNNNNNNNNNNNNNNNNNNNNNNNNNNNNNNNNNNNNNNNNNNNNNNNNNNNNNNNNNNNNNNNNNNNNNNNNNN
NNNNNNNNNNNNNNNNNNNNNNNNNNNNNNNNNNNNNNNNNNNNNNNNNNNNNNNNNNNNNNNNNNNNNNNNNNNNNNNNNNNNNNNNNNNNNNNNNNNNNNNNNNNNNN
NNNNNNNNNNNNNNNNNNNNNNNNNNNNNNNNNNNNNNNNNNNNNNNNNNNNNNNNNNNNNNNNNNNNNNNNNNNNNNNNNNNNNNNNNNNNNNNNNNNNNACACGGAAAGCAC
TGTCTCTTGAGTTCGAGCATTCCGCAAAGCTGGAATGCTTTGCAATCTTTTTTCTTGTTAAATTTTGATCAACGAAGCTTCTCGTCTTTAAAGCGTTATACTTCGATA
TAGTCCACAAGAGATCAAAAAATATTGAAAAAATATCAAGAAGCAAAAGTAAATCCGTTTAAATAAACAAACCTTTGATTTTCACTTTAACACAAGAAGATGATATTT
TGATTCTTTTAGTGGATTCAAATCAATGTGAATTCAGGAAAAAGCCGACTCGTTTTTAAAGTATCCTATTAATTTTATTCTTCAAGCAATTATTTGAATACATTTTAA
AGATTACATTTTCATAGTTATATTATAATTTCCGTCTAAATATTTTCTAAATATAATTTTTATAAAATAATAGTTAAATAAAACACAGAAAAACATATAGGTCAGAAC
CACGATTATTCTGAGTGTTTAAAAAAAACTCAGTAAAATTTTATAATTTTGTAATGATTTTTTTCATATAATTTAAATCAGATTCTTTGTCATCCTAAAAAAGGCTAA
AGCTTAGATTAATATTTCCATTTACTTTAGGAAATATAAATTTAGCTATAAGTATAGTTCTAATTTGTCTAACTCTGTATTCTCTCTAACACGACAAATCTAAACAGG
TAATGTTTTATAATCAAATCACAATGCAGACTAAATCAGCTCTTAGACGCCTTCTGTGCCAAGATTTGGCATTCTCAACCTGGTCATTGCCTTTTAAATTGATATTTC
GTCATTTAACGATCTTTCGTTATAGTAAAAAATTGTTAAACGAGTTTTCTTAAATCGTTAATGCATCTTCTACAAAAGAATATCTTAAAATCTCTCGAGTATTCCATT
TTATATGCCAAGGTCATCTTCTTTTGTAGAAGAGATTAAAAAACTGGGCGGGAAATGGTATTAGCTTCATTATTCAATTTAACTTTCGCCTCACTTTGTTATCATCTA
ATCACGAAGTGCTCTGTCATTGTAAAGAATTTGATATGAAAATCGCAAATGTTCTGGCACTTTTCAGTGGGCGAATGGAGTGATTTTTTCCTCTTTCAAGCGCGAAAA
TATATTCACTCAATTTGTGTTATTTTTGGCTGTCTTATCAGCAAAAGTTCTGGGTGGAAATTACCGGCAAACTGTTTACTTTTATGAGAATATATGGGATGTGTTAGT
TGAACTAATTCGACCCTGGAGATCGTTGATATAGTGAAAGAGCTTCGTCATGTCGAAGGCAAATATCCAGTATATGGTGGCAACTGAAACAGGGGAGACCAACACAGT
CAAGTGTCATGGGAAGAAAGAAGTGCTTCCCGGAGGGTGGAGTGTGCAGCCAATACACGATCTACGGGTAAGTTAATGAGGAAGACTGAGAGAGTGGTTAAAGTGAAA
TTCCACTGGTGTTGTACGGATAGGAGAAGATAAGGCAATTTCCTAAAGGGGCAGATTAAATTTTCACGGCTTTTTCCATCTTCGTTAAAGGAATCGGTTAAATTCTTC
TTACTATTAATGACATAGTTTTAAGGCCTAAGAATATTATGAAATTATTAACATCATTAATATTTTTTGTTGAAGTTTTGTAATTGATTTCATAAAATTACTTGAAA

ACGGATTTCTTAAAAAGAAAAATTGGAAGTATTGTAAATATTTATCGAAGTATAACAGTTTTATAATAGTTAAGGGGGAAGTGCCCATGCGTTATACGATCCCAAGCT
TCGTAATGACTAAATTTTACTTATGTTTCTGAATAAATATGACTCCGCAATATATTTTAAAAACTGGGCAACTTCCCCTAGTTTTTAAATAATATACAGTGGTGGCAA
AAATAATAGGACCGCCCTTGCTAAGCCTACTTTTTTTAACTCTTGTAATTTCTTATTCCAATGCGTTGAAATAATTTTGATGTAGAAATGTTCAAATGATATCCAAAG
ATCCATAGAGATCTCTATTTTGACATGTAATACGTGAATAGTGATAATCTGTTCGGACAATAAAATATAGGACCACTTTTATATATAGTAATAAACTCAAATTTATTT
CAAGTATTTAAAAAAAATAACAAAATTTTAGTAGAATGTCAATAATTATTTAGTGTCTTGTCAATTAATTTTCCAAGTTTGGATAAAGAAAACTATGAGAAATTGGAG
CAAATGTAGAACTTTCTCGTTAGAGACTCTATTAGATTTTGAGGCTACGAAAGCATATGGAATGATCGTGAATATCATAGCTGACTACATTAAATTACGATAATCAAT
AATCCATAACGTAATATAAGTGTTTACGTAAACANNNNNNNNNNNNNNNNNNNNNNNNNNNNNNNNNNNNNNNNNNNNNNNNNNNNNNNNNNNNNNNNNNNNNNNNNNN
NNNNNNNNNNNNNNNNNNNNNNNNNNNNNNNNNNNNNNNNNNNNNNNNNNNNNNNNNNNNNNNNNNNNNNNNNNNNNNNNNNNNNNNNNNNNNNNNNNNNNNNNNNNN
NNNNNNNNNNNNNNNNNNNNNNNNNNNNNNNNNNNNNNNNNNNNNNNNNNNNNNNNNNNNNNNNNNNNNNNNNNNNNNNNNNNNNNNNNNNNNNNNNNNNNNNNNNNN
NNNNNNNNNNNNNNNNNNNNNNNNNNNNNNNNNNNNNNNNNNNNNNNNNNNNNNNNNNNNNNNNNNNNNNNNNNNNNNNNNNNNNNNNNNNNNNNNNNNNNNNNNNNN
NNNNNNNNNNNNNNNNNNNNNNNNNNNNNNNNNNNNNNNNNNNNNNNNNNNNNNNNNNNNNNNNNNNNNNNNNNNNNNNNNNNNNNNNNNNNNNNNNNNNNNNNNNNN
NNNNNNNNNNNNNNNNNNNNNNNNNNNNNNNNNNNNNNNNNNNNNNNNNNNNNNNNNNNNNNNNNNNNNNNNNNNNNNNNNNNNNNNNNNNNNNNNNNNNNNNNNNNN
NNNNNNNNNNNNNNNNNNNNNNNNNNNNNNNNNNNNNNNNNNNNNNNNTCAGATAAGTCCTGACTGAGTTGGAAATAGATTTTGCACAACAGGGTTATTTTGAAGGAA
GAATATGATGATGCAGTTAAACCACATACATAAACCTTTATGGCTTTCAGACCGGATATTCAGACAAGCAGTCTCGGTGGTTTTTGAAAATTTGAGAGGCCATTTGCC
TTTAATATCTAATCTTAATTCAAAATCTTTCCAAAAATCTTGTCTGATAAGAAATTATTAAGATTAAGCCAGATAGATAAACCTAGGTCTGACTGAAGCCTTACCCTG
AAACCCGATTAGATGATCTCTTTGACAATTCTGCATTTTATGTATAGGTTACACTTTAGGTAAATGATAAATCTTAAGTCCTAAATCTCTGTGAGAAGTAGGTTCACC
ATCTTATGACCAGTATTAACTATTTTCCTTGAAAATTGCTCATTGCGGTCATTAAAGAAAACGAGCAATTTTATTTTTATTGGAAAAAAATCGAAATATATCAAATAA
ACAACATTCTGTATTAAAAAAAAAACATTTTAAACTCTCTAATATTTTTTTATATACAGAGGAATTAGGGTAAGTGTGCCAAATTTCGGCATAGTTGCATGCAAGCAC
CAAAGTCTCAAGTTTGAAATGTAATATCTTTAATAGAAATTGATTTTATTCATTCCTTCTACTTAAGGAGTGTTGCTTAGAACCTTGTAGACAGTTTATCGTCTTTAT
TTACTTTAAAATCATTCTTAATACATTTTAAAATAAATAAAAATGTAGACATAGCTTTGGTGCCCTATTTTGGCCACCTTCATTCTCATAGTTTTTTGCCTTCGGGAA
TTCTTCCAATGTCTTTTTCACATCATCTCGTTTGTCGAAGCTACATTTTTTGTTATTCTTTTGAATTGTATAATCTCTAAAGTATGTAGAAACTAAAAATTCATGCAA
ATTCGAAAAACAAAAAAGGTGGCTGGAATTGCAAGCTGGCTAGAATTTGGCACACTTACCCTACGTCATTCAAAATTCGATTTTTACTCCTTCGAAATAACAGGGGCA
TGACATTTCCTATGTTTCCCCATATGTTTCTAGCGCGCCGAAAAAACTTTTGGGTTTATTATCTGTTCTCTGTCACTGTAGAATGAATTAGCAAATAATAGTAATACA
AAATAAAAGCCAATTTAATATAGAATAGGCAACCGAAAACCCCAAATTGCCAACCTACATAGTTTTGGAGATATCTCGTAAAATGCGTATGAAAACAGGGAAAAATTT
AGCACTAAAATTGGTCGCATTTTTTAGAGCTAATGTCACCCCCCTGCGAAATAACGTACTTTGGTTTATTAGAAATGTTTAAAAATATATAATTTATTAATTTACTAT
AACATTTAGACCTAAGATTTAAGCAAATGGCTTAACTTATCTAAGTACTCTTCATGAAATATTTTTTGGAAAAAAATCGACCGAAAATTGGATGTTAAGAACAAATTA
TCCTCTAGATCTTGAAAAAAAATAATAAAATTGTTTTTATTGATAACTTCGGTANNNNNNNNNNNNNNNNNNNNNNNNNNNNNNNNNNNNNNNNNNNNNNNNNNNN
NNNNNNNNNNNNNNNNNNNNNNNNNNNNNNNNNNNNNNNNNNNNNNNNNNNNNNNNNNNNNNNNNNNNNNNNNNNNNNNNNNNNNNNNNNNNNNNNNNNNNNNNNNNN
NNNNNNNNNNNNNNNNNNNNNNNNNNNNNNNNNNNNNNNNNNNNNNNNNNNNNNNNNNNNNNNNNNNNNNNNNNNNNNNNNNNNNNNNNNNNNNNNNNNNNNNNNNNN
NNNNNNNNNNNNNNNNNNNNNNNNNNNNNNNNNNNNNNNNNNNNNNNNNNNNNNNNNNNNNNNNNNNNNNNNNNNNNNNNNNNNNNNNNNNNNNNNNNNNNNNNNNNN
NNNNNNNNNNNNNNNNNNNNNNNNNNNNNNNNNNNNNNNNNNNNNNNNNNNNNNNNNNNNNNNNNNNNNNNNNNNNNNNNNNNNNNNNNNNNNNNNNNNNNNNNNNNN
NNNNNNNNNNNNNNNNNNNNNNNNNNNNNNNNNNNNNNNNNNNNNNNNNNNNNNNNNNNNNNNNNNNNNNNNNNNNNNNNNNNNNNNNNNNNNNNNNNNNNNNNNNNN
NNNNNNNNNNNNNNNNNNNNNNNNNNNNNNNNNNNNNNNNNNNNNNNNNNNNNNNNNNNNNNNNNNNNNNNNNNNNNNNNNNNNNNNNNNNNNNNNNNNNNNNNNNNN
NNNNNNNNNNNNNNNNNNNNNNNNNNNTCATGGTCGAGCATTTATTAAAGTTTAAAAATTAAAAAAGTAAAAACATAAAAACATAGTGACATATTATGACACACTGAC
AACCATTGGCAGTTACCCTGTTTTAAACTTTTCAATTAATAATACTTGTAAAAAGCATTAAGTAACTATTCCATCAATTTCATGACATTCCAATATTTTCTAGAATAA
ATAAAAATTCACCTTAGTAATAAATTTTTCATGAAAATTATTAATCTTTCTGCATTTCTGGATAGTAAACAGTTTTGATCAAACTATCAAGATGCATATCTGTGCAAT
ATACGGAAATATTATATGTATTTTCTAAATTGCAACCAAAAAAAAACTTTTAATACGTCAAAAAAATCTAGGAAAGATTAATTGTTTTTTTTTAAATTCTTCTCTTTA
GCAAGAATTTAAAAAAAAATTGTCGGATGTTTTATCAGATCTTATGATTAGTAATTATTAATTCATTTACCAGACACGAAATACAGTATCGGCCAAAAGTTTCTTGAC
AAAGTTATATTCGAGAAATCAAGTGGGAAGAATTATTAAAAAATTATATTTTGTTATTTTTCTTTTTACAAAAGACTTGCCTGTAATCCATAGATATTATTTATGCAT
TTCAAAACAAAAATAATTTTATATCATATCGCAGATAATTGTCTTTCAAAAGTTGATCAGTTTCTATCGTCCAAAAGTTTCTTGACACACTTCAAAAAATTACTCAAA
TGATCAAATACGTGCTTCCAACAATATATTATTCTTTTATATTTTGAAGTTCCGACATTTGAGAGGAAAATTAGTGAATTTGAACATCTCTAAAGTTTTCAATGGTAA
ATGTATGCACATTAAAATGATGATAAAAGGAAGTAATGTATTTCTTGTTAATTTATAAGAAAAAAAAAATAAGAAATTAGAAAACTTAAACTTTAAACCTTAACATAAA
ATCATAAATTTTAAACATTTACCTATAAACCGTAAACCCTAAAACGAAATTGGTTTAAGTAGAATTAAGTTCCCCAGTATACGATTAATGGTCTGATATCTTTTCAAT
ATCAAAAAGGCTATTTTATCGAGTTAGAAACGTGTCCTATAAATTGAAAACACAATATATTTAGGCCTAAAGTTTTTTTTTTATATGGCTTAATGCTCTTAACTGCTC
TAATATCTCAAAAGACGATTTTTTTAGAAAAAAAATTTAATGAAAGTCATATTTTATGGGCAAATGGCATATCAGACATTGTAAAATCATTACAATTGCTTGTTATTTA
GAAATCCAAAACCTTTTGGAACTATTTAATAGTTCCAATATAGTTCCAATAATACGGGCTTTAGATCTAAGGTTTAGCGAAATGACTTAGTTTCCTCTAATTTCTCAT
AATAAAATTTTTTAGGAAAGTTTTAATTTGGAATCGAATATTAATGGTAAATGACCATTTAGGGGAAAGTGCCCATGCTTTGCACGATCCCAAGCTTTACAATGACTA
CATTTTTCTTATATTTCTGAATAGGAAAAATTCTATGTTTCTGGTGTTTTCCCATCAGTCTATTTCCATACCCGAAGAAGGAGCCATCAAGCTCCGAAACGTTGGCAA
GGAATAAAGGAGGAGAAGATCAAATATCAAAATATCGTGTTTTAACTTCTTATAAAAAAAACCATTAGAATTTTTTGTTTTTTTTTTTAGAAATTCAGCTATTTCGGG
AACCAGGCTTAATCTCCAGTCTGAAATATGCACAATATGCTAGACATATATACGACATTACCCGATTTCATTGCCATTAATTTTTCACTATGTCGTCTCTCGACTTAA
GCCATAAGTTTGTCTAGATCATAAATTTTTAGTCTTATGGAATCTACACAGAAAAAGAAATTTTCGTCAAATATTTCTTTCAATTTTAAAGTTTAATACACTGAGAAA
AAAGGGGGTGCGATTAACTTTTTTTTCCTTGTAAATTTAACGCTTTTTAGGTGTAAAAATACATCAACATTTTTTATGTTNNNNNNNNNNNNNNNNNNNNNNNNNNNN
NNNNNNNNNNNNNNNNNNNNNNNNNNNNNNNNNNNNNNNNNNNNNNNNNNNNNNNNNNNNNNNNNNNNNNNNNNNNNNNNNNNNNNNNNNNNNNNNNNNNNNNNNNNN
NNNNNNNNNNNNNNNNNNNNNNNNNNNNNNNNNNNNNNNNNNNNNNNNNNNNNNNNNNNNNNNNNNNNNNNNNNNNNNNNNNNNNNNNNNNNNNNNNNNNNNNNNNNN
NNNNNNNNNNNNNNNNNNNNNNNNNNNNNNNNNNNNNNNNNNNNNNNNNNNNNNNNNNNNNNNNNNNNNNNNNNNNNNNNNNNNNNNNNNNNNNNNNNNNNNNNNNNN
NNNNNNNNNNNNNNNNNNNNNNNNNNNNNNNACTCAGAAATTCTCGAAAACACGAGTATCTTCACAATCTTCGACACAGACGGTAAACAATATTACAGGTCGATATAC
TGAGAAAAACCGCGGTGCGATTAACTTTTTTCCTCATAAATTTAACACTTTTTAGATGTAAAAATATATCAACATTTTTAATGTTAATTTTACATCTTTTTAAGGGTA
AATTTAACATGAAAAAGGGTAACTTTAACCCATGAAACACCTAAAAAGCATAATATTTACACCGATTTCGAATCAATACAGCAGGGCAAAATAAACATTTCCGGAATG
TTATTTTAACTTTTTCAGATTTCTCTCAGTGTACACTGGCACTGTTCAACACCATTTTGTCCCTTGTTCTTCCAAGTTTAAATACCCTTTACAAGAATTTAAAAAAAA
ATAAATACTTTAAAAACCACTGTCATTAAATATCCAAAAGAACAAAAAGATATTCTGCTCACCTGACCGGAAAATTGTTGGCTGTTGTGTGGTTGTTGTAGCTATAAA
TTGAAACTTTGAGATTATCAACAGAGAAGTTGAAGTATTTGTCTAATAATCACGATCTTTCTGCAGCC**ATGCCGATGGGTCAGATGCCCGTGCTAGAAGTGGATGGACGA**
CGAGTTCACCAGAGCATTTCGATGGCACGCTACCTGGCGAAGCAAGTCGGGCTCGTTGGTGCTGACGCGTGGGAGGATCTGCAAATTGACATTGTGGTGGATACAATCAATG
ACTTCCGCCTCAGTAAGTTTATCTTAGATGATTAAAAATTCAAATTAGAAAAATCCCTAATAAACTTAATATTCTTTTGCAG**AAATTGCTGTTGTATCTTATGAGCCTGA**
TGATGATGTGAAGGAGAAGAAGCTGGTCACACTCAACAACGAAGTCATTCCCTTCTACCTGGAAAAGTTGGATGCCATTGCCAGGGAGAACAAGGGACACTTTGCATTGGGT
AAACTCACATGGGCTGACCTCTACTTTGCCGGCATCCTCGACTACCTCAACTACATGACCAAGACCGACCTCACTGAGAAATATCCCAACCTCAAGGCTGTCGTGGACAATG
TGTTGGGCATTGAGAGCATCAAGGCCTGGGTTGAGAAACGACCTGTTACTGAAGTTTAAACTCTTCAGATCTCTCATTCATACTAAAACAAAAATCAATCCCGGCGCATT
TGATCGCCAATCACACCTACATCCCCCTGCACCTCAAACTATCCCCTATTTTTACGAATAACATCACTCTCTGTAATTTTTTTGCCGGAAGGAAATAAAGGCCTTTCT
TTCAGTTTTCGGTGCTAAAAATCTGACTTGTTTTACTTTCATTTTCACATTTTTACAGTGTTTGTCGGTCCGATGGAAATCAGCCTGAACTTTGTAGGTTATCAAATC
ATTCCTCCTCTTGATTTTGTGGAACAAGCTCCAATGTATTCCCATCTTTTTGCCAATATCTCGCACGCAAAGGTTGAGTTGCTTCTTGCAAAGCTCAAGCACTTTCTT

TTTTTTCCTCTTGTCCTGAATTCCAGGCTTTTAGTCACTTCCGCCTGGCTTCTAAGCAACAGTCTTTGACTTCCTTGAACCGAAGTATAATCTTCCGGACAGTGGAGC
GGAACAATTTTCGGTCAATTTCGCGATCTCAGCATAACTTGCATTTGGATTTTTGAGATTATTGTCAACATTTAATCTTTGGACGCTCTCCATCTTTTTGCCCTTACT
CAGTNNNNNNNNNNATTGGTTAAATAATTCAACTGGCCGAGTTGAGGAACCGGCCGACTAGAGGGTACTTTACCTTACACGCTTTACCCCTAAGTGTATGCATTTTTC
GATGCGGTAAATTTTAAATGCGTAACATATGAATCATTGACACTTAAAATAGCCCAAGAAATTGTCGCCAACATATTTTGCATATTTTACGGCGTTTTCGTAACTTAT
TGAAAAGAGGTCGTCTCCTGAAAGTATGCAAAATTTCTGTATGCATAAAACCGTTTCACGCACCTTTTCTTGCTCCCGAAAATATGCATAATCTGGGATCCCCTGTAA
GTCAAATTTTTCAAAGAAATCTGGGAAAAATATAAAGTGTTTGAAGCCAGAGTACGAAAACTGAAAGCCTTCCCCTATTTTCTAAGAAAATCATTTTTTTATACAAGG
TATAAGTATTGTGTGTAGTATCAGTCTTAGTTCACTTTTTTCTGAGTGTATAATCCAACAATCATTTGACTCTTCTAGAAAATCGGTTGGAATATAGACTTGATATTG
TATATTCTAGCAAAATCTTATGTAAAATTATCGTCTTTACTACGAGAATCACTAGCAAGGACACCCCTTTTTGTATTGGCGGGGAAATTTCCCTCAAAATATCAGGCC
CTTCTAAATGTTATCCTGCACAGATGATGGTAGCACAGACTGCCTCAAAAATTCCTTGCCAATTCAATTCCTGCAATCTTCCCTAAGGATACAATATCCTCAATCCCC
TCTATATTGCTACAAATGAGTGTTGAATGAAAAAGAGGGAAGTATTTCGAAGGGAAGAAACATATAGTTGAATAGAAGGTTTGCAGCATCTGCTGTAGGCTTGTTGGT
ATTCTCGTTTGAGGAAGTGTTCATGTCCTTTCGAATGGAATTTCCCGGTGAAAATTTTCCCATGCAAAATGGTCGGAAAATTTCCCTCAATGGCGCCTCACCACGCAT
TCAGCTGGAAGTGTTTCTATCGAAATTTCGCGAGTGAAGCCCATTTCCTGTGAGGACGTTTTTAGTGTTTATATCAAATATCCTTTTCAAGTGGAATTCAGGTGAGAA
ATTGCAAGAGGTTTTGAGAATGTCCATTCGGGTGATTTGTGGGGGTGATTGTCGCGTGTGTTTTTCCGCAGTGATTTTTCTTCTGCATTGTTAAACGGTGGAAGGTGT
TAGGAAATTTTCGCGTGTTTGTCCTCATAACATATTTTTAATTCCAATCTCGTGATGATTTTTCCGCGAATGATGCTGCCCTGGGTGGCAGAATTGGCATTTTTTGGG
GTCATTTTGATGGTGCAGTGATCACTGAAGGACACACTGTGTGACTCCTTGCAGGAGGAACACCCCCTTTGGTGAAAGAGAGAGAAAGAGAGAGAGAGAGAGTGGTGC
AACTT

>PPAI002540_a (203 aa; 3 exons) | ACROSS 2 SCAFFOLDS
MPNYKVIYFNVKALAEPLRFLLAYGGIEFEDLRVSREEWPTLKSSMPMGQMPVLEVDGRRVHQSISMARYLAKQVGLVGADAWEDLQIDIVVDTINDFRLKIAVVSYE
PDDDVKEKKLVTLNNEVIPFYLEKLDAIARENKGHFALGKLTWADLYFAGILDYLNYMTKTDLTEKYPNLKAVVDNVLGIESIKAWVEKRPVTEV-

[The full length scaffold JH662805.1 up to the gene PPAI002540 was extracted and searched for evidence of the first part of the gene. A BLASTx search of the 6-frame translation of the scaffold upstream of PPAI002540 against *Aedes aegypti* and *Anopheles gambiae* predicted proteomes identified nothing. The scaffold was very fragmented, with a lot of sequence gaps, which may account for this. tBLASTn searching identified exon 1, unannotated, on scaffold AJVK01074729.1. tBLASTn searching using the missing 'middle' exon identified nothing that did not belong to already-annotated genes.]

####### LLOJ009036, *LLOJ009037* (*LLOJ009037* in italics) - GSTS #######

>JH689328 forward | **LLOJ009036**, ***LLOJ009037*** (pre-editing) | LLOJ009036_a, LLOJ009037 (post-editing)
TTGGAGAGGGATAGAATACAAATAAGAAAAATTAAAAAAAAAAAGAAGAATTAATACGATCGCGCGCCAAATTCAGAAGGAATATTTTGGGCGCCAAAGCGATCCGCC
ATATAAGGGCAAAGAGAATGGGAGGTTGTCTAACGCGAAGAAATCTCGTTTTCTTGCAGATTCACCCACAAGACGCAGTTAAGAGAAGCAAA**ATGCCCAACTACAAAGT**
GATCTACTTCAATGTGAAAGCCCTGGCTGAGCCACTGCGCTTCCTCTTGGCATATGGTGGCATTGAATTTGAGGATCTGCGCGTATCACGCGAAGAATGGCCCACCCTAAAG
TCCTGTAAGTATACAGCTATGAATTTTTTTAAG**GCAGAGCAAACAGCCTCTTTTTTTGGCAATTGACACACTCACCCCGATCGGAACAGAAATAA**CCCACCTTCATGGGC
CTATCAATTGATTTCTTCACTAACTCACAATTTCTAATAATACTTCCAATTGTGTGACCCACATTGAAGCATTCAATGCATGCAATTCACTTCAGATCTTCCCTTCAA
TCTACTTTTGTTGCCTTTTTTTCCTCATCTTCATCATCTTGCAAACAAGATCGCACGATCAGTGATCCCCAATGTGATCAATATTCGCGCGATCACAGCTCAGGAATT
CCCTCACAACATTTTCCCACGATTTTTGTGGATTAATGAAAAATTTTCTCTACCAATTTCCACCACTCACGAGGCTCACGAGAGTCACGATCGCGCCAAAAAAATAAT
TTAAGTAAATCTTCCTAAATTAAGTATACAAACGCAGGAATTTTTTTTACAACACTGTAAAAGAGAAGAAAAAACAGAATGGAGAACACTAAACACTCCAGAGTCAAT
TGAAGTGGTGTTTAGAATTTAATTGGCCATAAATTTGCGTGATAAGAATACTTAAACATTGTAAAATGAATAAGCGGGTGAATATGCGATAAATTTCAATTAAGGAGA
GAATGAAAATTTGGAAATGTTTCAATTTAATTTAGGGGACTCAGGGGGGAGTTGATGCATACAGCTTATTAACACATCCCGCTTCTATCCTAACGTTTGATGTTNNNN
NNNNNNNNNNNNNNNNNNNNNNNNNNNNNNNNNNNNNNNNNNNNNNGTCAGTGAGTGCGCAATTGTCTGTGTGTGAAATTCCGTGTAGTCACCTCAAGGGCAAAATGT
CAAAACAAATGTGGGGAAAATTGAAAATAAATTCTTGGTTTTCCGGCTTTTCCATGTATTTCATGGCAGTTTTCCTATTTATAGTAGTGATAAAAGTGATCTACTAGT
GAAAAACCCACAAATTACGGCAAAAACAGGGGGTCGCAATTGGAACACTCGGGGGGTCGCAATTAGAACACCGTGGTGAAAAAGTGCAATTTTAGCAACTTTTTTTAA
TTACATTATTTCTCAGAACAGGTAAGAGTTAGAATGTTTGCATCTATGGAACAAAGTTAGCCTATTAAATGACCTTTCCAATGGTACCACACTTGGGAAGATTTAATT
CCTTTTTATAGGACTTTTTCCAATCCTTCTGAAACAGAATTTAGGGGGTCGCAATTGGGCCACGTTCCCCTACACATACAAATGGTATGGCTTCGATGATGTTCGGCC
GGCATATAAAAAGAAGCGCCGGGTGATTCGCACCTAGTCCACTTGGGAGGTTCGTCAGCTCGCGGTATCGTGCTATCCGGCCCACTGTCTTCAGGTAAGGCGGTCTAC
TCTCAGTGGAAAATGCGGAAAAATCTCAAATGGAAATATTGTGAAATTCCCTTCTCTCTCTCTGGAAAAATACTCTGTGCTCGATGATTGTGTTTAGTGATCTTCGCG
ATCGTAGCTCTATGTGGGTGATCATTTCATTTTTTTGGTGATCTCAAGGTCATTCTATTAGCGAATTCTCTTCTTTTTTTTGTGTGCACCAAAAGGCAAAATGAGTTT
AGCAAATTGAATTAGAAAAGTGAGTTTTTTTGTTACAAATAAGAGAGAAAAAGTGTCAACTGTAGATCAGTGTTGATCGCGAACAACGCCCACATAGAGGAAACTATT
TAGTGATCGTGTCGAAGAGTGGGGAAAAAAACGTTCTCAATTGGAATTTTCTTGGCGCCTGAGAAGCTTTTTTGGAGAGGGATAGAATACAAATAANNNNNNNNNNNN
NNNNNNNNNNNNNNNNNNNNNNNNNNNNNNNNNNNNNNATCCCTCTCCAAAAAAAGCTTCTCAGGCGCCAAGAAAATTCCAATTGAGAACGTTTTTTTCCCCACTCTT
CGACACGATCACTAAATAGTTTCCTCTATGTGGGCGTTGTTCGCGATCAACACTGATCTACAGTTGGCACTTTTTCTCTCTTATTTGTAAACAAAAAAAGAGAATTAA
TACGATCGCGCGCCAAATTCAGAAGGAATATTTTGGGCGCCAAAGCGATCCGCCATATAAGGGCAAAGAGAATGGGAGGTTGTCTAACGCGAAGAAATCTCGTTTTCT
TGCAGATTCACCCACAAGACGCAGTTAAGAGAAGCAAA***ATGCCCAACTACAAAGTGATCTACTTCAATGTGAAAGCCCTGGCTGAGCCACTGCGCTTCCTCTTGGCATATG***
GTGGCATTGAATTTGAGGATCTGCGCGTATCACGCGAAGAATGGCCCACCCTAAAGTCCTGTAAGTATACAGCTATGAATTTTTTTAAGGCAGAGCAAACAGCCTCTTTT
TTTGGCAATTGACACACTCACCCCGATCGGAACAGAAATAACCCACGGATGGCCCCACCTTCATGGGCCTATCAATTGATTTCTTCACTAACTCACAATTTCTAATAA
TACTTCCAATTGTGTGACCCACATTGAAGCATTCAATGCATGCAATTCACTTCAGATCTTCCCTTCAATCTACTTTTGTTGCCTTTTTTCCTCATCTTCATCATCTTG
CAAACAAGATCGCACGATCAGTGATCCCCAATGTGATCAATATTCGCGCGATCACAGCTCAGGAATTCCCTCACAACATTTTCCCACGATTTTTGTGGATTAATGAAG
AATTTTCTCCACCAATTTCCACCACACACGAGGCTCACGAGAGTCACGATCGCGCCACAATTAATCCCTCCTCGGAGTATCAATGTTAACAGGAATATTGAACTTCAT
TACATAACCCACGAAAGATGATTAAAAAAATGAAAGTGAAGAGCATTGCTCAGCAAAACATCAAACAATTTTATCAGTAAATACGTCGTGATAAATTGCCGCCATAAT
CATGGAAATGTAATTAAAAATAAAATCCGAAGAATGCCTCAAAACATCTTTTTCATAGGGGAAAGGGTGGTAATGTTAGTGAAAAATTAGAAGAATAAAATGAAGGAG
AAATCTCATATTTGAATAAACGTATTCCCTTTAATTTGACGAATTAACGTGATTATCCTTGAAGAGAAGTATTTGAAGGGGCTCTCATGATGTCAAACAACGTAAGGT
CTATTTAATAAAGGAGTGTAGAGTTATTATTTATGAAATTTTTCTCAAAAAAGACGATTTAAAATGCATTTTTCCAGACTTTTCCGATAGATTAGAATGATCTTCTCT
ATCCCAGGTTCCCCTATTTGGCCCTTATCACAAGCAGCGTTATCAAAACGTTTTTTTTTCTATTTACTCAGTCGATTATAAAAAAATAATTTAAGTAAATCTTCCTAA
ATTAAGTATACAAACGCAGGAATTTTTTTTACAACACTGTAAAAGAGAAGAAAAAACAGAATGGAGAACACTAAACACTCCAGAGTCAATTGAAGTGGTGTTTAGAAT
TTAATTGGCCATAAATTTGCGTGATAAGAATACTTAAACATTGTAAAATGAATAAGCGGGTGAATATGCGATAAATTTCAATTAAGGAGAGAATGAAAATTTGGAAAT

GTTTCAATTTAATTTAGGGGACTCAGGGGGAGTTGATGCATACAGCTTATTAACACATCCCGCTTCTATCCTAACGTTTGATGTTAATTTTCCAATTTTAGACATTTG
TTTTTTAACGTTTGACAATTCATTTTAACCTTTGCTGTTCTGTCTTCTAACTTTCGATCTTCTTGAATTTATTTTTTTCTAACTTTTAACATTTTCTTTTAACATTTG
ACGTCTTTTTTTTATTTGAGGTTTCTTTTCCAACGTTTGAGGTTTCTTTTTTAACGTTTAATGATTTTTTAACTATAATTTAAACTTTCATTTAACTTTGACATTTTT
CTTCTAACGTTCAATTTATAAATCGTTTGAAAATTCTTTTCTTGCTGATTGAATTTCAGCGTTAAAATAACTTACATCAACCTACGTCAACAACAAAAATTAAAATAC
ATAACTTCTTTTGACCTACGAAGTAAAACATAAAATTCCTGAAGTTTAAGAAGATGTTTAGACAACCAAATACTCTCCCTATCTCCCTCCCTTTTCAGCAACTCAAAG
CAAAAGAATTCTGAATTCTTATGAAAACTTTCATTCTTTTCATTGAAATTTTTAAATTCTAACACATTGTGTATTCAATTAAAATTTAAAAGAACAAAAAGAGATTCT
TTTTCAATGTATCTTTTCTGCTCAACCAGCTAGAAAATTGCCCCCTTATTGTGGGCTGTGCGTGAATGATTTTCGAAGCTCCCAGAGAGGAGGAAGAAAAATGAGGAA
TTTTCCTAAAATATTTCTTTTTCTGTGTACCTTTCTGGCCATTTAG***CTATGCCAATGGGCCAGATGCCCGTGCTGGAAGTAGATGGACGACGAGTTCATCAGAGCATTTC***
AATGGCACGCTACCTGGCGAAGCAAGTGGGTCTCGTTGGTTCCGATGCGTGGGAGGATATGCAGATTGACATCGTTGTCGACACAATCAATGATTTTCGCCTCAGTGAGTAG
AATTTTCCTTTTTCTTCGGTCTTTTAGTTTTTTAAATGATCTCTAATGCTGGAAAAATGATTTTCTTGCAG***AAATCGCCGTCGTTTCCTACGAGCCCGATGATGATGTGA***
AGGAGAAGAAGTTGGTCACACTCAACAACGAAGTCATTCCCTTCTACCTGGAAAAGCTCGATTCGATCGCCAAGGAGAATAAGGGACACTTTGCATTGGGAAAGCTCACATG
GGCTGACCTCTACTTTGCCGGCATCCTCGATTACCTCAACTACATGACCAAGACCGACCTCACGGAAAAGTACCCCAATCTCAAGGCCGTTGTGGACAATGTGCTGTCCATT
GAGAGTATCAAGGCATGGGTTGAGAAGCGACCTGTCACCGAAGTCTAATCTCCTCAAAACCATTCACACACACCCCAATGCACAAAAATCATCAACCCTCCGTTGGCATG
GCATCATTGTCAATGGATATTGCATCTGGATTCTTCTGCACCATGTGTCCTATAATTACTATTTTTACAAATAACACACCACTCTCAGTAATTCTGTTGCCCGAAGGA
AATAAAGGCCTTTCTTTCAGTTTTCGGTGCTAATTTTGTGTTTTTTTTCTCTCTTTTACTTTCCCCTGTCCTTGAATGCCCTTGACCTATAAAAAGGGGGGGAGATGA
AAAAGGAAGAAAGGATATCAAAAATCCTCTGATATTATGGGNNNNNNNNNNNNNNNNNNNNNNNNNNNNNNNNNNNNNNNNNNNNNNNNNNGGAAATAGTCTAAAAAC
TCAGGTTCTGATATAAGAAATGCAAAAAAATTAATTTATTATCTAACTAAGAAGCATTTTTTTTAATATAATAAGCAATCTTCTTTAAATCTTAAAGAATAAACAGTG
ATTTTTTGATATTAGAAAAGATCTTTTGATCCAAGAATTTATCTTCTAGTTTGTCTTCTTGTCTGAACCAAGTAAAGATATTTCTATTTGAGAAACCTTTGTTAATAA
CCAAAATTAGCCCTTTTGCGTCAACTAGCTTAACTAAATGTGAAACATCGGGAGGAATCCACAAATATCTTCGGCCTAGTTGATTTTTTTTCTATGTTAGGCCTCTGT
TTTATAGGCCAGACTTAACTTTTCTAAGGCTAAGCTATAATGGTTTTAAGGTTAAGTCAATTTGAATCAGGACAGAATGAATTTTTCTTTAAATATTTTGCAAAAACG
AAAAAATATTTTTCCACCAGTTAGCGTCAAAAGTCGGACCTCAGTCCAGAATTAACTTTGAAACTCATATATTCGATTGTTGGCTAAACGACTGTTCTTTTAAAACTT
AAACCTAGGTTTTAAAACTTAATTTTTATTAAGCCAAACTACGATGTTCAAAAATAAATTGTACAGAAAATCGTTTTTCCACCGGTCAGCGTTGGAAACTTAAGTCTG
ACATAAGAAAAGACTAGACTAACTTCAAGGAACAAAAGCCTGATTATAGAATGAAAAAACTTGGGCCAAGCTCACAAAACATAAAAGTCTGATCTTAACAAACTTAAG
TCTAGCTTAAAAAACCTAAAAG

>LLOJ009036_a (>44 aa; >1 exon) | EDITED (PARTIAL - C-TERMINAL MISSING)
MPNYKVIYFNVKALAEPLRFLLAYGGIEFEDLRVSREEWPTLKS

>LLOJ009037 (203 aa; 3 exons) | UNCHANGED
MPNYKVIYFNVKALAEPLRFLLAYGGIEFEDLRVSREEWPTLKSSMPMGQMPVLEVDGRRVHQSISMARYLAKQVGLVGSDAWEDMQIDIVVDTINDFRLKIAVVSYE
PDDDVKEKKLVTLNNEVIPFYLEKLDSIAKENKGHFALGKLTWADLYFAGILDYLNYMTKTDLTEKYPNLKAVVDNVLSIESIKAWVEKRPVTEV-

#########################
####### GST theta #######
#########################

####### PPAI000341 - GSTT #######

>JH662026.1 forward | **PPAI000341** (pre-editing) | PPAI000341_a, PPAI000341_b (post-editing)

TGGGGATTTTCTGCAAGGACATTTCTCGTATTGGGAACGAGGAAGTCCTTGAGGATTACTTGAATTTATTTTCATTGACAAATTGTGAATTTTTATCAAAGAAAACAC
CATTTGAGTCTATTTACTCAGCTGACACTTTTTCTTTACCTTTTATCTGCTGATCCACAGGTGTAAAGTTCAAGAGGAAAAATTTTGCAGAAATGTCGTCAATTTTCCTT
TAAATTCTACTATGACTTGCTCTCACCCCCTTCGAGAGCTCTAATCACATTTTTCAGGGTAGCGAACATCCCGGCAGAACCCATTCCAATAGCTCTGAGGAAAGGTAAATTG
GCTTTTGGGGTTCTTGGTCAGTGGGATTGTGTCCAGATATTCGGATTTAAGGATTTTCCCTGTTTCAGGTGAACATCTTACAGAGAAGTACAGGAAGGAAGTCAGCAGAT
TCCCTACCCTTCCCTGCATCAATGACAATGGGTTTAGGCTTTCAGAGAGCGTGGCTATTGTGAGGTATCTGAAGAATACCCGAGGATTTGATGATTTTTGGTATCCAGAAGA
TCCAAAAGCTCAAGCACTAGTTGATGAGTATCTGTCATGGACACACAATAACATCCGGATGACTTCTGGTCTCTATTTTATCACAAAGTGGCGTACTCCAATTTTGACTGGA
CAGAAGGCTGATGAGAAGGAAGTTGCTTTAGTGTTGGCCACCATGAAATGGACATTGCACGCAGTGAAAATTGCCACCTGGACCCCGAGGCTGTGGCTGATCTCAACTACGA
GATAAAATTAATTGTACCAGAGGGACTTGTCTGCGCCATCAGCAAATCATCCCCAAACACCATTCTCTGGAAGCATAAAGTAAGTGGGTTTATTTTATTTTCTTGTCA
ATATTACCTCCATCACAACAGAGTAGAAAAAAAATTGGAAAAGCGTCCTAGGCTTTGCAAAGTGCTCGAACTCGTGTCTTATACGGGCTTCAGACCTAAGGGCCTTGA
CAGACCTGAGGATTAGCCGAGAAACGGCTTAGTGTAATTACACTCGACATAATGGTTAAACTTCATTTCCATCATTTTCCACTAAGTCGTCTCTCGGCTTAAGTCGTA
AGTGTGTCTAGGGCATAAAAAGCTTAGCCATCTGGCTTAACTCCTCTAATTCCTTATCAAGCATGATTTTTTAAGAAATTGCTGTTTAGAATTTGATATTAACCCCTTT
AAGGACAATTGGAACACCGGTGTCCCATAAAGAAAATTATTTTCCTGACTACCCAAAGTTATTTTTTTTATGTTTGTACGTAATTGTAAAGTAGAAGGTTGAAGGAAT
CCAGAATATTTTTTGCAAGTCTCCAGCTATTTGCTAGATAGTTAAATATTCAAGCTCAAAAATGACGAATTTTTAAATTCTCAAATTCATAATTGATTTTATTTATTT
TTTATACTTCCAATTNNNNNNNNNNNNNNNNNNNNNNNNNNNNNNNNNNNNNNNNNNNNNNNNNNNNNNNNNNNNNNNNNNNNNNNNNNNNNNNNNNNN
NNNNNNNNNNNNNNNNNNNNNNNNNNNNNNNNNNNNNNNNNNNNNNNNNNNNNNNNNNNNNNNNNNNNNNNNNNNNNNNNNNNNNNNNNNNNNNNNNNNN
NNNNNNNNNNNNNNNNNNNNNNNNNNNNNNNNNNNNNNNNNNNNNNNNNNNNNNNNNNNNNNNNNNNNNNNNNNNNNNNNNNNNNNNNNNNNNNNNNNNN
NNNNNNNNNNNNNNNNNNNNNNNNNNNNNNNNNNNNNNNNNNNNNNNNNNNNNNNNNNNNNNNNNNNNNNNNNNNNNNNNNNNNNNNNNNNNNNNNNNNN
NNNNNNNNNNNNNNNNNNNNNNNNNNNNNNNNNNNNNNNNNNNNNNNNNNNNNNNNNNNNNNNNNNNNNNNNNNNNNNNNNNNNNNNNNNNNNNNNNNNN
NNNNNNNNNNNNNNNNNNNNNNNNNNNNNNNNNNNNNNNNNNNNNNNNNNNNNNNNNNNNNNNNNNNNNNNNNNNNNNNNNNNNNNNNNNNNNNNNNNNN
NNNNNNNNNNNNNNNNNNNNNNNNNNNNNNNNNNNNNNNNNNNNNNNNNNNNNNNNNNNNNNNNNNNNNNNNNNNNNNNNNNNNNNNNNNNNNNNNNNN
NNNNNNNNNNNNNNNNNNNNNNNNNNNNNNNNNNNNNNNNNNNNNNNNNNNNNNNNNNNNNNNNNNNNNNNNNNNNNNNNNNNNNNNNNNNNNNNNNNNN
NNNNNNNNNNNNNNNNNNNNNNNNNNNNNNNNNNNNNNNNNNNNNNNNNTATTTAAATTGTCCACACGAATTATACCTAGATTCATAAAATGACAAAAACAGATGGTTCTAACAGA
TACAAATATTAAATAATTATCATTTTTATTTATAATTGGCATTATATTTTCTAAAAAAATAAAGCATGATCAGGTATAAAGATTGATATTGTATCATAAAACATCAAAA
TAGGAAAATCTTTGAAGGTGTGCTATTTATCTTGTCGCCACTGTACATGCATGATGCATACATGACCTCAAAATGATTTTAAATGTAACCGTCGACAACTCGTCCGGA
TTACGGCGATCCGGATTAGAGAGTGGGTACTGTAATTATATCAAATTGATTCAAAGTCAATTGCAATAGCTATGCTATTATTTGTGAGCAACTGTATGAATTTGAAAT
AACTGTAAAAATTAAAGAAAACTATTATTCACCTGAAAATATTTCCTCGGAAAAAATTACAACACAAATTAAGCTGTAATAAATTTGAGCTTGCTCCATCGCATTGTCA
TAATCAACTTGTAAAATGTCAAAAAAGATCTTTTCAAATTACACTGCCGTTCGAATTAAAAATATGGAATTAGGCAGAAAAGATTTTATAATAGGGCACTTCCACTTC
CAAAGACTTCCAAGCACTTCATTTTCAGCCGTACACCACTTCCAACCCTAATATATCCCCCTTGCCCAAACCGTTTTTTTGCTCAAAATCTAAGAATCCAGAACAAAA
ATCTCTGAAAACAAAATCCTCAATATTAATTTTTCAATCAGGAATTTGTGGCTTTGATCCTCTGAATTCCCGGCCCAATCTTACAGCATGGTTTAGTCGTGTTAGGGA
GAAACTCGATCCAATCTTCACGGAACATCACAAATTCATCTACAAGTACGGTCAGCGGTTCCAGGAGGCTTCCAAACTCTGAAGGAATTTACTGCGCCGGCGTGGATT
CCATGAGTTCCACAGGCCAGGTGCTTTTGATTGTCAATGTGACCATGGATATTTTGGGGTGAAGGAAGGGAAAAAACAAGTGGTTTTTAATTAATAAAAGAGCATCAG
TGCGAACTGATAAGACTTCCGATTGAATGTTTTATTCCTTGAGAAGTGTACAGGTGTGACTAAAAGACTTCCAA

>PPAI000341_a (167 aa; 2 exons) | DOES NOT ALIGN WELL AT C-TERMINAL END BUT CANNOT IMPROVE

MSSISFKFYYDLLSPPSRALITFFRVANIPAEPIPIALRKGEHLTEKYRKEVSRFPTLPCINDNGFRLSESVAIVRYLKNTRGFDDFWYPEDPKAQALVDEYLSWTHN
NIRMTSGLYFITKWRTPILTGQKADEKEVALVLATMKWTLHAVKIATWTPRLWLISTTR-

>PPAI000341_b (>51 aa; >1 exon) | DOWNSTREAM OF PPAI000341_a, POSSIBLY ONE GENE BUT CANNOT RECONSTRUCT

QSGICGFDPLNSRPNLTAWFSRVREKLDPIFTEHHKFIYKYGQRFQEASKL-

####### PPAI003974 - GSTT #######

>JH660972.1 forward | **PPAI003974** (pre-editing) | PPAI003974_a (post-editing)

TTACTCCAGTGGAGACGCCGGGTAAGGTAAAATGGGAAAATTGAGAATTTTTGCTTTTAAGCTCTTTTTGCAAAGAATTAACAAAAATTGTTAATGATATTCTTAAGA
ATAGTGTTTAAGATAAATTGAAAACCTTGAGATTGAGATGTGTAAGTTTTTCTTTGGGAAAAACACCAAAAAATAAATTCTAGATGGTGTGCATGGTTACCCGGCATTC
CCCTAAGGAAACTCTCCTCAGTTGGGAAGTAAACATCACAGGGAAAGCAGCAAGAAAAGCTAAACTCCCGGGAAAAATGTCGAAGCCAGTGAAATTCTACTATGACCTGTTG
TCACAGCCATCCCGGGCGATGGTGATTTTCCTCAAATTGGCTAGAATCCCCTACGAGGATTTGCCAGTAGCCCTCCGGAATGGAGAACATCTCAGTGAGGACTTCAAGAATC
AGGTCAATCGCTTCCAAAGGGTTCCCTGTATCAATGACAATGGCTTCAAGTTGTCCGAGAGTGTAGCTATAGTGAGGTAAGAAGAGTCTCTTGCCCCCCAAATACAATAAT
TCCTGAGAATATACCCCGCTTTCCCAGATACTTGGCCAATAAGCACAAGATCCCAGACACCTGGTATCCACAGGATGCCAAGAAACAGGCTCTGGTTGATGAATACCTGGA
ATGGCAGCACAATAATACCCGAATTACCTGTGCCCTTTACTTCCAACTCATGTGGCTGAAGCCTCTTCTGACCGGCAAAAAGCCCACGGAGAAGGAAGTGGACACGCACCTG
CAACGCGTAGTGACCACCTTGGATGCCATTGAGAACATCTGGCTGGAGAAGACACCGTTTCTCGCCGGCAATGAAGTCACAGTGGCTGATCTTTGGGCCGCATGTGAAATTG
AGCAGCTCACGCTCACTCCGTACGACTTCCGCAAAGGACGACCAAGGCTCACAGCATGGCTGGAAAAAGTACGTTCATCCTCCAATCCCCATTACGACGAGGCACACAAAAT
CCTCAAAAAAACTTGCTGAAAAAACATCTGCAAAATTGTAGGAAAACATTTTATGTAAAAAATGTTGGTTGAGGGGCATTTTTATCGCAGAAAATAAAGGCTTTATCTCTG
ATCCAAATATCTCTCTTTGCTCTTTGCCAATTGGATGAAGAGGAACCCTTGACCTTTGAACCGTCCGCGATGAGTTGCTTTTATCAAAATTTATAGATGATGGCTCCC
AGAGGGCTCTTCTAAAAAAAAATCGCGGGAATGGTAATTTTTGCGCCAAAATGATACTCCTCATTAGCATTTTCTCACGAATTATATTTACACAGAAAATGATCTTAC
ATTTTTACTGATAAATTTGACAAAAAAAAGAGATAATTCCATGGTGTGTAAGAAATCTACATCTCTATGAAAAAAAAAACAGTGGCAATCATAATAATAGCAACATCC
ATTTTTAATTTCTTCTAAAAAAAAATCATAGTCATTGTTCTTATGGTTTTATGGTCTCTACACACTGTGAAAAATTTCTGTCAATATTTGGAATTGAAAAAATTTTGA
TAGTTTTTCCGTGTACACTCAATCCCGGTATTATGCACATTCTGTGGACCGAAAAAAACGCGCGAAATGGCATTACGCATCGAGAAAATTTGCATACCCGCATGGGCT
TTCTTTTATATAAAACGGCCGTAAAACGAATTTCGCGAGGAGTAAAAGTATAGTCAGAACAAAAAGATTCAACTGTTTAATAGAATGCATTAACAAACAGTACTTTT
TGGCCAGAGTTCTTCAATAAATGGTTAAAATATTTTTAAAAAAAATTACCTTAATAAAAGAAATTAAAGTTTTATTCTGAAAAAGTAGCAAAATGTTTTCAAATTTCCC

GCGTCGCAACATAATATACATTCAAGCATATTTCAAAAATGATTTCATAAATTGACTCCCAATCGTAGGCAAACTTGCATAATTGTATGTGCATAATTCCGTCAGGTG
CATGATCCCAAAGGGCTTAATGCCGGGACTGAGTGTACGTAATTTCCTTCAATATACCAAATTTTGACGGAAACTTTTTATAATGTGTAGTGAGATACCATAAAAATC
AAGAAAATTATTTTAAAACAAAAATTACTAACCGAAAAAACGAAAACCAGAAGTCCGAAACCCGCCTTGAAGCCTAAAACTCGATTTAAAACAAAATATTTAACAATT
TTATGCTTTTCTATAATGTTTTCCGCGCATTAGAGAAATTTGTGCCCATATTTGGCATTACAGTAGAGTCTCTTAAATTCGAACGCTCGGGGGGTATTTTTGACATTT
CTCACCTCCCAAATTCNNNNNNNNNNNNNNNNNNNNNNNNNNNNNNNNNNNNNNNNNNNNNNNNNNNNNNNNNNNNNNNNNNNNNNNNNNNNNNNN
NNNNNNNNNNNNNNNNNNNNNNNNNNNNNNNNNNNNNNNNNNNNNNNNNNNNNNNNNNNNNNNNNNNNNNNNNNNNNNNNNNNNNNNNNNNNNNNNNNNNNNNNNNNN
NNNNNNNNNNNNNNNNNNNNNNNNNNNNNNNNNNNNNNNNNNNNNNNNNNNNNNNNNNNNNNNNNNNNNNNNNNNNNNNNNNNNNNNNNNNNNNNNNNNNNNNNNNNN
NNNNNNNNNNNNNNNNNNNNNNNNNNNNNNNNNNNNNNNNNNNNNNNNNNNNNNNNNNNNNNNNNNNNNNNNNNNNNNNNNNNNNNNNNNNNNNNNNNNNNNNNNN
NNNNNNNNNNNNNNNNNNNNNNNNNNNNNNNNNNNNNNNNNNNNNNNNNNNNNNNNNNNNNNNNNNNNNNNNNNNNNNNNNNNNNNNNNNNNNNNNNNNNNNNNNNNNNN
NNNNNNNNNNNNNNNNNNNNNNNNNNNNNNNNNNNNNNNNNNNNNNNNNNNNNNNNNNNNNNNNNNNNNNNNNNNNNNNNNNNNNNNNNNNNNNNNNNNNNNNNNNNNN
NNNNNNNNNNNNNNNNNNNNNNNNNNNNNNNNNNNNNNNNNNNNNNNNNNNNNNNNNNNNNNNNNNNNNNNNNNNNNNNNNNNNNNNNNNNNNNNNNNNNNNNNNNNNNN
NNNNNNNNNNNNNNNNNNNNNNNNNNNNNNNNNNNNNNNNNNNNNNNNNNNNNNNNNNNNNNNNNNNNNNNNNNNNNNNNNNNNNNNNNNNNNNNNNNNNNNNNNNNNNN
NNNNNNNNNNNNNNNNNNNNNNNNNNNNNNNNNNNNNNNNNNNNNNNNNNNNNNNNNNNNNNNNNNNNNNNNNNNNNNNNNNNNNNNNNNNNNNNNNNNNNNNNNNNN
NNNNNNNNNNNNNNNNNNNNNNCATTCATCAAGAATACTAACTTTCTAATATAACCTCACAATTGACATTTTGAATCCAAACGTCGTTCGAATTTGAGAGATTCAGAT
TTGAGACTCTACTGTAATTGAAAAAAGTTTGACAGTTATTCGAAAATTAACATTTTCGTACATAATTCGTAACACGGTTTGGGCTAATGGCCTCTACATACTAAGGGA
AATGTATGTCAAAATTAGGCATTTTTGAAGAATTTGATATTTTCCCTGCAGCAGACATAAGAGGATTTGCTCCAATTTTACAAAAATTAACAAACTGTAGCATCCAGA
ATGGTCTCTACATACCAGAGAAATTTGTGTCCATATTTGAAGGTTTTTCCTCAACGGCCTCTACACACTAGAGAAATTTTTGTCCTTAATGAAGCAAATTCGCTATGC
TCGTTTAGAAAAAAAGCTCTTTAATGTCTTTATTATGGACAAAAATTTGTCTAGTGTGTAGAGTGTGTAGAAACCATACTGAATATATTGATTAAGAATTTATCAAAT
AACATTTTATCTTTATGACCCTTCAGCAACTAATAAAAGCTATACCAAACAAACACCAAATGCTGATAATGATACTCAGCATAATATTTCGAACAGTAAAAAAGTAGC
GAAACCGTGCTGAATTTCAGTATTCGGTGGTGGCAGTACAAGACATAGATTTTTACAAAAATCTATTAGCAAAAGCAATAGGGTGGAATAACCACACTTTTCGTCATA
TTGCCCCACCTGCCCCCCTTTTCGCCACTTATATTGATATCGCAATTTTTCATTATTTTTTGGGTAAATGAACTGCAAAATTATTGGTATTTCTACTGCTACGGATAG
AGTGAGAAAAAGTTATTTGTTTTTTGTACGCCCAAAAATAAAATTTTTTATACGCCCAATAAATATTTTTGTCTACTGAATATTGAAATACACTTGTAGAATAAATTA
TATTAGAGTTTATGTAGTTTTCATTTTATATTATTTTTACGCATGTTTATGTGAAAATGAGGCATGGCGGAAAGTAGGATTATTCTGAAATTGATTTTACCACCTGTC
GCCATTGCTTCTCATCTCAATAGATCACTTGCGTGAATCAGTTTCACTTGCATAGATTTCCAATTGTCAAAACTGCATTGTTTGATTCCGGAGTAAGTTTAATAATTT
ATTTTGTTTTTCAAATAAAAACGAAGAAAAATCATTTAAAGACAATGAGTAATAAACCCTACATACTAGAAAAGTTTATGTACATATTGAAGAGGTTTTCCTACACAA
GCGTAGGGAATTTGCTTCAATATGGACATAAATATCTCTAATGTGTAGAGGCCATAAAACTCAAATTGTATCAGTTTCAATCTACTTGAGAGTTTACTGTATGGCCTC
AACTCATTAGAAGTGATTTTCGTCAAAAAATGCTTTTTTGAAGGAAATTGCCTGCACTATCTCGAAATTCCGTCAAAAATGCAATTTTTGAGAAAAAATTGCTTCCAAT
GTGTAGAGGCCTTTCAAAAGCATTTTTTAACAAGTCACACACCTTTTTTGGGTTATATTAGCTTTCTTGCTGAGTACTACAACAAATTTGTTCAATATCGATAAAGAA
AAAACATTTTTAATAAAATATTGAAAACTAATTAAAAAAAAAGAAATAAATATGAATCAATGAAAATGTAGAGTCGAAATCGGAAATTTGAAAGAAAATCTCACGTAT
GGCATCTACACAATGTCCATATTGAAGCAAATAACCTATGCTTGTGTAGGAACACCTCTTCAAAATGGACATAAATTTCTCTAGTGTGTAGAGGCCATAAATCATTGA
GATTTTATTCAATAAAAAAATATGCAATGTAATCTAATTTATTTATTTCTCGAAAAAGAATTAACCACTATTTTGTTGATTTTTTATTTTTATCAATAAGGAGAAAATCA
TTGGATAATTCCCATTTTTGCTGACAAAGGGTTTCAGGTTTCTCGAGTTTAGCGTTGCAATTTTTGGATTCCTCGGGTTTTCCCAAGACTTTCTCTAGCAATTGACAA
GGAAGGAATCACCCTTTGCGTTTTATATTTCTGCCATTTCATTTCATTCTACTTTTTCCTTTTTAATAAAAACAAGCCAATTGAGTAATTTTACTGTTTGAACTCCA
CGGAGACAGCAGTATATATAATCCCCAGCTCTAGAGGTCAAACGGTAAGAGATAGCGACTTCGGACCTAAGGGGGACCCCCCATAAGTCTACCCAAGGATCGTTAATA
TGCCGCTCTAAAGTCCTTTTGAGGTATATATAATCTAAGTCACGAGTTCGAGCATTTCGCAAAGCCTGGAACGCTTTGCCACCCTTTTTTTCAACTTCTAACTCTGTA
GAACATAAGAAGTACATGAGATTACCGCTAGTAGCCGTTAGGGTAAGTGTGCCAAACAAGCGCCTGGCAAGTTGGTCTTTTTATGCCTTTTTATATGGAGCTATTTGA
AGCCAATTCCTAAATTGATCTCATCCCAGCTAGAACGTCCGGCGAATCCTCAAACCAACTTTTTTTTTTAAANNNNNNNNNNATCTTATAACACGGAAAAACTTCTTA
CTTTTTTAAATATTTAGCGTAACGATCACGAGTGAAGAAAAATTCCCATACGCATTTGTATGTGAAATTAAGAAATTGGCTTCAACTTTGAACGCACTCGCCGGGGAC
TAGGTGCCAAATTTCGCCATAGTTGCATGCAAACGCCAAAGTCTTAAGTTTGAAATGTAATATTTTTAAT

>PPAI003974_a (227 aa; 2 exons) | EDITED
MSKPVKFYYDLLSQPSRAMVIFLKLARIPYEDLPVALRNGEHLSEDFKNQVNRFQRVPCINDNGFKLSESVAIVRYLANKHKIPDTWYPQDAKKQALVDEYLEWQHNN
TRITCALYFQLMWLKPLLTGKKPTEKEVDTHLQRVVTTLDAIENIWLEKTPFLAGNEVTVADLWAACEIEQLTLTPYDFRKGRPRLTAWLEKVRSSSNPHYDEAHKIL
KKLAEKTSAKL-

####### LLOJ005757 - GSTT #######

>JH689696 reverse | **LLOJ005757** (pre-editing) | LLOJ005757 (post-editing)
ACATAAGCTGTTACAATTAAAAGAAACCCTCCGAGCAATCGTCAACTAAATCAGCAGCTCTTGCATCTGTTGTTTAGCAACATAAAACTTTTGCTGATTAAACCGACG
AAAATAATCACAAGAAGATTAATTTTATTGGAGGAGAAGGAAAATCCAGAACTTGCCCAGAAAAGTGAAAATTCTGTGAAAATCCCGTGAAA**ATGGCAAAGCCTGTGAA**
ATTCTACTACGATCTCCTATCCCAACCATCGAGGGCTATGATGATCTTCCTGAATGTTGCAAAGATCCCGTATGAGAGCCTCCCGGTAGCCCTGCGGAAGGGAGAACATCTC
ACGGAGGAATTCAAAGCCATCAATCGCTTCCAGAAGGTCCCCTGCATCAGTGACAATGGCTTCAATTTATCCGAGAGTGTTGCAATTGTCAGGTAATGCTGGATTTTAGTA
GATGTGCCGGTGCTGGGAACATCATTGAGGAGATTTTATGCTCTTTCAG**GTATCTCGCTGCAAAGCATAAAATTCCAGACAGCTGGTACCCTACGGATGCTAAGAAGCAA**
GCCAGGGTGGATGAATACCTGGAATGGCAGCATCTCAATACACGCATCAGCTGTGCTCTGTACTTTCAGCTAATGTGGCTGAAACCCCTGCTGACGGGCAAACAACCAAAAC
CCGAAGCAGTTGAGGAGCATTTGGGGCGCGTTGTGGACACTTTGGATGCAATTGAGAACATTTGGTTGGAGAAGACACCCTTCCTGGCTGGAAATGACATCACTGTCGCTGA
TATTTGGTGCGCATGTGAAATTGAGCAGCTCATCCTCACTCCCTATGACTTCCGGAAGGGACGTCCACGTCTCACCGCTTGGCTCGAGAAGGTACGAACACAGGCTAATCCC
CACTACGATGAGGCTCACAAAGTTCTCCGGAAGATCTCCGAAAGGACTTCGGCTAAGCTGTAAAAGGAGGCTCTACAGGTGGTCAGGAGGATGAAGGATTTGTGGTTCAG
GGTTTCATTTCTCTTAGACTGATTTATGGTGCTTTATCTGCAAAATAAAAATGCCTTCTTGCTGAAGATTAAACTGGAGTCTTTTGACCTTTGACCGCAGTTCTTGAG
GGGCATGATCCGCCGATTTGTGCCTCTCCCCAAAACTTCTAGATGGCACACCAAACGCCAATTTGTGTCCAACAAGAATCCAGTCTCCCCTATAAAATCTTCATAGTG
AAAAATTAAGGAAAAAAGGATTTTTTTTATTATATAATAAATTTATTGGGAAAAGAAATTAATTATATAAAATAAAAATAATAAAGTGTGGAGAAATCTACAATTTTTTT
TGCTTAATTCCTTATTAAGATTAGTAACATCCACGACATAGAGATGATTTTTTCCTTTATGGTGCTATTCACGTTTCTCATGTTAGAAAAAAATGATCATGACATTTT
TTTTCATTCCATCTCACTCCCACTAATGTATTTTCCTATCTTTCGTCTTTCTCTCACGCATGTTTCCGACATCTTTTTTTTCATGGCATGCTTTTTTTTCTTGCGCACA
ACTGTTTTTATTTCCTTGAATTTTCTCAACATTTGGTGTATTTTCCCCAAATAAGTGAAAATGTACGAAGTAAAAATCAAAACATTCTTTCCCTGACCAATTTTTACG
GGATTCTTTTTCGGATTTTCACCACACAAATCACTTCCTGCAGGCTTTTTGTACCTTCCTACAGGTCATCTGCATACGGAAGGTCTGTCAATGAATTGTCTCCTGGAT
CGTACAAAAAACTCCTGAACACGGAAAAACTGGTCCGGAAGTGAATGTTTTGCTGTCGAACTTCACTGTTTTCACATATTTTTCCCGATAAATGAACGTAAATTTCCA
TTTTCACTGTTTTTCTGGCACTGTGGACTCTTTAGCTAAGCATTTTCCACTTTTCTTTGGGGAAAATATCCCCAAATGTTGAGAAAATT

>LLOJ005757 (226 aa; 2 exons) | UNCHANGED
MAKPVKFYYDLLSQPSRAMMIFLNVAKIPYESLPVALRKGEHLTEEFKAINRFQKVPCISDNGFNLSESVAIVRYLAAKHKIPDSWYPTDAKKQARVDEYLEWQHLNT
RISCALYFQLMWLKPLLTGKQPKPEAVEEHLGRVVDTLDAIENIWLEKTPFLAGNDITVADIWCACEIEQLILTPYDFRKGRPRLTAWLEKVRTQANPHYDEAHKVLR
KISERTSAKL-

####### LLOJ002025 - GSTT ########
>JH689467 forward | **LLOJ002025** (pre-editing) | LLOJ002025 (post-editing)
TGCTATTTTAGGTACACACAGAGAGATTAATTATATTATCTCGTATTGGCTTTCAAGCGATAGCATAGCTCAATCTCCATCGCAGAAGATGTATTGAGTTGTTGTTGT
GCCACAAAAAGTCGACCTTTCTCACTCACTTTCAGCCAGCTCACGCTTGTCAATTTTTTCGTGTTGATTTCACTGAAATTTCTGCTCCAGAA**ATGGCCTCAAAATATAA**
GTTTTATTCCAATTTAATGTCCCAGCCGTGTCGTTCCTTGCAAATTGTTATGAATTTGGCTAAAATTCCCTTTGAAACTGTTACAATAGCATTGGGTGAGTTTTATGAAGC
TTTTTGGGTGTTATTTGTCCAGGAATTATGTAAGGTGTTTGTCTAATCTCTCGTTGTAG**GTGATCACGCCAAGGATTCCTTTGCCAAAGAGGTCAATAGTCTCTGCACAA**
TACCCTGCATCAATGATGGGGGCTTCAAACTAGCCGAGAGTATTGCCATTCTGAGGTATTTGGCAACCAAGAGTAGCCTCATTATACGATGGTACCCAACGGGGGCACGAAT
GAGGGCACGAGTTGATGAATACTTGGAGTGGCATCACCTCAACATCAGAGCTCCCTGCACGGGATACTTCCGCAAAAGTTGGCTCGAGCCTCGCAACACGAAGCAGCCACCA
AATCAAACGACCCTCGATAGCCTCATGGGTCAAGTTAATCGCTCCCTGGATTTCCTTGAGAACATCTGGCTAGCCGAAGGGGATTTCCTCATTGGCAGTGATGTCTCCGTGG
CAGATATTTGGGCTATTTGTGAAATTGAGCAGCTCTGTGAGTTTTTTGAATTTATTTTAACAAAAATAATTATTTTTTAAATTAATTTTCCTTTGCAG**TTTTAACACCTC**
TAGACCCCACAGAAGGACGCCCTAAGCTTAAGGCATGGATGGAGAGGGTACGAAGGGACACTAATCCCTTTTACGATGAAGCCCACTACACTCTATGGAGTGTTCGTGATAA
ATCCGGCCAGAGATCGCAGCTTTAAGATTTATTTTTCTTTATTAAATCTACTACAAAAGGGGGTACTCATAAACCATTCCAAGGGATATTTTAATACAGGACCAATACT
TATAAATTTGTTATTTTCTTTTTCAAGGAAAATTGTTAAATGTTTGCAAATAAAATATTATTAAATTGTGAAGAATGTTTTTGTTTTACTTGAAATTATTTAATTAAC
TAGGTCAG

>LLOJ002025 (227 aa; 3 exons) | UNCHANGED
MASKYKFYSNLMSQPCRSLQIVMNLAKIPFETVTIALGDHAKDSFAKEVNSLCTIPCINDGGFKLAESIAILRYLATKSSLIIRWYPTGARMRARVDEYLEWHHLNIR
APCTGYFRKSWLEPRNTKQPPNQTTLDSLMGQVNRSLDFLENIWLAEGDFLIGSDVSVADIWAICEIEQLFLTPLDPTEGRPKLKAWMERVRRDTNPFYDEAHYTLWS
VRDKSGQRSQL-

####### LLOJ008830 - GSTT #######
>JH690086 reverse | **LLOJ008830** (pre-editing) | LLOJ008830_a (post-editing)
GTAGCTCGCTAGGTAAGAAAATTTTATTTCATCAATGTTTCATGAAGCCAACGTTCTCAAAGTTTAAATATAGTTTGTGAGGAATATTTCTTCAGACTTGTCTACGAT
ATTCCTTGGAGCACAAAAACATTCCTTACTGGGTAGTGACATATACCACAACAACATAAATATAACAAAATTAAAAGATTTTGCAAAGAAAA**ATGTCTTACTCTTACGT**
TGAATTGGGTAAGAATTTATTAATTATTTAAAGAATTAAGAAATTCTACAAACATTAAATCCTTTCAG**ATTTTGACAATCCCTCAAAGTACACAATACCCCTGAATAAGT**
ATCGGAAGAGGACGCAGAAGAGCACGAAGGAGCAACACTACACCTATGTTTGCTTCCTGGAAAAGCATCCCAGACTCTGGGAACCTTCTCACAAGAAGTACGATCACATGTG
GAATAAATTGACAGAGATTCTAAATACGAAAGGTCCACGGAAGACTGTGGATCAATGGCAGACAAATCTCCGGGATTGGCGGTACTCGGTTACATTTAAGTACAGGAAGTAC
AAGAAACTTGGAAAGCCAATTGCCGATATGGAAATGAAGTTGTTGCAAATTCTCGGAGTACTTGAAGATGAAAAGTACAAGAGAAGATTGTCATCGTTTGAGCTCAGCTCAC
AGAATGGGCAGGAGTTCACTTTAGAAGATGATGAGATGTCTGGAGATCCTCTCCTTGAGGAAGTAGGGAACGATGAGGACACAGAAGCAAATGAGGAGAGCGCCATGGATGC
AACTGATAGTGCTGCTGATATATCTTCAGTGTGGTTTCCAGAGGTGAAGAAGGAAATCACCTTGGAGACTCCATCAGAGAGAGATATCTTCGAAGATGCACAACCAGCACAA
TCTGCTGATAGACTTCAACATGTACGATGGACAGATAGGAGCATTAGAGATCTCCTGAACTTTGTAAAGATGACACTTGAGCAGTTTGAGCAACCAAATCCACGAACATACT
ACCGCAACTACCTGCAACGTGAACCACCAATTGAGCAGTTTATTGGCGACAACTGGCAAGCTGTTCAGTTGAAGATGAACTCGTTCCGACAGCAGTACATGCAGACACAGTC
GTTGATCAAACAGAAATTCGGAATCACAGCAACATACAACAACTTATCGCAAGGATCGAGAGGTAGAAAAATTCAAATAATTAAAGATTTTCAACAAAGCTAACGATTTC
TTTCAAATATTTCAG**TACAAATCCTAACTAAATGTCCCTACTTTTTCGACTTGGACAAAATATTCGGCAAGAAGGATCAACCTGCGCAGGAAACCAGACAAGGAGACAGAG**
GGAAACTTCCGGGAGATGAATTTAAGCTCTCCCTGCCATCACAGATAACCGTTCGCAATATCAAACCAAAACAGCACACAAATCCAACTCCAGGACCATCTAGAACTCCACC
AAGCTCAACTTCACAGCCAAATGCAACAAAAGATCTCCAGAGAACACTAGAACTGGAGCGACTTGACATTGAAATTCGACGTTTTGAACTAGAGAAGAAACGCTACGAGCAG
GAGAAGGAAATGGAGCGTCAGAAATTCCTCTTTGAATGCGAAATGAGGAAGGAAGAGATGAAATTGCGAAAGAAGGAATTGGAGAATAAACGACGTGAAGTTGAGAATATGA
TGCAGATTGAGAATCAACGCCTGGAGCAGGAAGAACGAATTGAACGGTACAGGATTCAGATGGAAACTGTTTGAAAGAAATAAAAACAAGAACTTTAAAAACCCACCGCC
AGAGGGCGGACGTTTTATTATTAGTTTTGTGGAAGAAAAAGGTAAACAAGTTTGAGAAAATTTTCGTGAATTTTGTAG**TGGAAAATCATGAAGAAGCAGGAGGATAATT**
TGGATTCTGATGAGAGTTTCGATGAGGCCATGAGGTGAGTAATCAGTTTTTGTTAAAGAATCATTAATCTACTGAGGATTTATTGCTTTTTTTAG**TCCTGAAAGTTTTGA**
CAATTCTTCCTCATGTGAATCGGGGGAAGATGCTGAACCACCCGCAAAAGTGGCGAAAGTTCAAATGGCAAAACCTCAATTGAGTGACATGAAGAAGTTTCTGAAGTACCTC
AAGGATTATCCTTATGCATCAGAAGGATCTGAAAACAAGGAGGCCTCATATGAAGCATACTACACGAGATTTCTGGAAACTTATCCGGATATTAATGACACCTGGCAGGTTC
TTCATCGTAGGGTTACAGGTATGCGGCATCAGTATGACCTTACAAAGAAGTGGCTACAGGAGAATGCGGCGGATTTTGATGAGAATGTCATTGAGAAAGGTAAGAATATAT
ATATTTTTTGCATTCAGGTAAAGTTCTACAGTTATGAGCAAAATTGTCCTTACTTTAAAAGAATAATCTCCTTAAGAAATATTTCTAAAATAAACCTACTGAAATTTC
AAGATTTTAAGTAAATTTCAAGGATTTCTTGGTCGCTGAATAAATGGATGAAATGTTGGAATGACGTCCAGCCCAAGTAAATTAATCCCTTCGCACCTGCTTGAAACA
TAGAAACATTGAAACATGCGATCATTCTATCGCGATATTTATTCTGAAAATGCTCCCAAAGGATATTTCGAAGTCGAAACGTCTTCTTTAGCTCTTTTTACGTAGTCT
TAGCGTATTTCCAACTTAAAAAATATATTTAATTAATTAAAATTCGATAAAATAAAATGTTTTACGTTTGAGTCAGCGCATGGACGCAAAAGGGTTAAAAAAAGAAGA
ACACGCTGACCTAAAATGAAACATTCCCTTTTTTTTTAAATTACTAATGAAAATTGAAATTTCTGAAGAGTAGAATTGCGTTGAATTTATGAAAAAGAGATCAGAGAA
AACATTTTCACAGAAAACGATTTTCTAAATGAAATAATAGAATAGAAATCCCAATAAAAGGCTCAATGTTTTAATGTTATTTCCTTAATGTTTCATGCCTGATTTGCA
AGGGTCGTAAGGGTTAACGAATATAATTTATTCAACTTATTCAGTGAGAAATTAAGGGGGGTCTCTTGGGAAAATTGTTAGAATTTACATAATTTTTAATGATGTAAA
TTTCGGGGTTTTCTTAAACATGGTTTTCCGATGTAGGTTACATTTGAAGCGTAATTATTGAAGTGTAAGAAAATCACTTTTCGCCATCTTAGGTGCGACGCCATCTTG
TGATTTTCCGACTTTTCCATAGAACTGCCCTAAAACATAAAGGTAGGTTTTTCTCAGGATCTACCGGATGGATTTTGATGGGGTAAAAAGCAAATGAAAGAGGAAATA
CCAATGCAGATTNNNNNNNNNNNNNNNNNNNNNNNNNNNNNNNNNNNNNNNNNNNNNNNNNNATATTTCGAAGTCGAAACGTCTTCTTTAGCTCTTTTTACGTAGTCT
TAGCGTATTTCCAACTTAAAAAATATATTTAATTAATTAAAATTCGATAAAATAAAATGTTATACATTTGAGTCAGCGCATGGACGCAAAAGGGTTAAAAATAAGAAG
AACACGCTGACCTAAAATGAAACATTCTCTTTTTTTTTAAATTACTAATGAAAATTGAAATTTCTGAAGAGTAGAATTGCGTTGAATTTATGAAAAGGAGATCAGAAA
AAACATTTTCACAGAAAAAGATTTTCTAAATGAAATAATAGAATAGAAATCCCAATAAAAGGCTCAATGTTTTAATGTTATTTCCTTAATGTTTCATGCCTGATTTGT
AAGGGTGGGTTAATAAATATAATTTATTCAACTTATTCAGTGAGAAATTAAGTTTTTTTGGATTTTCTTGGTTCTGATCGCGTGAAAAAAATTTGTGAGTCGTTAAAC
GGTTAAGAGATGAAGGTGAAAACTCTCTTAACACTGATAACGATATCAGACACTTCAAATATAATACAGATTAAACAGACGAAAAGAATTATATCTCAAACTTCTCAT

TCGCTTCAATTTGTCTTTAGTGGTTTACATCAATTTTTGTGTTTTCTGTGCTTTTAATTCAACACGAAAATGTCCAAAAATATAAAACTTTATTCAAATGTTATGTCC
CAGCCATCAAGATCTCTCCATATTCTCTTGAATATGGNNNNNNNNNNNNNNNNNNNNNNNNNNNNNNNNNNNNNNNNNNNNNNNNNNTTGGAAACTTATCCGGATATT
AATGACACCTGGCAGGTTCTTCATCGTAGGGTTACAG**GTATGCGGCATCAGTATGACCTTACAAAGAAGTGGCTACAGGAGAATGCGGCGGATTTTGATGAGAATGTCATT**
GAGAAAGGTAAGAATATTTTTTGCAATCAGGTAAAGTTCTACAGTTATGAGCAAAATTGTCAAAAACTTGTGAACAAAAGTCACATTTTTCCACTTTTCTCAGCCCCAG
AGTGGAAATTCAAAATTGTGTTTGAGGAAAATCTCAAGATGGCGTCGCATCTAAGATGGCGGAAAGTGATTTTCTTACTCTTCAATAATTAGGCTTCAAATGTAACC
AATATTGAAAAACCAAGTTTAAGAAAAACCCCAAGAAAAATAAAAATATTTAATTTCACTAGCTCTCACAAGAGATCCCCCTTAAGTTTTTTTGGCTTTTCTTGGTTC
TGATCGCGTGAAAAAAATTTGTGAGTCGTTAAACGGTTAAGAGATGAAGGTGAAAACTCTCTTATCACTGATAACGATATCAGACACTTCAAATATAATACAGATTAA
ACTGACGAAAAGAATTATATCTCAAACTTCTCATTCGCTTCAATTTGTCTTTAGTGGTTTACAGCAATTTTTGTGTTTTCTGTGCTTTTAATTCAACACGAAAATGTC
CAAAAATATAAAACTTTATTCAAATGTTATGTCCCAGCCATCAAGATCTCTCCATATTCTCTTGAATATGGCAG**AAATTCCATACGATCCAGTAACAATTGCCTTGAGA**
GAAGGTAAGTTGTAATGAAGTTGTAATAAAGGTAAGTTGTGTTACCCTAAATGAAATTGAGAATAAAGGATTTATGGAACTTTTCCAG**GTGATCAATTCACAGAATCTT**
TCTCAGACGAGGTAAATAAACTCAGAACAATTCCTTGCATTAATGATGAAGGCTTCAAAGTGGCAGGAAGTGTTACAATTGTGAGATATTTAGCAGAGAAAACTGATGAAAT
TACGCAATGGTATCCGGTGGATGCTCAAAAGCGAGCACGCGTTGATGAATATCTGGAATGGCATCATCTGAATACGCGAACACCCCTCTCTGGATACTTTGTGGCAAGCTGG
CTTATACCACGAAAGACAAGGAAGCCACCAAATGCAAAAAAGATGGAAAAACTTCGCAATGAAGTTAATAAATCTCTGGACTTGTTGGAAAATTTATGGCTCCGCAGTGGGG
ATTTCTTAATTGATAACCAATTGACCATTGCAGATATTTGGGCTGTATGCGAGATTGAGCAACTCTGTAAGATTTCTCAGATCTTTAATAACTATTTTATTAAATAATGA
TTTTTTTGCATTCTTTAACGTTCTTTTTCAGCACTAACACCGTTGGATGTTATGAAAGAACGTCCCAAACTCAAAGCTTGGATGGAGAGAGTGAGAAATGCAACAAAT
CCCTTCTACGACAATGCTCACAAGACTTTATGGAGTTTTAGTCATAGAAATGAGCAAAAATCCCATTTGTAAAATTCATGAAGGTGCAATAAAATTATTTTGAACAGG
TTTTCTTCTTTTTGTGTTAAAAACATTTCGTAATGCCCTCAGAAGACATTCTTCGATCAAAATGTATTTCTTGGCATCTTGTATGAAAATTGAGCGAATCAAAATAAA
ATGTTTCCCGTTTTGATCAGCTTATGACCTAATGGGCGTGAAAGGGTTAACCTCATAATACATATGTATTATGTAAAAAAAACAGCTTTTTTTTTTAAAGCTTTGCAA
TTTCACGAAATTTTAAAATCTAAAGGAAATTTTGCTCTCAAATGAACATAAGAAGGAATTGGAAAGAAATATTCTAATAAAATTACACATAGCTCATAATTGAAAGAT
AATAAATTAAAATTAATCACAAAATGTGCCTACATTGTAATTTCCAG**AG**GTTAAACTCCGATGTCCGTTTTTCTATGAATTGGATGAGGTATTCAGGGATAAAAACTT
CCCAGGCCCGGAGATGGAAGAAGGAACGAACGTACCAAAGGCAGATGATTCAGTGCGAGAAAATAGGCCGAAGGTGACGCAATTTCCTACGCTATTTCCGACCCGGAT
GGATGATATAGATAGCAGGAGGTTGCAACTCGA

>LLOJ008830_a (229 aa; 3 exons) | EDITED
MSKNIKLYSNVMSQPSRSLHILLNMAEIPYDPVTIALREGDQFTESFSDEVNKLRTIPCINDEGFKVAGSVTIVRYLAEKTDEITQWYPVDAQKRARVDEYLEWHHLN
TRTPLSGYFVASWLIPRKTRKPPNAKKMEKLRNEVNKSLDLLENLWLRSGDFLIDNQLTIADIWAVCEIEQLSLTPLDVMKERPKLKAWMERVRNATNPFYDNAHKTL
WSFSHRNEQKSHL-

####### LLOJ002682 - GSTT #######

>JH691041 forward | **LLOJ002682** (pre-editing) | LLOJ002682_a (post-editing)
AAGATAATTCTGTGAAAATAAATTGAGATTTTAATTAATTAATTAAGCTTTTCAAACGACAAAATTAATCATCAAGTTCGTCATCTTATTAATAGCTTTCAGGTGTGT
GATAGTGGTGATAGTGCACCTGTTTATCAACACAGCCAAAAGCAACAGGGTTCAAAGCTCATTTAGCCGGAAAAATCGCGAATTTTCCAGAA**ATGTCGGGGGTTGCTTT**
CAAATTTTACTACGACCTCCTCTCTCCACCTTCCCGGGCACTCATAACATTCTTCCGTGTTGCAAATATTCCCGTGGAACCCATTGCTGTAGCCCTCCGGAAAGGTAAGTCA
TGCACCCACAGAGCTTGGGCATCACTGAACATAAATCTTGCATTTCAG**GAGAACATCTCACGGAGAAGTACAACAAGGAAGTGAGTAGATTCCCAAAGCTTCCGTGCATC**
AATGATGACGGGTTCCGGTTGTCTGAGAGTGTAGCCATAGTGCGGTATGTCAAGAAGACAAGAGGATTTGATGATTTCTGGTATCCAGAGGATGCCCAGGCACAGGCACTTG
TGGATGAGTACCTGGAGTGGACACACACAAACATTCGCATAAGTTCCGGATTGTACTTCTTCACATTGTGGCGGAATCCCTTGATGACGGGGGAGAAGCCGGATGAGGCAGA
AGTGCGGAAGTTGGAGGCTCAGCTGAACAACACATTGGACATCATGGAGAATACCTGGTTGAAATGCAATTCCTACATTGCCGGAGAACAGTTTACAATTGCTGATGTTTTT
GCAGCATGCGACATTGAGCAACCGAGTGAGTACATAATAATTTATGTTGCTGTGTTGAGAGGCTGAGAGACTCTGTTGCATGCTCTCTTGCGAATAATTTTAGCAGCATT
CAAGGTTTTATTTTGAGATTTTCTGATTGTTTGATCTCGGTTTTATGAATTTGCATAAAAGGATTTTATTAATTTTTCTTCTAAAAAATCTGAAAAAAATTAAAGTAA
AAAAAATTCTTCCAGGAATTTGTGGCTTCAATCCCCTTGATTCCCGACCAAAGCTCACAGCATGGTTTGACCGTGTGAAGAAGAAGCTCGATCCTGCTTTCACAGAAC
ATCACAAATTTATCTACAAATACGGCCAACGGTCTCTGGAAGCCGCCAAACTCTGAGAATGAGGAAGACCTTCTGCGCCGACGTGCCATCCACACGTTCCACAGCAGG
TGCTTCTGATGTTGTCATACTCATGACCAAAAATTTTGTGGTGCAGGATAAAATAAAAAGAGAAAAGGGGCTTCTTTGAATAAAATTCCTTAGGGTACAGTGCACTTC
CTTTCTTTTCTGTGATCCCCCAAAATAGGATAG**GATCGTCAAATAATGGAGCAGAGCAGTGGTGGAAGAGTTAAGTTTCTCGACGAGCATATGGAGAACTACAACATTGAA**
GTGAGTTCATTGAGGTCACTGCTGGAAGGATTCTCAATTTATTTGATGAGATTTCCATGATTTTGCAG**CATTCTGCCCTCCATCGGAAGCTCCAGAGCAAAGTTGAGGCA**
TTGAAGATTATGCGAAAGGAGCTGGAGCGTTTTCGCACGGAGAGGGATCAGTTCAAATTGATGGCGGAAACACTGCAAATCCGCTACACAGCCATCAAGAAGAGTCTAAATT
CCGGCGAATCCGGAACCGTTGAGTTCACTGAGAGATCCAGCGTGGCAGCTATTGTGAATGACCTGCGTGAGAGGAATACAGCTCTGACGACGGAACTCGAGTCCCTGAGGCA
GAGTTTGTCAGAGAAGGAGGGGGATATTAGGGTGCTGCGAGAGCGGAAGTGCAGGGTGTGCGAAAAGTGTCAGAATGGTAGTCACAGTGGGCAGGAGGATTCACCTCGTGCC
GAATGGAATGAAGAGAAATCCCAATTCATTGCTCAACTGGAGGATCTCAAGAAAAAAGTAAGAAAATTGAAAGGAAAAATTCTTAAATTGATTTTTATTTCAAACTTGAT
GGAATGTCCAATTTTTCTTTCCCAG**AACGCTCAGCTGAAATACGATTTCCGGTCACTGCTTGATGAGAAGGAGGAAGTTGTCACTGAAAGAGATGCCTTCAAGTGCAAGGT**
TCATCGACTGAATCATGAGCTGTCGGTGGCTCTCAAGGGGAATGAATCTCAAATCAAGGTTCTCGATATTGATTCACTTGTGCGGGAGAACAAATATTTGCAGGAGAGACTG
AAAAACGTGGAAAATGAGTTGGAGCTCGTGCAGCACTCGCTCAACAAATATAAGGTAAGATGTTCAATTTCCTCCTCATTTCCTTGCAATTCTCTAAGAAAACTCCAGGA
TTTCCCTTCCAAACACATGCGCTCATTGATGTTAAAAGTGGCACAAGGGGGTACTTTGGGAGCTCTGTCTGAGCTTTTCCAACGTATTTTTGCAATATATTTATCAAT
CGTTTTTCATTGTAATAG**ATTGTAAATTAA**TTGAAACACTTTTTTATGATTTTAAAAAATCAATTTTCATCAGTTTTTTTTGGGGGAACATCAAAATTGATTTTAAAAT
GTTTGGTTTATGGATCAAAACTTAATTTATATTTAGGAAGAAAAAGGTTTCCCAATAGTTTCACAAAAAAAGCAATTTTTTGGGAGGGACGGGGGTAATGTTTTCAT
AAATAATAATTAA

>LLOJ002682_a (230 aa; 3 exons) | EDITED
MSGVAFKFYYDLLSPPSRALITFFRVANIPVEPIAVALRKGEHLTEKYNKEVSRFPKLPCINDDGFRLSESVAIVRYVKKTRGFDDFWYPEDAQAQALVDEYLEWTHT
NIRISSGLYFFTLWRNPLMTGEKPDEAEVRKLEAQLNNTLDIMENTWLKCNSYIAGEQFTIADVFAACDIEQPRICGFNPLDSRPKLTAWFDRVKKKLDPAFTEHHKF
IYKYGQRSLEAAKL-

######################
####### GST Xi #######
######################

####### PPAI008305 - GSTX #######

>JH665757.1 forward | **PPAI008305** (pre-editing) | PPAI008305_a (post-editing)
CACAACCATCCAATTTTGATAAATCACAATCCTGATAACAGTCATTTGTTTATGCTGTCTCATGTATAGGTCCATAATGGTAAGCTTACGATGATAATGATATGAAGT
GATAATACTGATAAGAGTTTCAGTATTCAAAAAGAAATGTTGCGCTCAGTCTCTTGCGGATCTTTGTGAAAAGCAATTGTGAAACTAACAA**ATGGCACCTTTGAAACT**
GTACTACTATCCCCCGTGTCCTCCCTGCAGGGCAGTTCTTTTTGCCGTGGAAAATCTAAAAATCGAGGTTCAGAAGATTTTGGTGGATCTTTCTAAAGGAGATCAGTTGAAA
CCTGAGTTCCTGAAGATCAATCCTCAGCACTGCTTGCCCACAATTGATGATGATGGATTCATTATGTGGGAATCCAGGGCAATTTTGGCATACTTGGTCAACTCACGAGCAC
CTGGAAGTTCCTTCTACCCACTGGACCCCAAGAAACGAGCCTTAGTAGACTCAAGGTTGAGCCTGGATTCTAGCATAATGCATGCGCTCGGAAATATTATTGTAAGTTTTTA
GAGTTTTTGGAGATTGAGAATTGGATTACGACAACTTAAAGCCATGACGAAGTATGTGCTAAGAACTGTGATTTTGATAATTAGAAATAATAAATCGTTTACTTTCAG
ATTTGTCGTTGTAGCAAGTATCTTTATAGTGCAACTCATGACACTGTCTTACTTCTTGAATGGAAGTCTTCTATTTAGAAT

>PPAI008305_a (>114 aa; >1 exon) | EDITED (PARTIAL - C-TERMINAL MISSING)
MAPLKLYYYPPCPPCRAVLFAVENLKIEVQKILVDLSKGDQLKPEFLKINPQHCLPTIDDDGFIMWESRAILAYLVNSRAPGSSFYPLDPKKRALVDSRLSLDSSIMH
ALGNII

[blastx found an odorant receptor gene just downstream of the gene, but no GST.]

####### PPAI010868 - GSTX #######

>JH660956.1 reverse | **PPAI010868** (pre-editing) | PPAI010868 (post-editing)
AGTCTTGGGTGAGGCGTCAACCAACCTTTATCACAAAAAGTATCACATACCGGTTCCATTCGGTGATTTTTAATTGAAAGTGAGTTGGAGTATTTCTATCTTTAGAAC
TATTCTTGCGGTTTCAAGGTTTGTTTTTTGTGATTTTGAGATTATTTTGTGATTTTTTTTCTCCAAACACCATACAGTAACATCTCAAAACA**ATGGCTCCAGTGAAGTT**
GTACCATTTTCCCATCAGTGCTCCCTCCCGCGGAGCCCTTCTGGCCATACGGAACTTGAATCTCGATGTGGAGGTAGGTTTTGGTGCACAAAATCAAATGTATTTGTGAGT
AAATTAAGGAGAAATTGAGAGATTTGCATGTTAGTGAAAGTTTTTTGACCGGCAATTGAGTGGGTTTACAACTGGATCAGTGAATGTATCATTATGCGTTGAAAATCA
AATGATTCAATTCTAGCTGCTTTAATGAAAATACGGTATCTAAATAATTTAAATACGGTTTAATAGGTTTGACAAGAGTACTTAAAAATATTTATTCAAAGATTGCTA
ATGTCTTATGCAAACTCTTTAGAAATCACTTGTAACAGGACAAGAAATAATGTATTATCAAATTATTTTCTTATATAGTTATATTATTATAATCTAATCCGTATTTTA
AAAATTTATCGAAATAAAGAGTTTGCGTATCTATCTCTTGGAATCTAATAATAATTTATTAATAGTCTTAACAATAAAAAAACATTAACCATGAATATTGAATTAATA
AGGCAAAACTATGATCCCAACAAAAACTATACAAAGCCTTCATACAAGCAAATTTATCGATTATACATATAGACGTTTCTAAAGATTGTCTAAGTGAGAGATCTTTTG
TTGTACAGGTTCAAGGGTAATACGGAAGGAATCTGCTGTTTATCTCTTCGGCAATTCGAAACCTTGAATACATTTATTATAAAAAAAATTAAGGCCTATTTAATTAAA
AAAAACCTTTGGATAACATACGATTTTTAAATAATAACGTCACGCTGATTTCTCATTCTTACACTGTCCACGGTCAATAGAACTAGAACTTTGGCTTTTCAGAGTATC
CCCCTTAAAACGACCTTTGCGTCCCTAATATGACCCATATCTCTGCTTCTCTTCCAAATACGAGATTATTTATTATAACAAGTGTGAAATTATTGGAAAAATCTTTTG
GGGAAATCAGCTTCGGACGACTCATTTTTTAAAATATGTTTTTAATTAATTTGACCCATTCATTATATTTTAATAAGGCTGATGCCTAAAATTTCTCGAAAATCCATT
AAGAACATTAGGAAAAAAGTTTTACGAAGCTTAAATCATCCGAAGGTACCGCTTTTACCCTTAGTACTTTGTCCTTTTATTTCTATGAGATTTTTACCAATCTTTGAA
GTAAACTAAGAATTGATTCAAATTGGTTGTATGCCAATAAACAAGTTTCTTCTTCGGAACAATGTTTCTAACTCCTGTACTAAAAATTTCGAGCCGTTTAAGCTTATG
TGACCGAAACGTTTCCATCCTTGTTCATGTGGCAGTAAATAACTAAATTAAAAAATATTTAAAATAAACATAAAAACTAGAATAAAAGTGATCCATGGCTTATGGCCT
AGACACACTTACGACTTAAACCGAGAGACGGCTTAGTGGAAAATGATAGAAACGCGGTTTAACCATTATTTTAAATATAATTACGCTAAGTCGTCTCTCGGCTAATCC
TCAAGTCTGTCGAGGCCCTTGCCCGAATATCTAGCTATGCATGAGCATTTATAATTTTTCTATTTTAACTTTGGAAAAAACCTTAAGAACGATATAGGGGAAAGCTTT
CAGGGTTCGTATACAATACTTTATGTTTTTTTTCATATTCCCTTAATATACCTTACCCATTTTTTCAGGAAATGTNNNNNNNNNNATTTCGAAAATTAAGAAAAGTGT
TAATTACATTATAAACTAATAATTTTATAACTTTTCGAAATCTGTTTTACGTAAAAGCTCAAGATTTTTTCACATTGTCCATAAGTGTATAGCATCCATAAGTGTAGA
CTCCACCCTACACCACTTTTATTTCTTTACATTTTTTGCTATTTTGTATAAAAAATTATGCATTTTTCAACAGTTTTCGATAAGAATCTTATTTTGGATAACATAAGG
AACATAAATATTTGTATCTCAACAAAGAAAAAAATAATTTGAGAAGTCGTTGAGCTGTTTCAAACTTGAAAGTGCTAAAATGTGTCCCGAAATACCACACGTTACCCT
AGCTATTAAAATATATTGCCATAGATACATATTATGTCAGATTCATTAATGAAATATGAGAAGAATATGGAGTATTCGTTGCCAGAGTGTGTGCGAAGCTTGAAAGCC
TTCTAGTCCAAAATTTTGTGAATTTGTCAACTATAATAATACAAACAACTTACAAATTACAAATTTGTCATTATTAAATGGATTTTTCACAAAAAGTTTCGAACAGAC
TTGAAACTTCCAACACTTCGTTTATGTATTAATTTTGAATGCAATGTTTAGACAAAAATAGTCGAGCCTTTAAGAATGAATATTGATTAGGAGCAAATCTTTTAGTTT
ACTGTGACTAACATTAAATTTTAATTTATGATCTGGTATTTAATATCTTGGCATCTTCTGTGTTTGAGATTAGCATTTAAATCTTATTTTCTGGTTTAG**ATCATCGAGG**
TCGACCTTATGAACAAAGCTCAATTGTCTCCTGAGTTCGTCAAAATCAATCCTCAACATACCGTTCCCACTCTCGATGACAACGGCTTTGTTGTCTGGGAATCTCGAGCTAT
TGCTACCTATTTGGTGAATTCCAGGGCTCCAGGGAGCTCTCTTTACCCGGATGATCCCAAAGTCAGGGCAGTTGTGGACTCTCGATTGTACTTTGATGCTTCAAACTTATTC
CCAAAGGCTCGAAACATTGTCGTAAGTTTACGGTTTTGGGATTTTGGTATTCGGATTTATAGCTCAATTCTTTTTTAG**TTTCCTATTCTTTTCCTGGGAGTCAAGGAAGT**
CAAAGAGGACTTGAAACAGACCCTATATCAAGCTCTTGATTTCATGAATACATTCTTGGAAGGGCAAGATTGGTTTGCTGGAGACAAGCCAACTGTCGCTGATTTAGCCCTC
TTGTCCTCTTTCTCAACTATTGTTGTAAGTATAGTACTGTAGATTCTCCATCGTAAATTCGTTAGAAGCCTTCGTAAATTCCACAAAAAGCGCTCAATTATAAAGAATC
ACGATAAAATAGGAAGAACTACAGCGAATTTGAGCAAATTAGCTTCATTATTAAACATGAAAATTGTCAACAAAATTTTTCGCCTGATTGAAAAAGAGCCGATTGAGC
GAGAGTCTACTGTTTTGAAAAATTTTTGGCAGGGTTAATGTTAATAACTTTGATTTTTCAATAG**CATGCTGGAGCAAATGTGAGCAAGTACTCAAACTTGAATGCTTGGT**
ACAAGCGGTGTGAATCTCTGCCAGGATTCGATGAAAACGAGGCTGGTGCCAAAACCTTTGGAAAAATGGTTAAAACAAATCTTGGCATTACAGGCACATGGGATTAAAGACA
ACGTGTGATTTGGATTTGGAATTCTTCGTTGCACGTGTTTTTTGTGTGGTTGTTTCACAATTTCTTGAAATAAAATCGATTAATCGATGAATTTTTGAGTGTTGGTTT
CAATATTATTCGATAAATTGATGTTTTAGTAGATTAGTATGTTTAGATGCTGGATAACGTTTTCGATGCGCTATGGTTTTTCAGGTT

>PPAI010868 (221 aa; 4 exons) | UNCHANGED
MAPVKLYHFPISAPSRGALLAIRNLNLDVEIIEVDLMNKAQLSPEFVKINPQHTVPTLDDNGFVVWESRAIATYLVNSRAPGSSLYPDDPKVRAVVDSRLYFDASNLF
PKARNIVFPILFLGVKEVKEDLKQTLYQALDFMNTFLEGQDWFAGDKPTVADLALLSSFSTIVHAGANVSKYSNLNAWYKRCESLPGFDENEAGAKTFGKMVKTNLGI
TGTWD-

####### PPAI010869, *PPAI010870* - GSTX #######

>JH660956.1 forward | **PPAI010869**, ***PPAI010870*** (pre-editing) | PPAI010869, PPAI010870_a, PPAI010870_b,
PPAI010870_c (post-editing)

GGAACAAGTTTAAAGACGTGAACAAAAAACAACATTGACTAAAATGCTTTTTCATTCCTGTCCCTATTGATTAGTTTAAAATTACTAAAGTCATTCTTGGTCACACGA
AAATCTGTATAAAAGACTGTAAATACTCATGTTGCAATTCAGTACCAATTTGTTCTCTGATAAGATAGACCTTTATTTTAAAAAATATTGAT**ATGGCTCCCTTAAAATT**
GTACCACTTCCCAGTGAGCCCTCCATCCCGTATTGCTGTCCTAGTAGTTAGGAACCTACATCTAGACGTGGAAATTATAACAGTTGACCTAATGAATAGGGGTCAATTGACC
CCAGAATTCTTAAAAATTAACCCTCAGCATACCGTGCCAACAATTGAGGATAATGGATTTATTTTATGGGAATCCCGTGCCATTGCTTCGTACTTAGTTTCGGCTAAAGCTC
CTGGAAGTTCATTGTATCCGACAGACCCTAAGAAAAGAGCTATTGTGGATGCAAGATTATATCTGGATCAAGCTCTGCAACTTGCACTGACAGCAATTGTCGTAAGACGAT
AAAGTTTATATCTTAAAAAGTGTTTATGTGTATGCAATATGTAAAAATCTGTAATGTTTCAG**TTTCCAATTCATGCTCATGGTGCCACAACCATTGAAAAAGATAAAAAG**
GACAAAGCTTATCAGATTCTCGAGAATCTGAACACTTTTATGGAAGGAAAACCATACGCTGCTGGAAACGAATTAACAATTGCCGATTTGGCTCTTTTAGCCACAATAACAA
GCTTTTATGTGAGTTGAAGTGTTTATAAATGTTTATGTATAAAGTGTATAAATCTAATTTAATTTACTGTATTTAG**GAAATGGGAGCCAATATTCCGAAATTCAAGAATA**
TTACATCTTGGTACAAAAGACTAGAGTCTATCCCTGGCTTTAAAGAGAATGGTGAAGGTGCTAAAGCTCTAGGAGAGTATGTAAAATCGAAAGTTACGGTTACCGGAACTTG
GGATGATTAATGCGATATCCTTTTATTATAAAGTAAATTTTAACAGAAATCATATTTTGTCAAATAAAAAAGGGGCAAAGTGATTTTGCATGTTTTTCGTTATCTCAGA
AAAATTAAAGAGGCCTGATAGGCAAGTGTGTAAATCCCATTTCATTTTATCTTTCCTTAGATTGCGTTTATCATTCTGCTATAGTGAAAAGTCATTCTACGATGCTGC
AAAGCAAACAAAATTTGTCATTATTTTAGTTTGTTGTAAACTGTTGAGTTAGGTGGTTCTTTTCCGTAAAATTTTACTAAATATCTTGCAAA***ATGGCACCTATTAAATT***
CTATCACAATTTCTTGAGTGGACCTAGTCGAGGTGTTCTCCTGACTATTCGAAGCCTTAACTTGGACGTAGAAATAGAAACATTGGATCTACTAAAACAAGCTCAATTGGCT
CCTGAATTCGTTAAAATCAATCCTCAACACACGGTTCCAACCATTGATGATAATGGATTTGTTTTGTGGGAATCACGTGCAATCGTTTCGTATTTGGTTTCAGCCAAAGCCC
CTGGAAGCTCCCTGTATCCAACGGATGTCAAAAAGAGAGCTCTTGTTGATGCAAGATTATACCTAGACCAGAGTCTACAACTTGTAATGAGTTCAGCTGTGGTAAATAGAC
CAATAATTCTCTTGTTTTAACATTTGTACTTTACCCATTTGAAATACTAAAAGAACTAAATTTTAG***TATGTCATATTCACTGGAGAGGCTACAAAAATTCCGGTCGATAA***
GAAGCAGAAACTCTTCCAGATTTTAGAGCATTTCAATACATTTTTGGAGGGAAAGAAATATGCTGCCGGTGATGAGTTAACAGTAGCTGACTTAGCTCTTTTGGGTACCATC
TCAACTCTCTACGTAAGTTTATCAATCAGCTGAGGTTTTACATATGGCTAACATTCTTATCATTTTATCACTAATGAGCTGACAAGGATTACTAAATTTTGCAAGCGCA
TGATATTTGTCTAAGATTGATGGAAATCTATCCTCCTTATTCCGATGTACAGATAATCTTAATATTTAACCATATTTCTGTAACTTCATATATTTTACAAGTTAATTT
TTAAAAAAAAATACATTCAAAGTTTTTTTTTATAAATCTCCTTCACTAACTTTTCAATTATTATAATGTTTATGTTAAGTTTACAATCTTATCAAAAGGGCTTTAAAA
AATTAACGCTGAAAAAAAAGAAAGGAAGTAAATGATGCTTTAAATATTCATGTCAAAATTAAGAGTTTGAAAGAATGGAGCAGTTTCACGTATGTATAACTTCAAAAA
AATATTCTTGTAATATAATTAAATATCTAGAAATTGTGCTATACGTTTCCAAGTAATCTAATATCAAGAGAAGCTAATATGGTAATACTTAGTTTTGATACTTATAGT
TAAATTTTATTTATCAAAATTATAGTCAGAAAAATCTTTACCTTCGTTATAACGTTTATAAAAATGGCTTCGTGAAACTACCCAATTCTCCCATATAAATTTAATCGC
CTCTTTGTACAGAACTTTTTAAAACTTCTTAACAAAGCAAAATAAGTTAATAATTCTTTAATTTTTTACTTGTAG***GAACTTGGAGCAAATGTTAGTAAATACACGAACA***
TCGATGCTTGGTACAAAAGGCTAGAGTCCATTCCAGGTCATCATGAAAACCTTGAAGGTGCAAAGGCTCTGGCACAGTACATAATAACGAAACCCAATTTTAGTTGGGATG
ATTAATGTTATGTATTTCCGGAAAGATAATAAATGATTTTCCAGAACAATGAAAGGGGTAGCGATGGTTATGATATGGATTACAAATTAAATTCTCATCAACAATATA
TGATAATAAATTATGTTTATTAGTAATTTGGAGATCTATGCCAATATTTCACGATTTTATTTTATAATATGAGTCAATTGATAAAGCACTAGCTCTACAATGCAAATA
TCTCAAGTTCGAATCCCCTACAAGACACTAGAAATTTTCCAAAATTAAAAAGGTCGTATCGCACTCAGTCACTAAACTTAATTCAACTGAGATTGAGATTGTGAAATT
CATCCATACTTATCTAGAAATCTAACTTGTGAAAAGCATAATAATATTTATTAACGATTTTAATGTGCAAAGCCGAAGACACGGAAAATATTGAGCATATTTCAGTAA
AACTAATTTTCTGTCAAAAATAATACTCTTAATGTGTTAGTTTTTCGTTAATGTCTCTCATAGCTCTAAAAAGGTCATTTTACTGCTCGTATTCCACAGAAAGAGCAA
TGTGTAGATTCCCTCAATTTTTCACGCCCCAAAATTTCTACCTTTCATCCCCTGGGGACCGCTTTTCTCAACTTTTCTTCGCATTCTAGACTATGTCTGAGAAATACG
TCATACTGTTTGTCTGTCCGTACGCCAATCTGTCCTTCCGACCGTTACCACGCCTATGGATCAAACAATTCAAGATAGATACTTGGGATCTTCGAAAGACCCCTCCAT
AAATTGATCCTGGGGTCAATACCGTTAAATCGTTCTCGATAACGGTTATTAAATGTCAAATGTCGTTTTATGTTTTTCAACATAATATTTAGTATCATATTTTGGGCT
CGTTGGAAAGCTTTTAAAATTTCTAACATGATTGAAGGAGTGCCAATCCCAATCAACATTTTCTTTCCGATTGGAAAGCTGCCCAAATTTCGTCGACACAGTGTCGAC
AGATATTGGACAGATAATTTTCCAAGCAATTGGAAAAATTTCTGTCCAAAAATTTACCAAATGGACAGAAATTGGACAATTATTGGAAATTTTTCTTTCGAAAAAATT
TCGACTGAGTCTTTCGAGATTTTGTCGACAAAAAATCGACAAAATTCTTACCATGTTCGTTCGATTCTTCTTCTTCTTTGCTTTTCTGTTCAGTTTCTTAGTGCAATG
TGCAAGCAATCAGGTATCATAGATCTTTTTACCTTTGCTTCACACTTTGAAATAAAAATGAAGAACCAATGTCGCGGAAATTCTGAACGTTTACGGATTCCAAACCCG
TTTCGTTTTATTTTGTCTGTTGGAGTTTACAAAAGTTTTAATGACAGGTCAATTTAGGTGAAAAATAAGTATTAGAAATGCTTAATATCATATTTTCTCTACCGCGGA
TTAAAAATCTGTTAAAATACTACAGTAGACTCTCGCTCAAGCGGCTCTTTTCCAATCGGGTGACATATTTTGTTGACAATTTTCACGTTTAATTATAAAGGTAATTTG
CTCAAATTCGTTGCAGTTCTTCCTATTTTATCGTGATTCTTNNNNNNNNNNNNNNNNNNNNNNNNNNNNNNNNNNNNNNNNNNNNNNNNNNNNNNNNNNNNNNNNN
NNNNNNNNNNNNNNNNNNNNNNNNNNNNNNNNNNNNNNNNNNNNNNNNNNNNNNNNNNNNATTGTCGACAGCGATGTCGACCGTATGTCGACAGATTATTCCCCATTT
TCGACAATCTGCCCAGCGTGTGCCCAGTTTTCGCCCATTTCTTGACCTCGTTTTTCGACATTTTGTCCAATTTTTGACCAGAAATGTTTATTGGGATAGGTCCTGAAC
CGGTTAAGAATCTAATATGAACGTATAATTAAACCGGTTAATAGAACCGGTAATTTTGATCCGAAAATCAAATGTATTACTCCTAAGCTTGGGCGAACTTCTGAGCGA
TTACATATAAATCAGTTCAAATCATTGGTGAATCAGTAAACGGTAAATGATGCGAAAGCACTTTTAAAAGAGAAGTCATGAGTTTGAGTATTTCGCTAAGCACTAGAC
GCTTTGCTACCCTTTTTTGCGTGTATCAAAGTAATTTTGGAATATCATGTTAATTTTTATGTATTGTATACCTGAAGTGGTATGCGTAGAGATAATTCTCATCACATA
AAAAGAATTTTGTAGTAATTATCTAATAACATCCAACTTAGAATCAGTATCAGTGCGACTTTCAAACTGTCAAAGTCACTTTAATTTATAGATCTCGCAGTGAAATAA
AATTCTGAAATGACTCCATTTAAGTTCTATCACCATCCCATGAGTCCACCTTCGCGTTTGGTTTTTTTGACTATCAAGAACCTAAACCTAG***ATGCGAACATTGAAGTCC***
TCGATCTATTTAATAAAGCCCAATTAAGCCCTGAATTTGTGAAGAAAAATCCGCAACATACCGTGCCAACAATTGAGGATAATGGATTTATTTTGTGGGAGTCTCGAGCTAT
TGCTTCGTATTTGGTTTCAGCCAAAGCTCCTGGAAGCTCATTGTATCCGACAGACCCCAAGAAAAAAGCTATTGTGGATGCAAGATTATATCTGGATCAAGCTCTGCAACTT
GCACTGTCAGCAATTGTCGTAAGAGCAAAAATTTTATATCTTGAAAAGTGTTCATGATCAAGTAATATGTTAAAATCGGTAATGTTTCAG***TTTCCAATTCATACCCAAG***
GTGCCACAACCATTGAAAAAGATAAAAAGGACAAAGCTTATCAGATTCTCGAGAATCTGAACACTTTTATGGAAGGAAAACCATACGCTGCTGGAAATGAATTAACAATTGC
CGATTTGGCTCTTTTAGCCACAATATCAAGCCTCTACGTGAGTAGAAGTTTTTTTTTTAAGTTTACGTATAAATCTAATTTATTTTATTGTATTTAG***GAAATGGGAGCCA***
ATATTCCGAAATTCAAGAACATCATGTCTTGGTACAAAAAACTGGAGTCAATCCCTGGCTTTAAAGAGAATGGTGAAGGCGCTAAAGGTCTGGGGGATTATGTAAAATCGA
AAGTCTCTATTACCGGAACTTGGGATGATTAACACGATATCCTTGTACAAAAATGTACATTTGAATATAATTATTTTTGATTTGTCAAAAAGAAAGAAGTGCAGAAAA
TAAAAAAAAAATAAAAAAGACAAGTGATTTTGCATTTTTTGTTATCTGGAAATAAAATGAAGAGGTCAAGAGACCTGATAGAAAAGTGTAAATCTTACTTTTAATTTA
TATTTTCTTGCGTTCATCATTCTACGATAAGGAAAAGTCATTTTACGAATGAAAAGTCATTCTACGATACAGCGAAATCGATAAAATTTAGTTTAGAAGAGTTGAGTA
ATGGTCTTTTTTTTTAATTCCATCACATCAAGTCTCTACGGCAGTTTTTTTTTTATTTGAAGATAATCTTTTCACTAAAGAGATAAAAGGAAATGATTTAATTATTATGA
AAGCGCATGATTCAAGGGCGCAGCCAGCATTTCAGTAAGAGGGCGTACAAATTAAAAGCTTCTCCCCAAATGAAACCGTTTTTCGAACTCAAAGAACAATAATTTTCA
AGCAAATTTACTAAAGAAGAAATTCAATTTTTATTAATATGAGCTTTTTTTAAAAATTAATAAAATCCTTATTAAGACCTTTAGGAAAAAATACATTTAGGAACGCTT
TTTTCGAATAAATTATGTTTTTGTTTTCAAATTCATTTCCGCAACGATTTTGACGATTTTAATTGATTTAGTAAGAAAGTTTTCATTATGTATTTCAGTGGTTTTTTT
TATTTCAGTGGCTTTTTATTTTAGTGGCTTTTAAACCAATGTCCCAAGACATGCTCTTAGACTGAAAAGCCATTAGATCTTTAAAACATTTAACATTTAAAAGGTTTA
GTCTTTATACTTGAAAAATCAGCACAAACCTTGAGGAGTTCTCAGGTGAAAAAAAATTAAATCTAAGCTTTTGGTGAATTTTATAAGCTAAACGCTTGTTAAAGTCAC
TAAACTTGAATAGAAAATATTCTTCATGAATCACAAAAACTTTTTGCAAGTTTGTTTTTCGAAAATTCAAAACTAACCCCATTTTGCTTATTTTGAGGTCAAATGTGG
AAAGCTCTAAAGATACAATAAAAGCTCTTCTGAGACGACGTTGTATTATTTTTTTTGGGAAAAATTGGTAGAAAATTCCCTAAATTTCTCTCAAGAGCCTATTTGAAA
ATTTTAAAAATTAGATTTTGTGCAAATATTTTCAAGAAAATCACCGTAATTTGACTTTTAATTCATAATTAATTTATATGGGACCTTTTGGGAAGCTTATAAGATTCA

ACAAGAGACATTTCAAGAGAATAAAAGCGTCTTGAGGTGAGCTAAATGCTAATGTTCGAGATTTTTTTTCGCTAGGCCTTACAATTATTTTATTAATTATTATTATTA
ATTACAAGCTAGGCCTTACAATTATTTTATTTAGATGCACAATTGTTTTGTCTATCCAAATTACATAATCTTAAAATTCAGCTTCCTTTAGAAATATGCGAAAAAATC
GTACAACAATGTAACCCCACAGCTCAAAGTAGCCCCACCTCCCCCTAACGAATTAATATCCACATGTATTGATTTTGTCGTATTTTATGGCCTCTACACACTAGAGAA
ATTTATGTCCATATTGAAGAGCTTTTCCTATACAGGCGTAGGGAATTTGCTTCAATATGGACATAATTTTGTCTAGTGTGTATAGGCTATTAGAGTTATGTAAAATCA
TGAAAAAATCTTACTAAAGCAAAATTAGTCAAACTGTTATTTCTAAGAACTTTATGCAAATGTTAGACAATGCAAATTATCGAGCTTGGTACAAAAGACTGGAGTCCA
TTCCAGTTCATCACGAAAACCTCGAAGGTGCAAAAACTCTGGCACAGTACATAATAACGAAACCCAATTTTAGTTGGGATGATTAATGTTATGTATTTCCGGAAAGAA
AATAAATGATTTTCCAGAACAATGAAAGGGGTGGCGATATCTATGATACGGACTACAAATTAATTTCTCATCAATATTATACGATAATAAATATGTAAAGTAGTTCAA
TTTGGAGCATATTATGCTTATGAATTGGAATGTTGTAAGAAGTTTTGTTCATATCGTTATTTCTTAAGGATATACAAAAAAAAATGTAATTTTTTTCATGAAACTTGTG
AGTAATTCAAGAAACACATTTGAATATTGTGTACTAAAAAATGTTGTGTACTAATCCAGAATAATAAATTAATATAAAGCATGTTATTGAGATTAGATCAATATAGAG
ATCTATCTGGATATTTTACGATTGTATTTTAACATATTATTCAAAAGAATTTCGGTTCGACAGAATGTAGCAGGAAATAGACGGAGACAGTGAACCTTTCTTGATTTT
CACAATTTTATTCTCTACTTTTTAATATCATTCAATTGATAAAGTGCTACCTCTACGATGCCATTATCTCACGTTCGAATCCCTTCTAAGGGCCCAAATAGACTCAGG
CTGATTCTCGAGAATATTTAGCCTATTTATAAAATTTGGCGGCAAAGGATTAGGTAAAGGAACTTTATGCAAAGGACCTCTAATCGATGATTCTAAAGCCTCAAATCA
TTATTTTGATTTATAACCATATTAAGCCGTCTCTCGGCTTAAGTCACAAGTGCGTCCAGTACATTAAGCTTGGATCTGTCAACTCCATCCTTACTTGTCTAGAATTCC
ATCTTGTAAAAAAATAATTTGTCAACAGACTTAGTTTGAAAAAAACTAAAACACGGAAGAGATTGAGCAAAAAGCAAAAGCAATATTTTAGCCAAAAGTAATTCTGTG
TCAAAAATAATACTTTTATTGTGTTAGTTTTTTGTTAGTGCCACTCGTAGCTCTCTACAAAGTCGTATTTTGTGTGTATCGAAAATGAAGTTGCGTATAAAAAAATTTG
GAATACCATGTCGATCTCTTGTGTATTTGCATACTCGAATTGGTATGAGTAAATATATTTCTCATCAAATAAAAAAAATGTATTTATTATCTAATAACGCCCAATTTAG
AATCAGTATCAGTGCGACTTTCAAACTAAGTTAAAGTTACTTGGCTTTGTAG***ATTTTGCTGTGA***ATAATAAATAGTGAAATGGCTCCAATTAAGTTCTATCATCATCCT
ATGAGTCCACCATCGCGTATCGTCCTTTTGACTATCAGGAACTTAAATCTAGACGCGAACATTGAAGTCGTTGATCTTATGAATAAAGCCCAATTAAACCCTGAATTT
CTGAAGATGAATCCGCAACATACCGTGCCAACTATTGAGGATAATGGATTTGTTTTGTGGGAGTCCCGAGCTATTGCTACGTACTTGGTTTCGGCTAAAGCTCCCGGA
AGCTCCTTGTATCCTTCAGACGTAAAGAAGAAGGCCATTGTGGATGCCAGATTGTATTTGGAACAAGCCTTGCAAATTTCTCACAGTACAATAGCGGTAAGATCAGTC
GTATCACTCCGATTCGTGAGAAAATCATTAATTATGTATATTTTATTAAACTTTCAGTATCCAATTTTTCTGGGAGAGGCTACAACTATTCCAGATGATAAAAAACAA
AAAGTCTACCAAATTTTGGGGAATTTAAATTCATTTTTGGAAGGTCAAAAATATGCTGCCGGTGATGAGCTAACCATTGCTGATTTGGCTCTTTTGGCTACCATCACA
AGTATCTACGTAAGTGAAGCAATGTTTTTATATTTTCTCAGATAACATTATCTATGAATTTTAATTATTAGGAACTTGGCGCGAATGTTAGTAAATTCAAAAACATTT
ATGCTTGGTACAAGAGGATGGAGTCTGTTCCAGGTCATCAGGAAAATTATGAAGGCGCAAAGGCTTTAGGAAACTTCCTACGATCAAAAGCTGATATATCAAGCAACT
GGGATGATTAGATGTATCAGTGGATATGTTTGTCATAATTCAATAAAAGTTACAGCTGGGGTTCACGAATTTTACAATACTTGAGAGGATATTCCAAGTCCCAATTGA
TAAATTGATTAGCGCAGAGATTAAAAAGCACAACAAAAGCATTGCTTTAACTGTTTGGTTTCCAAAGTCGTATGAAAAGGTAAGATAAACCATGATGAATAAT

>PPAI010869 (221 aa; 3 exons) | UNCHANGED
MAPLKLYHFPVSPPSRIAVLVVRNLHLDVEIITVDLMNRGQLTPEFLKINPQHTVPTIEDNGFILWESRAIASYLVSAKAPGSSLYPTDPKKRAIVDARLYLDQALQL
ALTAIVFPIHAHGATTIEKDKKDKAYQILENLNTFMEGKPYAAGNELTIADLALLATITSFYEMGANIPKFKNITSWYKRLESIPGFKENGEGAKALGEYVKSKVTVT
GTWDD-

>PPAI010870_a (219 aa; 3 exons) | EDITED
MAPIKFYHNFLSGPSRGVLLTIRSLNLDVEIETLDLLKQAQLAPEFVKINPQHTVPTIDDNGFVLWESRAIVSYLVSAKAPGSSLYPTDVKKRALVDARLYLDQSLQL
VMSSAVYVIFTGEATKIPVDKKQKLFQILEHFNTFLEGKKYAAGDELTVADLALLGTISTLYELGANVSKYTNIDAWYKRLESIPGHHENLEGAKALAQYIITKPNFS
WDD-

>PPAI010870_b (221 aa; 3 exons) | EDITED
MTPFKFYHHPMSPPSRLVFLTIKNLNLDANIEVLDLFNKAQLSPEFVKKNPQHTVPTIEDNGFILWESRAIASYLVSAKAPGSSLYPTDPKKKAIVDARLYLDQALQL
ALSAIVFPIHTQGATTIEKDKKDKAYQILENLNTFMEGKPYAAGNELTIADLALLATISSLYEMGANIPKFKNIMSWYKKLESIPGFKENGEGAKGLGDYVKSKVSIT
GTWDD-

>PPAI010870_c (221 aa; 3 exons) | EDITED [GENE JUST DOWNSTREAM OF PPAI010870]
MAPIKFYHHPMSPPSRIVLLTIRNLNLDANIEVVDLMNKAQLNPEFLKMNPQHTVPTIEDNGFVLWESRAIATYLVSAKAPGSSLYPSDVKKKAIVDARLYLEQALQI
SHSTIAYPIFLGEATTIPDDKKQKVYQILGNLNSFLEGQKYAAGDELTIADLALLATITSIYELGANVSKFKNIYAWYKRMESVPGHQENYEGAKALGNFLRSKADIS
SNWDD-

####### PPAI010871 - GSTX #######
>JH660956.1 forward | **PPAI010871** (pre-editing) | PPAI010871_a, PPAI010871_b, PPAI010871_c, PPAI010871_d, PPAI010871_e (post-editing)
GTTAACCCAAACATTTTCCATATTTGGACAAAAACTCCAATGCGTTTTCACGAATTGTTTCCACACAGATAGTAGAAAAAGTGAGTCTCAATGATAAGAAAAATGTGT
ATATAAGTGTCTGCACCTCAATAACGCCACTTAGTATTGAACTACCAACAGGTGAAATTGTGCTTTGTGAAAATTCTTGAATAAACAAAAAC**ATGGCCCCAATTAAACT**
TTACTATTTTAATCTTAGTCCTCCATCACGTTTAGCTTTATTAACTATTAGGAACCTGAAATTGGATGTGGAAATAATTGTTATCGACACCATGAAAAAGGAGCAAATGACT
CCTGAATTCATGAAAATTAACCCTCAACATACAGTGCCAACCCTTGACGACAATGGATTTATTTTGTGGGAGTCTCGTGCAATTGCATCGTATTTAGTTGCGGCCAAAGCCC
CTGGAAGCTCACTATATCCTTCAAACATAAAGAAGAAAGCCATTGTTGATGCTAGACTATTTTTGGATCAAGAATTACTGAATGCAGCTGTACCGGTTATGGTAGTTTATA
AATTTGGAAGAAATATTTCCAGAAAAACTAACACTCCAATATTTTTAG**TATTCAATTTTCGCACAAGAAACAACGCCAGAAGCTGCACAAAAGAAGGAAAAGGTTTATAA**
GATCCTTGGAAATTTGAACACATTTATGGAAGGGCAAAAATACGTTGCCGGTAACGAATTAACCATAGCCGATCTCGCACTTTTGGCCAATATCGCCACCCTCTACGTGAGT
CAACTGAATTTTAATCATTTACAATCTGTCTTATAACCGTTTTCTTTTCAG**CAAATTGGTGCTAATGTCAACAAATTCAAAAACATTGCTGCTTGGTACAAGAGGTTGGA**
AACTATTCCTGGTTACCAGGAGAACCTCGAAGGTGCAAAAATTGTTGGACAAAATCTAAAACCAATGATGACCTTTAAAGGAACTTGGGATGATTAAACTTCCATGTAT
TTTGTTGTTATTTTAAACAAAATAAATGACGTTGCAAAGTTCTCATATTGAAATCTTTTATAAATTCTAATCACGCTAAATATCTCAGCTAAATCATTGAAACAGAGC
AGTTGTTTCTGGAACTATAGTAAGATCCACTGCGACTTTTTAATTAAATTTTAGACTAGACGGGGAACTTTATGCTCTCAATTCTTCCGGTGTTCGTCAGAAGGGTAT
TCCTCTTTTCAAATTAAAAGAATCTGTTTTTTCTCCAAAGCTTTCAACCCTTGGTATGGGTATTCTTCAGTGGGTCAATTTAGAGACTTTTAAATATTTGAAATATTT
AAATATTTGAAATAAATATGATATTTTCTCCAAAGCTTTCAACCCTTGGTATGGGTCTTCTTCAGTGGGTCAATTTAGAGATTTTTAAATATTTGAAATATTTGAAAT
ATTTTCAAATATTTAAAAGTCTCTAAATTGACCCACTGAAGAAGACCCATACCAAGGGTTGAAAGCTTTGGAGAAAAAACATATTCTTTTAATTTGAAAAGAGGAATA
CCCTTCTGACGAACACCGGAAGAATTGAGAACATATTAGACTAGACTTAGGGATGCTTGTCCATTGAAGAAATGGTCGTCATGGTCGTCAATAGTCGTCATAGTTCGA
ATGAGTTTAGAATAAAAATCAGTGTTCAGAAATAAAAAAAATGATCAGTGGTGCCCCAGTTTCACTTATAATTTTTCTTTTATTTCTATAAAATCGTTTTAGTAGTT

CTAGATGATTATTTTCTAAGCAATTTTACGAAAATTGGATTTGTTTGAAAGGTAAGACAAGACCACTCGCCATGGGCTAGAATAAATCCAAATGGTGGAAAATGGATG
CGTAACGGTTTGTTTGAAAGGCAAAAAATCCAAAACGGGGCTAGCTAGAGTATTCTAAATCCAAAATATCGCAGACTTCCTTTATCAAAGCGTTAAAATAAAAAGTAC
TACCAATTGGAAGAAGGCAATGTTGAACGTTTAGTGTTTAGTGGAAGCGGCAGGGAATCATGAAATACTAGTTGCTTACCAACTATCAATAGACATCGATAAATTTCG
ATCAAGTATTCAAAATCCGACCAAGCCCGATCAAAGAACATAATAATACTGATATTAAAACATGACAACGCTTCATGGCATTGGACTTATATGTTACAAGACATCATC
AAAAGCTAAAATGGCTAAAATAAAATGTGCTAGTAATTCCTGTTGTACTTTTTAAACTTATCTCCTTCCAATAACCATTTTTTTGCTAATTTCTCATGAACAGCTTGA
GCAGCACTTTGCCAATTTCGAAGAAATAAAAAATTAAGTGTTTATAATATGATTTTGGTCTAAGGACGTCTAGTTTTATTGTTGAGGTATCCATGTGTTGCCTGAAAT
ATGGGAAAATATTCAGGTAGCAAGGACTGATATTTAAATTAGATTTTTTTTTTTGTATTACTCGAGAAATTTCCTATTTTCAATTGACAAAAACCTTAAAAAAAACAA
ATAGTTAAAGACCTTAGTAATTTTGATCCAAAATGTTTTTTAAATTACTGTAAAACCCAGTTTCCATTAGAAGTATGTAAAAAATAGCGTTTCCAATGGAGTCCCAGC
TCAATGTAACCCCACCTCTCCTATGCGCACTTCATATCTAATAAAATTTAACCATTTAGTTCAACTCTTCAAATTTCTCATCAAGCAAGATTTCTGAACATTTTTTTT
AAGATCTTAAGATTTTGACTTTGAATCATCATTATCATTTTCAACCGCTTATCCCTATTGGGTCGTGGACCTATGACTGCCAGTTTAGCAGCCCACGCCTTTCGATCC
TTTGTCTTTATTAACAAACAGCCCAGCAAACATCAAATACTAAAGCATTTCAATTCAGCTGAAAACATTTGTATTTTCAGCTGAATTCTGCTAAACATAAAGGAAAAT
TCGCGAGGCAGAGCCATTTCCATATTTGGTTAGGGGAAACTGATCTGCCTTTGAATGTGTCAATTTTAAAGTCCTTTTCCAATTTTAGTCCTTTAAAAAAAGACGTGT
TCTGGAAGATGTTCAAGTGCGATCTTAAGGGGTTTCATGGGTCTCTCAGGTCAAAAAAATCATTTTTTTTAATTTATAGTTATAAAATTTGAAAAATATTTTCTGAAA
ATTTCAAGTCAATCGAAGTAAAACTCTCGGAGGTAGAGCAGTTTCAGTAATGCATTCCGCCTGCGCGCGCATATTATTGTAAAACTTTAAACGCGTTTTTTTTTTCAA
AACACCGTTTTACAATGCGGTAGTCAAGATTACTGAGAGTCTACTGAACCGATCTTTCTGAAATTTTCCATACATGTCTTGAGAAGTATTATCTACTGTTTGAACGAA
GGATTTGCACTTTCTTTAATCAGAACCATTTTTACAACACAAAATGTGTTGAAAAATAGGGTAAAATTAGTATTTTTTTACAAAACTTTTGCCAAAAATCCAAATTT
TGTTTATTTTTTTTCAAAAGTCCTTCGTTCATCCACGGGTCCAACTAATCCTCTAACAGAGTGTATTCGGTTTTTTGATTACAGATGAACCTACTCGGAGAAATCCTG
CCTACCGCAAGGTCTGTTTTTCGAAGAATGGTTCCGAAAATCAGCTACTAACGGTCTAATTTTCAAAATTTTTCAAAAATTTTTTTTGTTTTGTTCTACTAAAGTTAC
TCTTTCATATCCCAAATGTTGGAATTTCTAAAATATTTTCGACATAATAAATTATTATTAGAAAAAAGGCCTATTTTTTTAACCTGAGAGACCCATGTAGCCCCTTAAG
GCACTCCAAAGCAAAATCCTGAATTTGATTCAGCCACCTTGTTCTAGGTCGGCCCCGAAGTTGACCCTTCTTCACAATGATTTTCATAGCTTCTTTGGGAAATCTGTC
ATGATTTTTCTTTTCAGATCATTCGCTTTCTTTTTTGACTTTGAATATTAAGGATAAGTAATCCATTAGATTTGAAATTGTTTGTTTTAACAGATTTTGAGACATCAA
GAAATAGGCTAAATCGCTAGTCTGAAACATGTATTAAATAGTTCAAGTACCCTAATATTCATAAAATTAAATGGCGTTTTTCATGAGAAGATGGAGTGCTAAAAATAA
ATTTGTCTTGATTGAATTTTATAGTTATTCTTTGTTTGTAAATGTTTAAATACATGAAAAAGTATATCTCTGGTTTGTCTGTTTTTTTAGATTAAATGAAGTGATAAG
AAAACAGTAATGAATACCGCAAATGTTTGTTTTTTAATATTATTTTTAATTAAATTTTATTCTAGATCTTTTGTGGATAATTTTGAGTCAAGAAACACGGTATATAGA
CCTTTTTAAAAACTTCTCTATTTTCTCCTGCTGAGGAAGGAGACAACCAATCTCCGAAACGTCGTAGCAGGAGGAAATAGAGAAGTTTTTAAAAAGGTCTTATACCGT
GTTTCTTGACTCAATATTATTTTTAACATAATTCAGAATATTTAAAAAATAATAGGGGAAAGCGTGCTACATTTGGCTGACTCCAGCTTTGAGCAATTAATTTTTTTT
CAATGTGTTTTAAATTAATATGGCCCATTATGATTATTAAAGTTTAATAGATAATCCGGCTAGTCCAACGCCTCAAATTTTTTTAAAAAAAGAGCAAAGGGTGAAATC
CATAAAGAATATAGAAAAAATTAGATGTACGAAGTTTGAGTCATCCGATGATAGCGCACTTTTCTTTAATACTGTCTTGCTTACATCTTGAATTTGATTAAAGTTTTG
AAAGAAGATCATCTTTAGTGAACTACTAAAAACTGTGTCTGAGTCATAAACTCAGATTTTTTTTACCTTTGAACTTTTTGCAATACTATGACTAATTTGCAGAAGCTT
CGAGAAAATCAAAAGTACTTATGGTAAAAATGCTGCTTCATTTTAATGCGTCTTTGAAGATTATTTTTATACAGATAGCAGAAAAGGTATCAATGATTAGTGAAGGGT
ATATAAGGGCTTTCACTTCTCACACAAATAACATCAGTTTGTGTTGGACTACAACCAAGTGAAGTTGTGTTTCTGATAAGAAGTTTAAAGAAAAAGTGAAG**ATATGGCC**
CCAATTAAATTTTACCATTTTAACATTAGTCCCCCATCACGTTTACCCATGCTAACTATTAGGAACCTGAATTTGGATGTGGAAATAATTGTTATCGACACCATGAAGAAGG
AACAAATGAGTCCTGAATTCATGAAAATCAACCCTCAACACACCATCCCAACCATTGATGACAATGGATTTATTCTCTGGGAGTCTCGTGCTATTGCTTCGTATTTAGTATC
GGCCAAAGCTCCTGGAAGCTCACTGTATCCTTCGGACATAAAGAAGAGGGCCATTGTGGATGCGAGATTGTTCTTGGATCAAGAAATGCAAAATGCTACTCTCCCAGTTATG
GTATAGTTTGTGAAATTTAAGAAGTTGCTTCCAGTAAGACTGACAGTTCTATATTTTTAG**CATGCCATCTACAACCAAGAACCAAAAGATGTCTTTATAAGTAAGACTGA**
AAAGCTCTACAAGATCCTTGAAAATTTGAACACATTTATGGAGGGACAGAAGTATGTTGCCGGTAATGAACTAACTATAGCCGATCTGGCATTTTTAGCCAATATCTCCACC
CTTTACGTGAGTCAACAGAATTTTAATCACTTTCATTCAGTCTTATCAGTGTTTTATTTTTAG**GAAATTGGAGCAAATATCAACAAATTCAAAAACATTGCTGCTTGGTT**
CAAGAGGCTGGAGAATATTCCTGGTTACCAGGAAAACCTTGACGGCGCTAAAGTCATTGGGGATACTTTTAGACCAAAGATGACCTTCAAAGGAACTTGGGATGATTAAT
TGAATTGATATCAATATGATCATGAGTAATCAAAATAAATATTGCAGAGCAGAGCTATAACAGAAAAATCTTTTTCATAATCAAATAACGAATAAAGTATCAACTGAA
AAAAAAATACAAAAGCTATATCGATTTACTACTCAAGTTGTATCCCCTCCCAGGTGTAAATGGTTCACATAATTTACTTATCTCTAACTTAGGCAGGGCTGAATAAGC
TTCATGGTCTTTTGCAAATTTATATATCAGCGTCTCGATTGGAGTTTCTGGCGTTAAATGTGTTCGAATTGCATCCAGTGAGCTTCACTGCACTTGATTCCACGGGGC
ATGGGACTGATAACCCCATCCCTGTATAAGAAAAAATAATAATGGATGTCCCGAATTCTCCTTGTGAGAAGGCCTAGTTCTTCCATGGAACGTTGTGCCACCATTATT
ATTATTATGTCTCGAATGGTTCAGCAACTGTACTAACAGCATTTTCTTCGTATCTAATTCACTGCAAATTCCATGCAGTATTTAGTGGTTTACAATGAATGCAGACAC
AAAGCAAATACAGTTAGTGCAGTCGCTGATCTAACCAATGCAACGATATACCTATATTTCTGAAGTTTGCAAAGTTTTGAATAAGTACTATCTTCAACAATATACTAA
AATTTCTCATTGCAGCTGAGGCATAAATTAGAAGAAAAAAAAACAATATAATAATAATAACTTGTAAGCATCCAAGACAGAGTTATGACTAATTTAACAGAAGTTTTG
AGTAAATTAAAAGCACTAAAATGCTAAAATGCTGTTTCATTTTACACATAGGTGAAAAGTGAGTTTTATTGATTAGAGTAAGGTGTATATAAAGTCTTCTATTTATC
TCGAAAAATCAGTTTTATTTGGATTACCAACAGGTGTACTCGTGCATTGTGATAAAAATCTTGGTTGTACGATTAAAAATGGCCCCAGTTAAACTTTATTATTTTAAT
GTTAGCCCCCATCACGTATAGCTCTATTAACTATTAGGAACCTGAAG**TTGGATGTGGAAATTATCATTATCAACACTATGAAAAATGAGCAGATGTCTCCAGAATTTAT**
GAAAATCAACCCTCAACACACCATCCCAACCATTGATGACAATGGATTTATTTTGTGGGAGTCTCGTGCTATTGCTTCGTATTTAGTTGCGGCAAAAGCTCCTGGAAGCTCA
TTGTATCCTTCAGATATTAAGAAGAAAGCCATTGTTGATGCAAGATTATTCTTAGATCAAGATTTATTCCATGCTTCAGAAGAGGCTATGGTATACTTTGTATATTAAGAC
AAGTGCTTCTAGAAAGACTGACAATATCATATTTTTAG**TATCCAATTTACGAACAAAAAGCTACCACAGTTGCTGAGGATAAGAAGGAAAAGATCTACAAGATCCTTGGGA**
ATTTAAATACATTTATGGAAGGACAGAAGTATGTTGCCGGCAATGAACTAACTATAGCCGATCTGGCACTTTTGGTCAATATCTCCACCCTCTATGTGAGTCAGCAAAATT
TTAATCAACTTTGCGTAGTCTCTTATCACTATTTTATTTTTAGGAGATTGGAGCAAATGTTAACAAATTTAAGAATATTGCAGCCTGGTACAAGAGACTAGAGTCTAT
TCCCGGTTACCAGGAAAATCTCGAAGGTGCTAAAATCATTGGACAAATTCTGAAGCCACAGATGAACCTTAAAGGAACTTGGGATGATTAGTTCAGATATTAATTTTT
CACAATTTTAATCAAAAAAAAAACATTGCTGAATAGAGGGAAATAAACACCTAGGGTAAATGTCCTAATTCAAAACCATTTCCAGGCGCTTTAACTTTGACAAAAAAT
TCAACACATTAAGTTCGAATTTTTTCTGAAGAGAATTAGGGGAAAGTTCCCATGCTTGATACTATAAAATGATGTCCAAGGTTTGTGATTTTTTTCTGATTAACACAC
AAACCGAAGCGATTCTCTCACTATGACAAAAAAATGTCTCACTTATAAGGTTCATAATCCCACCTCAAATAGTTCCTGAAAATCCTTAGATTTCCTCAAGAAGAAAA
TGGAAATTCAGTGGCGATTTTTATACAAGTTGGGTCCGGGGCCTTCCTGTATAATAATTGATTCGACAATTCGGAGGCCCATTTTCACTGCAAAAATTTCACCAGATA
CAAAAAATTGCACATCAATATTAAGACGAAACTCAAATCTATCTGCTTAAATCGTTTGTACGACTGGTTATTAGTTTTAAAATCATTTTTATGTAATTTTTCTAAGCC
GTGTTTTTATGACATTAGGACGCTAGCGAAAACCATTTTTTTCGCTTTGAGTCCATGAAAATATCACTTTGCAACCTTAAAATTCAGGCAACAGGGTTGAAGGCTATAT
CAATTGTCGATAAAATTGGAGGATTACAATAGGTGTAATTTTGAAAGAATCACATCTAATGACACATTTAAATTATCATTCATGGACCCTTCTTAAAATATAGGATGG
ACACAGGTAGGATTTATGAATTTGTAACCATGCACAAAAACAGAATCCACCAAGTAAAAATATTTTTACATAAATATTAATACTGATGAACCCTCTATGTGCCTATGA
TAATAGTTTTCCTGACAGCGACATTAATTAAGAAATACTTATAGAAATAATCNNNNNNNNNNNNNNNNNNNNNNNNNNNNNNNNNNNNNNNNNNNNNNNNNNNNNNNNNNN
NNNNNNNNNNNNNNNNNNNNNNNNNATGATAATTTCCACATCCAACTTCAGGTTCCTAATAGTTAATAGAGCTAGGATCCCAATTTTTTTAGGAAATGTGAAGCTTAG
GATCAGCTATTAAAATATATTGCTATGAATCACAATTATTGATTAACGTAGGAAAAAAGTAGTTATTATCAAGCTTGGATCGTATCAAGCATGGGCACTTTCCCCTAC
ATAAATTTGCTACTTTGCTCTTCTAGAATGTTACTATTTACACAGAAAAAAAATATTTTGCAACCATCCCGAAACAAATTAGGAAAATTATTTATCATTCTCTATGTG
AATCTCTATTGAGTTATGGGGTTGAGATCTGGGGCTGTACAAATAAAACACTATTAAAAAAAGTTCGAATAGCACAAAACAAATTAGTAGAGACAATATTTAATTATA
ATAAATTCACAAGTAGTAAAATATTATATAAAGAATTAGATTTGCTTCCATTACAAAATTTAACCAAATTACAACAAATACTTCACATACATAAAATGAAAATTGGTA
AAATTCACACACAAAGCAAAATAGACTTAAAAACACAAGACAGGAGAAGTAAAGGAAATATTATTAACCCAAAAGTTAAAACCACTAAATATGGTTTAAATTCTATAA

AATATATTTCCATCACAAACTATAATAAATTACCACAAAAACTAAAAACTATGGCAAATGAAATCCATGTTAAGAAATTTAAAAAATTAATCAGGGAATACATTGTAA
GCTAGAATTCTGTATTATGGGCGGTCGCAACTCAATGTAAAAATCGTACATATAGTCTTAAGAAAGGATCGTTGGATCCAAAAGGCAGGAAATAAACTGAAATCTAAT
CTAATCTAAAAATTGTTCGTAAATGTTTGTGAAATCCTATGGCAGACTTACGAAATGCTCGTGAATTGTATAACCCACAAATAAGTTCGTAAAATTTTGTACTTTTTT
CACAAACATTGTTCGTAAAATGATCATTTGACGAACATTTTTTGTACTTTTACAAACATTTGTTCGTAAATGTTCGTAAACATACAAAAAATGTTCGTAAAATTTTGT
CATTTGTTCGTATTTGTATGTAATTTTTTTCCAGACAAACAAAAATTATTACGTTATATTACAGTGAAAATGTCTTTTTCTAGACATTTAGATCTTTGCATCGGGCGG
AGCTCGAACTCACAATACTGTGAGTTAAGCCGGGGATTTATCCACCCAAGAACAAACAATCTTGCCCATTGCACCACTGAGATCCCCTACAATTACCCTAAACCGAAA
ATTAAAGTCATACGTAAGTTTGTAAGATTCTACACATATTGTCCCATTGAACTAGTTAATTTAAAGAAGACTCGAGAACTAATTGAGCTTAAATTAACTTAAAATTAC
TCGAAGTTATAACATGGATTCGAGCAAAACCAGTGGTGATCTAAACTGCTATTTCATTCTTCCAATACTTCTGCATTTGGATATCTACAGATAGACCAAACAAAACAA
GTCATTCTCATTGATAGAAAAAAATTGTGTATATAAAACTCACAATCACCAGCACCATCAGTTTCATTCAGACTGTTAAAAGGTGAACTTGTGCTTCGTCAATTCATA
AG**ATTCAATTAAGCTACGAAATGGCTCCAGTAAAATTATACTATTTCCCCTTGAGTCCTCCATCACGTGCAGTGTATCTAACAGTTCAAAATCTAAAATTGGATGTGGAAAA**
GACTGTTATCGACACTACGAAAAAGGAGCAGTTGGCTCCAGAGTTTGTAAAGATCAATCCTCAGCATACAGTCCCAACCATTGATGACAATGGATTTATTCTCTGGGAGTCT
CGTGCTATTGTTTCGTATTTAGTATCGGCAAAAGCTCCCGGAAGCTCACTGTATCCTTCGGACATAAAGAAGAAGGCCATTGTGGATGCGAGACTATTCTTGGATCAAGATT
TATGGGCTGCAACCTCGGCTATCATAGTACGATTTGTACAAAATTATTATTGATAAAGTATTTACTACAATATTCTTAATTTTTAG**TTGTCGATCTACGCTCAAGGAGCT**
ACCACAGTTGCTGAGGATAAGAAGGAAAAAGTCTACAAGATCCTTGGGAATCTGAATACATTTATGGAAGGACAGAATTATGTTGCCGGTAACGATTTAACAGTAGCCGATC
TGGCGCTTTTGGCCAATATCTCCACTCTCTACGTGAGTCAATAGAATTTTAATCATTTTTGGGTAGTCTTATCAGTGTTTTATTTTTAG**GAAATTGGAGCTGATGTTAAC**
AAATTCAAAAACATTGCTGCTTGGTACAAGAGGCTGGAGACAATTCCTGGTTACCAGGAGAATCTAGAGGGTGCCAAAGGCATTGGAAACTTCCTCAAAGCAAAAATGAC
TCTCAAAGGAACTTGGGATGATTAATTCACCACTTAATTTTTTTTTGGAAAATAAATAAAGTAGTTGCTTCTTTTGAAATCGAATCATATCAGTCTGGTGATAAGAAT
TCAATGTTTAGGGTAAGTGTACCAAATTCCGGCCAGCTTGCAATTTCGGCCACCTTTTTTGTTCCTCGAATTTCCATGAACTTTTAGATTTTACGTACTCTAGAGATT
ATGCAATGCAAAAGAATAACAAAAAAATGTAGCTTCGACAAACGAGATGACGTGAAAAAGACATTGGAAGAATTCCCGAAGGGCTAGGAACTATGAGAATGAAGGTGG
CCGAAATAGGGTACCAAAGCTATGTCTATGTTTTTGTTCATTTTAAAATATATTAAGAATGATTTTAGAGTAAATAAAAACGGTAAACTCTTTACAAGGTTCCAAGCA
ACACTCTTTAAGAAGAAAGAATAAAAAAATCAATTTGTATTTAAAATATTACATTTCAAACTTGAGACTTTGACGCTTGCATGCAACTATGCCGAAATTTGGCACACT
TACCCTACCACTTCTAAAAATGGAAAAAATCTAAAAATCATTGTATAATACTGCAACTTCAGGTGCTTTAGCACTATCTTTTGATATTCTGTATCTAGTGTTTCCTTA
GATTTTGATAAGATTGGAACAATTCAAGCCCTGCAATGGCTCCTATTAAATATTACTATTATCCAGAAGGAGCTCCTAATCGCGCAGCTCTTTTGACAATACGGAATT
AGAATCTTGAAGCAGAATTGATCGTAGTGGATTTACGAAAAAAGAACATCTAACAGAGAAGTTTATTAAGATGAATCCGCAATGTTGTATGCCCGTTTTGGATGACAA
TGGATTCATTTTGTGGGAATCTAGGGCTATATCTCAATATTTGGTCACATCTAGAGCTCCTAGGAGTTCACTCTATCCAACTGATCCTAAAATGAGAGCTGTAGTAGA
TGCTAGATTATTCTTGGATACAACTCTTCATGCTGTTTCAAGATCCTTCTTCGTAAGTTGGAAATTGAATTTGAGGATTATTGATAGACCCTAAAATTAAAACGCTTT
TTTTCAGTATCCTATACATTCTTTAGGGGAAAAGCAAATCCCAGAATTACGAAAATAGAGGCTTTATCAATTGCTAG**GTTACCTGGACAATATAATGGAAGGTCAAAAG**
TATGTTGCAGGAGATGAATTAACCATCGCCGACTTTTTAATTTTGGCATCTTTTTCGACTTACTTTGTAAGTTTTACAAGTTTTAAGTATTGCAAAATCGTAAAATACGT
CTAATTATCTTTCTTAG**CATGCAGGAGCAAATGTAAGCAGTTTGAAAAACCTAATGTCTTGGTACAAGAGATGTGAAAGTCTTATCGGTTTCCAAGAAAACGAAGATGAAG**
CAAAAGCTTTCGGAGAAATGTTAAAATCTAAATTAGGTATTAAAAATACTTGGGATGAGTTGCAATAAAAAGCATTTGAAAAGAAATCAACTTTTTTATTAGTGAGTTAC
ATAATCATGACCTTGAAGAAATTGTACATCAAAATTACTTTTCACTTTCATTTTCACATCTCTTATCAAATTAGATTAGACAATTAACTGATAAAAACGCTACACTTA
AATTCATTTGAAAAAAATAATTATAATGCAGAATTAGTCAGTTTTAATCC

>PPAI010871_a (221 aa; 3 exons) | EDITED
MAPIKLYYFNLSPPSRLALLTIRNLKLDVEIIVIDTMKKEQMTPEFMKINPQHTVPTLDDNGFILWESRAIASYLVAAKAPGSSLYPSNIKKKAIVDARLFLDQELLN
AAVPVMYSIFAQETTPEAAQKKEKVYKILGNLNTFMEGQKYVAGNELTIADLALLANIATLYQIGANVNKFKNIAAWYKRLETIPGYQENLEGAKIVGQNLKPMMTFK
GTWDD-

>PPAI010871_b (221 aa; 3 exons) | EDITED
MAPIKFYHFNISPPSRLPMLTIRNLNLDVEIIVIDTMKKEQMSPEFMKINPQHTIPTIDDNGFILWESRAIASYLVSAKAPGSSLYPSDIKKRAIVDARLFLDQEMQN
ATLPVMHAIYNQEPKDVFISKTEKLYKILENLNTFMEGQKYVAGNELTIADLAFLANISTLYEIGANINKFKNIAAWFKRLENIPGYQENLDGAKVIGDTFRPKMTFK
GTWDD-

>PPAI010871_c (221 aa; 3 exons) | EDITED
MAPVKLYYFNVSPPSRIALLTIRNLKLDVEIIIINTMKNEQMSPEFMKINPQHTIPTIDDNGFILWESRAIASYLVAAKAPGSSLYPSDIKKKAIVDARLFLDQDLFH
ASEEAMYPIYEQKATTVAEDKKEKIYKILGNLNTFMEGQKYVAGNELTIADLALLVNISTLYEIGANVNKFKNIAAWYKRLESIPGYQENLEGAKIIGQILKPQMNLK
GTWDD-

>PPAI010871_d (221 aa; 3 exons) | EDITED
MAPVKLYYFPLSPPSRAVYLTVQNLKLDVEKTVIDTTKKEQLAPEFVKINPQHTVPTIDDNGFILWESRAIVSYLVSAKAPGSSLYPSDIKKKAIVDARLFLDQDLWA
ATSAIILSIYAQGATTVAEDKKEKVYKILGNLNTFMEGQNYVAGNDLTVADLALLANISTLYEIGADVNKFKNIAAWYKRLETIPGYQENLEGAKGIGNFLKAKMTLK
GTWDD-

[The cyan highlighted region indicates the start to the end of an additional gene (PPAI010871_e), but no complete gene model could be reconstructed.]

####### PPAI010872 - GSTX #######

>JH660956.1 reverse | **PPAI010872** (pre-editing) | PPAI010872 (post-editing)
GCCCCCGGCGTTACCGAGTACACTGGCAATGGATCCGACTCTGTACTCCAAAATTATCTCACGTCAAAATTCTCTGTAGTAGTGAAAGGCAAAGTTAAGTATAGAGAA
AGTCAGTCTGATAAGTGTCTTACTCGAAGGTAGGCGGAAACTCTCAATTTCTCCAGTAAAATTCTAATGGAAAATTTCCACTAATTCTAGAA**ATGGCCCCTCTAAAGTT**
CTACTACTTCCCTGCGAGTCCCCCATGTCGGGGAGCTCTCTTGGCGATTCGCTATCTGAAGCTGGATGTAGAGATGATCAGTTTGAACTTGCGTGAGAAGGAACAATTGAAG
CCAGAGTTCCTAAAAGTCAATCCTACTCACAGTGTTCCTACTCTGGATGACAATGGTTTCGTTCTGTGGGAGTCTAGAGCTATATCTCAGTATTTGGTCGCTACTAGGGCTC
CTGGAAGTTCTCTGTATCCAGATGATCCCAAGAAGAGAGCTATAGTAGATGCCAGATTGTATCTGGATGCTTACGTTCAGGCAACTGCAAGGCTCATCTTTGTGAGTTCTT
TTATGAGTTTTCTAATGGAATTGGTGTTATGATCCCTTTAATGATCATTTCAG**TATTGCATTCATACTTTGGGAGAAAAGACTATCGCTCATGATAAGAAGTTGCGTCTC**
TATCAATTGCTGGATAGTATGGAGACAATTATGGAAGGACAGAAATACGTTGCAGGAGATGAGATTAGTCTAGCTGATCTTGCTTTTCTGGCTTCCTTCTCTACCCTTTTAG
TAAGCTAAATAAATTTTTCTACTAGCGGTTTCTTATCAACATCGTTTGTTTTCAG**TACGCTGGGGCCAATGTTGAGAAATACAAAAACATTCAAGCCTGGTACAAAAGAT**

GCGAAAATGTTCCTGGCTATCAGGAGAATGATCAAGCTGCTAGAGCTTATGGAGATTTCCTTAAGGGTCTGTTGGGAATTACTGGAACCTGGGATTAAAACTGACTAATTC
TGCATTATAATTATTTTTTTCAAATGAATTTAAGTGTAGCGTTTTTATCAGTTAATTGTCTAATCTAATTTGATAAGAGATGTGAAAATGAAAGTGAAAAGTAATTTT
GATGTACAATTTCTTCAAGGTCATGATTATGTAACTCACTAATAAAAAAGTTGATTTCTTTTCAAATGCTTTTTATTGC

>PPAI010872 (220 aa; 3 exons) | UNCHANGED
MAPLKFYYFPASPPCRGALLAIRYLKLDVEMISLNLREKEQLKPEFLKVNPTHSVPTLDDNGFVLWESRAISQYLVATRAPGSSLYPDDPKKRAIVDARLYLDAYVQA
TARLIFYCIHTLGEKTIAHDKKLRLYQLLDSMETIMEGQKYVAGDEISLADLAFLASFSTLLYAGANVEKYKNIQAWYKRCENVPGYQENDQAARAYGDFLKGLLGIT
GTWD-

####### LLOJ001842 - GSTX #######
>JH690728 reverse | **LLOJ001842** (pre-editing) | LLOJ001842_a, LLOJ001842_b, LLOJ001842_c, LLOJ001842_d, LLOJ001842_e, LLOJ001842_f, LLOJ001842_g (post-editing)
TATTAAAGAATGAAATTTGAAGCTTTTAAACTTCAAACTTTTGAGTCATATTGGTTATTTTGTCAAGCCAAGAGTACAAGGGCTAAAAGAGAAAGTTTCAATGATTGG
CCCTAAGGCCAAAGTGTTCAATACAAGGTTCCCTCTCTAAATTCTATAGTGATTTTTTTTTATACTCTTCTATCACTAATTTAATCAGCAACATGGCCCCAGTGAAGTT
CTACCATTTACCATTAGGCCCCCCATCTCGTGGGGTTCTTTTGACCATTCGGAATTTGAACCTCGATGTTGAGGTAAGATTTTTGTAAAATTTATTTAATAAAAAATATTC
CCAATAAATTAATTTTTGCATATCATTTTCAGATTATTGAGGTTGACATCTTCAAAGGGGAGCATTTAACCCCTGAATATTTGGAGATGAATCCTCAGCACACCATTCCAG
CTCTCAACGATAATGGCCTTTACTTGGGGGAGTCCAAAGCCATTTCCACTTATTTGGTTAATTCAAAAGCCCCCGGAAATCCGCTCTACCCAACTGATCCAGCTGTACGAGC
TCAGGTGGATGCAAAACTCTACTTTGATGCTGCCACAATATTTCCACGAATGAGAGCAATCTTTGTGAGTTCTTAAAAAAAAGGAAATTTAAAAGATTTTCATTGTTAAT
TTATGATTTTATTTGTTTTCAGTTCCCTATTCTTTTCCTTGGTTCGAAAACCATTGAAAAGGAGAAGAAGGAAGCTTTCTATCAGGCCCTTGATTTTATGAATACCTTCTT
GGAGGGCCGCACGTGGTTTGCTGCAGATCATCCCACACTCGCTGATTTGGCTCTCTTGGCTTCTTTTTCAACATTTGTCGTGAGTTTTATGAATTTTAACTTGAGGAATTT
ATTAAAGAATTGTTTAAAAATTATTTAAAAATTTTCAGTATTGTGGAGCAGATGTTTCGAAGTATACAAACACCCTAGCTTGGTACAAACGATGTGAATCCTTGCCTGGAT
TTGAGGAGAATGAAGAGATGGCTAAAAAACTAGGAGGACTTGTGAAGGAGAAGCTCGGCATTACTGGTTATTGGGAATAATTTTATGTTTCTTTTACAAATTCATTCAAT
ATTTGAGATAAATTCATTATCATTCCAAACATCACAATAAAAAGTGTTTTGAGAAAACAGACTGGGTTTTGTCTTTAATATTCGTTTTTTCGTTGCACTGTATATAAA
AATCCTGTTTCATTAAACTTTATTTCTTACGCTTAATACCTACCTTCTAAACAGACGCAATGGCCCCTCTGAAGTTCTACCATTTTACTCTTGGTTCTCCCTCTCGTGGT
GTCCTTTTGACCATCCGGAATTTAAATCTCGATGTTGAGGTAAGATTTCCTGTTTTTTTCAATATTTGATTAAACATTTAATATAATTACATGTTTTACAACTAGATAAT
CGAAGTGGATACCGTGAAGGGCGAGCAAATGAACCCTGACTTTGTGAAGATTAACCCCCAGCACATTCTCCCCACAATCGATGACAATGGCTTTGTTCTGTGGGAATCTAAA
GCCATTTCCACGTATTTGGCCAACTCAAAAGCCCCTGGAAATCCTCTCTATCCAACTGATCCAAAAATCCGAGCTCTTGTGGATTCTCGCATGCAATTTGATTCGGATAGTT
TATATCCCAAAATCAGAGATAATTTTGTAAGACAATTTTGAGATTTTTTTTGAGATAGAGAATTAATTTTTAAGCTTAATTTCTTGCAGTTTCCTGTCCTCGTAAGTGGC
GTAAGAAAGATTGAGGAAGAGAAGAAACAAGCAATTTATCAGACATTAAGTTTGATGAATACTTTCTTGGAAGGACGTCAATGGTTTGCTGCAGATCATCCCACACTC
GCTGATTTGGCTATTTTGGCATCATTCTNNNNNNNNNNNNNNNNNNNNNNNNNNNNNNNNNNNNNNNNNNNNNNNNNNNNNNAATGCGGTCAATTGTCGAATGGAGGGGGCT
GGCTTGCACGAGTGTTACAGCCCATTAGTCTGGGAAGCGACGCAAAGCATTCTGGATCATCTGTGAGGGCTTGGGCGGTTTGTGTGTATTGTGTTATGGCGTGCTGGT
AGTTTGACCAATTTTGATGTGCAATTCTTCGTTTTATAGTATAACGGTGTCGTATTGCTGAAGTGAGTTTGTATTTTTATGCGCTTTTTGAATGTTTTTCCCAGATTT
ATGTCACTATGTTGGTACTAAGATGTACATATTTTGTTGTAGAGCCTGATGATGGTTTAAAATAAACCGAAACGTCGCTGAAAGCGTGAAGACGTGCGTTTTTATTGA
CTGTCTCCGATCAAAAAACAACATAACTTAAAAAACAGTCGTTAAATTCTTCAATTAGAGAATTAATGTTTTAGCTTAATTTCTTGCAGTTTCCCGTCCTCGTAAGTG
GCGTAAGAAAAAATTGAGGAAGAGAAGAAACAAGCAATTTATCAGACATTAAGTTTGATGAATACTTTCTTGGAAGGACGTCAATGGTTTGCTGCAGATCATCCCACAC
TCGCTGATTTGGCTATTTTGGCATCATTCTCAACTTTTGTGGTAAATTTGAACCTCTTCTTGTGGAGAAGAATCAAAGATTAATTTTCTGGATGATTTATTTTCAGCA
TTTCGGAGCTGATGTTTCAAAGTACAAGAACATCTTGGCGTGGTACAAACGATGTGAATCCCTTCCAGGATTTTCTGAGAATGAAGAAGGTGCTAAAGAGTACGCCAGGATG
GTTAAAGAAAACTCGGAACTTCCGGAATATGATTCATCGCGAATGTGAATCTTGTTTGAAAATTTGTTAAAAAACATTTTCAATAAACATTAAAAAAATTCATTAAATATT
TCTTTTATTTCAATCAAACTTTTGTTGATTGTCAGACTAATTTTTTATATAAAAATTCGATGATTTATGTCTCTTAACGGTCATAACAGTCATTAAAGTTAATAACCA
TGACATTACATAATTCACAAACAGCATTTTCCATAAATAGACTGTCTTATCGTATTTGTTTTAGTTTTTTTCTAAGAGTCTTTTTTGAGAGTGTTTTTAGATAATAAA
GGGTTATCACAGAGCGGAATTCCTTTACGATATATCAAGTTGAATGAGAAGTTAAACTTCTCCCCCACATCATTCAGTCTCGAGGAAAGCTTCAAGCGAGAGTGAACT
TCTGCATAATTAATTTTTAATTTCGGCGTTCGTGAATTAAAGTTAGTTTGGGTGTTTAAGTCTTTAATAATTTTCATTTAATCTTTTATATTGCAGATAATGTAATTT
TGTAATTAATAATTTATTTTACAGCTTCGATTTTGTAAAATTTCTTGCAAATTTTTCTCAATTTCTTCTTCCTAAAATTCTTTTATAATTTTATTTTCCCTAACACTT
CTTGCTTTTTCTCTAATTTTCCGATACTTTAGGAAATCCAACACAATGGCTCCTTTGAAGTTGTACTTTTTGGAAATTGGTCCTCCATCTCGTGGAGCTCTTCTTACTATC
CGAAATTTGGGTCTCGATGTTGAGGTAAATGAATAAATCAACTAAAATAATTAATTTAAATTAATAAAATAAATTTCTTCGTAGATCATCCACGTCGATCTTTTCAGTGG
CGAACATCTAACGCCTGAATTCTTGAAGATGAATCCTCAGCATACGGTTCCAACTCTCGATGACAATGGCCTCTTCTTGGGTGAATCAAAGGCCATTTCCACCTATTTGGTG
AACTCCAGATCCCCCGGAAATCCTCTCTATCCAACTGATCCAAAAGTTCGTGCTCTCGTGGATGCTCGTTTGTATTTCGATACAGCCACTGTCTTCAACAGGATCAGGAATA
TCACGGTTAGATGAATTATGCTCAGCATTGCCAGGAAGACATTCTAAGAATTTATTTTTTTGTAGTATCCTCTTCTTGTTCTTGGAAAGAATGTTGTGGATGAAGAGAAG
AAGAAAGCTCTCTATGATGCTCTCGATCTCATGGAAATCTTCCTTGAGGGACAAAAAATGGTTTGCTGCTGATCATCCCACTCTTGCTGATTTAGCTCTATTGGCTACCGTTT
CGTCCCTTCGGGTAATTTCTTTAGAATCTTTAGCAGTTTGTGAAAAATTGATTAAACTTCGGTGGTATTTTTGTTGCAGTACTTTGGATTGAATGTCCCGAAATACAAGA
ACATTATGGCCTGGTACAAACGTTGTGAATCCCTTCCAGGATTCTCTGAGAACGAGGAAGGAGCTAAGAAATACGGAGCTCTTGTTCAAGAGAAGCTTGGCAAGGGCTT
CTCATGGGATTAAAGAATACCTTTGAATCTTAAGCTCTTAAAATTGACTTTATTGTCTTTCTTGGATTGAAATTAAAAAATTTAAAAATTTAAAAAATCTTTTTTTCT
TAAGAAGTTAAATTAAAACCTGAAATTTAACGACGATTCTATTCACCCCGTTATCTTGTATATAAAACACGCACAGCCAAAAAATATTCAGATAAATAATTCATAAAT
TTCTTTAATCAACTTTGCCGTCAATTTGATTTTCTAATCTCTCCAGTGACATAACACATTTTACTTATCTTTGCTTTGTTGTAAAAGAACTTTAACAAATACTTGAAC
GAGTGACATGCGTGTGGGTATCTTTTGGGAGATTTTTTTTGCGATTGATTGCGTTATTTCTGTCTCACTTCTATTTCAATATTTAACATCTTTCACGAAGTTTTTCCT
ATTTATTCAGTTTTGACTGAGGCGCTGAACGAGAAGGTGCTGCTGTTCTAATTCTGTTCAAATTCTGTTTAAATTCTGTGCTTGTGAGATTGAAAGTGAGGGTTTCTT
TTGGCCTTTAAAAATAAATTCTTTCCAATCTTCTGGGTTGAGAAGTTTATTATCTCAATCTTTAACATTTTTCAATCTAATTCTTATCTAATTAATTTTCTTTAACTT
TCAATAAATTTTCTAATAAATTCTGTGAATTGCATTTTTTTATTTTCCAACATAGCTTTGAACCAAACACAATGGCTCCACTGAAGTTGTACTACATCGAATTAGGACCT

CCATCCCGTGCTGCTCTTCTGACCATTCGCAACTTGAAGCTCGACGCAGAGGTAAGTAACGAATTTTCAATTCTCTTGACGATTTAATGAAAAGAAATTTTCATTGAAAA
G**ATCATCAATGTCGATGTAATCAATGGAGCTCATAGGACCCCAGAGTACCTCAAGATGAATCCTCAGCACACAGTTCCAACTCTCGATGACAATGGCTTCTACTTGGCTGAC**
TCCAAGGCAATGTCAACGTATTTGGTGAACTCAAGATCACCCGGAAATCCTCTCTATCCAACTGATCCGAAAGCTCGTGCTCTTGTTGATGATCGTTTGTACTTTGATGGAG
CTACTGTTTTTCCCAGAATTAAGACAATCAGCGTATGTTTTACAGCAACAATTTTCCTAATAATTTGAGAGTAATCTTTGTTTTATTTTTTTTTGCAG**TATTCGGTTCTG**
GTTCTTGGCGTGAATAATGTGGATGATGAGAAGAAGAAAGCTCTGTATGAAGCTCTTGATTTTATGGAGATTTACTTGGAAGGACAGAAGTGGTTTGCAGGTGATCATGCTA
CCCTTGCTGACTTGGCTCTTCTGGCTACATATTCCTCTCTGGTGGTAAGGATAAAATATTTTCTAGAAAATATTCACCTTCTTAGTAAATAATTATACTTAAATTTTGAT
ATTTTTTGCAG**CATTTTGGATTGAATGTCTCCAAGTACAAGAACCTTCAAGCTTGGTACAAGCGCT**GTGAGGCTTTGCCAGGATTTGCTGAAAATGAAGAAGGTGCTAAG
AAGTACGGGGCAATGATGAAGGAAAAGCTTGGAAAGAATTTCTCTTGGGATGTATAGAATTTATTTCCTCAGAGTTTGCTTTGATTTCTTGTAATGAAAGATTGAATA
AAAATCAATGAATGAATTCAATTGGATTAATGAAATTGTAGAATGGCGTATTCTTATCTTTGAGCTCCTCTATCGCGCATTTTAAATACGTGAACAGATAGAAGGTTT
AGCAGATGAATCATCGATATCTATAGTTTTTTTCCTTTAATTATTTGCGAACTCATTTTAATAAATTACTGCCTTAGAGACTTATCAAATGTCTGCCAATCATCTTTA
CAAATAATTTTCATTAAATATTTTTTTAGAGAATTCTTAATCATTGTTTTGGATGAAAGGGTCATCTTCGAGAGAGATTTTTCTACTCTAGAAAAAAAAATATATGTTT
TTTTGGCTACACACTAATCTACTTCCAAGACTAAAACGTTCTTATCTCAATGATATGTACCATAATTTAGATTTTTCTGCAAAAGAGCTTTTCCAGAGTACTTTAGC
AAATGATATGTCTTATCATTGAATTGAATTTGATCTCAATTGACGTTATATGTGTGCCTCATTTTTTATTTACATATATTTTGGTATATATTTCTTTTTATAATCAAA
TTTAGTCTTAAGTCGAATCTCAGCGAAGACAGTGCTATTTAATACTCGTCTGCAATTTAGTGTTAAAGAATTTAAAATTAAAATTAAATTCTTAAAACTGCTATAAAA
TTTATTTTAATTGCTTTTTATTAATCTGGATAAACAG**ACCAAATGGCCCCACTGAAATTCTACCACATGCCAAAAGGACCTCCTTCCCGTGCAGTTGCCTTAACGATTCGA**
AATCTCAAATTAGATGTTGAAGTAAGAATTAAACGTTTAACCTTAATTTAATTTAATAAATTTTTTAAAACAAATAATTTCTAG**AAAATTGAAGTTGATGTGTTAAATGG**
CGAACAAATGTCTCCGGAATTTGTAAAAATTAACCCTCAACACACTGTTCCCACAATTGATGATAATGGATTCATTTTGTGGGAATCTCGAGCTATTATGACGTATTTGGTT
AACTCCAAAGTTCCTGGAAATTCTCTCTATCCAACTGATCCCCATATCCGAGCTTTAATAGATGCTCGTTTATATTTTGATACCTCAACTCTATATGCAGCCAGGGAGATCA
TGGTAAATCTTTACTTTTAAATTTTTCTCAATTAATCTGTATTTTTCATTCCTTTGTTTTTTTCAG**TTTCCTATTCTTTTCCTTGGTGCAAAGACAATTAATAAAGAGAA**
GAAAGAAGTTTTCTATCAAGCTCTTGATTTTATGAACACTTTCCTAGAGGGCCGAACGTGGTTTGCTGGAGATCATCCCACACTCGCTGATTTATCAATTTTGGCTTCTTTT
TCAACTTTTGTGGTAAGATAGAAAATTACTGATTTTTTTTCTAAAATGCATAAAACCGATAAATCTTTATCCAAACCTAACTTTTACATTTTAAATTAATTTCAG**TACG**
CTGGAGCTAACGTTAGCAAGTACAAAAACATCCTAGCTTGGTACAAGCGGTGTGAATCTCTGCCAGGATTCGATGAGAACGAAAGTGGAGCCAAAGGATTCGGGAACGTAG
TCAAGGAAAAACTCAGCATTACTGGCACATGGGATTAAACGCCGATTGAATCTCCGAAAGAGAGATTTCTCTAAAAAAGTTAAAGCCTCTTTAATCTGTATGAAGTCT
GAGTATTGAGTCTTATCTTAATGACACCACAATTTTAGATTTTCAGCAAAAGAGCTTTTCCAGAGCACTTCAGTGGATGATATGGTGTTATCATTGAATTAAATTTCA
TCTCAATGGACGTTATATTTCTGTCTCACTTACATTTCTATTTTAGTAAAACATTTTTCACGGTCAATCATTTAGTCTTGAGTCAAATCTCAACGAAGACAGTGCTAC
TTCTGCAATTGAAAGTGTGGGTTGAAGATTAATTCTAAGAGTGAAGGTCATTTTATTAACATTGTTTAGTGTTAGTGTATTTTGTTTGATTTAAAAAAAATAAGAATT
CCCATAAAACTTATTGGAATTAATTTTCTAATCTACCAAATTAG**TTAGAATGGCCCCTGTAAAATTCTATCATATGCCAAAAGGAGCTCCATCCCGTGCAGTTGCCTTAA**
CCATTCGAAATCTCAAACTCGATGTTGAAGTAAGAATGTAATATTGAACCGTAATTTAATTTAAGAAATTAATTAAATTCTTTAATTCCTAG**ATAATTGAAATTGATGTC**
TTAAATGGCGAGCAAATGTCTCCGGAATTTGTAAAAATTAACCCTCAACACACTGTTCCCACAATAGACGACAATGGATTTATTTTGTGGGAATCACGAGCTATTATCACTT
ATTTGGTTAACTCCAAAGCTCCTGGAAATTCTCTCTATCCAACTGATACCCAGATCCGAGCTACAATAGATGCTCGTTTATATTTTGATGCCTCAACTTTGTATGCTAGAGC
GAGGGAAATCATGGTAAGACCATTAAGCTTTTCCTAAATGAATCAATCTTTTTTTTAATTAAAAATTATTAAATATAAATTGAATTCTTAATAATTTCCTTTAG**TTTCC**
TATTCTTTTCCTTGGCGTTAAGACGGTGGATGAGGATAAGAAACAATCCCTCTATCAAGCATTTGAATTAATGGATACTTTCTTAGAAGGCCGAACGTGGTTTGCTGCAGAT
CATCCCATTCTTGCTGATTTATCAATTTTGGCTTCTTTTTCGACATTTGTGGTAAGATTAAGATTAAATTCAAGACTAATTAAGATTTTGTGTATCGTTTAACATCTCTT
AATAGATTTTTATAAAAGGAACATAGTTAGAAAAGTAACCGATTTTCTTTTCAAGAGTGATTAATTCACAAAAATTAATTATTACCTGATATATTTTTTTACAATTAA
TTTCAG**TATTGTGGAGCTAACGTTAGCAAGTACAAAAACATCCTAGCTTGGTACAAGCGGTGTGAATCTCTGCCAGGATTCGATGAGAACGAAAGTGGCGCTAAAGGATTTG**
GTAAGGTGGTTAAGGAGAAAATCGGCATTACGGGTACATGGGATTGAGATGCATTAATATTCCATCCGTTATATGTTTTCATCATGCTCAAAAGTATGTTTGTTATGCTG
TTTCACTCAATATCGATTAATCGATGAATCGGACCGGTTTGTGTCTCGGGTTGTGCTCATCGTTAGATGGTTCTAACGTTATGTGGAGAATATACATATAAGAAGAAA
ATATAAAAGAGATAAGCAAGAATATGTAG

>LLOJ001842_a (221 aa; 4 exons) | EDITED
MAPVKFYHLPLGPPSRGVLLTIRNLNLDVEIIEVDIFKGEHLTPEYLEMNPQHTIPALNDNGLYLGESKAISTYLVNSKAPGNPLYPTDPAVRAQVDAKLYFDAATIF
PRMRAIFFPILFLGSKTIEKEKKEAFYQALDFMNTFLEGRTWFAADHPTLADLALLASFSTFVYCGADVSKYTNTLAWYKRCESLPGFEENEEMAKKLGGLVKEKLGI
TGYWE-

>LLOJ001842_b (>167 aa; >3 exons) | EDITED (PARTIAL - C-TERMINAL MISSING)
MAPLKFYHFTLGSPSRGVLLTIRNLNLDVEIIEVDTVKGEQMNPDFVKINPQHILPTIDDNGFVLWESKAISTYLANSKAPGNPLYPTDPKIRALVDSRMQFDSDSLY
PKIRDNFFPVLVSGVRKIEEEKKQAIYQTLSLMNTFLEGRQWFAADHPTLADLAILASF

>LLOJ001842_c (>109 aa; >2 exons) | EDITED (PARTIAL - N-TERMINAL MISSING)
FPVLVSGVRKIEEEKKQAIYQTLSLMNTFLEGRQWFAADHPTLADLAILASFSTFVHFGADVSKYKNILAWYKRCESLPGFSENEEGAKEYARMVKENSELPEYDSSR
M-

>LLOJ001842_d (221 aa; 4 exons) | EDITED
MAPLKLYFLEIGPPSRGALLTIRNLGLDVEIIHVDLFSGEHLTPEFLKMNPQHTVPTLDDNGLFLGESKAISTYLVNSRSPGNPLYPTDPKVRALVDARLYFDTATVF
NRIRNITYPLLVLGKNVVDEEKKKALYDALDLMEIFLEGQKWFAADHPTLADLALLATVSSLRYFGLNVPKYKNIMAWYKRCESLPGFSENEEGAKKYGALVQEKLGK
GFSWD-

>LLOJ001842_e (222 aa; 4 exons) | EDITED
MAPLKLYYIELGPPSRAALLTIRNLKLDAEIINVDVINGAHRTPEYLKMNPQHTVPTLDDNGFYLADSKAMSTYLVNSRSPGNPLYPTDPKARALVDDRLYFDGATVF
PRIKTISYSVLVLGVNNVDDEKKKALYEALDFMEIYLEGQKWFAGDHATLADLALLATYSSLVHFGLNVSKYKNLQAWYKRCEALPGFAENEEGAKKYGAMMKEKLGK
NFSWDV-

>LLOJ001842_f (221 aa; 4 exons) | EDITED
MAPLKLYYIELGPPSRAALLTIRNLKLDAEIINVDVINGAHRTPEYLKMNPQHTVPTLDDNGFYLADSKAMSTYLVNSRSPGNPLYPTDPKARALVDDRLYFDGATVF
PRIKTISYSVLVLGVNNVDDEKKKALYEALDFMEIYLEGQKWFAGDHATLADLALLATYSSLVYAGANVSKYKNILAWYKRCESLPGFDENESGAKGFGNVVKEKLSI
TGTWD-

>LLOJ001842_g (221 aa; 4 exons) | EDITED

MAPVKFYHMPKGAPSRAVALTIRNLKLDVEIIEIDVLNGEQMSPEFVKINPQHTVPTIDDNGFILWESRAIITYLVNSKAPGNSLYPTDTQIRATIDARLYFDASTLY
ARAREIMFPILFLGVKTVDEDKKQSLYQAFELMDTFLEGRTWFAADHPILADLSILASFSTFVYCGANVSKYKNILAWYKRCESLPGFDENESGAKGFGKVVKEKIGI
TGTWD-

####### Ll cluster of GSTX genes on JH690204 #######

####### LLOJ009529 - GSTX #######

>JH690204 reverse | **LLOJ009529** (pre-editing) | LLOJ009529 (post-editing)
TCAAGTGCTGAGCACCCGGTGGATGCGACTCTGCCTTCCCAAACTCTTCAAATTATCTTCCGTCAGAGCCGCATTCTCCTTCTGTGTGTTGGTGTTGTGATCTCTGGT
TAATAGTGAAAGTCAGTCTGATAAGTTGTTGATAAGGGCGTGAAAACTCATCTATTTCTTCCACTAAAAACCTTCCTGGAAAATTCCTCACA**ATGGCCCCAATGAAGTT**
CTACTATTTCCCAATGAGTCCACCCTGTCGTGGAGCTCTCCTGACCATTCGCAACTTAAAGTTGGACATCGAGTTGATTAATGTTAACTTGCGCGAGAAGGAGCAACTCAAG
GAGGATTATCTGAAAGTCAATCCTGTTCATTCAGTGCCCACATTGGACGATGATGGCTTCATCCTTTGGGAATCCCGAGCTATCTCTCAATATCTCGTTAGTGCAAAAGCTC
CCGGAAGTCCCCTCTATCCGGCTGATCCCAAGAAGAGAGCTGTTGTGGATGCTAGACTCTACCTCGATGCTTACCTTCAGGCTTCTGCAAGGATTATCTTTGTGAGTATCC
TTCGATCTACTCGAGATCATGCAGAAGTTGTCATTTTCTTACCCTTCCTCGGGTTTCAG**TACTCCATCCATACTCTGGGCCAGAAAACCATTTCACACGACAAGAAAGTC**
CGCTTTTATCAACTCCTAGAGAGCATGGAGACAATCCTGGAGGGACAGGAATATGTTGCAGGGAATGAGGTAACATTGGCTGATCTTGCTCTCCTAGCTTCGATCTCCACGT
TTATTGTAAGTCTATAAATTTTTGAAACTTTTTTTTGATCTTCCGGAATGTTATTCAAACTTTGATGCATTTGCAG**TACGCAGGAGTTCCCATGAGCAAGTACAAGAACA**
TCCTGGCGTGGTACAAGCGATGTGAAAACCTCCCAGGTTATCAAGAGAATGACCAAGTAGCCAAGGCCTATGGAGACTTTCTGAAGAATCTTCTAGGGATCACTGGAACATG
GGATGATGTTCTGGAAGCCTAAAACACTTTCCTTGGATTTCATTCCAAATTAATTTTTTTTAAATATTTTTTTATTGCATTTTTTGGACTCTTTCTGAATGAGATAATA
AAGGAATTGGATGACTTAATAATTTTTTTAGTTAAAGTGATTATAGAACAATACAGATTATTCATCCCAATGATCTTTTACGTTGATTTTGGATTTAAACATTTGCCC
AAAAG

>LLOJ009529 (225 aa; 3 exons) | UNCHANGED
MAPMKFYYFPMSPPCRGALLTIRNLKLDIELINVNLREKEQLKEDYLKVNPVHSVPTLDDDGFILWESRAISQYLVSAKAPGSPLYPADPKKRAVVDARLYLDAYLQA
SARIIFYSIHTLGQKTISHDKKVRFYQLLESMETILEGQEYVAGNEVTLADLALLASISTFIYAGVPMSKYKNILAWYKRCENLPGYQENDQVAKAYGDFLKNLLGIT
GTWDDVLEA-

####### LLOJ009528 - GSTX #######

>JH690204 forward | **LLOJ009528** (pre-editing) | LLOJ009528_a, LLOJ009528_b (post-editing)
TTTTCAGTGTAAAAATGCTATTTCATTCCGCTGAGACTGACTGCTGATTAGAGCGAAACTGCAGATAAAAAAAAACTCATTCTAATCAAAGGGACAGAGAATGATGTA
GCTCAATATAAAAATCGTTCCAATTATTTCCCCTACATTCAGTTTTATTCCAACTACCGCAGAAGGTGAACCTCTTTCCCGCGCAAATTGAA**ATGGCTCCACTTAAGCT**
CTACCACTACCCCATGAGCCCACCATCGCGATGTGCCCTCCTTACGGTGCGAAATGTGAAAGTTGACGCGGAAGTTATTACGGTGGATTTGTTCAAAAAGGATCAATTGGCT
CCGGAATTCGTGAAGATCAATCCTCAGCACACAGTTCCTACAATTGATGATAATGGGTTTGTTTTGTGGGAATCTCGTGCTATTGCCACCTACTTGGCTTCTACTAAAGCTG
CAGGAAGTTCCCTCTACCCAAATGATCCCAAAAAACGCGCCATTGTTGATGCCAGGCTCTACCTCGATCAGGCTTTGCAGATTGCAACAGGAGCCATCATGGTATGTTGCG
AAGATTTTCATTGAGAAAAAAGAGATTTTTTTTGTGATTTTCTGAACTATTTTCCAG**TACCCAATTCACGCAGAAGGAGCTACATCAATCCCACAGGATAAGAAGGATAA**
AGTCTACAAAATGCTAGGTGATCTGAACACCTTCATGGAGGGAAATTCATACGCAGCCGGCAATGAGCTCACTGTGGCTGATTTAGCTCTCTTAGCCACTATCTCGACAGCT
CATGTAAGTTTATCTAATAGTACTTGATGTAAAGGATTATTGCTCAATCACTCCGATGGATTTATCAATGTTGATTTATGTTTTCCCCCACAGGGTATTGGGGCTAAT
ATCAGCAAGTTTAAGAACATCGCAGCATGGTACAAGAGACTCGAAACCATTCCTGGTTTCCAGGAGAACGAAGAAGGAGCTCAAACTCTCGGGAAGTACATGAAGGCC
AAGATTAATCTCATCGGAACTTGGGATGATTAGAAGTTAAATTTTTAAGAATTTATATTCAAATAATTTCGTAATAAAGATAAAAGAATCGTAACAGGTGCCTGAATA
TAGAACACCTGATACTGATATATTGATTACGTTGTTATCATTCTTGTCGTACTGAAGAGTTTTATTTTCTATCCCTTATCAGTCGAATGATTGGGAATGCTTCATATT
GTTTTTGTTTTGTTTTATGCAGGGTTTTATGTAGAAGAATAACATTTTAAAAAGTAGGCAATTCTAAATTATTTAAGTTTTAAAAAAAGAAATTAAAATTTCTAAGTT
TTTTTTATTCATTTGTTAGAAAGCTTGTTGATAAGCAACTTTTGAGAATACTTAGAAAATTCCTAAGTTAGATATGACTGATCCTAATTCGAATTTAAGAAAGAAGAA
GGTCAGACCTATATGGCCTATATAACATTACATAATAGCGAGGGCTTCATTGTAAAAACGACTAAGTCGGTTCGGAGCTCATACAAAGTTAGCCGGTTTTACAATCTA
GCCCTCGCTACGCTTAATCACAAAAATGCTTAAAATGCATGAAATATTAATTTAAGATTAAAAAAAAAAAGAAAACAGAAATAGGGACTAAGCCTTAATCAGGTCTGA
TCAGGCTATTTAGTAGCTCAGCCATAACTAAGTTCCTAAGAAAAAATCATTTAAATCAAGATCTTTTTTGAATATTTTTCTCTTTGTGCGCTTCTAAAGAACAGACAG
TCTTGAATGATTTATTTATTTTTATTTATTTTCTAATAGATAAAAGTACATTTTCTTCAAGTACACCTGATAACATTAGGCACGTTTTAGGTTCCCCTTGATCCACCC
ACTAGTCGTGACTTGGAAACTGTTGGACGTGCAACTAGATAAGATTCACTTTCAATTTCCTCGAAGATGGCTCCCTTTAAATATTATTACTACCCCGAAGGAGCCCCC
AATAGGGCTGCTTTACTAACTATTCGGAATCTTAATTTGGATGCTGAAGTAATTTTTGTGGATCTCCGAAAAGGAGAACATCTAACGGAGAAATTTGTAAAATTAAAT
CCCCAACGGTGTATGCCGCTCTTGGATGATGATGGCTTCATCCTTTGGGAATCAAGAGCAATCTCACAGTATTTAGTGACAACAAGAGCTCCTGGGAGTCCCCTTTAT
CCCACTGATCCCAAGAAGAGGGCTACTGTTGATGCCAGACTTCATTTGGATTGCACACTGCACGCAATTTCTAGGTCTTTTTTCGTAAGTTTCTTTCTTAAAATCCT
ACACTGAATCGTTAGAATTTAATAATTAGGACCTTCTCTTATAG**TATCCCATTCATTCTTTGGGTGAGACAAAAATTCCCGATCCGCCGAAGCAGAGACTCTACAAACTT**
CTTGGACATTTGAACGACATGATGGAGGGTCAGAAATTTGTAGCAGGAGACGAACCTACAATTGCGGACTTTTCACTTCTAGCTTCCTTTTCAACCTACTATGTAAAAAAT
AAGTTTAGAAGATTAAAACAGAAAATATTCTTACGATGGATTTTTCTTTACAG**CACGCTGGAGCTAATGTAAGAGATCTGAGGAATTTGATGGCTTGGTATAGGAGATGC**
GAAGCCCTACCTGGATTTCAGGAAAACGAAAAGGAAGCTCGAGCTTTTGGGCAAATGTTTAAATCCAAAATCAGCGTAAAAGATCATTGGGATGAATAACCTGTATTGTTC
TTTAATTTTTTTGTTAGAATTTGTCAGAAGACTTCTTGATCTGTTTTCTCTTTTTTCAAGACTTAATCACTAGCCTTCGACCCTAATAGCGGGTCTTCTTCAGTGGTC
TTGCTTGTCATTTCTTGGAGATTGTGCTCCTCCTGGAATGTTCCACACACTAGGGTGGATTTATTAGGAAGCTCGAGCTT

>LLOJ009528_a (221 aa; 3 exons) | EDITED
MAPLKLYHYPMSPPSRCALLTVRNVKVDAEVITVDLFKKDQLAPEFVKINPQHTVPTIDDNGFVLWESRAIATYLASTKAAGSSLYPNDPKKRAIVDARLYLDQALQI
ATGAIMYPIHAEGATSIPQDKKDKVYKMLGDLNTFMEGNSYAAGNELTVADLALLATISTAHGIGANISKFKNIAAWYKRLETIPGFQENEEGAQTLGKYMKAKINLI
GTWDD-

>LLOJ009528_b (221 aa; 3 exons) | EDITED

MAPFKYYYYPEGAPNRAALLTIRNLNLDAEVIFVDLRKGEHLTEKFVKLNPQRCMPLLDDDGFILWESRAISQYLVTTRAPGSPLYPTDPKKRATVDARLHLDCTLHA
ISRSFFYPIHSLGETKIPDPPKQRLYKLLGHLNDMMEGQKFVAGDEPTIADFSLLASFSTYYHAGANVRDLRNLMAWYRRCEALPGFQENEKEARAFGQMFKSKISVK
DHWDE-

####### LLOJ010488 - GSTX #######
>JH690204 reverse | **LLOJ010488** (pre-editing) | LLOJ010488 (post-editing)
CTTTTGTGCCCACAATCCCGCCTTTTGGTTCCAATTTGGTGAATTTTAACTAAAAGTGAGAATTTTTTTTGTGGCTCAAGAAGTTATTTCTGGACATCAAGGCTAATT
TTTTTGGGTGGAAAATCATTAAAAGTGATTAACTAAAGATTTTTTCGTGTGATTTCTTTTTGCTAAAAAGCCAAAAAAAAGAAAGATTCGCA**ATGGCCCCATTGAAGCT**
GTATCATTATCCACTCAGCGCTCCATCCCGTGGAGCTCTCCTGACCATCCGAAATCTCAATCTTGACGTTGAGGTAGGTCAATTTTAACAAATTGCTTCCGCAGTGTGAAA
TAAAAGAAGATTTTTAAATGGGAATTTATGTCGACAGAAAAATTCTTAATATACAGGAAAGAATTTAAATTTTTGAACATTGACCAACAATGAAATATTTTTATGTTA
AGTTAAAAAAAAAGAGTCAGGAAGTAGTTAAACCGTCAAGTTTTAAAAGTTAAAATGTGAATTTGAGTTTAAATAATTTTATTTTTCATAATTCTTTCAATTGTTCTT
AATCTGATGAAAATATTTTTTTCTTTCTTTTTTTTAGTTCAGAATATTTTTCAAGTTTAATCAAGATATAGAATATTAAATTTCTAGCTCCTGAATATGGACATGGGCA
ATTTTGCTAAAAGCGACAAGAAAATCAATAAATTATGCTTATTGCCACCCATTTTGTTAATTTTAGACAAGAAGTAAGGAAAAGCCTTGAAAATTTATAAAAAATCAG
TAAGAAAAACTTCTTAAAAAAAGATGAATTAAAAGATCAATAAATTGATTTCTCTGCTGTTAAAAAAAGTTGTTACTTTATTAAATATTTATTATTTATTTCATTTCA
ATTCACTTTGTCACCTTAAATGTTTTTCTTTTCTTTTAAAAATAAATTATTGAAACGAAACAAAGATCTATCTAATTTTTTTTAATTCACTGTTCGATTTTTTTCATA
CCAAAAAATCCTTTTTTTTTCTGTAATATTTTCCACGAACTCAAAATCAATTTTTATTTGTTTAAAAGCTTCAAAAATTAAAAATGTTGTTTAGAGAACTATGGGAGT
TCAGTACCTACATATATAATGTAGTATATTTAATAGATAGATAGATAGATTGTTTATTTCACTCGAAACTGTGCATACAAAATCAAACAATTTTACATCTAGTAAAAA
AAAAAAAATTCCAACATGTTCTTTATATTCTTCTCCCTAATCAG**ATTGTCCAAGTTGACCTCATGAATAAAGCACATTTGTCTCCAGAATTCATCAAAATCAATCCTCAG**
CACACTGTCCCAACTTTGGACGATAATGGATTTACTTTGTGGGAATCTCGTGCTATTGCCACATACTTGGTCAACTCGAAAGCTCCGGGGAGTCCTCTGTACCCCACTGATC
CCAAAACTAGGGCCATTGTGGACTCTCGTTTGTATTTTGATGGCTCAAGTTTGTATGCAAAAGCCAGAGACATTGTTGTAAGTTTGATTCTAAAAAAAATCTGGTTTTCTC
TTTGAATTCTTTTTAATTTATTTATTTTTCTTTAG**TTCCCAATTTTCTTCATGGGCGTGACAGAAGTTGATGAAGCTAAGAAACAGGCCCTGTATGAAGCTTTTGGTGTGA**
TGAATACTTTGCTTGAAGGGCAGGATTGGTTTGCAGCTGATCATCCAACTGTAGCTGATTTGGCTCTTCTTTCTACCTTCTCATCTTTTGTCGTAAGTCTTATTTTATAAG
TTTAAGATTCTTCAAGAGCTTGAGAATATTAAAAATGAAATTTAAATATAAATTAATATAATTCCAG**CATGCTGGAGCAAATGCCAGCAAATACTCCAATTTGATGGCCT**
GGTACAAACGCTGTGAGTCCTTGCCAGGATTTGATGAGAATGAGGCTGGAGCTAAAGCTTTCGGGCAGGCAATCAAGGGAAAGCTCGGTATCACCGGCACATGGGATTAGTT
TCAGCCTAAATATCTGGCTTAAAACCAAAGTGAATTAAATTCCGTCCAATATCCTTAAAAATGCACTTAATCCTTTAATTGTTTCAGCCACTTTTGTGCTTTAATAA
AAATTAAAAAATAAATTTCTGTTTTTTATTAAATTTTCTTCGAATCTTTTATTTGAAATTGAGCTTCCAAAAAGTGCTTTGTCATTTTTT

>LLOJ010488 (221 aa; 4 exons) | UNCHANGED
MAPLKLYHYPLSAPSRGALLTIRNLNLDVEIVQVDLMNKAHLSPEFIKINPQHTVPTLDDNGFTLWESRAIATYLVNSKAPGSPLYPTDPKTRAIVDSRLYFDGSSLY
AKARDIVFPIFFMGVTEVDEAKKQALYEAFGVMNTLLEGQDWFAADHPTVADLALLSTFSSFVHAGANASKYSNLMAWYKRCESLPGFDENEAGAKAFGQAIKGKLGI
TGTWD-

####### LLOJ010539 - GSTX #######
>JH690204 reverse | **LLOJ010539** (pre-editing) | LLOJ010539 (post-editing)
AATACCTACGTACATATTACCTCATTTAATAGCCAAGTATTTAGTCTTGGGTGAAGCTTCAATTAGGCTGTGCGGCAATTTTCCTGGTTTTTATTTTTCTTAAAGAAA
GGTGAGTAAGAAGATTTTTCTTGGTCGTTTTTTTCTCTAGTTTTGAAAGCAATTTTTGAAAAGTTTATTGTTGGTTTTTCTATTCAGCTTAA**ATGGCTCCATTGAAGCT**
CTATCATTTTCCTATCAGCATCCCCTCGCGAGTGGCCTTACTGGCCATCCGGAACCTTAATCTTGATGTTGAGGTAAGTTTTTTTTTAATTAATTCATTTCCCATTAGCAA
AATCATAGACTGAGAATCTTTAACAATGAATAAAATCCCAATTTTTAAATAATTGTTTTTACTAAAATGTTTTTCAAAAATTTTATCAG**ATTATAGAATTAGATCTTAT**
GAACAAAGCTCAACTATCCCCGGAATTTCTTAAGATTAACCCTCAACACACTGTTCCAACTCTTGATGATGATGGCTTCATTCTGTGGGAATCACGAGCTATTGCAATGTAT
TTGGTGAACTCAAAAGCTCCCGGAAGTACTCTTTATCCTACTGATCCCAAGGTCAGAGCAATTGTGGACTCTCGCTTGTTCTTCGATGGAACATACTTGCATCCAAAGGCTA
AAGAAATCATTGTAAGTTTAATCATTTGAGAATATTTTTAAAATATCCCTTTAAAGTAATTTTAATTATCTAAAATTTATTGCAAAAAATATTTTTAG**TACCCAATTTG**
GACATTGGGCGTGAAGGAGATCGATGAAAGCAAGAAGCAAGCCCTCTATCAGGCTTTTGATTACATGAATACCTTTCTGGAGGGAAAGGAATGGTTCGCAGCAGATCATCCA
ACAATCGCTGATTTGGCGCTCTTGGTTTCCTTCTCGTCTTATTTTGTAAATTTAATATTTAAATGCAAATTCAGGATAGAATTAAAACCATTAATATATTTTTAG**CACGC**
TGGAGCGAATGTAAGCAAGTACACGAACTTATTGGCTTGGTACAAACGTTGTGAGTCCTTGCCAGGATTTGAGGAGAATGAGGCTGGAGCTAAAACTTATGGACAAGTACTA
AAAGGAATGCTAGGAATCACCGGCACCTGGGATTAGTCAAGTCAAGAGATTTGAGATTATTGTTTTTTTATGAGAATAAAATATTATTTTAAGTAAATCTAACGCCTTT
ACAATTAAATTTCTGTCGAAGCTGGCCTCAGTATACCTATGCTATATACATATCTATACACAGCAGAGTCGAACTTACTTATGATTTTATGATTTTTATCCCCATGAG
ATATTAAAATCAAATCGTT

>LLOJ010539 (221 aa; 4 exons) | UNCHANGED
MAPLKLYHFPISIPSRVALLAIRNLNLDVEIIELDLMNKAQLSPEFLKINPQHTVPTLDDDGFILWESRAIAMYLVNSKAPGSTLYPTDPKVRAIVDSRLFFDGTYLH
PKAKEIIYPIWTLGVKEIDESKKQALYQAFDYMNTFLEGKEWFAADHPTIADLALLVSFSSYFHAGANVSKYTNLLAWYKRCESLPGFEENEAGAKTYGQVLKGMLGI
TGTWD-

####### LLOJ010538-RA, *LLOJ010538-RB* (shared exon 1 italic, not underlined) - GSTX #######
>JH690204 reverse | **LLOJ010538-RA** (pre-editing) | LLOJ010538-RA (post-editing)
ACTTCAGTGTTCCTTCTTTCCAGTTCAATTCGGTGAATTTCAATCAAAAGTGAGTCTTATGATCATTCTTGGCTGACACAGAGAATATTCTTTAAGCTCCAAGGCCAT
TTTTTCTTCTCAAATCTGGCATCAAGGCAATTTTTGTGATGATTTAATGAATCTTTTTTGTGTGATTTCTCCTCCAAAAAAGCAGCCAAACA***ATGGCCCCACTGAAGTT***
GTACCATTTTCCAATCAGTGCTCCCTCCCGTGGAGCTCTTCTCGCCATCCGGAATCTGAAGCTTGATGTTGAGGTATGTTTAAATAATGCTCCATGGTGCATAAAGTAAAA
TTTGTAACTTTTATTGTGATTTAAATGAAAAGAACTTGTTGAGAAATTACGCAGCTGAAAGTTGAATAGAATTTCTCTGAATGGAGGTGAAAGTGATAATAGTTTTGC
GTAGGTTGGGCAGAGAGTAATTCATTTAAAAGGGGACAGAAAAATGAATAATTGTTCATTAACTCTACTCTCGTATTCTTTCTCCCTTAAGGGCATTGCCATTTAACT
CGATGGCATGACATGATAAGTAAAATGGAAGTTATCTTGAAATTTTATTTTATCTTCATCAAATAAGAGACAAGTTGAGGGTGAAGTGTCGAAATAAAATTAAACAAC

TCACCTTAGTAAATATATTTAAACTTGAAAACCCTAATGAACCATGCTTGCCCATCTAATACAAATTGATTTAATTTGAAATCTTAAAAATAGTTTAGATATCCATT
AAAATTTTCATTTAATTTAGTTAGAAGTGAATTGTTAAAGAGCCTTGACATTATCGAAGCTCATCTATGTTAAAATATTAAGTAGATTCACTAAACAAATGAAAAATA
ATCCATAAAGAAAACATAAAACTTCTCCCTCTCTACTCAAACAAGTCATCATAATTATTCAAATTGATCAGTTGATAAGTCCAAAAGAATGGGTATAGAAAATGTTAA
AAATAGATTTCACTCGCTTCTGTTCTTTTCCTGAAGCATTCTTTGCTTCATTTTATAACAAAGAATCTTTAGAAAAATAAATCAAGCTTAAGAATTGGCCTAAAAATT
TATACTCGTTCTTGTAGATCGTGGAAATCAATCTCATGAACAAAGAACATTTATCTCCGGAATACGTCAAAATCAACCCTCAACATACCGTTCCAACCCTTGACGATAATG
GCTTTATCTTGTGGGAATCTCGTGCTATTGCCACTTACCTTGCAAACTCCAAAGCTCCTGGAAATTCCCTCTATCCCACTGATCCCAAAATTCGTGCTGTTGTGGATTCACG
CTTGTACTTTGATGCCTCCAATTTATTCCCAAAAGCCCGGAATATTGTTGTAAGTTCTCTTCCTCTATACTCCATCTTTCTTCTCAGTTTTGTTTTACAAAATATATTAA
AGTTAGTCGTCTTTTAGTTCCCTATTCTTATCCTGGGAGTAAAGGAAGTTGATCCAGAGAAGAAGCAAATTTTGTATCAAGCTCTGGAGTTTATGAATACTTACCTAGAAG
GGCAGAACTGGATAGCAGCTGATCATCCTACTCTCGCTGATTTGACGCTCCTATCCTCAATTTCATCGATTTATGTACGTAATTGTTGGAGCAGGGTTATTTTCTTTTACA
GAGTTTGATAAATTTTTACCCAATCCTTAATTTTCCTAAATTTTTTACAGCATGCTGGAGCTAATATCAGCAAGTTTCCCAACATCATGGCCTGGTACAAACGCTGTGAG
TCCTTGCCTGGATTTGAGGAGAACGAAACAGGCGCCAAGGCTTTCGGCCAGGCAATTAAGAAGAATCTCGGGATCACAGGCACATGGGATGACTAAATCACCAGGAATTTG
TTGAACGCTGTCTTTTTATACTCTACCATATGCACTTGTACTTTTCAGAGATTATTTGCAAGAGTTGTTTAATAAATATTTTTTCTGGGATTAAAATGCTCTGGTTTT
GATTTTATTTTAATTTTTTTTTCAAACGAAATGAATGCAGAATTTAAAAAAATATTTCTTTTTAATTTTATTTAGATTGTAGAAGTTGATCTGATCAACAAAGCCCAGC
TGTCGCCTGAGTTCGTCAAAATCAACCCTCAGCACACTGTTCCAACTCTTGACGATGATGGCTTCATTTTGTGGGAATCAAGGGCTATCGCTGCTTACTTGGTCAGCTCCAA
AGCTCCGGGGAGTCCTCTGTATCCTACTGATCCTAAGATCAGGGCCGTTGTGGACTCTCGCTTGTACTTTGATGGATCAAACTTGTACCCTAAAGCCAGGGACATTATTGTA
AGATTGAGCTTCTTCTGTTGATCTAAAAATTAATTTTTCTTAAAATTTTAATATCTGATTTTTCCTTTAGTTCCCAATTCTATTTCTGGGCGTTAAAGAAGTAGACGAAG
GGAAGAAGCAAATCCTCTATCAAGCTCTGGAGTTCATGAATACCTTCTTGGAAGGAAAAGACTGGTTTGCAGCTGATCATCCCACAATCGCTGATTTGGCTCTCTTGGCTTC
ATTTGCTTCAATTTTCGTAAGTTTTTCCTCGAGTTTTCTTTGCCAAATTATTAGATTTTTAATAAAATTTAATTTTCAGCACGCTGGAGCAAATGTGAGCAAGTACACGA
ACATATTGGCCTGGTATAAACGCTGCGAATCTTTGCCAGGATTTGAGGAGAATGAGGCTGGAGCTAAAACTTTCGGGCAAGCAGTGAAAGGAAAGCTCGGCATGACTGGAAA
TTGGGACTGAAAGTATTATTCAGAATATCATCATACTTAAATTTATAACATTTACTAATGATTTAATAAAATTTGAGCTTCATTGAATTCAAAATTAGTT

>LLOJ010538-RA (222 aa; 4 exons) | UNCHANGED
MAPLKLYHFPISAPSRGALLAIRNLKLDVEIVEINLMNKEHLSPEYVKINPQHTVPTLDDNGFILWESRAIATYLANSKAPGNSLYPTDPKIRAVVDSRLYFDASNLF
PKARNIVFPILILGVKEVDPEKKQILYQALEFMNTYLEGQNWIAADHPTLADLTLLSSISSIYHAGANISKFPNIMAWYKRCESLPGFEENETGAKAFGQAIKKNLGI
TGTWDD-

>JH690204 reverse | ***LLOJ010538-RB*** (pre-editing) | LLOJ010538-RB (post-editing)
ACTTCAGTGTTCCTTCTTTCCAGTTCAATTCGGTGAATTTCAATCAAAAGTGAGTCTTATGATCATTCTTGGCTGACACAGAGAATATTCTTTAAGCTCCAAGGCCAT
TTTTTCTTCTCAAATCTGGCATCAAGGCAATTTTTGTGATGATTTAATGAATCTTTTTTGTGTGATTTCTCCTCCAAAAAAGCAGCCAAACAATGGCCCCACTGAAGTT
GTACCATTTTCCAATCAGTGCTCCCTCCCGTGGAGCTCTTCTCGCCATCCGGAATCTGAAGCTTGATGTTGAGGTATGTTTAAATAATGCTCCATGGTGCATAAAGTAAAA
TTTGTAACTTTTATTGTGATTTAAATGAAAAGAACTTGTTGAGAAATTACGCAGCTGAAAGTTGAATAGAATTTCTCTGAATGGAGGTGAAAGTGATAATAGTTTTGC
GTAGGTTGGGCAGAGAGTAATTCATTTAAAAGGGGACAGAAAAATGAATAATTGTTCATTAACTCTACTCTCGTATTCTTTCTCCCTTAAGGGCATTGCCATTTAACT
CGATGGCATGACATGATAAGTAAAATGGAAGTTATCTTGAAATTTTATTTTATCTTCATCAAATAAGAGACAAGTTGAGGGTGAAGTGTCGAAATAAAATTAAACAAC
TCACCTTAGTAAATATATTTAAACTTGAAAACCCTAATGAACCATGCTTGCCCATCTAATACAAATTGATTTAATTTGAAATCTTAAAAATAGTTTAGATATCCATT
AAAATTTTCATTTAATTTAGTTAGAAGTGAATTGTTAAAGAGCCTTGACATTATCGAAGCTCATCTATGTTAAAATATTAAGTAGATTCACTAAACAAATGAAAAATA
ATCCATAAAGAAAACATAAAACTTCTCCCTCTCTACTCAAACAAGTCATCATAATTATTCAAATTGATCAGTTGATAAGTCCAAAAGAATGGGTATAGAAAATGTTAA
AAATAGATTTCACTCGCTTCTGTTCTTTTCCTGAAGCATTCTTTGCTTCATTTTATAACAAAGAATCTTTAGAAAAATAAATCAAGCTTAAGAATTGGCCTAAAAATT
TATACTCGTTCTTGTAGATCGTGGAAATCAATCTCATGAACAAAGAACATTTATCTCCGGAATACGTCAAAATCAACCCTCAACATACCGTTCCAACCCTTGACGATAATG
GCTTTATCTTGTGGGAATCTCGTGCTATTGCCACTTACCTTGCAAACTCCAAAGCTCCTGGAAATTCCCTCTATCCCACTGATCCCAAAATTCGTGCTGTTGTGGATTCACG
CTTGTACTTTGATGCCTCCAATTTATTCCCAAAAGCCCGGAATATTGTTGTAAGTTCTCTTCCTCTATACTCCATCTTTCTTCTCAGTTTTGTTTTACAAAATATATTAA
AGTTAGTCGTCTTTTAGTTCCCTATTCTTATCCTGGGAGTAAAGGAAGTTGATCCAGAGAAGAAGCAAATTTTGTATCAAGCTCTGGAGTTTATGAATACTTACCTAGAAG
GGCAGAACTGGATAGCAGCTGATCATCCTACTCTCGCTGATTTGACGCTCCTATCCTCAATTTCATCGATTTATGTACGTAATTGTTGGAGCAGGGTTATTTTCTTTTACA
GAGTTTGATAAATTTTTACCCAATCCTTAATTTTCCTAAATTTTTTACAGCATGCTGGAGCTAATATCAGCAAGTTTCCCAACATCATGGCCTGGTACAAACGCTGTGAG
TCCTTGCCTGGATTTGAGGAGAACGAAACAGGCGCCAAGGCTTTCGGCCAGGCAATTAAGAAGAATCTCGGGATCACAGGCACATGGGATGACTAAATCACCAGGAATTTG
TTGAACGCTGTCTTTTTATACTCTACCATATGCACTTGTACTTTTCAGAGATTATTTGCAAGAGTTGTTTAATAAATATTTTTTCTGGGATTAAAATGCTCTGGTTTT
GATTTTATTTTAATTTTTTTTTCAAACGAAATGAATGCAGAATTTAAAAAAATATTTCTTTTTAATTTTATTTAGATTGTAGAAGTTGATCTGATCAACAAAGCCCAGC
TGTCGCCTGAGTTCGTCAAAATCAACCCTCAGCACACTGTTCCAACTCTTGACGATGATGGCTTCATTTTGTGGGAATCAAGGGCTATCGCTGCTTACTTGGTCAGCTCCAA
AGCTCCGGGGAGTCCTCTGTATCCTACTGATCCTAAGATCAGGGCCGTTGTGGACTCTCGCTTGTACTTTGATGGATCAAACTTGTACCCTAAAGCCAGGGACATTATTGTA
AGATTGAGCTTCTTCTGTTGATCTAAAAATTAATTTTTCTTAAAATTTTAATATCTGATTTTTCCTTTAGTTCCCAATTCTATTTCTGGGCGTTAAAGAAGTAGACGAAG
GGAAGAAGCAAATCCTCTATCAAGCTCTGGAGTTCATGAATACCTTCTTGGAAGGAAAAGACTGGTTTGCAGCTGATCATCCCACAATCGCTGATTTGGCTCTCTTGGCTTC
ATTTGCTTCAATTTTCGTAAGTTTTTCCTCGAGTTTTCTTTGCCAAATTATTAGATTTTTAATAAAATTTAATTTTCAGCACGCTGGAGCAAATGTGAGCAAGTACACGA
ACATATTGGCCTGGTATAAACGCTGCGAATCTTTGCCAGGATTTGAGGAGAATGAGGCTGGAGCTAAAACTTTCGGGCAAGCAGTGAAAGGAAAGCTCGGCATGACTGGAAA
TTGGGACTGAAAGTATTATTCAGAATATCATCATACTTAAATTTATAACATTTACTAATGATTTAATAAAATTTGAGCTTCATTGAATTCAAAATTAGTT

>LLOJ010538-RB (221 aa; 4 exons) | UNCHANGED
MAPLKLYHFPISAPSRGALLAIRNLKLDVEIVEVDLINKAQLSPEFVKINPQHTVPTLDDDGFILWESRAIAAYLVSSKAPGSPLYPTDPKIRAVVDSRLYFDGSNLY
PKARDIIFPILFLGVKEVDEGKKQILYQALEFMNTFLEGKDWFAADHPTIADLALLASFASIFHAGANVSKYTNILAWYKRCESLPGFEENEAGAKTFGQAVKGKLGM
TGNWD-

####### LLOJ010537 - GSTX #######
>JH690204 reverse | **LLOJ010537** (pre-editing) | LLOJ010537 (post-editing)
NNTTTTTTTTTCAATTCTTTTTTTAAAACTTAAATTCTCACTTTTAAAATTTATATACATAAAATGTAATATATAGTCCTATTATAGATAATCATGTATCTCTTGGA
TATAATAAACCATTATTCATTCATTCATTCATTCATTCAAATATTTCAAGAGTTTTTTTGTACGATTTCTTTACATAAACACATCTAACATAATGGCACCATTGAAGTT

GTACCATTTCCCAATCAGTGCTCCCTCTAGAGTGGCCCTTCTGACTATTCGGAATCTAAATTTAGATGTTGAGGTAATTTTTCAATTGAAAAATTGAATTTTATTGAGATT
AATTATTGTTGATAATTGAAAAAAAAATAGAAAAGAACTAATATAAGATTTATTTACATATAAATTCATTTTCTATAATAACTAGGTATAGTTAGGAATTCAAGGGAA
TTGATAGAAATGCGAGGTTTTTATGAATTTGTGAACTTTATCAATTTTCGTGGCCTTTAATCCTCGCACTGTAAGTGTAGAAATGAATATTTCGTAACTTTGCGTTGA
TTTTATTTATAG**ATTGTTGAAATTGATCTAATCAACAAAGCACATTTGTCTCCGGAATTCGTGAAAATCAATCCTCAGCATACCGTCCCAACTTTGGACGACAATGGATTT**
ATTTTGTGGGAATCACGAGCTATTGCTCTCTATTTGATCAACTCTAGATCTCCCGGAAGTCCTCTGTATCCAACAGATCCCAAAATTCGAGCTGTTGTAGACTCTCGCTTGT
TCTTTGATGCATCAAGTTTATTTCCTAAAGCACGAAATCTCTTGGTAAGGATTTTTTTCTGGAGTTTTTACTGAATTTTCTTTATTGTTTTTTCTTTTTGTTGGTTTCAG
TTTCCAATCCTTTTCCTAGGTGTGAAGGAAGTTGATCCCGAATTGAAACGAATTCTCTATGAAGCTCTTGATTTCATGAATACCTTCTTGGAGGGACAAAATTGGATTGCAG
CTGATCATGTAACAATTGCTGATTTGACGCTCCTACCTCTGGTTTCTTGTATTATTGTACGTAAACTTTATTTAGGGAGATTTTTTAAAATTAAATTAAGGATAGTTACA
TAATATATTTTTTACAG**CATTCTGGAGCAGAAGTTTCAAAATTTTCCAACATAGAGGCTTGGTACAAGCGATGTGAGACTCTGCCAGGATTTGAGGAGAATGAATCTGGAG**
CCAAGGAATTTGGGAAATTGATTAAGGAAAAATTAGGAATTACAGGGACATGGGATTAAGCTATAATGGTAGCATTCTTCATATTTAATTTAGTAGAGAATTTTCTATAA
AAATATCTTTTTCTTGTATAAAAACTATAATTTTCTATTTTAGAACTTTCATCGGTTTCATGTGTCAATCATGACAATGCATAAAACTTAATTCCTTTTATTAAATTT
TCTTATCTAAATAATAAAGTTTTTCACTTGAAAATATTTTT

>LLOJ010537 (221 aa; 4 exons) | UNCHANGED
MAPLKLYHFPISAPSRVALLTIRNLNLDVEIVEIDLINKAHLSPEFVKINPQHTVPTLDDNGFILWESRAIALYLINSRSPGSPLYPTDPKIRAVVDSRLFFDASSLF
PKARNLLFPILFLGVKEVDPELKRILYEALDFMNTFLEGQNWIAADHVTIADLTLLPLVSCIIHSGAEVSKFSNIEAWYKRCETLPGFEENESGAKEFGKLIKEKLGI
TGTWD-

####### LLOJ010536 - GSTX #######
>JH690204 reverse | **LLOJ010536** (pre-editing) | LLOJ010536 (post-editing)
ACTGATATGCGTTATCAAAGGACTCAATAAGTGAATCACAAATCTCTGCTGTGGACTATATAATTTAATTTTCTGCAACAAATAGTTTAGTCTTTCGGTAGATGTCGA
ACAGATCAAACTATCTTGTCCTAAAAAGTTCTAGTTGGTGCATTTTAATTTATTTGTGAAGTGAATTAAATCGTGAATAACAGTCCTTAATT**ATGGCTCCTCTGAAGTT**
GTATTACATGCCAATTAGTGCCCCAGCTAGAGCCGTTCTCTTAACTATTCGAAGCTTAAAGCTAGACGTTGAGGTATATTTTATTTTAAGAAGTTCTAATTCAACAAGAAC
TCCTTTTAAAATCATTTTTGGGGATTTTCTAG**ATCATAAACATTGACATCTTAAAGGGAGAACAACTCACACCTGAATTCATTAGTATGAATCCTCAACACATGATTCCCA**
CAATTGACGACAATGGGTTTATTCTGTGGGAATCAAAGGCAATTGCTGCTTACTTGGTCAACTCCAGAGCCCCTGGAAATTCTCTCTATCCAACTGATCCTAAAATCAGGGC
TCTTGTCGATGCACGCCTCTACTTTGATACCTCCACCATATTTACAAAGTCAAAGGAAGTTTTGGTTAGTAAAATTTTAGTTAGTTAAAGCGACGTTTAACTGTTTAACT
TCTGAATTAATTGATTTTCCTTAATTTTAG**TTCCCAATCTTTGCTTTGGGAGCTAAAACGGTGGACGAGGAGAAGAAGAATGCTTTCTATGAGACACTAGATCTCATGAAT**
ACCTACCTAGAGGGAAAGAAATGGTTTGCGGGAAATAAACCAACTATAGCTGACTTTTCACTCTTGGCTTCCTTTGCGACTTTTGTTGTGAGTTTTTATTATAAAAGATTT
TTATGTTTTCTATCTTATTTCTTGATTTAAATTGCAG**CATTCGGGAGCTAATGTGTCCAAATACAAGAACATCCTAGCTTGGTATAAACAATGCGAATCCTTGCCAGGATT**
TCAGGAGAATGAAGCCGGTGCTAAGGAATTCGGGAATATGATTAAGGAGAAACTAGGCATTACCGGAACGTGGGATTAAATTTAATTAAAAGCTCTTCGTGATTGATTTTG
AAAAATGATTTAAAATGAGAAATATTTGAGAAATAAAATATTTTAACAAAGGAGTTTATTCAGGCCAGTATGAAAGATGATCCTCTATCGCAGAACAATTTTAACCCA
TTCCATCTTGTAGAGGATCAAAAAACATTGAAGAATCGCGAGAGAAACTCCCCAAAATCC

>LLOJ010536 (221 aa; 4 exons) | UNCHANGED
MAPLKLYYMPISAPARAVLLTIRSLKLDVEIINIDILKGEQLTPEFISMNPQHMIPTIDDNGFILWESKAIAAYLVNSRAPGNSLYPTDPKIRALVDARLYFDTSTIF
TKSKEVLFPIFALGAKTVDEEKKNAFYETLDLMNTYLEGKKWFAGNKPTIADFSLLASFATFVHSGANVSKYKNILAWYKQCESLPGFQENEAGAKEFGNMIKEKLGI
TGTWD-

####### LLOJ010535, *LLOJ010534* - GSTX #######
>JH690204 reverse | **LLOJ010535**, ***LLOJ010534*** (pre-editing) | LLOJ010535_a, LLOJ010534 (post-editing)
AGTTTTTATTATAAAAGATTTTTATGTTTTCTATCTTATTTCTTGATTTAAATTGCAGCATTCGGGAGCTAATGTGTCCAAATACAAGAACATCCTAGCTTGGTATAA
ACAATGCGAATCCTTGCCAGGATTTCAGGAGAATGAAGCCGGTGCTAAGGAATTCGGGAATATGATTAAGGAGAAACTAGGCATTACCGGAACGTGGGATTAAATTTA
ATTAAAAGCTCTTCGTGATTGATTTTGAAAAATGATTTAAAATGAGAAATATTTGAGAAATAAAATATTTTAACAAAGGAGTTTATTCAGGCCAGTATGAAAGATGAT
CCTCTATCGCAGAACAATTTTAACCCATTCCATCTTGTAGAGGATCAAAAAACATTGAAGAATCGCGAGAGAAACTCCCCAAAATCCACTGGGATCACATCGAAAAGT
GCAAATAAAAATTGCAAAAAAATTCAAATATTGCGACGCCATTTGACATTTTTATCCCTATAAAAAATGTTTGAAAACTTTTTTGTAGACTTGTCGCTCATTTTCCGT
GGAGTTTTCTTCAAAATAAATGTTTATTTTTTCGTTTCTGGAGTGATTTCTACATCTGAATCAGGTGATCCACAGCGAAATGAAGCTGAGGGAGGTTGATAGAACCAG
CCCCTTGCAAATCAAAAACAAATCTTTATACATTTTTTTGGGCCTCTTTGGCAGTGAGAATTTTAGCATATCTTACCGAGTTAAATCCCATCCAGCGCAATATACCTG
ATTCAGATGTAGAAATCAATATAGTAGTCAGTATCCGGAAACATACGACGGAGTCATCAAGCAAATCATGACCAGCACGGAGACATTTCTGAGAGTCACAGAACTGCC
TTCAAAGCTATTCTGGGTTTCCTCCTAAAAGTCTCCTTACGCGGATTGGACGGATTGGAACTGGAACACTGGGCAAGTCCACAAAATCTTCATCATCGTCTTTCCATA
CATCTTCCCCAAGAGCAATGACTTCCTGGAGCTGACAAAAGTGATTCTCAACTCTCATTTTGCCTCTCAGAGTTCTTTACTGCAGAATGCCCATGAGGTTTTGCTGTA
GAAATTGGGACTTCCGGAAGCAAAAGATTTGCTCCAGCAGATTGAAATTGAATCAATGGGCAGTGNNNNNNNNNNNNNNNNNNNNNNNNNNNNNNNNNNNNNNNNNNN
NNNNNNNGTGAATGGCGGTGAAAGTGATAATAGTTTTGCGTAGGTTGGGCAGAGAGTAATTTATTTAAAAGGGGACAGAAAATGAATAATTGTTCATTAAGTTTGTTC
TCGTATTCTTTCTCCCTTAAGGGCATTGCCATTTAAACTCGATGGCATGACATGATAAGTAAAATGGAAGTTATCTTGAAATTTTATCGCAATAGAAGCTTTTGAATT
TTAATTCTTCATCAAATAAGAGACAAGTTGAGGGTGAAGTGTCGAAATAAAATTAAACAACTCATCTTAGTAAATATATTTAAGCTTGAAAACCCTAAAGAACCTTGC
TTGTCCATCTAATACAAATTGGTTTAATTAGAAATCTTAAAAATGATTTTAGATAATCAATGAGAATTTTCATTTAATTTAGTTAGAACAGTTAGAAGTGAATTGTTA
AAGAGCCTTGACATTATCGAAGCTCATCTATGTTAAAATATTAGATTCACTAAACAAATGAAAAATAATCCATAAAGAAAACATAAAACTTCTCCCTCTCTACTCAAA
CAAGTCATCATAATTATTCAAATTGATCAGTTGATAAGTCCAGAAGAATGGGTATAGAAAATGTTAAAAATAGATTTCTACTCGCTTCTGTTCTTTTACATTTTTCCT
GAAGTATTCCTGCTTCATTTTTTAACAAAGAATCTTTAAAAAATAAATCAAGCTTAAGAATTGGCCTAAAATTTATACTCGTTCTTGTAGATCGTGGAAATCAATCTC
ATGAACAAAGAACATTTATCTCCGGAATACGTCAAAATCAACCCTCAACATACCGTTCCAACCCTTGACGATAATGGCTTTATCCTGTGGGAATCTCGTGCTATTGCCACTT
ACCTTGCAAACTCCAAAGCTCCTGGAAATTCCCTCTATCCCACTGATCCCAAAATTCGTGCTCTTGTGGATTCACGCTTGTACTTTGATGCCTCCAATTTATTCCCAAAAGC
CCGGAATATTGTTGTAAGTTCTCTTCCTCTATACTCCATCTTTCTTCTCAGTTTTGTTTTACAAAATATATTAAAGTTAGTTGTCTTTTAG**TTCCCTATTCTTATCCTG**
GGAGTGAAGGAAGTTGATCTGGAGAAGAAGCAAATTTTGTATCAAGCTCTGGAGTTTATGAATACTTACCTAGAAGGGCAGAACTGGATAGCAGCTGATCATCCTACTCTCG
CTGATTTGACGCTCCTGTCTTCTATTTCTTCGATTTATGTACGTAAATGTTGGAGGAGGATTATTTTCTTTTACAGAGTTTGATAAATTTTTACCCAATCCTTAATTTT

CCTTAATTTTTTACAGCATGCTGGAGCTAATATTAGCAAGTTTCCCAACATCATGGCCTGGTACAAACGCTGTGAGTCCTTACCAGGGTTTGAGGAGAATGAAACAGGGGC
CAAGGCTTTCGGTCAGGCAATTAAGAAGAATCTCGGGATCACAGGCACATGGGATGACTAAATCACAAGGAATTTGTTGAATTCTGTCTTTTTATACTTTACCATATGCA
CTTGTATTTTTCTGAGATTATTTGCAAGAGTTGTTTAATAAATATTTTTTTCTGGGATTACAATGCTCTGGTTTTGATTTTATTTAATTTTTTTTTGCTCAAACGAAA
TGAATGTAGAATTAAGAAAATATTTCTTTTTAATTTTATTTAGATTGTAGAAGTTGATCTGATCAACAAAGCCCAGCTATCGCCTGAGTTCGTCAAAATTAACCCTCAGC
ACACTGTTCCAACTCTTGACGATGATGGCTTCATTCTGTGGGAATCAAGGGCTATCGCTGCTTACTTGGTCAGTTCCAAAGCTCCAGGGAGTCCTTTGTATCCTACTGATCC
TAAGATCAGGGCCGTTGTGGACTCTCGCTTGTACTTTGATGGATCAAACCTGTACCCTAAAGCCAGAGACATTATTGTAAGATTGAACTTCTTCTGATTGATCTAAAACAT
AATATTTCTTTAAGTTTTAATTTCTGATTGTTCCTTCAGTTCCCAATTCTATTTTTGGGCGTTAAAGAAGTAGACGAAGGGAAGAAGCAAATCCTCTATCAAGCTCTGGAG
TTCATGGATACCTTCTTGGAAGGAAAGGACTGGTTTGCAGCTGATCATCCCACAATCGCTGATTTGTCTCTCTTGGCTTCATTTGCTTCAATTTTCGTAAGTTTTTCCTAG
AGTTCTCTTAGCTAGATTATTAGATTAAAAAAAGTTTAATTTTCAGCACGCTGGAGCGAATGTGAGCAAGTACACGAACATATTGGCCTGGTACAAACGCTGCGAATCCT
TGCCAGGATTTGAGGAGAATGAGGCTGGAGCTAAAACTTTCGGGCAAGCAGTAAAAGGAAAGCTCGGCATGACTGGAAATTGGGACTGAAAGTATTATTCAGAACATCATC
ATACTTAAATTTATAACATTTACTAATGACTTAATAAAATTTGAGCTTCATTGGATTCAAAATTATTTTTTGAGCATTCGCTGACAACTAGTTGGTGCGAAAAAGATC
ACGATAGGACTGATAAAATAAATTGGGGGAGTTTAATTATACGAGCAAACCCACTATCAATTCCACAACT

>LLOJ010535_a (>192 aa; >3 exons) | EDITED (PARTIAL - N-TERMINAL MISSING)
IVEINLMNKEHLSPEYVKINPQHTVPTLDDNGFILWESRAIATYLANSKAPGNSLYPTDPKIRALVDSRLYFDASNLFPKARNIVFPILILGVKEVDLEKKQILYQAL
EFMNTYLEGQNWIAADHPTLADLTLLSSISSIYHAGANISKFPNIMAWYKRCESLPGFEENETGAKAFGQAIKKNLGITGTWDD-

>LLOJ010534 (>191 aa; >3 exons) | EDITED (PARTIAL - N-TERMINAL MISSING)
IVEVDLINKAQLSPEFVKINPQHTVPTLDDDGFILWESRAIAAYLVSSKAPGSPLYPTDPKIRAVVDSRLYFDGSNLYPKARDIIFPILFLGVKEVDEGKKQILYQAL
EFMDTFLEGKDWFAADHPTIADLSLLASFASIFHAGANVSKYTNILAWYKRCESLPGFEENEAGAKTFGQAVKGKLGMTGNWD-

[No exon 1 could be found upstream of either gene. Exon 1 of LLOJ010535_a could be on the sequencing gap.
Both genes could be isoforms, with missing exon 1.]

####### LLOJ010533 - GSTX #######

>JH690204 reverse | **LLOJ010533** (pre-editing) | LLOJ010533_a (post-editing)
CTATCAATTCCACAACTGATCATTTAGTCTTTGTTCACCCATTCAACTGACTAGTGCTTCTGTCTTTGACTCAATTTGGTGTGAAGAAGCAAAGTTTTTAAATCAAAA
GTGAGTTTAATAAATATCTCATAAAAGAAATTTTTTTTAAATGATTTTAAGAGTTTTTTGTGCGATTTTTTTACATAAATACAGCTAACATAATGTCACCATTGAAGTT
GTACCATTTTCCAATCAGTGCTCCCTCTAGAGTGGCCCTTCTGACTATTCGGAATCTAAATTTAGATGTTGAGGTAATTTTTCAATTGAAAAATTGAATTTTATTGTGA
TTTATTGTTGTTGACAATTAAATAAAACAGAAGAATTAAGATTTATTTAAATATAAATACATTTTTTCTATAGTAACTAGGAGTAGATAGGTGTACTAGGGAATTTAT
AGAAATGCGAGATTTTTATGAATTTGTAAACTTTATCAATTTTCGTGGCCTTTAATAAGAATTAATCCTCGGTCTGTAAGTGTAGAAATGAATATTTCGTAACTTTAC
GTTGATTTATTTATAGATCGTTGAAATCGATCTAATCAACAAAGCACATTTGTCTCCGGAATTCGTGAAAATCAATCCTCAGCACACCGTCCCAACTTTGGACGACAATGG
ATTTATTTTGTGGGAATCACGAGCTATTGCTCTCTATTTGGTCAACTCTAGATCTCCCGGAAGTTCTCTGTATCCAACAGATCCCAAACTTCGAGCTGTTGTAGACTCTCGC
TTGTTCTTTGATGCATCAAGTTTATTTCCAAAAGCAAGAAATCTCTTGGTAAGGAATATTCCTCTGGAGTTTTTACTGAATTTTCTTCATTTTTTTCGTTTTCTTTTTTC
AGTTTCCAATCCTTTTCCTAGGTGTAAAGGAAGTTGATCCCGAATTGAAACGAATTCTCTATGAGGCTCTTGATTTCATGAATACCTTTTTGGAGGGACAAAATTGGATTGC
AGCTGATCATGTAACAATTGCTGATCTGACGCTCCTACCTCTCGTTTCTTGTATTATTGTAGGTAAACTTTATTTACGGAGATGTTTTTTTTTTTTTAATAAATTAAGGA
TAGTTACATAATATACATTTTACAGCATTCTGGAGCAGCAATTTCAAAATTTCCCAACATAGAGGCTTGGCACAAGCGATGTGAGACTCTGCCAGGATTTGAAGAGAATGA
ATCTGGAGCCAAGGAATTTGGGAAATTGATTAAGGAAAAATTAGGAAATACAGGGACATGGGATTAAGCTATAATGGTAGCATTCTTTATGTTTCATTTAGTAGAGAATT
TTCTATAAAAATATCTTTTTCTTGTATAAAAACTATAATTTTCTATTTTAGAACTTTCATCGGTTTCATGTGTCAATCATGACAATGCATAAAACTTAATTTCTTTTA
TTAAATTTTCTTATCTAAATAATAAAGTTTTTCACTTGAAAATATTTTT

>LLOJ010533_a (221 aa; 4 exons) | EDITED
MSPLKLYHFPISAPSRVALLTIRNLNLDVEIVEIDLINKAHLSPEFVKINPQHTVPTLDDNGFILWESRAIALYLVNSRSPGSSLYPTDPKLRAVVDSRLFFDASSLF
PKARNLLFPILFLGVKEVDPELKRILYEALDFMNTFLEGQNWIAADHVTIADLTLLPLVSCIIHSGAAISKFPNIEAWHKRCETLPGFEENESGAKEFGKLIKEKLGN
TGTWD-

####### LLOJ010532 - GSTX #######

>JH690204 reverse | **LLOJ010532** (pre-editing) | LLOJ010532 (post-editing)
GACTATATAATTTAATTTTCTGCAACAAATAGTTCAGTCTTTCAGTAGATCTGGAGTAGATCAAACTATCTTCTCCTTAAAAGTTCTAGCTGGTGTATTTTAACTAAT
TTTTTTGTGAAGTGAAATAAATTGTGACTATTATTATCCAAGGATTTTAAGAACAATTTTTCAGGAAAATTATCCCCAAACAGTCCTAAATAATGGCTCCTCTAAAGTT
GTATTACATGCCAATTAGTGCTCCAGCCAGAGCAGTTCTCTTAACCATTCGGAGCTTAAAACTAGACGTTGAGGTATATTTTCTTTCTAGAAGTTATAATTCAACAAGAAC
TCTTTTTAAAATCATTTTTGGGGATTTTCTAGATCATTAACATTGACATCTTAAAGGGAGAACAACTCACACCTGAATTCATTAGCATGAATCCCCAGCACATGATCCCCA
CAATTGACGACAATGGGTTTATTCTGTGGGAATCAAAGGCAATTGCTGCTTACTTGGTCAACTCCAGAGCCCCTGGAAATTCTCTCTATCCAACTGATCCTAAAATCCGAGC
CCTTGTTGATGCACGCCTCTACTTTGATACATCCACCATATTTACAAAGTCAAAGGAAGTTTTGGTTAGTTGAATTTTAGTTAGTTAAAGCAACGTTTAACCGTTTAACT
TTTCAATTAATTGATTTTCCTTAATTTTAGTTCCCAATTTTCGCTTTGGGAGCCAAAACGGTGGACGAGGAGAAAAAGAATGCTTTCTATGAGACACTAGATCTCATGAAT
ACCTACCTAGAGGGAAAGAAATGGTTCGCAGGCAATAAACCAACTATAGCTGACTTTGCACTCTTGGCTTCCTTTGCAACTTTTGTTGTAAGTTTCTTTATTAAAGATTTT
TATTATTTCTGTCTAATTCCTTGAGTTAATTTGCAGCATTCGGGAGCTAATGTGTCCAAGTACAAGAACATCCTAACTTGGTATAAACAATGCGAATCCTTGCCAGGATTT
CAGGAGAATGAAGCGGGGGCTAAGGAATTCGGGAATATGATTAAGGAGAAATTAGGCATAACCGGAACTTGGGATTAAATTTAATTAAAAGCTCTTCGTGATTGATTTTTG
GAAAATGATTTAAAATGAGAATTAATTGAGAAATAAAATATTTTAGCAAATTTGAATTAATTGGTCTCTTTTTCTTAATTTTAATTTTAATTAAGATAATAAAGAGCA
AATAAAGATATTAAATAATTTAATCGTTGTATGTTCAGCAATCAGTACTGCCTTTCATG

>LLOJ010532 (221 aa; 4 exons) | UNCHANGED

MAPLKLYYMPISAPARAVLLTIRSLKLDVEIINIDILKGEQLTPEFISMNPQHMIPTIDDNGFILWESKAIAAYLVNSRAPGNSLYPTDPKIRALVDARLYFDTSTIF
TKSKEVLFPIFALGAKTVDEEKKNAFYETLDLMNTYLEGKKWFAGNKPTIADFALLASFATFVHSGANVSKYKNILTWYKQCESLPGFQENEAGAKEFGNMIKEKLGI
TGTWD-

####### LLOJ010530 - GSTX #######

>JH690204 reverse | **LLOJ010530** (pre-editing) | LLOJ010530 (post-editing)
AAATTCGTTTGAAATTATTTTGATACAAAAAAGGTTTTTTTTGAGTCATATTGGTTATTTTGTCAAGCCAAGAGTACAAGGGCTAAAAGAGAAAGTTTCAATGATTGG
CCCTAAGGCCAAAATGTTCAATACAAGGTTCCCTCTCTAAAATTTTATAGTGATTTATTTTATACTCTTCTATCACTAATTTAATCAGCAAC**ATGGCCCCAGTGAAGTT**
CTACCATTTACCATTAGGCCCCCCATCTCGTGGGGTTCTTTTGACCATTCGGAATTTGAATCTCGATGTTGAGGTAAGATTTTTGTGAATTTTATTTAATAAAAAATATCA
CTAGAAATTAATTTTTGAAAATCATTTTCAG**ATTATTGAGGTTGACATCTTCAAAGGGGAGCATTTAACCCCTGAATACTTGGAGATGAATCCTCAACACACCATTCCAGC**
TCTCAACGATAATGGCCTTTATTTGGGGGAGTCCAAAGCCATTTCCACCTATTTGGTTAATTCAAAAGCCCCCGGACATCCGCTCTATCCAACTGATCCAGCTATACGAGCT
CAGGTGGATGCAAAACTCTACTTTGATGCTGCCACAATATTTCCACGAATGAGAGCAATTTTTGTGAGTTCCTCAAAAGGAAATTTAAAAGATTTTCTTTGTTAATTTCT
CATTTTATTTGTTTTCAG**TTCCCTATTCTTTTCCTTGGTTCGAAAACCATTGAAAAGGAGAAGAAGGAAGCTTTCTATCAGGCTCTTGATTTTATGAATACCTTTTTGGAG**
GGCCGCACGTGGTTTGCTGCAGATCATCCCACACTCGCTGATTTGGCTCTCTTGGCTTCTTTTTCAACATTTGTCGTGAGTTTTATGAATTTTAACTTGATGAATTTATTA
AAGAATTGTTTAAAACTTATTTAAAAATTTTCAG**TATTGTGGAGCAGATGTTTCCAAGTACACCAACAGCCTAGCTTGGTACAAACGATGCGAATCCTTGCCAGGATTTGA**
GGAGAATGAAGAGATGGCTAAAAAACTAGGAGGGCTCGTGAAGGAGAAGCTCGGCATTACTGGTTATTGGGAATAATTTTAAGTTTCTTTTACAAATTCATTCAATATTTG
AGATAAATTCATTATCATTCCAAACATCACAATAAAAAGTTTTTTGAGAAAACAGACTGGGTTTTGTCTTGAATATTCTTTTAGCTGTGTTTCTTTCAGACACAGCTT
TTGTGTACCGAGCAAAATCGAAAGTCGTCAGATCGGGCTCAAACTTGGGATGAGCAC

>LLOJ010530 (221 aa; 4 exons) | UNCHANGED
MAPVKFYHLPLGPPSRGVLLTIRNLNLDVEIIEVDIFKGEHLTPEYLEMNPQHTIPALNDNGLYLGESKAISTYLVNSKAPGHPLYPTDPAIRAQVDAKLYFDAATIF
PRMRAIFFPILFLGSKTIEKEKKEAFYQALDFMNTFLEGRTWFAADHPTLADLALLASFSTFVYCGADVSKYTNSLAWYKRCESLPGFEENEEMAKKLGGLVKEKLGI
TGYWE-

####### LLOJ010531 - GSTX #######

>JH690204 reverse | **LLOJ010531** (pre-editing) | LLOJ010531_a, LLOJ010531_b (post-editing)
CTTTTCTACTTTTCCCGACTTTGCGCGTATTTAAATTAATTCGCAATCTAGTTTGCTCTCACAAAATTGGTACACTGAAAGAAACACAGCTCTCGTAAGCTTGGTCAG
CTTACCAGTACATTTTTCTTGGCACTGTATATAAAAATCCTGTTTCATTAAACTTTTTCTCTTACGCTATACCTACCTTCCAAACAGACGCA**ATGGCCCCTTTGAAGTT**
CTACCATTTTACTCTTGGTTCTCCCTCTCGTGGTGTCCTTTTGACCATCCGGAATTTAAATCTCGATGTTGAGGTGAGAATTTATTAATCTTTAAAATCTTTATTACTATT
TAAATATTTTATTAAATCTTTAACACCCAG**ATAATCGAAGTGGATACCGTGAAGGGCGAGCAAATGAATCCTGATTTTGTGAAGATTAACCCCCAGCATATTCTCCCCACA**
ATCGATGACAATGGCTTTGTTTTGTGGGAATCCAAAGCCATTTCCACGTATTTGGCCAACTCAAAAGCCCCCGGAAATCCTCTCTATCCAACTGATCCTAAAATCCGAGCTC
TTGTGGATTCTCGCATGCAATTTGATTCGGATAGTTTATATCCCAAAATCAGAGATAATTTTGTAAGACAATTTTGAGATTTTTTTTGAATTAGAGAATTAATATATGT
TGTTTTTTGATCGGAGACAGTCAATAAAAACGCACGTCTTCACGCTTTCAGCGTACGTTTCGGTTTATTTTAAACCATCATCAGGCTCTACAACAAAATATGTACATC
TTAGTACCAACATAGTGACATAAATCTGGGAAAAACATTCAAAAAGCGCATGAAAATACAAACTCACTTCAGCAATACGACACCGTTATACTATAAAACGAAGAATTG
CACATCAAAATTGGTCAAACTACCAGCACGCCATAACACAATACACACAAACCGCCCGAGCCCTCACAGATGATCCAGAATGCTTTGCGTCGCTTCCCAGACTAATGG
GCTGTAACACTCGTGCAAGCCAGCCCCCTCCATTCGACAATTGACCGCATTGGGTAGACGAAGATGAATATACCAACATTCCATCACATTAAGGTGGTGGGAGAAGCG
AGCTTGATCCAAACCCGCAGAGATTCGAAGTCAAATTGGTGGCCTCTGTTCTCCTGATCCCTCACATGATTCACCAATGATGTGGCTTCGGTGTTGGAGTCATTCAAT
GTTCTTTTATGCTGTCTTATGCGCAACATAATCTTTTGAGACGTTCTACCAATGTATTCGCGTCCGCAACTACACGGAATTGAATAGATCACGTTAGATTTCTCTTCA
AGAGGAGTTTTTGTCTTTGAGTGGTGTAAATAGAGCGGCCACCGTGTTGTGTGACTTGAAGCTGATTGTGATGCTATTTGTGAATTGGCTGATAGTCTTTTTAATAGA
TTCACTCAATCCAGGAACGTAAGAGAGAGAAAGATAGCTACGCTCCACAATTTCTGGTCTCCTATTCGAATTCACAGGTCCATTGAGTAATCTCCCAATCAATTGCTT
GGGATAACCGTTAGTTCTGAGAATGTCGGTGATCACTGTGTTGGTGTCGTTCTTCGTGTCCGTGGTCAAAGTTTTTACACGATGGATTAATCCACGCGCTGTGTTCAT
TTTTTTGTGAAAGGGGATGTGNNNNNNNNNNNNNNNNNNNNNNNNNNNNNNNNNNNNNNNNNNNNNNNNNNNNNNNNNNNNNNNNNNNNNNNNNNNNNNNNNNNNN
NNNNNNNNNNNNNNNNNNNNNNNNNNNNNNNNNNNNNNNNNNNNNNNNNNNNNNNNNNNNNNNNNNNNNNNNNNNNNNNNNNNNNNNNNNNNNNNNNNNNNNNNNNNNN
NNNNNNNNNNNNNNNNNNNNNNNNNNNNNNNNNNNNNNNNNNNNNNNNNNNNNNNNNNNNNNNNNNNNNNNNNNNNNNNNNNNNNNNNNNNNNNNNNNNNNNNNNNNNN
NNNNNNNNNNNNNNNNNNNNNNNNNNNNNNNNNNNNNNNNNNNNNNNNNNNNNNNNNNNNNNNNNNNNNNNNNNNNNNNNNNNNNNNNNNNNNNNNNNNNNNNNNNNNN
NNNNNNNNNNNNNNNNNNNNNNNNNNNNNNNNNNNNNNNNNNNNNNNNNNNNNNNNNNNNNNNNNNNNNNNNNNNNNNNNNNNNNNNNNNNNNNNNNNNNNNNNNNNNN
NNNNNNNNNNNNNNNNNNNNNNNNNNNNNNNNNNNNNNNNNNNNNNNNNNNNNNNNNNNNNNNNNNNNNNNNNNNNNNNNNNNNNNNNNNNNNNNNNNNNNNNNNNNNN
NNNNNNNNNNNNNNNNNNNNNNNNNNNNNNNNNNNNNNNNNNNNNNNNNNNNNNNNNNNNNNNNNNNNNNNNNNNNNNNNNNNNNNNNNNNNNNNNNNNNNNNNNNNNN
NNNNNNNNNNNNNNNNNNNNNNNNNNNNNNNNNNNNNNNNNNNNNNNNNNNNNNNNNNNNNNNNNNNNNNNNNNNNNNNNNNNNNNNNNNNNNNNNNNNNNNNNNNNNN
NNNNNNNNNNNNNNNNNNNNNNNNNNNNNNNNNNNNNNNNNNNNNNNNNNNNNNNNNNNNNNNNNNNNNNNNNNNNNNNNNNNNNNNNNNNNNNNNNNNNNNNNNNNNN
NNNNNNNNNNNNNNNNNNNNNNNNNNNNNNNNNNNNNNNNNNNNNNNNNNNNNNNNNNNNNNNNNNNNNNNNNNNNNNNNNNNNNNNNNNNNNNNNNNNNNNNNNNNNN
NNNNNNNNNNNNNNNNNNNNNNNNNNNNNNNNNNNNNNNNNNNNNNNNNNNNNNNNNNNNNNNNNNNNNNNNNNNNNNNNNNNNNNNNNNNNNNNNNNNNNNNNNNNNN
NNNNNNNNNNNNNNNNNNNNNNNNNNNNNNNNNNNNNNNNNNNNNNNNNNNNNNNNNNNNNNNNNNNNNNNNNNNNNNNNNNNNNNNNNNNNNNNNNNNNNNNNNNNNN
NNNNNNNNNNNNNNNNNNNNNNNNNNNNNNNNNNNNNNNNNNNNNNNNNNNNNNNNNNNNNNNNNNNNNNNNNNNNNNNNNNNNNNNNNNNNNNNNNNNNNNNNNNNNN
NNNNNNNNNNNNNNNNNNNNNNNNNNNNNNNNNNNNNNNNNNNNNNNNNNNNNNNNNNNNNNNNNNNNNNNNNNNNNNNNNNNNNNNNNNNNNNNNNNNNNNNNNNNNN
NNNNNNNNNNNNNNNNNNNNNNNNNNNNNNNNNNNNNNNNNNNNNNNNNNNNNNNNNNNNNNNNNNNNNNNNNNNNNNNNNNNNNNNNNNNNNNNNNNNNNNNNNNNNN
NNNNNNNNNNNNNNNNNNNNNNNNNNNNNNNNNNNNNNNNNNNNNNNNNNNNNNNNNNNNNNNNNNNNNNNNNNNNNNNNNNNNNNNNNNNNNNNNNNNNNNNNNNNNN
NNNNNNNNNNNNNNNNNNNNNNNNNNNNNNNNNNNNNNNNNNNNNNNNNNNNNNNNNNNNNNNNNNNNNNNNNNNNNNNNNNNNNNNNNNNNNNNNNNNNNNNNNNNNN
NNNNNNNNNNNNNNNNNNNNNNNNNNNNNNNNNNNNNNNNNNNNNNNNNNNNNNNNNNNNNNNNNNNNNNNNNNNNNNNNNNNNNNNNNNNNNNNNNNNNNNNNNNNNN
NNNNNNNNNNNNNNNNNNNNNNNNNNNNNNNNNNNNNNNNNNNNNNNNNNNNNNNNNNNNNNNNNNNNNNNNNNNNNNNNNNNNNNNNNNNNNNNNNNNNNNNNNNNNN
NNNNNNNNNNNNNNNNNNNNNNNNNNNNNNNNNNNNNNNNNNNNNNNNNNNNNNNNNNNNNNNNNNNNNNNNNNNNNNNNNNNNNNNNNNNNNNNNNNNNNNNNNNNNN
NNNNNNNNNNNNNNNNNNNNNNNNNNNNNNNNNNNNNNNNNNNNNNNNNNNNNNNNNNNNNNNNNNNNNNNNNNNNNNNNNNNNNNNNNNNNNNNNNNNNNNNNNNNNN
NNNNNNNNNNNNNNNNNNNNNNNNNNNNNNNNNNNNNNNNNNNNNNNNNNNNNNNNNNNNNNNNNNNNNNNNNNNNNNNNNNNNNNNNNNNNNNNNNNNNNNNNNNNNN
NNNNNNNNNNNNNNNNNNNNNNNNNNNNNNNNNNNNNNNNNNNNNNNNNNNNNNNNNNNNNNNNNNNNNNNNNNNNNNNNNNNNNNNNNNNNNNNNNNNNNNNNNNNNN

NNNNNNNNNNNNNNNNNNNNNNNNNNNNNNNNNNNNNNNNNNNNNNNNNNNNNNNNNNNNNNNNNNNNNNNNNNNNNNNNNNNNNNNNNNNNNNNNNNNNNNNNNNNN
NNNNNNNNNNNNNNNNNNNNNNNNNNNNNNNNNNNNNNNNNNNNNNNNNNNNNNNNNNNNNNNNNNNNNNNNNNNNNNNNNNNNNNNNNNNNNNNNNNNNNNNNNNNN
NNNNNNNNNNNNNNNNNNNNNNNNNNNNNNNNNNNNNNNNNNNNNNNNNNNNNNNNNNNNNNNNNNNNNNNNNNNNNNNNNNNNNNNNNNNNNNNNNNNNNNNNNNNN
NNNNNNNNNNNNNNNNNNNNNNNNNNNNNNNNNNNNNNNNNNNNNNNNNNNNNNNNNNNNNNNNNNNNNNNNNNNNNNNNNNNNNNNNNNNNNNNNNNNNNNNNNNNN
NNNNNNNNNNNNNNNNNNNNNNNNNNNNNNNNNNNNNNNNNNNNNNNNNNNNNNNNNNNNNNNNNNNNNNNNNNNNNNNNNNNNNNNNNNNNNNNNNNNNNNNNNNNN
NNNNNNNNNNNNNNNNNNNNNNNNNNNNNNNNNNNNNNNNNNNNNNNNNNNNNNNNNNNNNNNNNNNNNNNNNNNNNNNNNNNNNNNNNNNNNNNNNNNNNNNNNNNN
NNNNNNNNNNNNNNNNNNNNNNNNNNNNNNNNNNNNNNNNNNNNNNNNNNNNNNNNNNNNNNNNNNNNNNNNNNNNNNNNNNNNNNNNNNNNNNNNNNNNNNNNNNNN
NNNNNNNNNNNNNNNNNNNNNNNNNNNNNNNNNNNNNNNNNNNNNNNNNNNNNNNNNNNNNNNNNNNNNNNNNNNNNNNNNNNNNNNNNNNNNNNNNNNNNNNNNNNN
NNNNNNNNNNNNNNNNNNNNNNNNNNNNNNNNNNNNNNNNNNNNNNNNNNNNNNNNNNNNNNNNNNNNNNNNNNNNNNNNNNNNNNNNNNNNNNNNNNNNNNNNNNNN
NNNNNNNNNNNNNNNNNNNNNNNNNNNNNNNNNNNNNNNNNNNNNNNNNNNNNNNNNNNNNNNNNNNNNNNNNNNNNNNNNNNNNNNNNNNNNNNNNNNNNNNNNNNN
NNNNNNNNNNNNNNNNNNNNNNNNNNNNNNNNNNNNNNNNNNNNNNNNNNNNNNNNNNNNNNNNNNNNNNNNNNNNNNNNNNNNNNNNNNNNNNNNNNNNNNNNNNNN
NNNNNNNNNNNNNNNNNNNNNNNNNNNNNNNNNNNNNNNNNNNNNNNNNNNNNNNNNNNNNNNNNNNNNNNNNNNNNNNNNNNNNNNNNNNNNNNNNNNNNNNNNNNN
NNNNNNNNNNNNNNNNNNNNNNNNNNNNNNNNNNNNNNNNNNNNNNNNNNNNNNNNNNNNNNNNNNNNNNNNNNNNNNNNNNNNNNNNNNNNNNNNNNNNNNNNNNNN
NNNNNNNNNNNNNNNNNNNNNNNNNNNNNNNNNNNNNNNNNNNNNNNNNNNNNNNNNNNNNNNNNNNNNNNNNNNNNNNNNNNNNNNNNNNNNNNNNNNNNNNNNNNN
NNNNNNNNNNNNNNNNNNNNNNNNNNNNNNNNNNNNNNNNNNNNNNNNNNNNNNNNNNNNNNNNNNNNNNNNNNNNNNNNNNNNNNNNNNNNNNNNNNNNNNNNNNNN
NNNNNNNNNNNNNNNNNNNNNNNNNNNNNNNNNNNNNNNNNNNNNNNNNNNNNNNNNNNNNNNNNNNNNNNNNNNNNNNNNNNNNNNNNNNNNNNNNNNNNNNNNNNN
NNNNNNNNNNNNNNNNNNNNNNNNNNNNNNNNNNNNNNNNNNNNNNNNNNNNNNNNNNNNNNNNNNNNNNNNNNNNNNNNNNNNNNNNNNNNNNNNNNNNNNNNNNNN
NNNNNNNNNNNNNNNNNNNNNNNNNNNNNNNNNNNNNNNNNNNNNNNNNNNNNNNNNNNNNNNNNNNNNNNNNNNNNNNNNNNNNNNNNNNNNNNNNNNNNNNNNNNN
NNNNNNNNNNNNNNNNNNNNNNNNNNNNNNNNNNNNNNNNNNNNNNNNNNNNNNNNNNNNNNNNNNNNNNNNNNNNNNNNNNNNNNNNNNNNNNNNNNNNNNNNNNNN
NNNNNNNNNNNNNNNNNNNNNNNNNNNNNNNNNNNNNNNNNNNNNNNNNNNNNNNNNNNNNNNNNNNNNNNNNNNNNNNNNNNNNNNNNNNNNNNNNNNNNNNNNNNN
NNNNNNNNNNNNNNNNNNNNNNNNNNNNNNNNNNNNNNNNNNNNNNNNNNNNNNNNNNNNNNNNNNNNNNNNNNNNNNNNNNNNNNNNNNNNNNNNNNNNNNNNNNNN
NNNNNNNNNNNNNNNNNNNNNNNNNNNNNNNNNNNNNNNNNNNNNNNNNNNNNNNNNNNNNNNNNNNNNNNNNNNNNNNNNNNNNNNNNNNNGAAAGGGTCATCTTCGAGAG
AGATTTTTCTACTCTAGAAAAAAATATGTTTTTTTTGGTCTACACACTAATCTACTTCCAAGACTAAAACGTTCTTATCTCAATGATATGTACCATCATTTTAGATTT
TTCTGCAAAAGAGCTTTTCCAGAGTACTTTAGCAAATGATATGTCTTATCATTGAATTGAATTTGATCTCAATTGACGTTATATGTGTGCCTCATTTTTTATTTACAT
ATATTTTGGTATATATTTTTTTTACAATCCATCGTTTAGTCTTAAGTCGAATCTCAGCGAAGACAGTGCTATTTTCGTCTGCAATTTAGTGTTAAAGAATTTAAAATT
AAAATTTAATTCTTAAAACTGCTATAAAATTTATTTTAATTGATTTTTATTAATCTGGATAAACAGACCAAATGGCCCCACTGAAATTCTACCACATGCCAAAAGGAC
CTCCTTCCCGTGCAGTTGCCTTAACGATTCGAAATCTCAAATTAGATGTTGAAGTAAGAATTAAACGTTTAACCTTAATTTAATTTAATAAATTTTTTAAAACAAATA
ATTTCTAGAAAATTGAAGTTGATGTGTTAAATGGCGAACAAATGTCTCCGGAATTTGTAAAAATTAACCCTCAACACACTGTTCCCACAATTGATGATAATGGATTCA
TTTTGTGGGAATCTCGAGCTATTATGACGTATTTGGTTAACTCCAAAGTTCCTGGAAATTCTCTCTATCCAACTGATCCCCATATCCGAGCTTTAATAGATGCTCGTT
TGTACTTTGATGCCTCAACTTTGTATGCTAGAGCCAGGGAAATCATGGTAAGACCGTTAAGCTTTTTCTAAATTAATCAATTCTTTTGGTAATTAAAAATTATTAAAT
ATAAAAATTCTTTTTATTTTGCTTTAGTTTCCCATTCT

>LLOJ010531_a (>115 aa; >2 exons) | EDITED (PARTIAL GENE - C-TERMINAL GAP)
MAPLKFYHFTLGSPSRGVLLTIRNLNLDVEIIEVDTVKGEQMNPDFVKINPQHILPTIDDNGFVLWESKAISTYLANSKAPGNPLYPTDPKIRALVDSRMQFDSDSLY
PKIRDNF

>LLOJ010531_b (>115 aa; >2 exons) | EDITED (PARTIAL GENE - C-TERMINAL MISSING)
MAPLKFYHMPKGPPSRAVALTIRNLKLDVEKIEVDVLNGEQMSPEFVKINPQHTVPTIDDNGFILWESRAIMTYLVNSKVPGNSLYPTDPHIRALIDARLYFDASTLY
ARAREIM

####### LLOJ010963 - GSTX #######

>JH689654 reverse | **LLOJ010963** (pre-editing) | LLOJ010963 (post-editing)
TGATAAATTGTATATGATTTGTTTATGCTCAGCACACAAAAACTTTTTGGCAGCTCTGTGATATCTTTGATAAGAATTCCTTCCATGAATTAAAATCAAAATTAGTCT
CTTGGGAAACTCGGTGGAAGTTGGTTGGTTTGAGAAGTTAAAGAATATTCTTTGAAAAAAGTTAAAGAATTGATTTTAAGAAGTTATAAAGA**ATGGCCCCATTGAAGTT**
GTACTACTACCCTCCTTGCCCACCTTACAGGGCAGTTCAATTTGCCATTGAATACCTCAAAATAGACGTGGAAAAGATCATTGTGGATCTATCCAAGGAAGAACAACTGAAG
CCTGAATTTCTCAAGATTAATCCTCAGCACTGCTTGCCCACAATTGACGACGATGGATACATTATGTGGGAATCTCGAGCAATTCTCGCATATCTTGTCAATTTTCGCGCCC
CTGGAAGTTCCCTCTATCCACTGGATCCAAAAAAGAGGGGCCTCATTGACTCCCGCTTAAGCCTAGACTCGAGCATAATGCATGCTCTTGGAAATGTTATCGTAAGACTTT
ATAAAACAATCAGGAAATCTTCCTCTAAAATTAATGTTAAATAACTTTTAAATTTTTTCCCCAAG**GGCCTGATCTATTCCGGCGAGACAACAATCCCACAAGAGAAGAAA**
GACAAGGTCTACACCCTTCTGGGGCACCTCAACAACTTCATAGAGGGCAAAAACTACATTGCCGGTGATGAACTAACAATTGCTGACTTTTCATTTCTTTCCACATTTGCCA
CACTGAAGGTGAGCTTCTTATCGCCCCTTATCGGGTTTGGCTTGTTAACGAACTATTTTATTTTTCTGGGCGTAG**GTTGTGGGCGCCAATGTTGCCAAGTTCCCAAACCT**
GATGGATTGGTACAAACGTTGTGAATCAGTGCCGGGATTTCAAGTCAACGAGGACGGAGCTAAAGCCCTAATTGACTACATTGCATCACGAACTTTATTCAAAGGGGAATCG
TGGGATGAATAAAGTTTAACCAATTTGTTTTAAATTAAATTAAAATTAATTAAAATCAAAATAAATTAAAATAAAATAATTTTAATTAAGATTAATTTGTTAAGAGAAC
TCACCTCTTCCGGCAAATTTTTAGATCTCCATAAATTAAGCAAATTACTTTTCAATGAATCGCCTCTCACAGAATGTTGTTTATCCAGAAGTTCTTATTCAGG

>LLOJ010963 (221 aa; 3 exons) | UNCHANGED
MAPLKLYYYPPCPPYRAVQFAIEYLKIDVEKIIVDLSKEEQLKPEFLKINPQHCLPTIDDDGYIMWESRAILAYLVNFRAPGSSLYPLDPKKRGLIDSRLSLDSSIMH
ALGNVIGLIYSGETTIPQEKKDKVYTLLGHLNNFIEGKNYIAGDELTIADFSFLSTFATLKVVGANVAKFPNLMDWYKRCESVPGFQVNEDGAKALIDYIASRTLFKG
ESWDE-

########################
####### GST zeta #######
########################

####### PPAI006902 - partial GSTZ (N-terminal; isoform A) #######
>AJVK01059004.1 reverse | **PPAI006902** (pre-editing) | PPAI006902_a-PA (post-editing)
TGCTAAAAGGGTTCAAAAAACGGCTTCAAAATTAATTACTCAAAGTACCACAACGCCATGAGTTTATCGGCGGCGATTTCAAAGGTTTTTCCACAGCAAACAGTAGAA
ATGTCACAGTGATTTTTCACACCCTCCTTTTTTCACGTATTTTAGCGAATTTGTGTGATATTTTCCACTGTAGTTATCTATTGCAAGTTGCTATGGCAAGCATCAGTT
GTAAATTGGTACAGTTTTCACTGTATTTTATTATTTTTCCTTTGTTTTCCTTCTGGCCTGTAGATTGAGGTTATGTGTGTTTTGTGGCTTTTTTTTCTTCCCGGAGCA
TAAATGTTTATTTTCGCTTTTGTTTTTATACCTCTCCCATCGCTAGGTGGCTTGTTTCCATCTGCGATGGCAGGAGAGGTGTGCTAGCTTGACTTGTGGCATGAAAAT
CTAACAGCCGGCGATAGCGCTGATGACCTTGAGTAAAATTTCCTCCTAGATGACAATAAACAAGCTTTCCAGCCGGTTTTTTGTATAATTGAGAGATTAGTATTTTTT
TATTTCCTCCGGCTTGAAATATCACCTCAATGCATGATGCCTAATCTTCAGTCTTGGCCAAGTCGTGGAAGTGTGCTATTCTGTTTGAAATATTTTTGCCTGAAGAGA
AATCCAGAAGGTTTCATAATCTGCAATC**ATGTCCAATTCGCAGCCGATACTCTACTCATACTGGCGTAGTTCGTGCTCCTGGCGTGTCCGGATAGCACTCAATCTTAAGGA**
GATCCCCTACGACATAAAGCCCATCAGCCTTATCAAGGCGGGCGGGGAGCAACACTGCAATGAGTACAGGTAAGAAGAAGCTTGTTTTACAAAAAGAATATGCTCCTATA
TGCAATCCCAGCGTCAAATAAGAAAGCCTTATACCTATAAGATTATTGTGCCAGGAAACAAAGTAACTAGAGGAAAGTTTAATTAGCTGCATGCGAGTTACAAAGTAC
TAACAAAATAAACAAATTCTCAACAAAGAATAGGGGAGCGTCAAAACCTTTGTAAATTCCACAAAAAACACACAATTATAAAGAATCACGATAAAATAGGAAGAACTA
CAGCGAATTTGAGCAAATTAGCTTCATAATTAAACGTGAAAATTGTCAATAAAATTTTTCGCCTGATTGAAAAAGAGCCGATTAAGTGAGAGTCTACTGTACAACTTT
TAAGGACCGTAAAAGTACTCGCTACGGATAAGTGGCGCTGCCCAGTGAGCACCAAATGTTAACCGATTTCAATTCAACTGAAAATATATGTATTTTCAGCTGAATTCT
GCTAATCTTAAAACAAAACTCACGAGCCAGTGCCATTTCCATACATTTGATTAGCATTTTTTGTTTAAGAACTGCTAACCAGTCAGCATATTTTTGCTGATCGATTCA
GCACAAATATGCTGAAAACGCTGCCTTTTTCAGCTGTGTGCTCGCTGGATGGACTATGCATTCAAATCAAGGGGTAACTCATATTGAGAAACCCTCGTCAATTGTCCG
AGAAACGCTTCGCGAAACCCGAGAAACCCAAAAATTGCATCGCGAAACCCGAGAAACCCAATTTCTGCATCGCGAAACCCGAGAAATCAGAAACCCTTGTCAGAAAAA
ATGGGAATGAGTTACCCCTTGGGGGCGAAATGTCATCCGGTTTATCGATAGATTTGTCAATTGGGCATTTGAGAGAGAGACTACTGTAAAGTCAATACAAGAACTAAA
CAAGGAATAGCATAACCTAGTATCTACATTAAGACAAATAGAGGGTAAAATTAACTGTTTTTATCATATAATTATACTTTTTAAGTTTTAAAGCATAACAACACTTTT
TTCATGTTAATTTTAAAACTTGTTAAGGGTAAAATTCGCATTACCAGAATGAAAAGTTTAGACATGGTTTTTGGTCTGGTTTAGACCGTTTAGGGTGCATTTGACCGT
CATTGTACAATTTTATTTAGATTTTTATGTAAAGGTAAGGATATACCCTATTTATCCAATTAATAATCTCATTACCTGATTAACGAAAAAGCGTTCTCGAAATGTCGT
TGAGTTTGGAAAGGAATAATCTAAACTAAACCCGTAAATCCCGAAGCCAAAATCCAGAAAAGTCAAAATCCCGAAGCTAAGGTCCCGAATGATCAAAATCCTGAAAGG
GACGAAATAATAAGCAGGATAATGTGTGAAATAATTTCCCAAAACAGAAAATTTCTCTTTGTCTCCAAAAAGTGCGGGTGCACACGTTAGAGTGGCTGTGAGACTTTT
TAAAAATTGAAGCTTTTGGGTTCAAGAAAATCTTCCAGCATCGACTACGAGACCAAGAGATAGACCACCTAAAAGGTGGTATGAGTCCTGGATATCAGAATCACAACT
CCTGCTGATGCTAAGTACCATGCCCCCTTCAAATACTCAAACGACGGCAAACAATCCGTAGCCTAACTGTGGAAAAAACAGCTATTTGTCTAATTAAAGTAGAAGAAG
AAAAGAAGATTTTAGCTTTCGGGATTTTGACCGAGACTCAATCTAAACCAGATCGAAGACTATGTTCTAAATTTCGTTAACTTTAAACTGACTAACCTGTTCCTATT
AACCCTATAAGGATCAGTAGTATACCGGTGTCCCTAAAACAATTTTTTTTTGCTTTAGGAAGTCATGTCTTCTAAATAGGTTTGTTTTTGCTTGTTCTCCACTGTGTT
TGTTTGTGCTCCACTTGTCCTTAAAAGTTTATTAGAATTATTTTCTACTTACTCGAATTCGAATAATCAATGAAACTCCAGATACGTCTAATTTTGACTAAAAAAATA
ATTGGTAATGGTCCCAGGATTTAAAAAAATCTTGTTCTCTTTCTACGGTAAGGTCAAGAAGGTTACAAATTTTATTTTCAAAAGCACACTGTGAATGATGAGACTTAG
CTGGGTAGAACGTTATTGAAAATTGTAGAAACCGGTTCTTCCATAACTTTTGAAACGGTCATATTTTGAAATTTACGAGTACTTTTCTGTAGATAGAACAGCACCGAA
GGGAACGTTTTCGCGTGGAGGGGAAGTCAGTCTTGGTTAGAGCGAGGAAGAGGATGCATTGCGCTTTGTATCCTGGGTCTGATAAGAATGATAAATTGCACATCCCAA
GGCCCAGGGCAACAAAATGTCATATCTTAGTTCCCAGGGTCGAGTTATAGAGCAGTCCGAAAGCATTCCAAATCAGGATCTTTAGCTATTTAGGTCTTATACACAAAA
ATGCACTTATAGACTTTTTGCGATCTTTCCGTGGTGTGCTTTCACCAAGATTATCAATGAATTATTTAAGTCCGAATAGGGAATTTACGAAGTTTCATTATGAAATTC
GATATCAAGTTGTTATTTTTCCTTGTTAGCATTAATAAAGGACGTGATCCAAAGTATATACTCGCCCTTTATAAACAATAATGTTACCGTAAGTGCGCGTGACTTTGA
CACCCCCCTGTTCGTATGCTTCTCTTTACTTCTAGAACTTAAGGATGGTCTTTACCGAATCGATCTGCCATGTTTGATCAGTAAAAACAATCCTTAGGTTCTAGAAAT
AAACATAAAAGATTACGAACAGGGGGCTGCCAAAGTCACCCTCAAGGGACAAAGTTCCTCATGAGAAATACTAATATCGTAAAATTTATATACATCACTGATTAATAG
TAGCACATTGTTCCTGTACTTATACAGCGCGTGTGATGAGTCTTATAGATCTAATCACATTAGTGTTGAAAAGAAGCATATTGTGCTTATTTTAACCCACGAACATGC
TTGATATTAAGAGATTAATATAAATCTGTGCTAAACAGTAGTGCATTATCTTGGAATATTGTATGTGAACTGATTATGTATAATCTTTAGTTACTACGGTGTTCGTTA
GAGGATTGCTTTTTTCTTAAATAAAAAAGACTCAATTTTTTCTAGAGCTTTCAACCTTCGGTATTTGTATTTATCAGTAGGTTGGTTATAATCTTCTAAATGATTTGG
AATCGTCTCGTCAGTAGTATTGTGCGAAATCTGATTCACAGAGGAAAATTCATACTGGTGATCGAAAGCTCTGGAGAAAATTGAGTCTCTTTGATTTGAGAACAGAAG
ACAGTCTTGTAACTAACACTGGAAGATCCGAGCATTTCAGTAGTATGCCTGTGTAATTAATTTTTAAAACCCGTTTAATTCAGTGATTAAAATTAATTATTAATGCTC
AATTGAACCTGCATCTACGGTATGTACAAAAAATTTGAGAAAGGAAAGTTTTCAGGCTTGGCACGTACTCTAGCTTCAAACATTTTATATTTTTCCCATGTTCCTTTA
ATGAATCAGAACAATTCCTATATAAATATATATAATCCAATAATTCTGTATTTTCGAAAGTAGTTCAGGTGTAAAACTACCCTCTGAACCCCATAATAAACACCTTTA
TTTGAGTATACTTCTTTAATTTCAATATAATCACGAGTAATGTCCGCAATGGTATTTAAATTTTTATTTATTTATTTATTTGATTTACAAAAGTGTTATTTTGAACCA
TCAAGTTGGTACTTTCAAGGCCAGGTTTTACACTTACATGTGTTAGAGTAACACATGAATTTCAAAATTGAGTATAAGGATGGCACATTTGAGTGAAATGTCAAGCAA
TTTGGAGAGTAAATGCCCAAAGCATGTCTTGTAAGGGTATTTTTCTCAGTGAATGTAAGAGAGCTTTCAGGGGGGTTGAAATTCGTGCCAGCGCGAAGTTGATGATAA
AAATGAAGTCGGAAACTCTTTATAAATGTTTAAATAGTTTCCTCCCAATGAATTTTTTTATGACGCTGTATTTTTCAG**TCCGTCGCTCCTTACGTGCGATCCTCACATT**
GGGGATAGCCCGAAAAAAAGGGGCGGATGGGGTCACACCAGGTGAAAATGGACGATGTGGTGTGCTGGAAGAGGCATACAAGATGATGAAGGTGGTGGACGTTGCATGCT
AGTAAATCGCCCCTTCATGCAAAAGGTACATAAACACTATCAGCATAATTCAACAGCAAACAATTCCTTCGCAACACTTTTTGCACCCTGCACCCGTGGGGCAAACACG
GGGTGAGCTTCCATAAATGAAGTTTAA

>PPAI006902_a-PA (>50 aa; >1 exon) | EDITED (PARTIAL GENE - C-TERMINAL MISSING)
MSNSQPILYSYWRSSCSWRVRIALNLKEIPYDIKPISLIKAGGEQHCNEY

####### PPAI006902 - partial GSTZ (N-terminal; isoform B) #######
>AJVK01059004.1 reverse | **PPAI006902** (pre-editing) | PPAI006902_a-PB (post-editing)
TGCTAAAAGGGTTCAAAAAACGGCTTCAAAATTAATTACTCAAAGTACCACAACGCCATGAGTTTATCGGCGGCGATTTCAAAGGTTTTTCCACAGCAAACAGTAGAA
ATGTCACAGTGATTTTTCACACCCTCCTTTTTTCACGTATTTTAGCGAATTTGTGTGATATTTTCCACTGTAGTTATCTATTGCAAGTTGCTATGGCAAGCATCAGTT
GTAAATTGGTACAGTTTTCACTGTATTTTATTATTTTTCCTTTGTTTTCCTTCTGGCCTGTAGATTGAGGTTATGTGTGTTTTGTGGCTTTTTTTTCTTCCCGGAGCA
TAAATGTTTATTTTCGCTTTTGTTTTTATACCTCTCCCATCGCTAGGTGGCTTGTTTCCATCTGCGATGGCAGGAGAGGTGTGCTAGCTTGACTTGTGGCATGAAAAT
CTAACAGCCGGCGATAGCGCTGATGACCTTGAGTAAAATTTCCTCCTAGATGACAATAAACAAGCTTTCCAGCCGGTTTTTTGTATAATTGAGAGATTAGTATTTTTT

TATTTCCTCCGGCTTGAAATATCACCTCAATGCATGATGCCTAATCTTCAGTCTTGGCCAAGTCGTGGAAGTGTGCTATTCTGTTTGAAATATTTTTGCCTGAAGAGA
AATCCAGAAGGTTTCATAATCTGCAATC**ATGTCCAATTCGCAGCCGATACTCTACTCATACTGGCGTAGTTCGTGCTCCTGGCGTGTCCGGATAGCACTCAATCTTAAGGA**
GATCCCCTACGACATAAAGCCCATCAGCCTTATCAAGGCGGGCGGGGAGCAACACTGCAATGAGTACAGGTAAGAAGAAGCTTGTTTTACAAAAAGAATATGCTCCTATA
TGCAATCCCAGCGTCAAATAAGAAAGCCTTATACCTATAAGATTATTGTGCCAGGAAACAAAGTAACTAGAGGAAAGTTTAATTAGCTGCATGCGAGTTACAAAGTAC
TAACAAAATAAACAAATTCTCAACAAAGAATAGGGGAGCGTCAAAACCTTTGTAAATTCCACAAAAAACACACAATTATAAAGAATCACGATAAAATAGGAAGAACTA
CAGCGAATTTGAGCAAATTAGCTTCATAATTAAACGTGAAAATTGTCAATAAAATTTTTCGCCTGATTGAAAAAGAGCCGATTAAGTGAGAGTCTACTGTACAACTTT
TAAGGACCGTAAAAGTACTCGCTACGGATAAGTGGCGCTGCCCAGTGAGCACCAAATGTTAACCGATTTCAATTCAACTGAAAATATATGTATTTTCAGCTGAATTCT
GCTAATCTTAAAACAAAACTCACGAGCCAGTGCCATTTCCATACATTTGATTAGCATTTTTTGTTTAAGAACTGCTAACCAGTCAGCATATTTTTGCTGATCGATTCA
GCACAAATATGCTGAAAACGCTGCCTTTTTCAGCTGTGTGCTCGCTGGATGGACTATGCATTCAAATCAAGGGGTAACTCATATTGAGAAACCCTCGTCAATTGTCCG
AGAAACGCTTCGCGAAACCCGAGAAACCCAAAAATTGCATCGCGAAACCCGAGAAACCCAATTTCTGCATCGCGAAACCCGAGAAATCAGAAACCCTTGTCAGAAAAA
ATGGGAATGAGTTACCCCTTGGGGGCGAAATGTCATCCGGTTTATCGATAGATTTGTCAATTGGGCATTTGAGAGAGAGACTACTGTAAAGTCAATACAAGAACTAAA
CAAGGAATAGCATAACCTAGTATCTACATTAAGACAAATAGAGGGTAAAATTAACTGTTTTTATCATATAATTATACTTTTTAAGTTTTAAAGCATAACAACACTTTT
TTCATGTTAATTTTAAAACTTGTTAAGGGTAAAATTCGCATTACCAGAATGAAAAGTTTAGACATGGTTTTTGGTCTGGTTTAGACCGTTTAGGGTGCATTTGACCGT
CATTGTACAATTTTATTTAGATTTTTATGTAAAGGTAAGGATATACCCTATTTATCCAATTAATAATCTCATTACCTGATTAACGAAAAAGCGTTCTCGAAATGTCGT
TGAGTTTGGAAAGGAATAATCTAAACTAAACCCGTAAATCCCGAAGCCAAAATCCAGAAAAGTCAAAATCCCGAAGCTAAGGTCCCGAATGATCAAAATCCTGAAAGG
GACGAAATAATAAGCAGGATAATGTGTGAAATAATTTCCCAAAACAGAAAATTTCTCTTTGTCTCCAAAAAGTGCGGGTGCACACGTTAGAGTGGCTGTGAGACTTTT
TAAAAATTGAAGCTTTTGGGTTCAAGAAAATCTTCCAGCATCGACTACGAGACCAAGAGATAGACCACCTAAAAGGTGGTATGAGTCCTGGATATCAGAATCACAACT
CCTGCTGATGCTAAGTACCATGCCCCCTTCAAATACTCAAACGACGGCAAACAATCCGTAGCCTAACTGTGGAAAAAACAGCTATTTGTCTAATTAAAGTAGAAGAAG
AAAAAGAAGATTTTAGCTTTCGGGATTTTGACCGAGACTCAATCTAAACCAGATCGAAGACTATGTTCTAAATTTCGTTAACTTTAAACTGACTAACCTGTTCCTATT
AACCCTATAAGGATCAGTAGTATACCGGTGTCCCTAAAACAATTTTTTTTTGCTTTAGGAAGTCATGTCTTCTAAATAGGTTTGTTTTTGCTTGTTCTCCACTGTGTT
TGTTTGTGCTCCACTTGTCCTTAAAAGTTTATTAGAATTATTTTCTACTTACTCGAATTCGAATAATCAATGAAACTCCAGATACGTCTAATTTTGACTAAAAAAATA
ATTGGTAATGGTCCCAGGATTTAAAAAAATCTTGTTCTCTTTCTACGGTAAGGTCAAGAAGGTTACAAATTTTATTTTCAAAAGCACACTGTGAATGATGAGACTTAG
CTGGGTAGAACGTTATTGAAAATTGTAGAAACCGGTTCTTCCATAACTTTTGAAACGGTCATATTTTGAAATTTACGAGTACTTTTCTGTAGATAGAACAGCACCGAA
GGGAACGTTTTCGCGTGGAGGGGAAGTCAGTCTTGGTTAGAGCGAGGAAGAGGATGCATTGCGCTTTGTATCCTGGGTCTGATAAGAATGATAAATTGCACATCCCAA
GGCCCAGGGCAACAAAATGTCATATCTTAGTTCCCAGGGTCGAGTTATAGAGCAGTCCGAAAGCATTCCAAATCAGGATCTTTAGCTATTTAGGTCTTATACACAAAA
ATGCACTTATAGACTTTTTGCGATCTTTCCGTGGTGTGCTTTCACCAAGATTATCAATGAATTATTTAAGTCCGAATAGGGAATTTACGAAGTTTCATTATGAAATTC
GATATCAAGTTGTTATTTTTCCTTGTTAGCATTAATAAAGGACGTGATCCAAAGTATATACTCGCCCTTTATAAACAATAATGTTACCGTAAGTGCGCGTGACTTTGA
CACCCCCCTGTTCGTATGCTTCTCTTTACTTCTAGAACTTAAGGATGGTCTTTACCGAATCGATCTGCCATGTTTGATCAGTAAAAACAATCCTTAGGTTCTAGAAAT
AAACATAAAAGATTACGAACAGGGGGCTGCCAAAGTCACCCTCAAGGGACAAAGTTCCTCATGAGAAATACTAATATCGTAAAATTTATATACATCACTGATTAATAG
TAGCACATTGTTCCTGTACTTATACAGCGCGTGTGATGAGTCTTATAGATCTAATCACATTAGTGTTGAAAAGAAGCATATTGTGCTTATTTTAACCCACGAACATGC
TTGATATTAAGAGATTAATATAAATCTGTGCTAAACAGTAGTGCATTATCTTGGAATATTGTATGTGAACTGATTATGTATAATCTTTAGTTACTACGGTGTTCGTTA
GAGGATTGCTTTTTTCTTAAATAAAAAAGACTCAATTTTTTCTAGAGCTTTCAACCTTCGGTATTTGTATTTATCAGTAGGTTGGTTATAATCTTCTAAATGATTTGG
AATCGTCTCGTCAGTAGTATTGTGCGAAATCTGATTCACAGAGGAAAATTCATACTGGTGATCGAAAGCTCTGGAGAAAATTGAGTCTCTTTGATTTGAGAACAGAAG
ACAGTCTTGTAACTAACACTGGAAGATCCGAGCATTTCAGTAGTATGCCTGTGTAATTAATTTTTAAAACCCGTTTAATTCAGTGATTAAAATTAATTATTAATGCTC
AATTGAACCTGCATCTACGGTATGTACAAAAAATTTGAGAAAGGAAAGTTTTCAGGCTTGGCACGTACTCTAGCTTCAAACATTTTATATTTTTCCCATGTTCCTTTA
ATGAATCAGAACAATTCCTATATAAATATATATAATCCAATAATTCTGTATTTTCGAAAGTAGTTCAGGTGTAAAACTACCCTCTGAACCCCATAATAAACACCTTTA
TTTGAGTATACTTCTTTAATTTCAATATAATCACGAGTAATGTCCGCAATGGTATTTAAATTTTTATTTATTTATTTATTTGATTTACAAAAGTGTTATTTTGAACCA
TCAAGTTGGTACTTTCAAGGCCAGGTTTTACACTTACATGTGTTAGAGTAACACATGAATTTCAAAATTGAGTATAAGGATGGCACATTTGAGTGAAATGTCAAGCAA
TTTGGAGAGTAAATGCCCAAAGCATGTCTTGTAAGGGTATTTTTCTCAGTGAATGTAAGAGAGCTTTCAGGGGGGTTGAAATTCGTGCCAGCGCGAAGTTGATGATAA
AAATGAAGTCGGAAACTCTTTATAAATGTTTAAATAGTTTCCTCCCAATGAATTTTTTTATGACGCTGTATTTTTCAG**TCCGTCGCTCCTTACGTGCGATCCTCACATT**
GGGGATAGCCCGAAAAAAAGGGGCGGATGGGGTCACACCAGGTGAAAATGGACGATGTGGTGTGCTGGAAGAGGCATACAAGATGATGAAGGTGGTGGACGTTGCATGCT
AGTAAATCGCCCTTCATGCAAAAGGTACATAAACACTATCAGCATAATTCAACAGCAAACAATTCCTTCGCAACACTTTTTGCACCCTGCACCCGTGGGGCAAACACG
GGGTGAGCTTCCATAAATGAAGTTTAA

>PPAI006902_a-PB (>53 aa; >2 exons) | EDITED (PARTIAL GENE - C-TERMINAL MISSING)
MASISCKLPILYSYWRSSCSWRVRIALNLKEIPYDIKPISLIKAGGEQHCNEY

####### PPAI000943 - partial GSTZ (C-terminal) #######
>JH662192.1 forward | **PPAI000943** (pre-editing) | PPAI000943_a (post-editing)
CACTTTCTCGCTTTTTGCATTGTCTATCAAGGCACCTGCAGGGGTTCAGGTGGCCAAATACCTACCAATAATGAATAGATAAAGATCTTTAATAAGAAGATTGCATGA
ATGACTATTTCCCTTGAAATGTGCGATTGGTCAAAAATATCTTTCAAACTAAAGATAAAGAATATTAAGAGAAGAAAAAAATCTATCAATAGATGGACACACGCTGAT
TGAGTCCCTGTCAATA**ATGCACTATCTGGAGGAGACTCGTCCGCAGAGACCACTTCTCCCCCAAGATGTTCACAAGAGAGCGAAGGTGCGTGAGATATGCGAAGTAGTTGC**
ATCGGGTATTCAACCCCTTCAGAATCTCGTTGTGCTCATTCATGTGGGGGAGGAGAAGAAGAAGGAATGGGCTCAACATTGGATAACTCGTGGCTTTAGGGCCATTGAGAAG
CTCCTTTCGACTTCTGCTGGAAAATTCTGCGTAGGTGATGAGATCACAATGGCAGATTGCTGTTTGGTACCTCAAGTTTTCAATGCTAGACGGTAAGTTTATTCAGCTTTT
CAAACTCTGGGAATTACTTCAAGATTTTGAAAATTTTCATCAGCATTCATGAAGATTCCTTGAATTGCTTGAATTAACAAAAAACCCTATCAACACTCAGTTGACTGT
TTCAGTTGTTTTAGATCTGTTTCATGTTATTTTGTTTCCATGAGAAATTAAATGCAATAATGACGCCGAGAGTATTGATCAGGTGAAGAACTAAGATTAAGTGGACAT
TAATATATTGTAAATATTGTCTTGCTAGAAGGTTGTTAAGTCCTGTGCTAACAAAGTGTTTAGCCAAAGAAATTGTTTATGTTCAAAGTTCTTAATTGTTCTAAAGTT
GTCTAAAAGTGTGTAAACTCAATGTACCCATTAAAGACATCATCCTTGTCCCCGATTAAGACGAAGGCCCAGAAAAAGCCTAACATCTTCAACCACTGAAGAATGTGC
AGAGAGCACCGAAAGTTCTGGAAAGAAAAGAATATTTATTCTGGGCTGAAGCCTCTCCCATCCGACAATCATCGGATTTGTTCTTTGAATTAATATCAACATCGGTTT
CCTACATTTATCATTATTGTGCTGTAGGGGAAAGTACTCTCCCTTTGAACGTTCATGCCTTCGAATAATGTGAATTTCTTTAATGTTTCCTAGACTTACACATTATTA
TGACATAATTATCAATAATTGATAAAAAGCTAACTAATATTCAGTCGAAATGTGTAAATCTCTTAGAAAAAATAAAAGAAAATTTACATTATTCTAAGGCATGAACGT
TCCAAGGGAGAGTACTTTTCCCTACTTCTATGCGCACTACAGAAAAATACTGCCATAATAAAGTATTTGGACCACTAGTCTATAATATCCAAATAATTTTTCTTCTGT
GAGACACTCGGTCACATAAAACCTGATCATAGAAGATGTAGCACTATTAAGGTCGCGGAGTAGGGTTTAGCAAATAGAAACCTTCTCCCTCCTTGCCCGTACTTGCCT
TTGACTCCATTGAGTGGCTGAGAAAAGAACCAGGTTAAGGTTATCATGGAGGATACCATGTTTGCCATGTGAATAGAGAAAGGATGGTCTAAGAGTGTTTCTAGGACC
ATGTAGGAAAATATGAGCGTTTTCTTCTCTTAATGAAGAGGTTGTAGAACACGATCTTTGTTCAGTGATGTCCATAGTTGTGAGCACATGGGCTGCAGTAAACAAGAC
AACTTTAAATGTTCGAAGGCCTAAACTGCCAACTACCGTTTCGATGACCTCTCTTGGCTCACCTAATATGCACATAGCAATATAGAAGATTATTGAAAGTGGTTTATT

GTGCTTTTCCAGTGAAAAATAAAAAGGGAACATTTTTCTCCTGAATATTTTCTTTACTACATGACTGTTCTCATTTGCTTCTGCGTAATCAACTTTATGTTGGTTCCT
GTCCCGACTGTAAATTGCTTTTATACGACGAGGACTGGGCAAAACAGTCGTGACAGGAACCAACACAATAAAAAAACCATAACGCATAAGCATATGAAAACAGCCATG
TACTAAAAAAAAAGGTTCAGGAGAAAAATTTTATCCTTTTCATTTTTCTTTGAAACAGCATTTCGTGATGTCCTCATTTTAAAAGAAAATTAGAAACACTAGACAGACC
TATGGGATAAGTCGAGAGACTTTAGTAGAAAAAGGATATCTCTGGGCTAAGGGCAGAATNNNNNNNNNNNNNNNNNNNNNNNNNNNNNNNNNNNNNNNNNNNNNNNNN
NNNNNNNNNNNNNNNNNNNNNNNNNNNNNNNNNNNNNNNNNNNNNNNNNNNNNNNNNNNNNNNNNNNNNNNNNNNNNNNNNNNNNNNNNNNNNNNNNNNNNNNNNNNN
NNNNNNNNNNNNNNNNNNNNNNNNNNNNNNNNNNNNNNNNNNNNNNNNNNNNNNNNNNNNNNNNNNNNNNNNNNNNNNNNNNNNNNNNNNNNNNNNNNNNNNNNNNNN
NNNNNNNNNNNNNNNNNNNNNNNNNNNNNNNNNNNNNNNNNNNNNNNNNNNNNNNNNNNNNNNNNNNNNNNNNNNNNNNNNNNNNNNNNNNNNNNNNNNNNNNNNNNN
NNNNNNNNNNNNNNNNNNNNNNNNNNNNNNNNNNNNNNNNNNNNNNNNNNNNNNNNNNNNNNNNNNNNNNNNNNNNNNNNNNNNNNNNNNNNNNNNNNNNNNNNNNNN
NNNNNNNNNNNNNNNNNNNNNNNNNNNNNNNNNNNNNNNNNNNNNNNNNNNNNNNNNNNNNNNNNNNNNNNNNNNNNNNNNNNNNNNNNNNNNNNNNNNNNNNNNNNN
NNNNNNNNNNNNNNNNNNNNNNNNNNNNNNNNNNNNNNNNNNNNNNNNNNNNNNNNNNNNNNNNNNNNNNNNNNNNNNNNNNNNNNNNNNNNNNNNNNNNNNNNNNNN
NNNNNNNNNNNNNNNNNNNNNNNNNNNNNNNNNNNNNNNNNNNNNNNNNNNNNNNNNNNNNNNNNNNNNNNNNNNNNNNNNNNNNNNNNNNNNNNNNNNNNNNNNNNN
NNNNNNNNNNNNNNNNNNNNNNNNNNNNNNNNNNNNNNNNNNNNNNNNNNNNNNNNNNNNNNNNNNNNNNNNNNNNNNNNNNNNNNNNNNNNNNNNNNNNNNNNNNNN
NNNNNNNNNNNNNNNNNNNNNNNNNNNNNNNNNNNNNNNNNNNNNNNNNNNNNNNNNNNNNNNNNNNNNNNATCCCAAAAATATTCAAGTTCTTGTGCTTGTGTTTTG
GTAAAGAATTAGCCATCACAATAAATTATTTGTGTCTTCTGACCCTTTCTAAAAGAAATAGGAAACAAAAAAAGTATCAAAAGAACGAGAATTGAGATAATTTTCATA
AAAACTCTAAAAGATTCCGTTAATAAGGATCGTATGGGAGTAGGAAAGGAGTTCCGTAAAGCGTAAACACAACTATTAACATGATTTATAACAGATCGAAGTGATATT
TAAGAATGTTAAATAGTAGTACAATTCAATAAGTAGTTATAGAAAACAAAGTCTGGAAATCCTAGCCATAAATACATGCTGGATTTCAAATGTAAATCTCAGCTAAAA
GTTAAGTATACAATGGTTGTAACTTCACACGAAAATTAACATGTCCAAAATCTAATCTAATTCAAACTTTTCGACTACAATGTACTCTGGTAAGAATAGATTCGATTT
TGGGACAATCCAACATCAATATGATCCGAATTTAGCTTAAATCTCTACTCAAATTTTCTTTTAATAGTTTCAAATAGACTACTAGGAAACAGAATTGTTTAGTTATGG
TTTTTAAAATATTTGTAGTATTGAACTGTTTTATTTATTGGAATTTACTATTTAATTTATTAGAACTATTTTATTTTTAGGCATAGAAGCGCCAGATTTAATGGGAAT
GTTTCTTTGTCCCATTTATTAGGTTTTTTTTTTTACTCTGAATAAAAAAGTTTTATTAAACGGACAAATGTCTAATTCTAATTTTGCCAAAAGAATGTTATTTATTTAT
TTGACAAATGTTTTTTTTCATATTTTACATTAAAAAACATCCTTTTGACAAACTTTTGTGTTAATTTTATAAGACTTTTTTTCAGAGTAGCTTTGTGAATATTGAGAG
AGAATTTAATGAATTTTAATAATGAATAATGAATTTAATTACACTATCTATTGATTTGATACAATTTTAATTTTATTTAAATACTTCACGTTCTTCTGATATCGGCTT
TTGTATGAGAATCAAACCCACGGCATGTAAGTCACAGAGCCAATGCTTTGTTGGCCAAAGTACAGGGTTCCATGGGTTCCATGTGATAGTTTATTGATTGGATTCATA
AATCTAAGTCTTATTAACTATATAAGTTGTTCTAAATTATTATTATCGACGTCGAAATTGCATTTATCACAGGATTATTACGTCGAAACGTTGAAACAAAAATAACAT
GCAACATTCATTGCATCCAGTGTGATGAATGAAAAAAAAACAACTGAGTGTTAAGAATTTCAAAGAAAGTCTTCTTAGGATTAGCGAGAATATCATTTTGAGTGATTT
TTTTTGCCTAG**ATTCCATGTGGATCTTAGACCTTATCCCATCATTTTACGCATCGATCGCGAATTGGAGAGTCATCCAGCTTTCAGGGCTGCTCATCCATCCAATCAGCCA**
GATTGTCCTCCGGAAGCCGCCAAATAAATAACGCGCACCCACTAAAAACAAAACAGAAAATTCAGGTTCAAAATAACACTCAAACTATTCTCTGTTTTTGTCTCTTTGC
CAACAAATTTTAGTTTAGAACGATGTGAAATGATAAAAGACATCCAAGAGTGATGTCCAACAAAGTTTTTAAATGATTTATTGCTAGAATTTATTTTAATAAAAATTT
GTTTTGACTT

>PPAI000943_a (>151 aa; >2 exons) | EDITED (PARTIAL GENE - N-TERMINAL MISSING)
GHTLIESLSIMHYLEETRPQRPLLPQDVHKRAKVREICEVVASGIQPLQNLVVLIHVGEEKKKEWAQHWITRGFRAIEKLLSTSAGKFCVGDEITMADCCLVPQVFNA
RRFHVDLRPYPIILRIDRELESHPAFRAAHPSNQPDCPPEAAK-

####### LLOJ000305 - GSTZ (isoform A) #######
>JH689424 reverse | **LLOJ000305** (pre-editing) | LLOJ000305_a-PA (post-editing)
TTGTAGTTTTGAGGTTGATTCCTTCACTCTTTTAAAATGATTTTTTTTTTAATATTTTATCAAAAATTTATTTTGATTTTAGAAAGATCAATCAATCTTGCAAACTCT
CACATTTTATATTTTTAATAAATTTTAGATTTCTTTCTATTCTTATTTCCGGCAATTTTATAAGCACTGAAGATCATTGAGAGAGCGATCTTATAATTTTGCAAACAT
TTTATGATCAATGCAAGATCTAACGACTTTATTAAACTTTTTAATTAGATAACATTTTTTATGATGTTACAGGATTTATTAATTTGATCACAAAGATCACGAAATAAC
TGAAAATTTACGATCCCAGAATTTCTTCTTTAAAGATCGCATTAGATCGCCGTTCATAAAAATATGCAATACCTAATTTTCTTATTATTAAAAAGATTTAATTTTATA
AGATTTCTTTTAATAATTTTTTTATCTTAAAATCATTAAAAACCTTAAAACCCTTCAAAATCTCACATCAGCACCCGTAAGTACCCCAGAAGTCCCAAAAATACATTT
CATTCTCAAAAATTGTCGGAAAAACAAGAAAAATCGTATTTTTGGAAAAGAAAAAAAACGTTAAAGTTCAAATCTCAGCAGTTTTTCCATTTTCGAATTTCGAAAGC
CGATAATTGCCGATAATTACCGAGAAAAGCCGAAGTTGCCCCGAAAGAAGGAGAAGGAAGGAAAACAAGAGAAAGTTTTGCCGGATTTGTAAAGTGAATTTTTTCGCT
AAAAAATTGTATAAAAGCCAAGCAATTGGCTGTGTCTTTTTGTGTTAAGCAAGTTCAATAAACGGCTTCAAAATTAATTACTCAAAGTATCACAACGCCATGAGTTTA
TCGGCTGCGATATCAAAGGTTTCTCCAACAACAGAAATGTCATCGTGATTTTTCTTTCACGCCCCCACCTTTTTATCCCGTATTTTATCGAGTTTGTGTGATATTATT
TGCTGTAGATTATATTTTGCAAGTTATT**ATGGCAAGCATCAGTTGTAAATTG**GTACAGTTTCCAATGTATTTCATTTTATTTACCTCTGTTTTCCCTGTTTGCTTGTTG
TTTGAGGTTATGTGTTTGGCTTGTTTTCCTTTCCGATTGCTTTGTTTTTGCTTTTTTTCCCTCTTCTGCCTGCCCCGGCTCCAGTTTCCGGGGAGGTGCTGCGAGAGG
AATGCGCCCAGATCACCACGCAAGAAGCACGACGATAAGGCTGATAACCTTGAAGGAGAATTTCCACCAATCAATGCGAGAACAAGCTTTCCAGCCGGTTTTTTGCAT
AATTCAGAGATTAGTTTTTCAACATTTTATGTTTGCCTCAAATACCAACCTGACAATGCATGATGCCCGTTCCCCAGTCTTGTCCACGTCGTCCGCTTGTGCTGTTGT
GTTGCTTGAGATCTTCAAAATAAAGTAGTCCCTTTGGAGAGAATTAATTAAAATCATGTCTAATTCCCAG**CCGATACTCTACTCATACTGGCGTAGCTCGTGCTCCTGGC**
GTGTCAGGATAGCCCTCAATCTCAAGGAGATTCCCTACGACATAAAACCCATAAGTCTCATTAAAGCAGGCGGGGAGCAACACTGCAATGAGTACAGGTAAGAATTCTCCT
TTATTGCTTCCTGTAGGACCTTGGAGACGTCCTGAAGCACTCAAAAGGATCCTTTATGGGTTTCTTATTGGCTTATTAGAGCTATTAAATTCAAGCTTCAATAAAAGA
TTAAATTTTATCACCTTGATAGAGATGTGACCTTGGCTTTAGCTCCAGATTAGGCGGTTTGTTGCAAATCCAATTGGTGGGTCAATTTTTTCTGTGGGACAACTTGAA
ATTGGTTTGGATTGAGTTTAGTTTGAGCTTCTTTTCCTCAATTTTAAACTTTTGATCAGACTGTTCTCATAAGAAACTTGATGAGAATTAACTTAAGCTTCCGATTAA
TTTACAACAAAGCTTAACCCTTTCGCGTCCTCATGAAACTCTCGATCTTCTCATCACGGTAATTATTCTGTGAAAGCTTTCAGAGAACATTTCTCACTAAGAACGTTT
TCTTTAGTCTAATTTACATAAATTCAACGCATTTCTCTCTTTGAAAAACAATTAAATTCATTAAAATTCGAGAAAATAACAAGTTTCACATTTGGGTCAACGTATGAT
CCGAACGCGAAAGGGTTACGTAAAGAAATAACTCTAGCTAGGCTCAGTGCAATTTCCTAATGAAAAATTAATTTAGAATATTAAAAATTTTTATCAGAAAAAAACGAT
TTTTCCTATTTCCTGAAGATTTTGTAAGATAAAATTGAAATTTTCTAATAAATATTATCTGAAAGCTCTCTTATAAAATTAATTTATTATCAAGATCATTTTCTCCTC
TCAATGCTTATCATGTCAACGATAACACATTATGATTTTTATATCATTTGAAAGGGATTCCATTTTACAAGCTTGCAGAGAGCACGCCAAGACGTTTTATGGCTTTTT
AATTATTCTATGGCCCTTGATAAAACTTTTACGATTGCTGTATAACAATGTTGAGAGAAGCTTTTAATTTTATTCTCAATCTTTTTAGCATCTAAAAGTAAACTTAAG
AGTTTTTTTTATGAAAGAATTAACTTTATTTATCTTTCATGTTTATTTGAGTAAAGACCGTGAACTTGTCTCATTTTCAAAATAAATTAAACAGAAAATGAATTTTAA
TTTAAAAATTTTCTTTGTAG**GGAGGTCAATGCAATGGAACAAGTACCAGCACTCCAAATAG**GTAAGTCTTTTTTCATATTTTTTTTTCACGGAGAATTTATTTGGGGTGA
AGACACGAAGAATTTGCAAAAAGTAGTAAGTTTGAGAAAATCATCCCATTTATTTCTGGCAACGTGAGTACACAAACAGATGTAGCGCCAAATTAGCAGCTCTGAGCG
TCGTTGCCTATCCTGGTGGCGTCACCACCAAAGAGACTGGAAGCTGTGAGGACACAGTTGTGGAAGTTAATGTTGGCAATGTTCATTCCTGTGCGGACCTCATTGAGG
TAGTACGCAAGGGAGTCTGCCATGCGGATGCTTTCGCGGTAGTCCCATGTAATTTGCAGTTCGAAACGAATTTGATCCTCCCACGTGACGGGATTGTAGGTTCCAATC
GTGTAGAGGGTTTGCAGGAGGGTAAAACTGGCCATTCGCTGGGCGTAGTTGATTGTGCCAAAGTAGCCTGTACCCAAACCGGCAGAGTAGTCACTTTCTGCGAGAACA

GCGCAACGATTGATGCTCTGTCCAGCTTCGGCTTCGCGTTCCTGGAGACGCTCACTGAGGGCAATTAGGCAGGGATTCTGATTCCCAATCTCCTGGGCTCGCTGATTG
ATGGCATCACGAGCTGACTGAGCGGAATCTTTGATGGCTGTAACGGAGTCCCATGCTCTGTCGAGGGAAACTCGATTAAAGTCAGCAATCTCCTCGGAAGAATCCAGG
CGTTGATTGCGAAGGAAGTAGTTGAGGTCGTAGTGAAAGTCCGTAAAATCCCGATGGAATGCTCGCAGTTCCCCCGCAAATTGACTCTAAAATGAACAAAAAAAGGAA
ATTCTTTTGTAATTCTCACTTCATGAAGGGAAAGAAAGACTCACATTTGCAGCAGCAAAGGCAAAAAGAGCCACGAAAGAAAGGAGAACCTTCATCCTGAATAAACTT
CCACCCTCAATGAGACAATGAGCTCAAGTGAATGCCAAGGGAAAGAACATACTGAACTACTTTTTCGCAACATCTCGCACTTTTCGATATGACTTTATCACAAAACAT
TTCCTACACATTAATTTAATTTGGGAGATTTTCATTTGATTACCCCACAACATGGGCACAGCTGTGTTGTATTGATTCCCTTTCAATTTCCTTGCATAATAAATGGAC
TGTTGAAGGAAATAAACTGACCTGTGAGTTTCTTGGAGCTCAATGTTATTTTCACACCAAAAAAAAATTGTGCAGATTTAGCACCGTCAAAGCAATTTAGAGCAACAT
CAATGCAGGAATTATTCCACACACTGGAGGGATTTATTTTGAGTAATTGCCACATGCAAAGGATTCATGTTAATAGCTGCAATTGCGGTTGGTTTACATCAAGCTTCC
CTTATTCCATTGAAAATTCAATTTACCAAATGAATGCAGCTTTTGCTATTAATTCAAATCCATTAAAGTTTTATATGATTTATGATGTGTAATTTATCAAATATAAAA
GCTTTTACAGGTTTTTAAATGAAGGAAATAGTTTTAACAGAGAGAGATTAAATTGAGCAAAGAAAATTAATAAAGAGTCTTTAATATGCGATTGGGCTCTAGAAGTTT
TTTTTTTACAGAATCTACTTCTAATTCAATTGAGGAATTATTTTATTTTAATTTTTTTTTTAATTATTAATAGAATTTTATTTTCTCGGTCATTTGCAAATAAAAAAA
AATGAAAACACAGAATGAGATTTTATCTGAATTTTCTTTTTTTTCTATTAAAAATAGAGAGAAAAAAATTTGTAAAGAAATCCGAGAGAAAACTTCTTTCTCGTTCAG
CAATTTCATGTAAAAGCAAAGCCGAGAACAATAGCTAACAGATGGCGCTGACTTTTACTTGTTCTTTCTATGACAGTTCGGAGATTTTTATCTCCTTCATCAGGTCCT
CAATTGAACATATAGCTAATTTTTTTTTTATAGATTGTGAGCAATCAATTTTGAAGAATTGTGTTCTCTTGTCTCAATTTAAAAAAAATCTTTTCTATATTTAATTTTT
AACGGCTAAGTTAAACCAACAGAGAAAAGAACTTTTTTTTTTTAAACGGTGTTGATTAGGCTAGAAAACTTGTTAGAAAATATTTCACATTCAACCAAAATAGTTTCT
TTTCTGAATCAGTAATAACTTCTAGCTTTCTAGTATAAATCCTCATCAATATTTTTCACCCATAAAATTTCTCGGGAAAAAATATTTAACATGAAAGCTATTTTTTTT
GATCTAAAATCATTTTTATTTTGATGTTTCTAAATAAAAATTTTCCTGCTGCCAATTAGGAAAAATAAAATTTAAAAAAAAAACATTTTTTCTCTAATTATAATCCAT
TTTTTATTTTTTATCACCGTATTTGTTTCATAGAAAAACGTTTTTAGTAAGCAAACCCGAGAAGAACTTGCGGTTTGCATTTTTTGGGTATCTAATGAGCATGCTAAC
AGTGGGAGAGATCATCCCTCAATTGGTTTACATCAATGTTGTAGAGATTAGCCACAATAAGGAGGCAGTTCTCCATGTTGATGTTGCCCGTGGTCTTGCCAACCTCTA
GCATGTCGCGGTAGTAGTTGATGTAGTACTCATCGCGGATGTTCTCGCGATGATCCCAGTTCTCCTGAAGCTCGAAGCGGATGTAGTCTTGATTTGTGACGGCATTGT
AGTGTCCAAGGGTGTAGAGGACGAGGAGTTGGAGGAAGTGGGACATACGCTGAGCGTAGTTGATGTGAATGAAGAAGCCATAGTCGAGGGCATGCTGGAAGTCACGGT
GGAGGTTGCGAGAGCAGCTGCTGATGCTCACACCGGCCTCTTCCTCGAGATCTGCCAATCGTGTGCGGATTTGAGCAATGCAGGGATTCTCTCCCACCTCGGCAGCAT
GATTGTCAATGGCTTGTTCAGCACTCACGGTGATGAGACGGACGGCTGTTACGTCATTCCAAACTTGCTCAAGAGCTACACGGTTGGCTTGTCTGATGCTGTCACTGA
CAAGAAGGCGTTGATCCCTAAGCCAGTAGTCCACCTCCTCGTGGTATTCATCGAACATCCTGTGGAATTCCTCAATTTCTTGAGAGAATTGTGCCTAAATTGAATGAA
TAAAAATCCCCATTAAGTCATAAAGTCATTAAATTGAAAATGCAGCAGATTTTCTTACCTGTGCCGTAGCCACAACCAGAGCAATGAAAACTGCAAGGACCTTCATGG
TTGCTTGTTATTCTTTTCCAAGAGAGAATTCCTACCCACAGTTCCTTCTCGGACGGTGATTAAATGGCAAATGGTAGCACAGATCGTGTGCAGGGTCGCATTTTATTC
AATGAACAAGGAGGGGCACGAATCTGCGTGAGAAGGAAGACACAATCAACGAAAGGAAAATCAACCATGTTCAATCTCAACGCGATAAGATGTTTTCGTTTAATTACT
CAAAACAATTCTTGTCTCTCTGCGAGTACTGTTGGGGAGGGCGCTTCTGCTACCTTCCGTGCCACCTGCAGAGAAATCAGGTGGCCAAATTTTGGCGCAATCACCAAC
AAGAATCTCCAATATAGATGAATGAATGAAAAATATTCCCATCGTCGATTCTTTTTTCGTAGAAATTAATTTTTTGTTGAATTTTTTGGATAAAAAAAATTTGTAG**AT**
GGACACACGTTGATTGAATCGCTCTCGATAATGCATTATCTGGAGGAGACGCGTCCCCAGAGACCACTCCTGCCGCAGGATGTCCACAAGAGGGCAAAGGTGCGTGAAATTT
GCGAGGTGGTGGCATCGGGAATTCAACCGTTGCAGAATCTCGTTGTTCTCATTCATGTTGGGGAGGAGAAGAAGAAGGAATGGGCACAGCATTGGATCACTCGGGGCTTCAG
GGCCATTGAGAAGCTCCTTTCAACATCTGCTGGCAAATTCTGCGTCGGTGATGAGATCACGATGGCAGATTGCTGTTTGGTACCTCAAGTTTTCAATGCTAGACGGTAATTT
ACCTTTGACAAAGAAATCTTTAAGTTTTCTTTAGAGAAAATTGTTATAAAAATGTTTTTTTGTGGTTCAG**ATTCCACGTGGATCTTAGACCTTATCCGATAATTTTGCGC**
ATCGATCGCGAATTGGAGAGCCATCCGGCATTCCGGGCTGCTCATCCATCAAATCAGCCAGATTGTCCTCCGGAAGCAGCCAAATAAACCTCATGCAACTGCAAAATGAAA
TGAAAATCAGGTTCAAAAGTAGAGCTCAACTCTTTCTCTGTTTCTTTTCAGCTCTTTGCCAAGAAAAATCATTTAAAGGAATTTTTTAATTTTTTTAAATGAAGACAC
TTGATCAAAAATTTTTGATTGATGAATTAAATGATACATTTAGTCCCTTGTTTCTATGCAATTTATTA

>LLOJ000305_a-PA (216 aa; 4 exons) | EDITED
MSNSQPILYSYWRSSCSWRVRIALNLKEIPYDIKPISLIKAGGEQHCNEYREVNAMEQVPALQIDGHTLIESLSIMHYLEETRPQRPLLPQDVHKRAKVREICEVVAS
GIQPLQNLVVLIHVGEEKKKEWAQHWITRGFRAIEKLLSTSAGKFCVGDEITMADCCLVPQVFNARRFHVDLRPYPIILRIDRELESHPAFRAAHPSNQPDCPPEAAK
-

####### LLOJ000305 - GSTZ (isoform B) #######
>JH689424 reverse | **LLOJ000305** (pre-editing) | LLOJ000305_a-PB (post-editing)
TTGTAGTTTTGAGGTTGATTCCTTCACTCTTTTAAAATGATTTTTTTTTTAATATTTTATCAAAAATTTATTTTGATTTTAGAAAGATCAATCAATCTTGCAAACTCT
CACATTTTATATTTTTAATAAATTTTAGATTTCTTTCTATTCTTATTTCCGGCAATTTTATAAGCACTGAAGATCATTGAGAGAGCGATCTTATAATTTTGCAAACAT
TTTATGATCAATGCAAGATCTAACGACTTTATTAAACTTTTTAATTAGATAACATTTTTTATGATGTTACAGGATTTATTAATTTGATCACAAAGATCACGAAATAAC
TGAAAATTTACGATCCCAGAATTTCTTCTTTAAAGATCGCATTAGATCGCCGTTCATAAAAAATATGCAATACCTAATTTTCTTATTATTAAAAAGATTTAATTTTATA
AGATTTCTTTTAATAATTTTTTTATCTTAAAATCATTAAAAACCTTAAAACCCTTCAAAATCTCACATCAGCACCCGTAAGTACCCCAGAAGTCCCAAAAATACATTT
CATTCTCAAAAATTGTCGGAAAAACAAGAAAAATCGTATTTTTGGAAAAGAAAAAAAACGTTAAAGTTCAAATCTCAGCAGTTTTTTCCATTTTCGAATTTCGAAAGC
CGATAATTGCCGATAATTACCGAGAAAAGCCGAAGTTGCCCCGAAAGAAGGAGAAGGAAGGAAAACAAGAGAAAGTTTTGCCGGATTTGTAAAGTGAATTTTTTCGCT
AAAAAATTGTATAAAAGCCAAGCAATTGGCTGTGTCTTTTTGTGTTAAGCAAGTTCAATAAACGGCTTCAAAATTAATTACTCAAAGTATCACAACGCCATGAGTTTA
TCGGCTGCGATATCAAAGGTTTCTCCAACAACAGAAATGTCATCGTGATTTTTCTTTCACGCCCCCACCTTTTTATCCCGTATTTTATCGAGTTTGTGTGATATTATT
TGCTGTAGATTATATTTTGCAAGTTATT**ATGGCAAGCATCAGTTGTAAATTG**GTACAGTTTCCAATGTATTTCATTTTATTTACCTCTGTTTTCCCTGTTTGCTTGTTG
TTTGAGGTTATGTGTTTGGCTTGTTTTCCTTTCCGATTGCTTTGTTTTTGCTTTTTTTCCCTCTTCTGCCTGCCCCGGCTCCAGTTTCCGGGGAGGTGCTGCGAGAGG
AATGCGCCCAGATCACCACGCAAGAAGCACGACGATAAGGCTGATAACCTTGAAGGAGAATTTCCACCAATCAATGCGAGAACAAGCTTTCCAGCCGGTTTTTTGCAT
AATTCAGAGATTAGTTTTTCAACATTTTATGTTTGCCTCAAATACCAACCTGACAATGCATGATGCCCGTTCCCCAGTCTTGTCCACGTCGTCCGCTTGTGCTGTTGT
GTTGCTTGAGATCTTCAAAATAAAGTAGTCCCTTTGGAGAGAATTAATTAAAATCATGTCTAATTCCCAG**CCGATACTCTACTCATACTGGCGTAGCTCGTGCTCCTGGC**
GTGTCAGGATAGCCCTCAATCTCAAGGAGATTCCCTACGACATAAAACCCATAAGTCTCATTAAAGCAGGCGGGGAGCAACACTGCAATGAGTACAGGTAAGAATTCTCCT
TTATTGCTTCCTGTAGGACCTTGGAGACGTCCTGAAGCACTCAAAAGGATCCTTTATGGGTTTCTTATTGGCTTATTAGAGCTATTAAATTCAAGCTTCAATAAAAGA
TTAAATTTTATCACCTTGATAGAGATGTGACCTTGGCTTTAGCTCCAGATTAGGCGGTTTGTTGCAAATCCAATTGGTGGGTCAATTTTTTCTGTGGGACAACTTGAA
ATTGGTTTGGATTGAGTTTAGTTTGAGCTTCTTTTCCTCAATTTTAAACTTTTGATCAGACTGTTCTCATAAGAAACTTGATGAGAATTAACTTAAGCTTCCGATTAA
TTTACAACAAAGCTTAACCCTTTCGCGTCCTCATGAAACTCTCGATCTTCTCATCACGGTAATTATTCTGTGAAAGCTTTCAGAGAACATTTCTCACTAAGAACGTTT
TCTTTAGTCTAATTTACATAAATTCAACGCATTTCTCTCTTTGAAAAACAATTAAATTCATTAAAATTCGAGAAAATAACAAGTTTCACATTTGGGTCAACGTATGAT
CCGAACGCGAAAGGGTTACGTAAAGAAATAACTCTAGCTAGGCTCAGTGCAATTTCCTAATGAAAAATTAATTTAGAATATTAAAAATTTTTATCAGAAAAAAACGAT
TTTTCCTATTTCCTGAAGATTTTGTAAGATAAAATTGAAATTTTCTAATAAATATTATCTGAAAGCTCTCTTATAAAATTAATTTATTATCAAGATCATTTTCTCCTC

TCAATGCTTATCATGTCAACGATAACACATTATGATTTTTATATCATTTGAAAGGGATTCCATTTTACAAGCTTGCAGAGAGCACGCCAAGACGTTTTATGGCTTTTT
AATTATTCTATGGCCCTTGATAAAACTTTTACGATTGCTGTATAACAATGTTGAGAGAAGCTTTTAATTTTATTCTCAATCTTTTTAGCATCTAAAAGTAAACTTAAG
AGTTTTTTTTATGAAAGAATTAACTTTATTTATCTTTCATGTTTATTTGAGTAAAGACCGTGAACTTGTCTCATTTTCAAAATAAATTAAACAGAAAATGAATTTTAA
TTTAAAAATTTTCTTTGTAG**GGAGGTCAATGCAATGGAACAAGTACCAGCACTCCAAATAG**GTAAGTCTTTTTTCATATTTTTTTTTCACGGAGAATTTATTTGGGGTGA
AGACACGAAGAATTTGCAAAAAGTAGTAAGTTTGAGAAAATCATCCCATTTATTTCTGGCAACGTGAGTACACAAACAGATGTAGCGCCAAATTAGCAGCTCTGAGCG
TCGTTGCCTATCCTGGTGGCGTCACCACCAAAGAGACTGGAAGCTGTGAGGACACAGTTGTGGAAGTTAATGTTGGCAATGTTCATTCCTGTGCGGACCTCATTGAGG
TAGTACGCAAGGGAGTCTGCCATGCGGATGCTTTCGCGGTAGTCCCATGTAATTTGCAGTTCGAAACGAATTTGATCCTCCCACGTGACGGGATTGTAGGTTCCAATC
GTGTAGAGGGTTTGCAGGAGGGTAAAACTGGCCATTCGCTGGGCGTAGTTGATTGTGCCAAAGTAGCCTGTACCCAAACCGGCAGAGTAGTCACTTTCTGCGAGAACA
GCGCAACGATTGATGCTCTGTCCAGCTTCGGCTTCGCGTTCCTGGAGACGCTCACTGAGGGCAATTAGGCAGGGATTCTGATTCCCAATCTCCTGGGCTCGCTGATTG
ATGGCATCACGAGCTGACTGAGCGGAATCTTTGATGGCTGTAACGGAGTCCCATGCTCTGTCGAGGGAAACTCGATTAAAGTCAGCAATCTCCTCGGAAGAATCCAGG
CGTTGATTGCGAAGGAAGTAGTTGAGGTCGTAGTGAAAGTCCGTAAAATCCCGATGGAATGCTCGCAGTTCCCCCGCAAATTGACTCTAAAATGAACAAAAAAAGGAA
ATTCTTTTGTAATTCTCACTTCATGAAGGGAAAGAAAGACTCACATTTGCAGCAGCAAAGGCAAAAAGAGCCACGAAAGAAAGGAGAACCTTCATCCTGAATAAACTT
CCACCCTCAATGAGACAATGAGCTCAAGTGAATGCCAAGGGAAAGAACATACTGAACTACTTTTTCGCAACATCTCGCACTTTTCGATATGACTTTATCACAAAACAT
TTCCTACACATTAATTTAATTTGGGAGATTTTCATTTGATTACCCCACAACATGGGCACAGCTGTGTTGTATTGATTCCCTTTCAATTTCCTTGCATAATAAATGGAC
TGTTGAAGGAAATAAACTGACCTGTGAGTTTCTTGGAGCTCAATGTTATTTTCACACCAAAAAAAAATTGTGCAGATTTAGCACCGTCAAAGCAATTTAGAGCAACAT
CAATGCAGGAATTATTCCACACACTGGAGGGATTTATTTTGAGTAATTGCCACATGCAAAGGATTCATGTTAATAGCTGCAATTGCGGTTGGTTTACATCAAGCTTCC
CTTATTCCATTGAAAATTCAATTTACCAAATGAATGCAGCTTTTGCTATTAATTCAAATCCATTAAAGTTTTATATGATTTATGATGTGTAATTTATCAAATATAAAA
GCTTTTACAGGTTTTTAAATGAAGGAAATAGTTTTAACAGAGAGAGATTAAATTGAGCAAAGAAAATTAATAAAGAGTCTTTAATATGCGATTGGGCTCTAGAAGTTT
TTTTTTTACAGAATCTACTTCTAATTCAATTGAGGAATTATTTTATTTTAATTTTTTTTTTTAATTATTAATAGAATTTTATTTTCTCGGTCATTTGCAAATAAAAAAA
AATGAAAACACAGAATGAGATTTTATCTGAATTTTCTTTTTTTTTCTATTAAAAATAGAGAGAAAAAAAATTTGTAAAGAAATCCGAGAGAAAACTTCTTTCTCGTTCAG
CAATTTCATGTAAAAGCAAAGCCGAGAACAATAGCTAACAGATGGCGCTGACTTTTACTTGTTCTTTCTATGACAGTTCGGAGATTTTTATCTCCTTCATCAGGTCCT
CAATTGAACATATAGCTAATTTTTTTTTTATAGATTGTGAGCAATCAATTTTGAAGAATTGTGTTCTCTTGTCTCAATTTAAAAAAAATCTTTTCTATATTTAATTTTT
AACGGCTAAGTTAAACCAACAGAGAAAAGAACTTTTTTTTTTTAAACGGTGTTGATTAGGCTAGAAAACTTGTTAGAAAATATTTCACATTCAACCAAAATAGTTTCT
TTTCTGAATCAGTAATAACTTCTAGCTTTCTAGTATAAATCCTCATCAATATTTTTCACCCATAAAATTTCTCGGGAAAAAATATTTAACATGAAAGCTATTTTTTTT
GATCTAAAATCATTTTTATTTTGATGTTTCTAAATAAAAATTTTCCTGCTGCCAATTAGGAAAAATAAAATTTAAAAAAAAAACATTTTTTCTCTAATTATAATCCAT
TTTTTATTTTTTATCACCGTATTTGTTTCATAGAAAAACGTTTTTAGTAAGCAAACCCGAGAAGAACTTGCGGTTTGCATTTTTTGGGTATCTAATGAGCATGCTAAC
AGTGGGAGAGATCATCCCTCAATTGGTTTACATCAATGTTGTAGAGATTAGCCACAATAAGGAGGCAGTTCTCCATGTTGATGTTGCCCGTGGTCTTGCCAACCTCTA
GCATGTCGCGGTAGTAGTTGATGTAGTACTCATCGCGGATGTTCTCGCGATGATCCCAGTTCTCCTGAAGCTCGAAGCGGATGTAGTCTTGATTTGTGACGGCATTGT
AGTGTCCAAGGGTGTAGAGGACGAGGAGTTGGAGGAAGTGGGACATACGCTGAGCGTAGTTGATGTGAATGAAGAAGCCATAGTCGAGGGCATGCTGGAAGTCACGGT
GGAGGTTGCGAGAGCAGCTGCTGATGCTCACACCGGCCTCTTCCTCGAGATCTGCCAATCGTGTGCGGATTTGAGCAATGCAGGGATTCTCTCCCACCTCGGCAGCAT
GATTGTCAATGGCTTGTTCAGCACTCACGGTGATGAGACGGACGGCTGTTACGTCATTCCAAACTTGCTCAAGAGCTACACGGTTGGCTTGTCTGATGCTGTCACTGA
CAAGAAGGCGTTGATCCCTAAGCCAGTAGTCCACCTCCTCGTGGTATTCATCGAACATCCTGTGGAATTCCTCAATTTCTTGAGAGAATTGTGCCTAAATTGAATGAA
TAAAAATCCCCATTAAGTCATAAAGTCATTAAATTGAAAATGCAGCAGATTTTCTTACCTGTGCCGTAGCCACAACCAGAGCAATGAAAACTGCAAGGACCTTCATGG
TTGCTTGTTATTCTTTTCCAAGAGAGAATTCCTACCCACAGTTCCTTCTCGGACGGTGATTAAATGGCAAATGGTAGCACAGATCGTGTGCAGGGTCGCATTTTATTC
AATGAACAAGGAGGGGCACGAATCTGCGTGAGAAGGAAGACACAATCAACGAAAGGAAAATCAACCATGTTCAATCTCAACGCGATAAGATGTTTTCGTTTAATTACT
CAAAACAATTCTTGTCTCTCTGCGAGTACTGTTGGGGAGGGCGCTTCTGCTACCTTCCGTGCCACCTGCAGAGAAATCAGGTGGCCAAATTTTGGCGCAATCACCAAC
AAGAATCTCCAATATAGATGAATGAATGAAAAATATTCCCATCGTCGATTCTTTTTTCGTAGAAATTAATTTTTTGTTGAATTTTTTGGATAAAAAAAATTTGTAG**AT**
GGACACACGTTGATTGAATCGCTCTCGATAATGCATTATCTGGAGGAGACGCGTCCCCAGAGACCACTCCTGCCGCAGGATGTCCACAAGAGGGCAAAGGTGCGTGAAATTT
GCGAGGTGGTGGCATCGGGAATTCAACCGTTGCAGAATCTCGTTGTTCTCATTCATGTTGGGGAGGAGAAGAAGAAGGAATGGGCACAGCATTGGATCACTCGGGGCTTCAG
GGCCATTGAGAAGCTCCTTTCAACATCTGCTGGCAAATTCTGCGTCGGTGATGAGATCACGATGGCAGATTGCTGTTTGGTACCTCAAGTTTTCAATGCTAGACGGTAATTT
ACCTTTGACAAAGAAATCTTTAAGTTTTCTTTAGAGAAAATTGTTATAAAAATGTTTTTTTGTGGTTCAG**ATTCCACGTGGATCTTAGACCTTATCCGATAATTTTGCGC**
ATCGATCGCGAATTGGAGAGCCATCCGGCATTCCGGGCTGCTCATCCATCAAATCAGCCAGATTGTCCTCCGGAAGCAGCCAAATAAACCTCATGCAACTGCAAAATGAAA
TGAAAATCAGGTTCAAAAGTAGAGCTCAACTCTTTCTCTGTTTCTTTTCAGCTCTTTGCCAAGAAAAATCATTTAAAGGAATTTTTTAATTTTTTTAAATGAAGACAC
TTGATCAAAAATTTTTGATTGATGAATTAAATGATACATTTAGTCCCTTGTTTCTATGCAATTTATTA

>LLOJ000305_a-PB (219 aa; 5 exons) | UNCHANGED
MASISCKLPILYSYWRSSCSWRVRIALNLKEIPYDIKPISLIKAGGEQHCNEYREVNAMEQVPALQIDGHTLIESLSIMHYLEETRPQRPLLPQDVHKRAKVREICEV
VASGIQPLQNLVVLIHVGEEKKKEWAQHWITRGFRAIEKLLSTSAGKFCVGDEITMADCCLVPQVFNARRFHVDLRPYPIILRIDRELESHPAFRAAHPSNQPDCPPE
AAK-

##############################
####### GST microsomal #######
##############################

####### PPAI007443 - GST microsomal #######

>JH665380.1 forward | **PPAI007443** (pre-editing) | PPAI007443_a, PPAI007443_b, PPAI007443_c, PPAI007443_d (post-editing)

TTTTTCTACGCCGCACATCTGCTCAAGTACATAGATTTTCTATCGATGTATTGCCTTATGTAATAATGCTATAGCATGAGATACCGAACACCATTGAGTTTATCTCAG
TGCTAGTCGGTGCTGGATTCACTAAAGACGTTAGTGAGTTTGTGACAATAAATTCAGGAGTTTTTCATTGAAAAATAAATAAAATACTCAAA**ATGGCCAATATCCTCGA**
TCTTCTGTCCCTGGACAATCCTGTCTTCCGGCACATGGCCCTTTGGTCTGGTGTATTACTTGTGAAAATGCTCCTAATGTCCGGCTTTACTGCCTTCTTCAGAATTAAAAAT
AAAGTGAGTTCACTTTTATAAAAAGAGAGAAATATGATAAGAAAAAGCCACGTAACTCGACTAAAAACAAGTATTTAATGTTGAGAAAATAACTTTCACATTTTAG**GC**
TTTTAGCAGTCCTGAGGATTTGATTTTTGGCGGGAAAAAGCCAAAGTACGATGATCCTAGTGTTGAAAGAGTTCGAAGGTTTGTTTATACCAGAATTTATCTATTGTGGAC
AGTTTGCACTAAAAATAAACTAACTTACAG**GGCCCATCGGAATGACTTGGAAAATGTTATTCCATTAATTTTTGCTTCCTTCTTCTACACTCTGACCAATCCAACACCCTT**
CCTGGCCATCAATCTCTTCCGGGTTGCAGCCCTGAGCAGGATTGCCCATACGGCCGTATATGCCTTTGGACCAGTACCTCAGCCAACAAGAGCCCTAGCTTTTGGCGTACCT
TTTGTTATTACACTTTACATGTCGGTTCAAGTACTCCTGCAATTCGCATAAAAAACCGTGTGGTGATTTTGATAAATCAGCGATTATCCCAAGTGTTCCAAGACAAAAA
TCGGTTTTGTAGTTTTTATATAAAAAGAAAATACAGCCATAAAATCTATATATGTTTTATTTTTTTCTATTTGTATGTGTTTGTTTGCCGGGCTTTTCTAATAACAC
GCACATTTCAAAAAAAAAATCTAACCTTGAGCAAACAAAAAGAGTTTTGCTAGAACATTTTGCAAGATAAATTTATGAAAATCTTTAGTATCGTCAGTGTTTGTGCGGA
ATTAGAATTGATAGCATCAGTGCTTGGATTGGAAATATTAAAAAAAATTGTGCAATTACTTAATTCTCAGTTTTATTTTTTAATTGTAAAATGACAGAG**CTAAAGTATA**
TTTTTTCAGTGGAAAATGAACTTTTCCGAACTTATGCATTCTGGTCTGTTTTATTAATCATTAAGATGTTCGCCATGGTCGTTATAACAGGATATCTACGACGAAAAACAGG
GGTAAGTAAGATTGAACTGAACCGTCTAAAGAGACTTTATTCTCAAATAAATAAAACCATTTTGTATTTCTTAAAG**ACTATGAGCAATCCAGAGGATATTTCTGTGGCC**
CCGCCTGGGTCAAAAATCAAATTTGATGATCCGGATGTTGAACGTGCAAGAAGGTAAATATTCAAGGTATTCAAAGTATTTTTGCAAAAAAAGAGGCATTATCAAATGAC
AAGGATTTTGTATATTACAAAAACTTTTTTTTGCTAACCAGTTGGATGTTTTTGTAAAATCAGAAATCGTTAATCAACAGAATACAATTAAGAATTGAAGTAATTTTC
TACCGGCAATTACATTTTAATTTGCAAAGGTGCTCTTAAAAACTATCTTTACTGAATCTTTTTAG**GGCTCATCGCAATGATCTAGAAAACATCTATCCCTATATTTTACT**
TGGATTTGGATACCTTCTAACTGATCCTACTCCATGGGTTGCAGCCCTTGTCTTTCGTGTAGGAGCAATTGCAAGAATTGCCCACACGATTGTCTACACGGTTTATGTAATT
CCGCAACCAGCAAGAGCAATAGCATTTGGCGTATGCTCGATTATTTCATATTATATGGCATCACAAATTATTTTGTATTTTCTCTAAAAGGGTTTTTCGATGATTAATCAC
ATAGAACTTATTATTATTAGTTTTTATAAATAAAAATAGCAGCTAACAGGTTTTAAAAATGAAACGACTTTTATTGTGATTTTGTTCTGCTGTTTGTTAGACGTTTCT
CATCTCCAATACTACGTTGGCAAATAGACTAATATATGTTTTCGCAAAACAAAGTCGTTAATTTCCTTAATACATTTCACTAATTCATCAACCACAAACAAAATCAAG
GAATAAACACAAAAGTTTAATGAGTTTACGGGTAAGTAATTAAAAGTAAGATAATGTGTGTAAGGTAATTTTAGTTAAAATCGTTCGTTTTTAACACTAAACCTACCA
AGACCCGGTCAAATTGACCGGTTTAAAATTAACACATATTTAAACGGTACAATGTCACTATCAAGCTTTATGGATTTCTTAAAATATGCATGTACTATATTTTTCAGT
GTTTAAATTTTAATTTTTTGGTCTTTTCCAAGACAAATTTGATAAGTAGGGGAGACCGGGGAGGTTTGGGACACTTTTTCATGTTTTGACTTTGAGACAGTATTCCAA
AAAATCACAATAGTGTATTACAATTTTATGCGGTCTATAGGATACTTATGGGTACTGACTTTATAAAAAGTACTCGATGGAACTTGGAAAACAACTGAGTTTTCTTGG
AGAAGATAAAACATTTAACTTGATACTTTGTCCCAAAACCTCCCCCATTTGGGGCAGTGTGGGACACAAAAGGGGATGTTTGGGACGCATTTTTTCTCGAATAATTTG
ATTTATTGGAACATATTAATTAATTTTAAATGCTATATTAAAAATATTTCTAACAGAAATAGATATATTTAATATTTTATTTCATACTTAAAAATCAATATCAATCTT
ATTTGTACAGTAGAGTCATTTTTGGTCAATCAGTTATTTAATGTATTTTCTTCTTATTTACCATCCTAGCTTTCTTTACTTTTCTATTCTTAATATTAGAATTATCAT
CATTCAAATTCCTCAGGCGAGTAGATTCTCTAGGACACTCTTAGTTTTGAACTCATTGATGGATTCCTGTTTGATTTTACGACAACAGCGTGGTAGTTATTTGTCCTT
ATTTCTCTGAATAAGCTCTCACCTTGCCTTTTGTAGAAAACTTGTGAGATTTGTAGAGGATATTGCTCGAGAAATTTTCCAGAAAATAATTTTTAACGGGATTGGACA
AAGGTGAATATTCTCAGGAGAAGAATTTATTAGTTCCCAAGACATTGATGGTTTAATTGTCTCTGTTGGAGACAACTGCTCAACTGGTAAACTTTGTACAAGAGGGAG
ATTTCCAGTAGAAACTGGGCATTACACTTCATTTTGACAATAAACAATTCGACAATCTTGTCGAAAATCAATGTCCCATTCATACCCTTTCAGGTGTCCCAAACATCC
CCGCGAGTAGTTTTGCTATTTAAAACCTTTAAGTAAAAAACAAGAGCATATAATCTCTGAAGCTTTTATAAAAAATATGTTCCCAGATTATACTGCTAGCTAATGAGAT
AAAAAAGTTTTGATTAGTTGGAAAGTACTCTCTCTTCGAAGTTCATGCCTTCGAATAATATGAATTTCCTATTAGTTTTCCTAAGAGACTTACACATTTCTATTAAAT
ATTAGTTGGCTTATCATCAATTGTTGATAATTCCGTTATAATTTATTGTAAATCTCCTACGAAAANNNNNNNNNNTAAGAAATTTGATGAAGGTTGATTAGTGAAAGA
ACGTTACATAATTTGCATTTTATTAGAATTAAAGAAATTTCAATATTTTGAAGAATTGCCATTAGGGGTTCCTTGTCGACCCTATATTAAAAATAATAACTTTCAATT
TTTACAAATATTTTTAGGGCTCATCGCAATGATCTAGAAAGTATATATCCCTTCATTTTGGTCGGATTTGGATATCTTCTAACTGATCCTACTCCATGGGTTGCAGTC
CTTATCTTTCGTGTAGGAGCAATTGCAAGAATTGTCCATACGATTGTCTACGCAATTTATGTAATTCCGCAACCAGCACGACGAATAGCATACGAAACATGTTCAATC
ATTACGTATTATATGGCATTACAGATAATTTGGTACTTTCTTTAAATGCAAAAATAGTTCATTCCATTAAAAGAGGAAGGCTATAGCTACACTTACATTTGATAGAGT
TAAAACATTATCGATGATTACTAAAATAGAACTGTTATCAATTTCAATGATTAAATGTTGATATGTATTTTATTGTATGTACAGCATGACTCGTCTAAATGTTGTTTT
TCACAAATTATAATCTTCCATAACTTTGTCTATGTAAGCAAAATTAAAGGTAAGTCAATGTCACGTCTTATTTTATTTCTTTTATATTCTAAAATATGCATTGATAAA
AGAGTTTAGTGACATAAATTTAAGTCTTAATGAACGATAACCGTTTTTTTAAATCAGCAATTTGTCGACCCAGATGTTATTCAACAATCCATTTTAATTCACCTTTAA
GATTTATGGGCTACAATAAAAAATTATTTACTTAAACTTCATTTTTATCAATCCATTTTTTTTAATAATATTTTTCTTCTATGTTAGGATTTACCTATTTTGGTAGTAC
TTATTTTCAGTTTTTCAGAGGCCATGAAAAATAATTTTTATAGTTCTTCCATAAAAGCATCCTAAAAATTTTTAGTGACAGACTTTTAGTCAAGTAAAAATAAGCTAA
TCATTCTTTTATTTAATGGAGCATTTTTCAGAAGTGCAGAGTTCAAAGAATTTCATATGTGACTTGCTAGAGTAAAAACGGTAGAAAACTCTTTCTCAGGTTGTATGA
GCCTCCATCCTCCAAAAAGAGTTTGCAAAGAAAATTTAAAATAAGCAAAAATTGATTTTTTTAGGCTCCACCGATACAGCCCTACGCTTGCCCCGCCTGTGCCTAAT
CTTCTCCTATATTTAAGATGTTAATGAAGGATTAAATTTCAAAGGAACAATCATTGAATTTTGTATAAATTTAACTAATCAATCACCATACAAATAGTAAAACGTTT
TTAATTTGTGCGTTTTCCGCTGTTTCATGTCAATTTTTTAAATTTATTTTCCCTATGAAAAACAATTTACATTGACCTTTTCAATATTTTACTGGTTTCAAAAGTAT
TAGCTCAAAAGCGGTAATAAGAATAACAATAATTTCTCGTTTACAATTAAATAATTAATGAAAAGCTAAGAAACTTTTTAAGAAAATAATACATTCTTAAAAACATAA
AATAAAAAAAAACAGAAAATAAACACATTAAAAAGCTTTAAAAAATGTACAATTGTACTAACAAAATTTTAAAAATATTTTTTATTATTTTTAGAAAATATTAAAACT
GAAATATCTTTTACTTTCTTTTGCATTTTAAGGATTCTAGATTTTAAAACAGCACTATTGCTATTTTTAAAATGTTACGGGTCCCGGTCAAAATCCCGAATTCTTAAA
AGTGTCACAGCTACTTCTACGATTCTACCCGCGCTTGCTGTAGGCAAAGAGAAATCTTCTGTATCTTAGGAAATTATTCTAAACATTTTCCTCCATATAATTTAATCA
ATTTCAGGATTTTGAAAATTCGGGATTTTTGCTTTCGGGATTTTGACCTTTTCGGGATTTTGGCGTTCGGAATTTTGGCTGCCACCGAAATATATGGATGTAGTTCAT
GCGAACACTTCGTTCATAGATACCTATGATAGAACAAAATTTCGAAAGTCAAAGTTAAACCACTGTAGAGGATCTATTTCCAAACAAATTTGGTTAAATTTAGATATT
TTGGAAAGGTCTTGGAATTTTCAACGCGACTGCATCGACTGCATTGGCACCTATGACAGAAAAAAAATTCTACATCGAATTTTCGAAGTTCAAAGTTAAACCGATTTG
TTTAAATTTGGAAAGTAAAATAGGAAGTAATAGGAAGAACTGAGTTCTAGAATTTCAAAAAGCTGGAATGCTTTGCTATTTCTTTAAAAATACTTTCGTGTTTTTTTT
TTTGTTTTGACTATTGTGATATTATTAAAATCGAGGAAATAATGAAGAATATATTATGTTAACATGAATTTTTCTTCTCAATTAGTCTTTTTATCACAACTCTGTAAT
CACAACCATTAAGAATATAAACTGAAAATCTAAATATTATGTCTTATTTATTTCACAGAGGTATTATCTATCAAAAGGTACTACACAGAAAAAAATATTTTGTAAAAA
TGTTCGTAAATGTTTGTGAATTCCTATGGTGGAATTACGAAATTCTCGTGAAACCCACAAACAAGTTCGTAAAAGTTTGTACTTTTTTCACAAACATTGTTCGTAAGA
TGATCATTTGACGAACATTTTTTTATACTTTTACAAACATTTGTTCGTAAATGTTTGTGAATTCCTANNNNNNNNNNNNNNNNNNNNNNNNNNNNNNNNNNNNNNNNN
NNNNNNNNNNNNNNNNNNNNNNNNNNNNNNNNNNNNNNNNNNNNNNNNNNNNNNNNNNNNNNNNNNNNNNNNNNNNNNNNNNNNNNNNNNNNNNNNNNNNNNNNNNNN
NNNNNNNNNNNNNNNNNNNNNNNNNNNNNNNNNNNNNNNNNNNNNNNNNNNNNNNNNNNNNNNNNNNNNNNNNNNNNNNNNNNNNNNNNNNNNNNNNNNNNNNNNNNN

NNNNNNNNNNNNNNNNNNNNNNNNNNNNNNNNNNNNNNNNNNNNNNNNNNNNNNNNNNNNNNNNNNNNNNNNNNNNNNNNNNNNNNNNNNNNNNNNNNNNNNNNNNNN
NNNNNNNNNNNNNNNNNNNNNNNNNNNNNNNNNNNNNNNNNNNNNNNNNNNNNNNNNNNNNNNNNNNNNNNNNNNNNNNNNNNNNNNNNNNNNNNNNNNNNNNNNNNNNN
NNNNNNNNNNNNNNNNNNNNNNNNNNNNNNNNNNNNNNNNNNNNNNNNNNNNNNNNNNNNNNNNNNNNNNNNNNNNNNNNNNNNNNNNNNNNNNNNNNNNNNNNNNNNNN
NNNNNNNNNNNNNNNNNNNNNNNNNNNNNNNNNNNNNNNNNNNNNNNNNNNNNNNNNNNNNNNNNNNNNNNNNNNNNNNNNNNNNNNNNNNNNNNNNNNNNNNNNNNNNN
NNNNNNNNNNNNNNNNNNNNNNNNNNNNNNNNNNNNNNNNNNNNNNNNNNNNNNNNNNNNNNNNNNNNNNNNNNNNNNNNNNNNNNNNNNNNNNNNNNNNNNNNNNNNNN
NNNNNNNNNNNNNNNNNNNNNNNNNNNNNNNNNNNNNNNNNNNNNNNNNNNNNNNNNNTTTTACGAACATTTTTTGTGTGTTTACGAACATTTACGAACAAATGTTTG
TAAAAGTATAAAAAAATGTTCGTCAAATGATCATCTTACGAACAATGTTTGTGAAAAAAGTGCAAACTTTTACGAACTTGTTTATGGGTTAGAGGTTAGACGATTCAA
GAGCATTTCGTAACTCTTCCATAGGAATTCACAAACATTTACGAACATTTTTACAAAAATATTTTTTTCTGTGTAAAATACCAAAATATAAATTGAAAATTGAGAAAA
GTAGATACGTTAAACTTTGCCCCCGTACTATACTCTTTAGGTAAATCTTGTAATAAAAATTAAATACATCTATCGTTTCTTATATCTAATATTCTTAGTAGTAATTTA
GGTAACAATTTAAATTTGTTTTGATATTCCAAGCTAATTTCGTCTCTTAGTTGAAATTGTCTTTTTATCTAAAGGCAAATGAAATTAAGGATTTTTCTAACTATACAT
TAATTCCTGGATTTTTTACTTTTTATTCTTCAACCTAATACTAATAAGCTTTTCTTTTAGTTTATCTATTTAAGAGAAGACTGACATAAAATTTAATTATGCAATTTT
ATAAGCTTGTCAAAAAATATGGAAATATTTCGTCAGACTGTCCGAATTAATTGTGTCATATTTAAAGCCAACACATTTGTATTAAATCCAATGATTTGCACTTTTCTT
CCTATACTCCCAGGAATGATACCTACATTCCGGTGTATTAATCAAAAACCTACACGGGTTGTCTATATATCTTATTCCGAGATAAAGAATTGCTAGTAGTAGGTTGTG
TTTATACAAATACAAATGCATGGATTTGACCAAGTATATAAAGTTGGAGATGGTGCTTATGGTGCTTAGAAAATATACTGTAAGCTTCAGTTCTATGGGAACATAGTC
CAGTGCCGGGTGTGTTGTGATTACCATACGCAAGTTTTTTAATAACATTCAAAACCAAAAAAATGGTGGCCTTTTCCGATCTTCTTTCCTATGACAATGTTGTCTTTA
GATCCTATGTGTTCTGGTCAACGGTTTTAATTATGAAAACTCTGGCTATGGCCTTTCTAACTGGACGTCAGCGTTTCAAAAATAAGGTACTATCATTTTGACTATGAATTA
TTGTATTACACAACCTAATACGATAACAATTATCTCACGTAGCTATATTATGTATACCATGACATTTCGATTTGAGCGCCTTAAAGTAGATTATAATTTTTTTTTAAA
TATGTTTTTCTTTAAAGGTTTTTGCCAACCCAGAAGATGCTGGAAAATATAAGGCTAAGGTCAAGTTTGACGATCCTGACGTGGAACGTGTGAGAAGGTATTTTTTCCACT
CTATAAATTTGTTAAACCTTGAAAGGTTATTACTTAAATTTGTTTTTGTAGGGCTCATCGCAATGATTTGGAAAATGTCTTCCCATTCATCCTTGTTGCATTCTTCTATG
TTCTGACAAATCCTGAAGCAGCCCTTGCTATCAATCTCTTCAGAGCTGCAGCAGTCGCAAGGATAGTCCATACCCTTGTCTACGCAGTTTTCGTTGTTCCCCAACCAGCCAG
AGGATTGGCATTTTTCGTATGCTTGGGATCTACATTGTACATGGCTTTTAAAACTTTAGTGTATTTCGCATAAAGAATCAGCGGATTTTCTATTCCATCTACTTTTGTTT
TTCTTTTTTTTTGTTACTTCTTTGGCCTTTCATCTTATCGTAATTTCTAAATACCAGGTTTTCTCTGGTCCAAAATGATTGTTATTGATAAGATAGCATTTGCACACC
ATAAACATACACGTATAAAGCTTTATAGTTTATTTTTTACATTTACATGTAACGGAAGTGTGAAGAAACTATCTTTAAAAAATATAATATAGTTCATACACTTGAGAT
TTAAAAAAAAGACTAACCTTTTATCTATCGACAGGTAATCTATTTTTCATAAACCATGCTGAAAAAAGTCCAAATCATTTTCAACGGTAATCGGCTATATCATTTTCT
GATAACTTTGGAGGAGGGCGGCTTTATCTTTAAAGTCAGTTCTATTTGGTCTGTCAATGAGTTTGGTTGTTTTTTTTTCTTTGAACTATCACTAAATATTTGAAATAC
TCGCTAAAAATAACCATGGGTGACGTCTTTGACTATCTATCCCCTACAAATCCGGTTTTTAGGACCTTTGCATTCTGGTCCAGTGTACTCGTAATAAAAGTTCTTTTT
ATGTCAATAGCTACTGGATTAAGAAAGCATACCTCAAAGGTACGTAAACCGAAATTTCGAAAATACTTTCTTACCTCATCTTGATATTACAAACAAATTCCTTAGGCG
CCAAAAGCGAAAAATGAAGTCAATGAAGATGTTGAGCGATTGAGGAGGTAAGGGAAAGGGATATGAAAGGGTTATGAGTTCTATGCATAATCATTTTTCACAATTTCA
GGGCTCATTTAAATGATATGGAAAATATCTTCCTTTTCATCATTCTGGGCTTCATTTATATCGTCACAAATCCATCTCAGTGGTTGGCTATGATGCTGATAAAAGCTTTCGC
TATCACCCGAATTCTTCATACACTTGTCTACGCTTTTGCACCGGTACCCCAACCAACGAGGGTCATTCTGTTCCTCATGGGTGTTGCAGTGAATGCCTACATGGGCTTATCA
GTTATTAGAGCATTTTTGTAAATTAAATAAAATCACAATTTGATTTTTTTCATAGTTTCTTGGTGTATTGAAAATATCATTGAAGAGATAATAGACAAAATGACGTTAT
CAAAATAAGTACTGATAAGTGAGGTTGTTTTGAACCGGTGGCAGCCAAAATTCCGAAACCCACAATCCCGAAAGCTAAAATCCCGAACGACAAAATCCCGAAAAGGCC
AAAA

>PPAI007443_a (151 aa; 3 exons) | EDITED
MANILDLLSLDNPVFRHMALWSGVLLVKMLLMSGFTAFFRIKNKAFSSPEDLIFGGKKPKYDDPSVERVRRAHRNDLENVIPLIFASFFYTLTNPTPFLAINLFRVAA
LSRIAHTAVYAFGPVPQPTRALAFGVPFVITLYMSVQVLLQFA*

>PPAI007443_b (153 aa; 3 exons) | EDITED
MTELKYIFSVENELFRTYAFWSVLLIIKMFAMVVITGYLRRKTGTMSNPEDISVAPPGSKIKFDDPDVERARRAHRNDLENIYPYILLGFGYLLTDPTPWVAALVFRV
GAIARIAHTIVYTVYVIPQPARAIAFGVCSIISYYMASQIILYFL*

>PPAI007443_c (151 aa; 3 exons) | EDITED
MVAFSDLLSYDNVVFRSYVFWSTVLIMKTLAMAFLTGRQRFKNKVFANPEDAGKYKAKVKFDDPDVERVRRAHRNDLENVFPFILVAFFYVLTNPEAALAINLFRAAA
VARIVHTLVYAVFVVPQPARGLAFFVCLGSTLYMAFKTLVYFA*

>PPAI007443_d (141 aa; 3 exons) | EDITED
MGDVFDYLSPTNPVFRTFAFWSSVLVIKVLFMSIATGLRKHTSKAPKAKNEVNEDVERLRRAHLNDMENIFLFIILGFIYIVTNPSQWLAMMLIKAFAITRILHTLVY
AFAPVPQPTRVILFLMGVAVNAYMGLSVIRAFL*

####### LLOJ010490 - GST microsomal #######
>JH689577 forward | LLOJ010490 (pre-editing) | LLOJ010490 (post-editing)
TATCACTGTACGGTACAACATATTAAATGCATACAAGTACAAGTTATATATGTTTGTAATCAGTAAACTTCGGTGATTAAATGTATAAGTACGTTTCTTTAGCTTCAT
ATACTCAAAATCTATCAAAAACATGGGCAGTGTTTGAAGAGTAGAGTCAAGTGATAGGTGTATTTTTCTACGGTCTCGAAAAACGACAAATCATGGTGGACTTTTCTGA
TATTATCTCTAATGAGAATGTTGTATTCAGAGCATATGTCTTCTGGTCAACTGTCCTTATAGTCAAAACTTTGGCTATGTCCTTTTTGACAGGACGACAGCGTTTCAAGAAT
AAGGTAAAAATATTTCTTTTTATGGATGTAATTACTCGCATATTAAGACATTCAATACCTATCTGACCTTTCTGTACTTTTTTTTTCATAAAGATGAATTATGGTGAT
TTGTCGAAATTCACTTTTTGCTTAAAAAACTAATTCCCTGTAGGATTGAATGTTTTAGCAAAATAATCATAATTCTATTTTATGTTTTATTACACAACCAACGATAAG
AATTATCTTATATATAGGTACATAATATATTTATATATAGTATGTGATTCGTTTTTGTATTTATGCGCCATAATGTAGTCTTTTTACAATTATTTTTGTCTTAGGTCT
TTGCCAATCCTGAAGATGCTGCTGGAAAAAAATTGAAGGTTAAATTCGATGATCCTGATGTAGAACGTGTAAGAAGGTATACTATAAACCACCTATAATATACTTTTGTGT
GCCAGTTCTACATACATATTTTTATCATCATTTTGCAGAGCTCATCGCAATGATTTGGAGAATATTTTCCCATTCGTCCTTGTTGCATTTTTCTACGTATTGACCAATCCT
GAACCCGCCCTGGCTATCAATCTATTTAGAGCTGCAGGGATTGCAAGAATAGTTCATACTCTCGTTTATGCAGTGTGGGTTGTGCCTCAACCTGCAAGGGGAATTGCATGGG
TTGTCTGCCTAGGATCTACCCTATATATGGCTTTTAAGTCAATTGTATTCTTCATTTAAAAAAAATTGAAGCAATATAAAATATGAAAAATGATGAACTTTACAATAATT
TTTTATTGATGTTTGAAACATCCTGGGATTAGTTAAAAATTCAAAATGACTAAGCAGATTTTCCCTATTAAAAATTCAAAGAATGAGTTGTCACTTTGCAATCGATTA
AATAATTGTTTTATATCAGAGGCCTTATATTTTCTATGGGT

>LLOJ010490 (151 aa; 3 exons) | UNCHANGED
MVDFSDIISNENVVFRAYVFWSTVLIVKTLAMSFLTGRQRFKNKVFANPEDAAGKKLKVKFDDPDVERVRRAHRNDLENIFPFVLVAFFYVLTNPEPALAINLFRAAG
IARIVHTLVYAVWVVPQPARGIAWVVCLGSTLYMAFKSIVFFI*

####### LLOJ004345 - GST microsomal #######
>JH689577 forward | **LLOJ004345** (pre-editing) | LLOJ004345_a (post-editing)
TAATATTCCACTATGAAGGTAAATGATCTATATATTTTAAAAGTAGGGGTGAACGACTATTTCTATTAAACATTTTCCCTGCACTGCATTGTTCTTTGGAAATCCAAG
CACAGTACTTGTGAGAAATCGAAGTGGTAAAGTCGTGTGCTAACAACTTAAATATCCTTAAAGATTTATTGATATTCTAAATTTAAAAAAAAATGAATAAAACTATAC
CAGCTTATTCGTTGATCTCTAGCGATAATCCAGTGTTTTCGGCATATGTTACGTGGATTTGTATTTTAACCTTAAAAATGCTTTTAATGTCCGTTCTCACAGGAACCT
TTCGTGTTAAAAATGCGGTTAGTTGTATTTAGTACGACGCGATGAAAAAGAGAATTTAATTTAAAATAAGTGTTCAATTTTGTAGGCTTTTGTTAATCCGGAAGATTT
ACCTAAGCAAGAA**ATGGAAATGAAGAGTGATCCACAGGTGGAGAGAGTTCGTCGTGCACATCTGAATGATATGGAAAATATTCTACCTTTCCTCACCATTGGGCTCCTTTA**
TGTACTAACAAATCCAAATAAAGTAATCGCCAGTAATTTATATCGAGTTGCAGCAACAGCTCGAATTATTCATACAGTTGTATACGCTCTCTACCCCATTCGTCAACCAGCT
CGTGCTATTTGCTTCTTTACATGCTACTTGATTGAAATATATATGGCAATAATGTGCATTATTAGATTTTGGTGAAATGTGCACGTGAAGTTGTGGAGGATGGCCGAAAT
TCCACCAAAATTGCAAACATTTTGCACTAAACTTTTCTCTTCTTTTAGTTATGAAAACTTGAGGAAATTTGTGAACTTTTCGAGTCAACTTAAAAAAAAAGTCAATCG
CTTAAGTTTATAGGTAAAAGTAAAGGTTTTATGTAATTAAAAGATATTTCAATATATACTATGGTATTAAATGAAAATTTTTTGATAAATAACCATCCTCCCACTCAT
ACATAAAATGTTACAACTTTATGTGAAAATTGTTGCATTTGACAAAAACGAGCATAAGATCCTTGTTAAAAATTTTATAATATAAAATCAGTTTCATTGTTTAAGAAA
ACCTAATTTAATATAAAAAAAATTTATGAGTGAGAATATTTAAATTTATTTTATTATTTATTATGTAATAATAAATTTCTATTATGTTTCATTCAATTATAAATCTAT
ATCTCTCATACATGAATGTGTAATTAGAATGCACTGAATTACATGGAAGAATAAATTCACATCAATAAAGTTTTTATTTTATATCTTTTAACCTTTACATAATAG**ACA**
TGACTATATTAATTGGACAAAATATACAAAATACGGAACCTAAAAAAACAGGACTTGAGGGACTATTGAAAAGGAATTTTAAATGACGATCCTTACTGAGACTAATGACC
AAAACGACCTTCAAGAAATGTTGCGTTGAAAAAAATAGATATCAATGATACGTGTAGATTTTAGTATCACTGTACGGTACAACATATTAAATGCATACAAGTACAAGT
TATATATGTTTGTAATCAGTAAACT

>LLOJ004345_a (153 aa; 2 exons) | EDITED
MNKTIPAYSLISSDNPVFSAYVTWICILTLKMLLMSVLTGTFRVKNAAFVNPEDLPKQEMEMKSDPQVERVRRAHLNDMENILPFLTIGLLYVLTNPNKVIASNLYRV
AATARIIHTVVYALYPIRQPARAICFFTCYLIEIYMAIMCIIRFW*

####### LLOJ002060 - GST microsomal #######
>JH689469 forward | **LLOJ002060** (pre-editing) | LLOJ002060 (post-editing)
GATGTTGTATGTTATGAAAAAAATATGTATATGTAGTACATTCATACATACATACATATCTCAAGGTGGCTAATCTTAATCCATCTGCCGCTGATTTTCTCTACGACG
CTTCCACAAAATCAATCAGAAGGTCAGTTCTTGGGGAATTGTGTTGAGTGTTGGATGTAAAATAATTTTCGGCAGTTTGAAAACGCTAAATC**ATGGTGAAGTTCTTTGA**
TATTATTTCCAATGAGAATGAAGTGTTCAGATCTTTTGCCTTTTGGTCGGTTGTTCTTCTTGTGAAAACCCTGGCTATGGCCTATCTAACAGGACGAGTACGTTGGACGAAG
AAGGTAAATTCAAGAAAATCCTTTTCTATCAGAATGATGTTTTTTTCGAAATAATTATTTTTGGAGGTCTTACACAATCTCAATTATTATCTCATTAATCGCATGACG
ATGTAAGTTCGTAAACGCCTCAATGTAGATTTTTTAAAAATAATTATTCTTCTTCCACTGTGTTAG**GTTTCCGCAAATGAAGAAGACGCCGCGAAATACAATGCAAAGAT**
CAAATTTGACGATCCCGACGTAGAACGTGTGAGACGGTATGAAAGAATTTTCACATTATGTACAACATGTGTGCTATATAGATCACAAATATATATTTTTTGTTTCACA
G**AGCCCATCGAAATGACTTGGAGAATATTTTTCCATTCATTCTCGTAGCATTCTTCTACGTGTTGACAAATCCCGAGCCAACACTTGCAATTAATCTCTTCAGAATTGCAGC**
CATTGCAAGGATCATCCATACTCTTGTCTACGCTGTGTGGGTTGTACCCCAACCTGCTCGGGGGTTGGCATTCTTCGTATGTCTCGCATCTACCCTCTACATGGCGTTCAAG
ACAATCTTGTTCTTCATGTGAAATTTGTTTATTTTTATTCTTTGTCTGTATTTAATATCCAAAAGGAGATTATTCAATCTGGGAAAGAAATTTTATTCTTAAAGGGATT
AATAAAAAGTATTTAAAACATTCAGTTTCTTATGAAGAATTTAAGAAAAATTCATTCTGAATTAATTGCAATAGCTCTGAAAAATAATTTACTGTAGTGCTTAATATG
GAAT

>LLOJ002060 (151 aa; 3 exons) | UNCHANGED
MVKFFDIISNENEVFRSFAFWSVVLLVKTLAMAYLTGRVRWTKKVSANEEDAAKYNAKIKFDDPDVERVRRAHRNDLENIFPFILVAFFYVLTNPEPTLAINLFRIAA
IARIIHTLVYAVWVVPQPARGLAFFVCLASTLYMAFKTILFFM*

####### LLOJ008423 - GST microsomal #######
>JH690025 forward | **LLOJ008423** (pre-editing) | LLOJ008423_a (post-editing)
TAATAATAATAAATTTATTGATGCCCCAAGTAATAAAATTACATAATGTGAGACTATACATTATTTTAATTTTATTTTTATATTTTATTTTTATATAAAATTTCAGTC
TGTAGAATAATTTTGTTCTCTTAAGAGAAACTGCGTGAATTTCATTTTCTAATTTTCTTCTAAGTGGATGTCGCCTATCCAATTAGTAAATTCAGCAATTTTTGGTGT
GTCACTACAATGGGTAAGAAAGACCCCAAAAACGGGAAGTGAACAGTTGAAAAAGCTCCTCCGTGGGGAAAATGCAGTGAAATGCAAGACGAAGGAATTTAATTAAAT
TAGGAGAAGAAAATGAACGGAGATTTTCCTTTTGGAAAACCCGGAGGGAGGTTGACGTGACTGTCACCTCTCATTGCCCATTCAGAGCCCCTTTTGCTCCAACAAATT
CTCTGTATGGCATTTCAGGAGGGATTGAAAAGACGTGCCCCCACTGTACGTGGTACACCAAGGACGATGTTGGATAATGGCGGTTAAATTTTGGCTTCTATTTTCTCC
CAGATGGAATATCAACACGTTGGCACAAGATTAGCATGTTCCTGTGTGTCTCTATCACTGCCAAAGCTGGCTANNNNNNNNNNNNNNNNNNNNNNNNNNNNNNNNNN
NNNNNNNNNNNNNNNCGTCTTCTTTTACAGTTTTAACGTGAATTTTATGATTTTCTATTAATAGTATTGAAAGTAAATGCGTAAAAAAAAATGATCAGAAAAAAACTC
CATGGCGGTGAATGTTTTAATGGGATTTATGAGCATGATTTGGTAGTGTTGGAGCTTACACAACCAACGATAAGAAATATTATCTTGCATTTCAATAAATATTACAAC
ACGATGGTGATGTGTAAAGGCAATGACAAAGCATTTCAAACGCCTCAATGTAGATTAAAATGATTTTTTCTTGATTTTTTTTTAGAGTTTTGCTAATCCTGAGGATTC
AGCA**ATGTACAAGACAAAACCCAAATTTGACGATCCTAATGTTGAGCGTGTACGAAG**GTATGTTACATATACTTTTTTAAATTATTTTTCTTATGCAACTTAGACGCAGA
ATTAGGGAATTTAGACACAGAATTAACAACTTAGACGCAGAATTAACAAACTTAGACGCTGAATTAACAACTTAGACGAAGAATTAACACACTTAGACGTTGAATTAA
CAAACTTAGACGCTGAATTAACAACTTAGACGAAGAATTAACAAATTTAGACGCAGAATTAACCAATTTAAACTCAGAATTAGGCAATTTAGATGCAGAATTTGCCAA
CTTAGACGCAGAATTAGATGCAGAATTAGCAACTTTAACGCAAAATTAGGGAATTTAGACGCAGTATTAACCAATTTAAACACAGAATTAACAAACTTAGACGAAGAA
TTAGGAACTTAGATGCACTGTCTGATCTGAGAATTTCACCCGAATTTCCTAATTTTTTCCAG**AGCCCATCGCAACGATTTGGAAAATATCTTCCCATTCGTCCTTATTGC**
CTTCTTCTACGTGCTAACCAATCCACAAGCTTGGCTTGCCATCAATCTCTTCAGAGTTGCAGCAATTGCCAGGATTGTGCACACTGTTGTCTACGCCGTTGTGGTTGTACCC

CAACCAGCTAGAGCTTTGGCATTCTTTGTATGCCTTGCTGTTACCCTTTACATGGCATTCCAAACAATTGTATTCTTCTTGTAAACATTAACATTAAGGATTTATATTAAA
AAAAAAGTGCACCATAAACCATTTTGCATTGTATTTCAAGGAAAAATTTGGAGTATTGAGGGGAAAACGTTTNNNNNNNNNNNNNNNNNNNNNNNNNNNNNNNNNNNN
NNNNNNNNNNNNNNGCCATTGGCAAATTCCCATAAATAACGCGCCGAAGCTATTCAATAAATCCC

>LLOJ008423_a (>107 aa; >2 exons) | EDITED (PARTIAL GENE - N-TERMINAL GAP)
SFANPEDSAMYKTKPKFDDPNVERVRRAHRNDLENIFPFVLIAFFYVLTNPQAWLAINLFRVAAIARIVHTVVYAVVVVPQPARALAFFVCLAVTLYMAFQTIVFFL*
